# Anticancer Effect of Pt^II^PHEN*SS*, Pt^II^5ME*SS*, Pt^II^56ME*SS* and Their Platinum(IV)-Dihydroxy Derivatives against Triple-Negative Breast Cancer and Cisplatin-Resistant Colorectal Cancer

**DOI:** 10.3390/cancers16142544

**Published:** 2024-07-15

**Authors:** Maria George Elias, Shadma Fatima, Timothy J. Mann, Shawan Karan, Meena Mikhael, Paul de Souza, Christopher P. Gordon, Kieran F. Scott, Janice R. Aldrich-Wright

**Affiliations:** 1 School of Science, Western Sydney University, Sydney, NSW 2751, Australia; m.elias3@westernsydney.edu.au (M.G.E.); shawan.karan@westernsydney.edu.au (S.K.); m.mikhael@westernsydney.edu.au (M.M.); c.gordon@westernsydney.edu.au (C.P.G.); 2 Medical Oncology, Ingham Institute for Applied Medical Research, Liverpool, NSW 2170, Australia; s.fatima@westernsydney.edu.au (S.F.); tim.mann@westernsydney.edu.au (T.J.M.); 3 School of Medicine, Western Sydney University, Sydney, NSW 2751, Australia; 4 Nepean Clinical School, Faculty of Medicine and Health, University of Sydney, Kingswood, NSW 2747, Australia; paul.desouza@sydney.edu.au

**Keywords:** breast cancer, colorectal cancer, cisplatin resistant, chemotherapy, platinum(II), platinum(IV), cytotoxic, mechanism, proteomics

## Abstract

**Simple Summary:**

Conventional intravenous platinum(II) chemotherapy medications, including carboplatin, oxaliplatin and cisplatin, have demonstrated remarkable efficacy in treating several forms of cancer. Also, this therapy is frequently accompanied by toxicity and development of resistance. More potent platinum complexes with fewer side effects may be of benefit. This study compares the in vitro anticancer effect of novel platinum(II) complexes and their platinum(IV)-dihydroxy derivatives with cisplatin to understand the influence of different mechanisms of action on potency. The investigation of these complexes is expected to advance our understanding to support further research into their application as cancer therapeutics.

**Abstract:**

Development of resistance to cisplatin, oxaliplatin and carboplatin remains a challenge for their use as chemotherapies, particularly in breast and colorectal cancer. Here, we compare the anticancer effect of novel complexes [Pt(1,10-phenanthroline)(1*S*,2*S*-diaminocyclohexane)](NO_3_)_2_ (**Pt^II^PHEN*SS***), [Pt(5-methyl-1,10-phenanthroline)(1*S*,2*S*-diaminocyclohexane)](NO_3_)_2_ (**Pt^II^5ME*SS***) and [Pt(5,6-dimethyl-1,10-phenanthroline)(1*S*,2*S*-diaminocyclohexane)](NO_3_)_2_ (**Pt^II^56ME*SS***) and their platinum(IV)-dihydroxy derivatives with cisplatin. Complexes are greater than 11-fold more potent than cisplatin in both 2D and 3D cell line cultures with increased selectivity for cancer cells over genetically stable cells. ICP-MS studies showed cellular uptake occurred through an active transport mechanism with considerably altered platinum concentrations found in the cytoskeleton across all complexes after 24 h. Significant reactive oxygen species generation was observed, with reduced mitochondrial membrane potential at 72 h of treatment. Late apoptosis/necrosis was shown by Annexin V-FITC/PI flow cytometry assay, accompanied by increased sub-G0/G1 cells compared with untreated cells. An increase in S and G2+M cells was seen with all complexes. Treatment resulted in significant changes in actin and tubulin staining. Intrinsic and extrinsic apoptosis markers, MAPK/ERK and PI3K/AKT activation markers, together with autophagy markers showed significant activation of these pathways by Western blot. The proteomic profile investigated post-72 h of treatment identified 1597 MDA−MB−231 and 1859 HT29 proteins quantified by mass spectroscopy, with several differentially expressed proteins relative to no treatment. GO enrichment analysis revealed a statistically significant enrichment of RNA/DNA-associated proteins in both the cell lines and specific additional processes for individual drugs. This study shows that these novel agents function as multi-mechanistic chemotherapeutics, offering promising anticancer potential, and thereby supporting further research into their application as cancer therapeutics.

## 1. Introduction

Every sixth death in the world in 2018 was due to cancer, which is the second leading cause of death globally with men more prone to suffer lung, prostate, colorectal, stomach and liver cancer, whereas women are more prone to suffer breast, colorectal, lung, cervical or thyroid cancer [1,2]. Pharmacological treatment approaches in clinics include chemotherapy, immunotherapy and targeted therapy. Platinum(II) drugs like cisplatin, carboplatin and oxaliplatin have a therapeutic effect against malignant tumours like ovarian, breast and colorectal cancers. Platinum(II) drugs were originally isolated by Rosenberg, VanCamp and Krigas, after a study of the effects of electricity on inhibiting bacterial cell growth [3]. They first investigated if electricity was the cause of the observed inhibition of cell division, but it was eventually understood that there was oxidation of the electrode to produce a platinum(II) complex, and cisplatin was responsible [4,5,6]. After several clinical trials, it became the first platinum(II) drug to be approved as a chemotherapeutic in 1978 in the U.S and in 1979 in the U.K. for ovarian and testicular cancers [6,7]. Over 40 years later, platinum(II) drugs remain among the most widely used anticancer drugs [6]; however, efficacy is limited by the development of resistance in some cancers and systemic toxicities linked to their reduced selectivity to cancer cells [8,9]. Long-term cisplatin treatments lead to various adverse effects including renal tube injury, gastrointestinal harm, neuromuscular difficulties, ototoxicity and neurotoxicity, posing significant treatment challenges [10]. Cisplatin crosslinks DNA, inducing damage, and interrupting homeostatic cell repair mechanisms, which subsequently leads to cell death by apoptosis [11]. Many tumours have developed resistance mechanism that prevents cisplatin from being taken up by the cell, this reduces cellular accumulation and decreases platinum–DNA adduct formation, which reduces its cytotoxic activity [12,13,14].

**Scheme 1:**
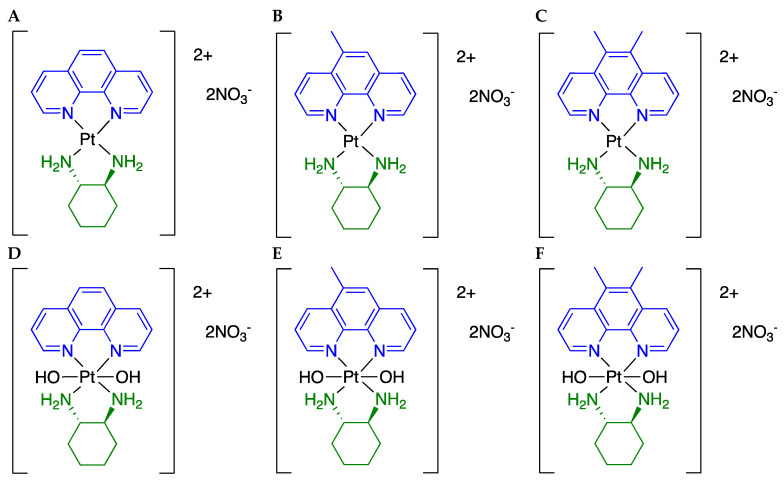
Chemical structures of **PtIIPHEN*SS*** (**A**), **PtII5ME*SS*** (**B**), **PtII56ME*SS*** (**C**), **PtIVPHEN*SS*(OH)_2_** (**D**), **PtIV5ME*SS*(OH)_2_** (**E**) and **PtIV56ME*SS*(OH)_2_** (**F**).

These impediments have initiated the search for other metal-based drugs with different characteristics. A promising approach to mitigating some of the barriers of platinum(II) drug therapy is to create platinum(IV) derivatives of these complexes [15,16,17,18,19,20,21]. Platinum(IV) complexes have six-coordinate octahedral geometry (low spin d^6^ electron configuration), where the two axial positions are available to coordinate additional bioactive ligands. The octahedral geometry is advantageous to the intracellular activity of the platinum(IV) complexes because it alleviates undesired biomolecular interactions due to their stability in the bloodstream, which limits toxic side effects and improves selectivity for cancer cells [22]. Oral administration would be a clinical advantage for cancer patients, with an improved experience of receiving treatment, coupled with a reduced cost [23]. Satraplatin was the first platinum(IV) chemotherapeutic drug to be orally administered in phase III clinical trials, although it ultimately failed the trial as there was no clear improvement in survival benefits [24,25]. Our team has synthesised and characterised platinum(II) and platinum(IV) dihydroxy complexes that have exhibited promising anticancer activity against several different cancers [17]. These include complexes [Pt(1,10-phenanthroline)(1*S*,2*S*-diaminocyclohexane)](NO_3_)_2_ (**Pt^II^PHEN*SS***), [Pt(5-methyl-1,10-phenanthroline)(1*S*,2*S*-diaminocyclohexane)](NO_3_)_2_ (**Pt^II^5ME*SS***) and [Pt(5,6-dimethyl-1,10-phenanthroline)(1*S*,2*S*-diaminocyclohexane)](NO_3_)_2_ (**Pt^II^56ME*SS***) along with their dihydroxy platinum(IV) derivative complexes [Pt(1,10-phenanthroline)(1*S*,2*S*-diaminocyclohexane)(dihydroxy)](NO_3_)_2_ (**Pt^IV^PHEN*SS*(OH)_2_**), [Pt(5-methyl-1,10-phenanthroline)(1*S*,2*S*-diaminocyclohexane)(dihydroxy)](NO_3_)_2_ (**Pt^IV^5ME*SS*(OH)_2_**) and [Pt(5,6-dimethyl-1,10-phenanthroline)(1*S*,2*S*-diaminocyclohexane)(dihydroxy)](NO_3_)_2_ (**Pt^IV^56ME*SS*(OH)_2_**) (Scheme 1). The addition of a 5-methyl and a 5,6-dimethyl group compared to the complex **Pt^II^PHEN*SS*** results in increased potency, which has been observed across several cancers [17]. Hitherto, **Pt^II^56ME*SS*** has been observed to elicit significantly enhanced biological activity compared to established chemotherapeutic agents, cisplatin, oxaliplatin and carboplatin, with its mechanism of action shown to change the cytoskeleton organisation and reduce mitochondrial membrane potential [26]. Given their structural similarities, we hypothesise that **Pt^II^PHEN*SS*** and **Pt^II^5ME*SS*** and their Pt^IV^ derivatives will have similar mechanistic effects. 

This study aims to explore the comparative anticancer activity and mechanism of action of the six complexes (Scheme 1) against triple-negative breast cancer (TNBC) (MDA−MB−231 cell line) and cisplatin-resistant colorectal cancer (HT29 cell line) relative to cisplatin. TNBC is a type of breast cancer that does not express human epidermal growth factor receptor 2 (HER2), estrogen (ER) or progesterone (PR) [27]. It is an aggressive form of cancer responsible for 15–20% of breast cancers [28]. Poor prognosis is primarily due to the highly invasive nature and metastasis to secondary organs such as the brain, lungs and bone [29]. Genetic history is a major risk factor for breast cancer, with 13–19% of patients reporting increased risk in family members less than 50 years of age who express BRCA1, BRCA2 and PALB2 mutants [28,30]. Other risk factors include longer duration of oral contraceptive use and higher breast density [28,31]. TNBC is commonly treated with chemotherapies such as taxanes (anti-microtubule agent), fluorouracil (anti-metabolite), cyclophosamide (alkylating agent) and anthracyclines (DNA intercalating agent) [28,32,33,34]. Cisplatin has been used as a neoadjuvant, and in combination with these drugs, to treat TNBC [35,36]; however, whether used together or separately drug resistance often occurs^−^ [37,38]. 

Colorectal cancer (CRC) is the third leading cause of cancer death globally [39]. CRC development is in response to diverse mutations and mutagens, so it is challenging to design a specific molecular therapeutic. Surgery (local excision) has been the primary course of treatment in early diagnosis, but is not effective in cases of metastasis, so neoadjuvant cytotoxic chemotherapies such as fluorouracil, cisplatin and oxaliplatin are used [39,40,41,42,43,44]; however, drug resistance and reoccurrence occur [39,45]. As with breast cancer, genetic history is a risk factor linked to CRCs, with 7–10% being hereditary-related cases, while the major risk factors are prophylactically linked to diet and lifestyle. These are exemplified by high-fat diets, low fibre, inactivity and smoking [46,47].

Accordingly, the mechanism(s) of action of the platinum complexes (Scheme 1) will be assessed in MDA−MB−231 and HT29 cancers by determining the mode and localisation of uptake, cellular reactive oxygen species production, the influence on the mitochondrial membrane potential and the type of cell death by flow cytometry, immunofluorescence and through proteomic studies. This will better define the intracellular targets of these complexes and their potential for further drug development. Future investigations of the complexes in vivo will be dependent on the outcomes of these in vitro studies.

## 2. Materials and Methods

### 2.1. Chemicals and Reagents 

Dulbecco’s Modified Eagle’s Medium (DMEM), (with 4.5 g/L glucose/L-glutamine/sodium bicarbonate/sodium pyruvate, liquid, sterile-filtered) (Invitrogen #11995-073), Dulbecco’s Modified Eagle’s Medium (DMEM), no phenol red (Invitrogen #21063-029), Dulbecco’s Modified Eagle Medium/Nutrient Mixture F-12 HEPES (DMEM/F-12) (Invitrogen #11330-057), Roswell Park Memorial Institute medium (RPMI) (Invitrogen #11875-119), Dulbecco’s Phosphate Buffered Saline (PBS) 1× (with MgCl and CaCl_2_) (Invitrogen #14040-182), Foetal Bovine Serum (FBS) (Invitrogen #26140-079), Horse Serum (HS) (Invitrogen #16050-122), Trypsin-EDTA 10× (Invitrogen #15400-054), Penicillin–streptomycin (P/S) solution (5000 U/mL) (Invitrogen #15070-063), PageRuler Prestained Protein Ladder (Invitrogen #26617) and SeeBlue Prestained Protein Standard (Invitrogen #LC5625) were purchased from Thermo Fisher Scientific (Brisbane, Australia), unless otherwise stated. MEGM^TM^ Mammary Epithelial Cell Growth Medium SingleQuots^TM^ Kit # CC-4136 was purchased from Lonza, Australia. A MilliQTM system (Millipore Australia Pty Ltd., Sydney, NSW, Australia) provided the deionised water (d.i.H2O) needed for the studies. All chemicals and reagents were of spectroscopic grade and used without additional purification. Methanol was purchased from Honeywell Research Chemicals, NJ, USA, Ammonium Bicarbonate, Dithiothreitol (DTT), Chloroform, RNase A, Triton X-100, Sodium Chloride (NaCl), Tris Base, Bovine Serum Albumin (BSA), Sucrose and D-phenylalanine were purchased from Sigma-Aldrich, Bayswater, VIC, Australia. Cell Proliferation Reagent WST-1 was from Roche Diagnostics, Indianapolis, IN, USA. 10× Tris/Glycine/SDS (TGS10×), 10× Tris/Glycine Buffer (TG10×), 10× Tris Buffered Saline (TBS10×), and 4× Laemmli Sample Buffer were purchased from Bio-Rad (Gladesville, NSW, Australia). Anti-Rabbit secondary antibody (horseradish peroxidase (HRP) Linked) (#7074S) and Anti-Mouse secondary antibody (HRP-linked) (#7076S) were purchased from Cell Signaling Technology, Danvers, MA, USA. Unless otherwise noted, all antibodies were acquired from Abcam (Cambridge, MA, USA). All cell culture flasks and plates were purchased from Corning Inc. (Gilbert, AZ, USA), unless otherwise specified.

### 2.2. Cell Line Maintenance and Complex Synthesis 

Triple-negative breast cancer cells (MDA−MB−231) (HTB-26), colorectal cancer cells (HT29) (HTB-38), breast cancer cells (MCF−7) (HTB-22) and breast epithelial cells (MCF10A) (CRL-10317) were purchased from the American Type Culture Collection (ATCC, Manassas, Virginia, USA). Ovarian cancer cell lines A2780 (no. 93112519) and ADDP (no. 93112517) were purchased from Sigma-Aldrich, VIC, Australia. MDA−MB−231, MCF−7 and HT29 cells were cultured in DMEM supplemented with 10% FBS and 1% P/S. A2780 and ADDP were cultured in RPMI supplemented with 10% FBS and 1% P/S. MCF10A were cultured in DMEM/F12 supplemented with 5% HS, 1% P/S, EGF (20 ng/mL), hydrocortisone (0.5 mg/mL), cholera toxin (100 ng/mL), insulin (10 μg/mL), P/S (100× solution), (Lonza #CC-4136 MEGM kit without gentamicin/amphotericin solution). All cell lines were grown in a humidified incubator with 5% CO_2_ at 37 °C.

Synthesis and characterisation of **Pt^II^PHEN*SS*, Pt^II^5ME*SS***, **Pt^II^56ME*SS***, **Pt^IV^PHEN*SS*(OH)_2_**, **Pt^IV^5ME*SS*(OH)_2_**, **Pt^IV^56ME*SS*(OH)_2_**, (Scheme 1) were accomplished according to published methods by Aldrich-Wright Research Group as reported in Supplementary Method S2 and Result S2 [17,48,49,50,51].

### 2.3. Cytotoxicity of Platinum(II) and (IV) Complexes 

Cells were seeded in 96-well flat-bottom culture plates at a concentration of 1000 cells per well. The platinum complexes, and cisplatin, were assessed for their cytotoxicity using ten 3-fold dilutions starting from 150 to 0.007 µM. After 72 h, the medium was removed and replaced with new medium. The cell viability reagent WST-1 (10 µL, Roche Diagnostics, Indianapolis, IN, USA) was added to each well. After an hour, the absorbance was measured at 450 nm using a versatile microplate reader (Bio-strategy, Campbellfield, VIC, Australia). Three separate experiments were performed, each with *n* = 3 replicates. GraphPad prism 10 was used to calculate apparent IC_50_ values using the nonlinear regression model Y = Bottom + (Top-Bottom)/(1 + 10^(X-LogIC50)^) where Top and bottom are plateaus in the y-axis (% survival). The R^2^ value of the curve fits ranged from 0.8972 to 0.9794.

### 2.4. Cytotoxicity Evaluation of Platinum(II) and (IV) Complexes in a Cancer Spheroid Model

To evaluate the cytotoxic effect of platinum(II) and (IV) complexes in 3D cell culture, MDA−MB−231 or HT29 cells were used to bioprint networks or spheroids with the non-contact drop-on-demand 3D RASTRUM bioprinter (Inventia Life Science, Alexandria, NSW, Australia) [52,53]. The 3D cultures use two constituents: bioink and activator. Cell pellets (2 × 10^6^ cells) were resuspended in an activator reagent which forms an instant gel after its addition to the bioink by the bioprinter. The 3D large plug cell model with polyethylene glycol bioinks formulated to mimic the properties of in vivo tissue were chosen as follows. Matrix Px02.31 (activator F177 and bioink F242 with RGD: Fibronectin) was used for MDA−MB−231 3D embedded networks, Px02.00 (activator F177 and bioink F119 without RGD additives, Table S1) was used for MDA−MB−231 spheroids and matrix #Px02.09 (activator F177 and bioink F236 with adhesion peptides including, RGD: Fibronectin, and GFOGER: collagen type I to recapitulate rich in vivo tissue extracellular environments) was used for HT29 spheroids (Inventia Life Science) (Table S1). Each cell line was printed at 10,000 cells/well and loaded with the bioink fluids into the selected printer cartridge. After the automated priming of all fluids into the printer nozzles, each cell line was individually printed on tissue culture plates (Nunc^TM^ MicroWell^TM^ 96-Well, Nunclon Delta-Treated, Flat-Bottom Microplate, ThermoFisher #167008), with RASTRUM creating the 3D Large Plug across the 96-well plate. Following completion of the print run, 200 μL DMEM culture medium was added to each well using multichannel pipettes. The plate was then incubated for 7 days (humidified incubator with 5% CO_2_ at 37 °C) for compact and homogenous spheroid formation before the cytotoxicity assay [52,54]. When the spheroids developed, post-7-day incubation, media was removed and replaced with 100 µL DMEM. Platinum complexes and cisplatin were assessed for their individual cytotoxicity using ten 3-fold dilutions ranging from 150 to 0.007 µM. Cytotoxicity was then assessed using the WST-1 assay as previously described in Section 2.3.

### 2.5. Cellular Uptake of Platinum(II) and (IV) Complexes

A final concentration of 10^6^ cells/well of MDA−MB−231 and HT29 cells were seeded in 6-well plates and incubated overnight to adhere. The cells were then treated with a final concentration of 3 µM of either Pt^II^ or Pt^IV^ complexes. After 0, 0.5, 1, 3, 6, 12, 24 or 30 h, the medium was removed, and the cells were washed three times with cold PBS and allowed to dry. Then, 400 µL of 69% HNO_3_ (Baseline grade nitric acid with PPB level reported impurities, Seastar Chemicals, Sidney, BC, Canada) was added to each well for 90 min to ensure complete digestion. The digests were then moved to 15 mL centrifuge tubes (Labcon, Petaluma, CA, USA), to which 7 mL Milli-Q water was then added, which resulted in a final acid concentration of 3.5–4%. Iridium 193 (^193^Ir) was mixed online and introduced into the ICP-MS to check uptake efficiency and matrix interference. All ICP-MS parameters are described in Table S2 and elaborated in Method S3. The results represent an average of three different experiments run in triplicate (±SEM) and expressed as nmol/10^6^ cells or µM/cell. Quantification of the cellular uptake of Pt was based on external standards (Certified Standard from Sigma-Aldrich, VIC, Australia) containing Internal standard Ir. The calibration curve is shown in Figure S1.

### 2.6. Mode of Uptake of Platinum(II) and (IV) Complexes

MDA−MB−231 and HT29 cells (10^6^ cells/well) were seeded in 6-well plates and incubated overnight. Cells were then treated with 3 µM of either Pt^II^ or Pt^IV^ complexes in the following conditions, to establish the mode of uptake. Exploratory experiments were used to estimate the incubation times. To investigate the impact of temperature, cells were cultured in DMEM media with Pt^II^ or Pt^IV^ complexes for 2 h at 4 °C or 37 °C [9]. To impede transferrin receptor (TfR)-mediated uptake, the cells were pre-treated with 1 µg/mL of anti-transferrin antibody for 2 h [9], prior to the addition of Pt^II^ or Pt^IV^ complexes (3 mM) followed by another 2 h incubation. To block Clathrin-mediated endocytosis, the cells were pre-treated with sucrose (0.45 M final concentration) in serum-free medium for 30 min [55,56] prior to incubation with Pt^II^ or Pt^IV^ complexes (3 µM) for 2 h. To inhibit the SLC7A5 transporter, cells were treated with D-phenylalanine (1 mM) [57] for 2 h and then treated with Pt^II^ or Pt^IV^ complexes (3 µM) and incubated for 2 h. The media was then removed, and intracellular uptake of platinum was measured by ICP-MS, as described in Section 2.5.

### 2.7. Cellular Localisation of Platinum(II) and (IV) Complexes

MDA−MB−231 and HT29 cells were seeded at a final concentration of 10^6^ cells/well in 6-well plates and incubated overnight. Cells were then treated with 3 µM of either Pt^II^ or Pt^IV^ complexes for 24 h. The media was next removed, and cells were washed three times with ice-cold PBS. Then the cells were trypsinised. The isolation of individual fractions was carried out using the Fraction-PREP Cell Fractionation kit (ab288085, Abcam, Cambridge, MA, USA) [26]. The cell pellet was resuspended in ice-cold PBS and moved to Eppendorf tubes to spin for 5 min at 700× *g* and supernatant was removed. The pellet was then resuspended in 400 µL of cytosol extraction buffer mix and incubated for 20 min on ice. Next, samples were centrifuged at 700× *g* for 10 min and the supernatant was collected as the cytosolic fraction. The pellet was then resuspended in 400 µL of ice-cold membrane A extraction buffer mix and vortexed for 10 s, after which membrane B extraction buffer mix was added and incubated on ice for 1 min. The sample was then vortexed and centrifuged for 5 min at 1000× *g*. The supernatant having the membrane/particulate fraction was then transferred to cooled Eppendorf tubes. The pellet was then resuspended in 200 µL of ice-cold nuclear extraction buffer, vortexed for 20 s and then incubated on ice for 40 min with constant vortexing every 10 min. The samples were then centrifuged for 10 min at maximum speed. The supernatant was collected as the nuclear fraction into cooled Eppendorf tubes and the pellet was the cytoskeletal fraction. The sample buffers of all fractions were then evaporated at 100 °C and then incubated with 180 µL of 69% HNO_3_ for 1.5 h. Milli-Q water was next added to a final volume of 3 mL. Platinum cell localisation was then measured via ICP-MS, as described in Section 2.5. 

### 2.8. Cell Death Analysis

Annexin V-fluorescein isothiocyante (Annexin V-FITC) and Propidium Iodide (PI) staining (ab14085, Abcam, Cambridge, MA, USA) were used to analyse cells at 72 h of treatment for each complex. MDA−MB−231 and HT29 cells were seeded at a concentration of 2 × 10^5^ cells/well in a 6-well plate and treated with IC_50_ concentration and incubated for 72 h. The supernatant was transferred to correspondingly labelled conical tubes on ice. The cells were trypsinised and the detached cells were moved to allocated tubes. Cells were then counted under the inverted microscope (Nikon Eclipse TS100) in a 1:1 mix with trypan blue. A final concentration of 500 cells/µL was transferred into Eppendorf tubes and centrifuged at 500× *g* for 5 min at 4 °C. After discarding the supernatant, the pellet was suspended in 100 µL of 1× Annexin V Binding Buffer. Each sample was transferred to round bottom polystyrene tubes (Interpath services, Somerton, VIC, Australia). To each tube, 2 µL of Annexin V-FITC and 2 µL of PI were added and allowed to sit in the dark for 10 min. Cells were then analysed using the BD FACSCanto II Benchtop Flow Cytometer (Biosciences, Erembodegem, Belgium). The Annexin V/PI data were analysed on FL1-H vs. FL2-H scatter plot using FlowJo™ v10.9 software. The experiment was repeated in three independent experiments in triplicates.

### 2.9. Cell Cycle Arrest

Propidium Iodide (PI) staining (Abcam, Cambridge, MA, USA) was used to analyse cell cycle arrest at 72 h post-treatment of each complex. MDA−MB−231 and HT29 cells were seeded at a concentration of 2 × 10^5^ cells/well in a 6-well plate and treated with IC_30_ concentration determined for 72 h of treatment of each complex and incubated. After 72 h, the supernatant from each well was transferred to correspondingly labelled conical tubes on ice. Cells were trypsinised, and the detached cells were transferred to allocated tubes. Cells were then washed with PBS twice and resuspended in 70% ethanol and stored at 4 °C overnight. The fixed cells were then washed with PBS twice and stained with 50 ug/mL of PI in 10 mM Tris-Cl, pH 8.0, 10 mM NaCl, 0.1% Triton X-100, and 100 μg/mL RNase A for 45 min [26,58]. The cell cycle profile was then analysed using the BD FACSCanto II Benchtop Flow Cytometer (Biosciences, Erembodegem, Belgium). The data were analysed using FlowJo™ v10.9 software. The experiment was repeated in three independent experiments in triplicates.

### 2.10. Reactive Oxygen Species Detection Assay

DCFDA/H2DCFDA-cellular ROS Assay Kit (Abcam, Cambridge, MA, USA) was used to investigate the production of ROS in treated cells, as previously described [9,16,17,18]. MDA−MB−231 or HT29 cells were seeded at a total concentration of 2500 cells/well in 96-well plates and incubated overnight. DMEM was then removed, and cells washed with 1× kit buffer, and stained with 25 µM 2′,7′-dichlorofluorescein diacetate (DCFH-DA) for 45 min. The cells were then treated with IC_50_ drug concentration for each investigated agent after removing the DCFH-DA, washing with 1× kit buffer, and adding phenol red-free media (Invitrogen #21063-045). After that, the plates were scanned using the Glo-Max^®^-Multimode microplate reader (Promega Corporation, Alexandra, VIC, Australia) at an excitation/emission of 485/535 nm to detect fluorescence (relative fluorescence units, or RFU) at various time points. A positive control was generated using 20 µM *tert*-butyl hydroperoxide (TBHP), and the same procedure was applied with DCFDA. Three independent experiments in triplicates were implemented.

### 2.11. Mitochondrial Membrane Potential 

A TMRE-Mitochondrial Membrane Potential Assay Kit (Abcam, Cambridge, MA, USA) was used to study the MtMP changes in treated cells, as previously described [18,19]. A total of 2500 cells/well of MDA−MB−231 or HT29 cells were seeded in 96-well plates. Cells were then treated with an IC_50_ drug concentration for each complex. At 24, 48 or 72 h, DMEM was removed, and cells were washed with PBS. Then, cells were incubated with 1 µM tetramethylrhodamine, ethyl ester (TMRE) stain for 30 min. TMRE was then removed, and cells were washed twice with PBS (0.2% BSA), after which phenol red-free media was added. After that, the plates were immediately scanned using the Glo-Max^®^-Multimode microplate scanner (Promega Corporation, Alexandra, VIC, Australia) at an excitation/emission of 549/575 nm to detect fluorescence (expressed as RFU). The positive control was generated using 20 µM carbonyl cyanide 4-(trifluoromethoxy) phenylhydrazone (FCCP)) incubated on the cells for 10 min. The cells were then stained with TMRE, as described above, and the resulting solution was scanned for fluorescence. Three independent experiments in triplicates were implemented.

### 2.12. Immunofluorescence Morphological Changes in Microtubule Organisation Using Confocal Microscopy

To study the change in expression and morphology of actin and tubulin in treated cells, MDA−MB−231, HT29 or MCF10A cells (1000 cells/well) were seeded on Millicell 8-well chamber slides (Merck, Darmstadt, Germany) overnight. Cells were then treated with IC_50_ concentration of each complex for 72 h, except for MCF10A, which was treated with IC_50_ of MDA−MB−231. Supernatant was then removed, and cells were washed with PB (75 mM disodium phosphate, 25 mM monosodium phosphate, pH 7.4) for 5 min. PB was removed and cells were then fixed with 200 µL of 4% paraformaldehyde (PFA) in PBS for 10 min. PFA was then removed, cells were washed for 5 min with 250 µL of PB, which was removed, and cells were then permeabilised with 200 µL of 0.2% Triton X-100 in PBS for 10 min. Triton X-100 was removed with cells again washed with 200 µL of PB for 5 min, then PB was removed, and cells blocked with 1% Bovine Serum Albumin (Sigma-Aldrich, VIC, Australia) in PB for 30 min. Blocking solution was then removed and cells were washed once with 1× PBS and twice with 0.1% PBS-Tween. For immunofluorescence staining, cells were then stained for 1 h at room temperature with 100 µL/condition of β-tubulin antibody (Rabbit mAb (9F3) Alexa Fluor(R) 488 Conjugate, Cell Signaling Technology, Danvers, MA, USA) with a 1/100 dilution prepared using 0.5% BSA solution. Cells were then washed twice with 0.1% PBS-Tween and then stained with phalloidin antibody (iFluor 555 reagent, ab176756, Abcam, Cambridge, MA, USA) for 1 h at room temperature with 100 µL/condition. Next, cells were washed with 0.1% PBS-Tween twice, the chamber was removed off the slide, and cells were mounted with ProLong^™^ Gold Antifade Mountant with DAPI (Thermo Fisher, Eugene, OR, USA). Then, coverslips were added and sealed to the slide with clear nail polish and allowed to air-dry. The cells were observed under the Zeiss LSM 800 confocal microscope. For fluorescence intensity measurements, imaging parameters were kept the same for all conditions and were taken at a 20× magnification. Actin and tubulin expression was analysed using CellProfiler^™^ software 4.2.5. For morphological changes, images were taken at a 63× magnification objective. Images were acquired with an Airyscan detector and processed using the Zen Blue Airyscan processing module. *n* = 30 cells were analysed.

### 2.13. Wound Healing Assay 

Cells were seeded at a density of 10^5^ cells/well in an Incucyte^®^ Imagelock 96-well plate (Sartorius, Göttingen, Germany) and incubated overnight. The Incucyte^®^ 96-Well ESSEN Bioscience Woundmaker Tool (Sartorius, Göttingen, Germany) was then used to simultaneously make wounds in all 96 wells as previously described. Following wound formation, the media from each well were removed and cells were meticulously washed with PBS twice. DMEM was then added, and cells were treated with IC_50_ drug concentration for each complex for 72 h. The plate was then incubated in the Incucyte^®^ Live-Cell analysis system for scanning for 72 h (objective: 10×, channel selection: phase contrast, scan type: scratch wound, scan interval: every 4 h). After 72 h, data were processed and interpreted using the Incucyte^®^ Scratch Wound Analysis Software Module (Cat. No. 9600-0012). Three independent experiments were implemented, and each sample was run in triplicate.

### 2.14. Western Blot

Cancer cells were seeded at a final concentration of 2 × 10^5^ cells/well in 6-well plates and incubated with IC_50_ concentration of the platinum complexes for 72 h. Protein extraction was then performed on ice. Media were collected in Eppendorf tubes, and centrifuged at 500× *g* for 5 min at 4 °C. Meanwhile, 200 µL of lysis RIPA buffer, made up of 150 mM NaCl, 50 mM Tris-HCl, pH 8.0, 0.1% Triton X-100, 0.5% sodium deoxycholate, 0.1% SDS and 1 mM sodium orthovanadate, was added to each well, the cells were scrapped into the buffer, and added to the media-derived pellets. The tubes were placed on the shaker for 10 min at 4 °C. Then, cell extracts were centrifuged at 12,000× *g* for 15 min at 4 °C, and the supernatants (containing the proteins) were collected [9]. The Bio-Rad-DC protein assay (Bio-Rad, Hercules, CA, USA) was used to assess protein content. Laemmli protein sample buffer (4×) with *β*-mercaptoethanol (9%) was used to prepare a final protein concentration of 20 µg. The samples were then heated at 100 °C for 5 min. A final volume of 30 µL of each sample was added per well and subjected to SDS-PAGE using Bolt 4–12% Bis-Tris gel (Thermo Fisher, Eugene, OR, USA) at 80 V for 30 min and then 120 V for 60 min with PageRuler™ Prestained Protein or SeeBlue Prestained Protein Ladder (Thermo Fisher, Eugene, OR, USA). Proteins were subsequently transferred to a 0.2 µm PVDF membrane (Amersham, Cytiya) using a semi-dry blot transfer. The membrane was then blocked in 5% BSA in TBST, for 1 h and probed overnight at 4 °C with primary antibodies against several cell proliferation, apoptotic, anti-apoptotic, microtubule and autophagic protein markers listed in Table S7 as per the manufacturer’s protocol. The primary antibody was then removed, washed three times for 10 min with TBST and then membranes were treated with either mouse or rabbit HRP-coupled secondary antibodies (1/3000) for 1 h and 30 min. Protein band detection was then performed using the chemiluminescence ECL kit (Thermo Fisher, Eugene, OR, USA). Blot images were obtained using the Odyssey^®^ FC imaging system (LI-COR Biosciences, Lincoln, NE, USA) and band intensities were quantified relatively to glyceraldehyde-3-phosphate dehydrogenase (GAPDH) and then normalised each replicate gel to its corresponding no treatment control using the ImageJ v1.53t software (National Institutes of Health, Bethesda, MD, USA). Each marker results from the same experiment or parallel experiment where the gels were processed in parallel. Three independent experiments per marker were conducted.

### 2.15. Proteomics 

A total of 2 × 10^5^ cells/well of MDA−MB−231 or HT29 cells were seeded in DMEM in 6-well plates. and incubated with IC_50_ concentration of the platinum complexes for 72 h. The supernatant was then collected into 15 mL tubes, then the cells were washed with PBS and trypsinised. The detached cells were transferred to allocated tubes and centrifuged at 500× *g* for 5 min at 4 °C. Cell pellet was washed twice with PBS and then resuspended in 300 µL cell lysis buffer (RIPA) for 30 min on ice. Cell extracts were then centrifuged at 12,000× *g* for 15 min at 4 °C, and the supernatants (containing the proteins) were collected into Lo-Bind Eppendorf. The Bio-Rad protein assay (Bio-Rad, Hercules, CA, USA) was used to assess protein content. For protein extraction and delipidation 100 µL of the cell homogenate was moved to 1.7 mL Lo-Bind Eppendorf tubes and 450 µL methanol was added to the mixture and vortexed. Chloroform (150 µL) was next added and vortexed. Then, water (450 µL) was added to the mixture, vortexed and centrifuged for 5 min at 12,000× *g*, which formed the protein pellet at the organic/aqueous interface. The layer of chloroform was removed and then 400 µL of methanol was added. The sample was then vortexed and centrifuged at 12,000× *g* for 15 min. The supernatant was then aspirated and discarded. The pellet was then washed with methanol (400 µL). The supernatant was removed, and the pellets were air-dried overnight. Next, steps were performed for reduction, alkylation, and tryptic digestion of the protein. 0.1% RapiGest (Waters, Milford, MA, USA) in 50 mM aqueous ammonium bicarbonate (20 µL) was added to the air-dried pellet and vortexed for effective suspension. The pellet should completely dissolve. Then, 50 µL of 7 mM DTT in 50 mM aqueous ammonium bicarbonate was added, to give a final concentration of 5 mM DTT. Samples were then heated for 30 min at 60 °C and cooled to room temperature. Iodoacetamide (45 mM) in 50 mM aqueous ammonium bicarbonate was next added to give a concentration of 15 mM. The mixture was allowed to react in the dark for 30 min. Then, 10 ng/µL of Trypsin Gold, Mass spectrometry grade (Promega, NSW, Australia) was added for digestion to occur at room temperature overnight. And 50 µL of 4% (*v/v*) aqueous trifluoroacetic acid was then added to each tube. Samples were next heated for 45 min at 37 °C to effect decomposition of RapiGest. Samples were then centrifuged at 12,000× *g* for 10 min at 4 °C. Supernatants were then transferred to total recovery vials and run on a nanoAcquity Ultra-Performance Liquid Chromatography (UPLC) coupled with a Synapt G2-Si instrument (Waters, MA, USA) was used for label-free quantitative protein profiling. Nano-proteomics method is detailed in Method S1. Three independent experiments in triplicates were implemented, while all samples were analysed in a single run with a single injection (1 µL) for each trial. Mass spectrometry (MS) data were processed using Progenesis QI for proteomics (version 4.1, Nonlinear Dynamics, Newcastle upon Tyne, UK), and aligned using UniProt Homo Sapiens reference database IDEP9.6 utilised to cluster and retrieve the initial signature analyses [59]. To identify the differentially expressed proteins under the effect of specific treatments, a differential expression analysis (DEA) of the normalised data was carried out using the limma R package; differentially expressed proteins (DEP) were assessed with a false discovery rate (FDR) < 0.01 and FC > 2 [60]. The most significantly differentially expressed proteins in the triplicate samples in both cell lines were visualised as a volcano plot. Upset plots were generated using the R package UpSetR [61]. Enrichment studies were performed using gene lists that corresponded to the identified proteins. Overrepresentation analysis was performed based on Gene Ontology (GO) Biological Processes, GO Cellular Components, and GO Molecular Functions using iDEP. Enrichment maps and STRING apps were used to construct pathways networks and explore protein–protein interactions [62,63,64].

### 2.16. Statistical Analysis 

Data were presented as Mean ± SEM from three independent trials, and statistical analysis used for each data set is described in Figure legends. Group differences were considered statistically significant if * *p* < 0.05, ** *p* < 0.01, *** *p* < 0.001 and **** *p* < 0.0001 in comparison to the control group, unless otherwise stated. 

## 3. Results

### 3.1. Cytotoxicity of Platinum(II) and (IV) Complexes

**Figure 1:**
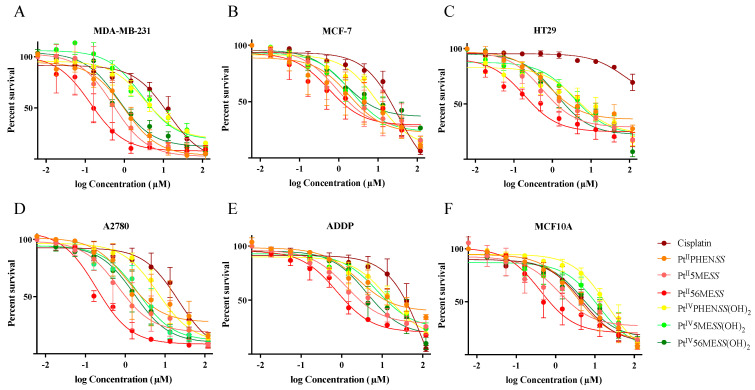
Effect of platinum(II) and platinum(IV) complexes on the survival of cancer and normal epithelial cells: (**A**). MDA−MB−231, (**B**). MCF−7, (**C**). HT29, (**D**). A2780, (**E**). ADDP, (**F**). MCF10A. Cells were treated with 3-fold dilutions of the different agents Cisplatin, **Pt^II^PHEN*SS***, **Pt^II^5ME*SS***, **Pt^II^56ME*SS***, **Pt^IV^PHEN*SS*(OH)_2_**, **Pt^IV^5ME*SS*(OH)_2_** or **Pt^IV^56ME*SS*(OH)_2_** starting with a concentration of 150 µM and assayed for cell viability, as described in Section 2.3. Data points denote mean ± SEM from three independent experiments where samples were run in triplicate.

The cytotoxic effect of Pt^II^ and Pt^IV^ complexes was examined on MCF−7, MDA−MB−231, HT29, A2780 and A2780CisR (ADDP) cancer cells and MCF10A epithelial cells 72 h post-treatment (Figure 1 and Table 1). Both Pt^II^ and Pt^IV^ complexes exhibited more potent cytotoxicity compared to cisplatin (measured by Student’s *t*-test) across the cancer cell lines as follows. The potency of **Pt^II^PHEN*SS*** relative to cisplatin was highest in HT29 cells (102-fold, *p* < 0.0001) > ADDP (44-fold, *p* < 0.0001) > A2780 (19-fold *p* < 0.01) ~ MCF−7 (18-fold, *p* < 0.001) ~ MDA−MB−231 (13-fold, *p* < 0.01). **Pt^IV^PHEN*SS*(OH)_2_** exhibited an order of cell line potency relative to cisplatin of HT29 (17-fold, *p* < 0.0001) > ADDP (8-fold, *p* < 0.0001) > MDA−MB−231 (4-fold, *p* < 0.01) ~ MCF−7 (3-fold, *p* < 0.001) = A2780 (3-fold, *p* < 0.01). **Pt^II^5ME*SS*** order of cell line potency relative to cisplatin was similar to that of **Pt^II^PHEN*SS***; HT29 (149-fold, *p* < 0.0001) > ADDP (85-fold, *p* < 0.0001) > MCF−7 (38-fold, *p* < 0.001) ~ A2780 (33-fold, *p* < 0.001) > MDA−MB−231 (25-fold, *p* < 0.01). **Pt^IV^5ME*SS*(OH)_2_** exhibited a cell line order of potency relative to cisplatin of HT29 (21-fold, *p* < 0.0001) > ADDP (15-fold, *p* < 0.0001) = MCF−7 (15-fold, *p* < 0.001) > A2780 (10-fold, *p* < 0.001) > MDA−MB−231 (5-fold, *p* < 0.01). Relative to cisplatin, **Pt^II^56ME*SS*** exhibited a cell line order of potency of HT29 (432-fold, *p* < 0.0001) > ADDP (147-fold, *p* < 0.0001) > A2780 (113-fold, *p* < 0.001) > MDA−MB−231 (93-fold, *p* < 0.01) > MCF−7 (59-fold, *p* < 0.001). As with all complexes, the order of cell line potency of **Pt^IV^56ME*SS*(OH)_2_** relative to cisplatin was highest in HT29 cells (62-fold, *p* < 0.0001) followed by ADDP cells (27-fold, *p* < 0.0001). The remaining cell lines showed order of potency of MCF−7 (24-fold, *p* < 0.001) > MDA−MB−231 (18-fold, *p* < 0.01) > A2780 (13-fold, *p* < 0.001).

The selective cytotoxicity index (SCI) (Table 2) was calculated for all complexes across the different cancer cell lines by dividing their IC_50_ (Table 1) in MCF10A by that of the cancer cell line. The SCI measures the window between cytotoxicity and anticancer activity. The greater the SCI, the greater the selectivity and therapeutic efficacy of the complex to cancer cells over non-cancer cells [65,66]. SCI values greater than one indicate some selectivity for killing cancer cells while numbers less than one indicate greater toxicity for the non-cancer cell line over the cancer cell line. Based on this measure, complexes were more toxic to cancer cell lines over the non-cancer cell line than cisplatin. It was expected that cisplatin would have the least SCI in the cisplatin-resistant cell lines HT29 and ADDP relative to the remaining cisplatin-sensitive cancer lines, with SCI of 0.06 and 0.04, respectively. Except for **Pt^II^56ME*SS*** on MCF−7 and ADDP cells and **Pt^II^5ME*SS*** on ADDP cells, all other complexes were more potent in killing cancer cells than the non-cancer cell line. The complexes showed a degree of preference towards some cell lines over others. In MDA−MB−231 cells, **Pt^II^PHEN*SS***, **Pt^IV^PHEN*SS*(OH)_2_**, **Pt^IV^5ME*SS*(OH)_2_** and **Pt^IV^56ME*SS*(OH)_2_** had a SCI of 3.45, 6.13, 4.38 and 6.92, respectively, while that of **Pt^II^5ME*SS*** and **Pt^II^56ME*SS*** was 2.27 and 2.93, respectively. In contrast, the SCI in HT29 for **Pt^II^PHEN*SS***, **Pt^IV^PHEN*SS*(OH)_2_**, **Pt^IV^5ME*SS*(OH)_2_** and **Pt^IV^56ME*SS*(OH)_2_** was less variable at 4.59, 4.57, 3.36 and 3.93, respectively, while that of **Pt^II^5ME*SS*** and **Pt^II^56ME*SS*** was 2.15 and 2.28, respectively. MCF−7 and A2780 showed greatest SCI with **Pt^IV^5ME*SS*(OH)_2_** complex of 5.66 and 5.71, correspondingly. Despite the greater SCI in some cancer cell lines compared to cisplatin, these complexes are not selective for all cell lines. The IC_50_ of **Pt^II^5ME*SS*** in ADDP (IC_50_ of 1.32 ± 1.33 µM) overlaps with that in MCF10A; IC_50_ of 1.18 ± 1.75 µM. This is also observed with **Pt^II^56ME*SS*** in MCF−7 and ADDP with IC_50_ of 0.56 ± 2.08 µM and 0.76 ± 1.27 µM, respectively, which overlaps with MCF10A; IC_50_ of 0.41 ± 1.52 µM.

### 3.2. Single-Cell 3D Diffusely Embedded Breast Cancer Model and Spheroid Model of Breast and Colorectal Cancer for Cytotoxicity Evaluation 

**Figure 2:**
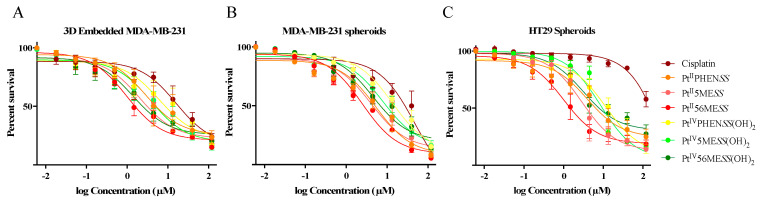
Effect of platinum(II) complexes on the survival of 3D Embedded (**A**). MDA−MB−231 networks, (**B**). MDA−MB−231 and (**C**). HT29 spheroids. Networks and spheroids were treated with 3-fold dilutions of the different agents (Cisplatin, **Pt^II^PHEN*SS***, **Pt^II^5ME*SS***, **Pt^II^56ME*SS***, **Pt^IV^PHEN*SS*(OH)_2_**, **Pt^IV^5ME*SS*(OH)_2_**, **Pt^IV^56ME*SS*(OH)_2_**) starting with a concentration of 150 µM and assayed for cell viability, as described in Section 2.4. Data points denote mean ± SEM from three independent experiments where samples were run in triplicate.

Three-dimensional cell spheroid models are more biologically relevant than two-dimensional cell culture models and provide a more comprehensive understanding of tumour cell biology, which should better predict in vivo drug response [52,53]. Spheroids from MDA−MB−231 and HT29 were bio-printed, grown and treated with platinum(II) and platinum(IV) dihydroxy complexes to determine the cytotoxic effect of the complexes in 3D models at 72 h post-treatment (Figure 2 and Figures S2–S4, and Table 3). The order of potency of each of the complexes in 3D models for both MDA−MB−231 and HT29 cell lines was largely unchanged relative to 2D growth. All complexes showed increased potency relative to cisplatin (measured by Student’s *t*-test). No significant differences were observed in IC_50_ among all complexes in both 2D and 3D growth conditions except in MDA−MB−231 spheroids treated with **Pt^IV^PHEN*SS*(OH)_2_** compared to **Pt^II^PHEN*SS*** (*p* < 0.05), **Pt^II^5ME*SS*** (*p* < 0.01), **Pt^II^56ME*SS*** (*p* < 0.01) and **Pt^IV^56ME*SS*(OH)_2_** (*p* < 0.05). Not unexpectedly, the IC_50_ for all complexes tended to increase in 3D culture relative to 2D culture with a non-significant average 1.5-fold increase in MDA−MB−231 3D network culture and a 4.0-fold and 5.3-fold increase in 3D spheroid culture for MDA−MB−231 cells and HT29 cells correspondingly. Within this trend to increase in 3D spheroid culture, two complexes showed significant increases in IC_50_ in both cell lines, viz., **Pt^IV^PHEN*SS*(OH)_2_** (MDA−MB−231 spheroids; *p* < 0.01 and HT29 spheroids; *p* < 0.05) and cisplatin (*p* < 0.0001). Interestingly, the ratio of the IC_50_ cisplatin/IC_50_ complex showed cell-type- and drug-class-specific effects. In HT29 cells, all three Pt(II) complexes showed little change in their potency relative to cisplatin between 2D- and 3D-spheroid cultures, while the Pt(IV) complexes showed a 2.0–2.5-fold greater potency than cisplatin in the 3D spheroid culture than in the 2D cell culture. In contrast, Pt(II) complexes showed a 30–75% reduction in potency relative to cisplatin in the MDA−MB−231 3D network and spheroid culture compared to the 2D culture, while the Pt(IV) complexes showed little change in potency relative to cisplatin in these cells.

### 3.3. Cellular Uptake Platinum(II) vs. Platinum(IV) Complexes 

**Figure 3:**
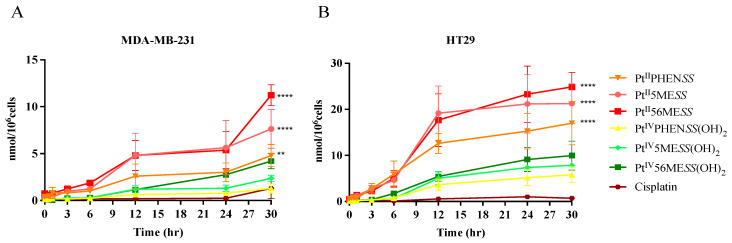
Cellular uptake of **Pt^II^PHEN*SS***, **Pt^II^5ME*SS***, **Pt^II^56ME*SS***, **Pt^IV^PHEN*SS*(OH)_2_**, **Pt^IV^5ME*SS*(OH)_2_** and **Pt^IV^56ME*SS*(OH)_2_**: ICP-MS analysis of the uptake of platinum in (**A**). MDA−MB−231 and (**B**). HT29 cells at 0, 0.5, 1, 3, 6, 12, 24 and 30 h as described in Section 2.5. *n* = 3 from three independent experiments where samples were run in triplicate. Data points denote mean ± SEM and expressed in nmol/10^6^cells. ** *p* < 0.01 and **** *p* < 0.0001 in comparison to cisplatin, as measured by two-way ANOVA.

The cellular uptake of Pt in cells treated with **Pt^II^PHEN*SS***, **Pt^II^5ME*SS***, **Pt^II^56ME*SS***, **Pt^IV^PHEN*SS*(OH)_2_**, **Pt^II^5ME*SS*(OH)_2_** and **Pt^IV^56ME*SS*(OH)_2_** relative to cisplatin, each at a fixed concentration of 3 μm, was measured by ICP-MS. As shown in Figure 3, cisplatin showed the least cellular uptake of any complex in both MDA−MB−231 and HT29 cell lines. The Pt(IV) complexes showed an intermediate uptake, while the Pt(II) complexes showed the highest level of uptake. Interestingly, HT29 cells showed greater uptake of all complexes relative to MDA−MB−231 cells. The time course of uptake was biphasic for all complexes in HT29 cells, with the rate of uptake highest between 0 h and 12 h, reducing from 12 to 30 h. Each complex also accumulated over time in MDA−MB−231 cells with more variable kinetics among complexes. The greater cellular uptake observed with the platinum(II) complexes (**Pt^II^PHEN*SS***, **Pt^II^5ME*SS*** and **Pt^II^56ME*SS*)** comparatively to their relative platinum(IV) complex (**Pt^IV^PHEN*SS*(OH)_2_**, **Pt^IV^5ME*SS*(OH)_2_** and **Pt^IV^56ME*SS*(OH)_2_**) may be attributable to the smaller square-planar geometry compared to that of the larger octahedral. Cells have an approximate volume of 1.7 pL [67], and **56ME*SS*** is equally distributed inside the cell; the drug’s concentration is about 5298 µM per MDA−MB−231 cell and 11,711 µM per HT29 cell, which is respectively around 1766 and 3904 times the starting concentration (3 µM). The ratio (intracellular/extracellular concentration) was calculated knowing that the extracellular concentration was 3 µM for each platinum complex (Figure S5). A significant accumulation of all platinum complexes was observed within both cell lines and at all time periods. For example, at 30 h, **Pt^II^56ME*SS*** recorded the greatest intracellular/extracellular in both MDA−MB−231 and HT29 (1765.95 ± 526.75 and 1509.01 ± 3903.51, respectively); a result that is correspondingly 8.7- and 34.8-fold higher than the maximum ratio reported for cisplatin (where a ratio of 202.79 ± 518.14 (MDA−MB−231) and 112.14 ± 83.99 (HT29) as reported after 30 h post-treatment) (Tables S3 and S4). These amplified cellular concentrations support an active transport mechanism of cell uptake. This effect is also observed in the case of cisplatin, which could enter cells via a combination of passive diffusion as well as active import via membrane transport proteins, specifically CTR1 [68]. The relative uptake of the complexes to cisplatin and to each other is evaluated by a two-way ANOVA with the first variable being time and the second variable being response of each complex. In MDA−MB−231 cells, the uptake of **Pt^II^PHEN*SS*** was significant relative to **Pt^IV^PHEN*SS*(OH)_2_** (*p* < 0.05) and to **Pt^II^56ME*SS*** (*p* < 0.01). **Pt^II^5ME*SS*** was significant relative to **Pt^IV^PHEN*SS*(OH)_2_** (*p* < 0.0001), **Pt^IV^5ME*SS*(OH)_2_** (*p* < 0.0001) and to **Pt^IV^56ME*SS*(OH)_2_** (*p* < 0.01). **Pt^II^56ME*SS*** was significant relative to **Pt^IV^PHEN*SS*(OH)_2_** (*p* < 0.0001), **Pt^IV^5ME*SS*(OH)_2_** (*p* < 0.0001) and to **Pt^IV^56ME*SS*(OH)_2_** (*p* < 0.0001). In HT29 cells, the uptake of **Pt^II^PHEN*SS*** was significant relative to **Pt^IV^PHEN*SS*(OH)_2_** (*p* < 0.001), **Pt^IV^5ME*SS*(OH)_2_** (*p* < 0.01) and to **Pt^IV^56ME*SS*(OH)_2_** (*p* < 0.05). In both MDA−MB−231 and HT29 cells, **Pt^II^5ME*SS*** was significant relative to **Pt^IV^PHEN*SS*(OH)_2_** (*p* < 0.0001), **Pt^IV^5ME*SS*(OH)_2_** (*p* < 0.0001) and to **Pt^IV^56ME*SS*(OH)_2_** (*p* < 0.01) and **Pt^II^56ME*SS*** was significant relative to **Pt^IV^PHEN*SS*(OH)_2_** (*p* < 0.0001), **Pt^IV^5ME*SS*(OH)_2_** (*p* < 0.0001) and to **Pt^IV^56ME*SS*(OH)_2_** (*p* < 0.0001). The uptake of **Pt^II^PHEN*SS***, **Pt^II^5ME*SS*** and **Pt^II^56ME*SS*** was significantly greater than cisplatin in both MDA−MB−231 and HT29 cells (Figure 3).

### 3.4. Mode of Uptake of Platinum(II) and Platinum(IV) Complexes 

**Figure 4:**
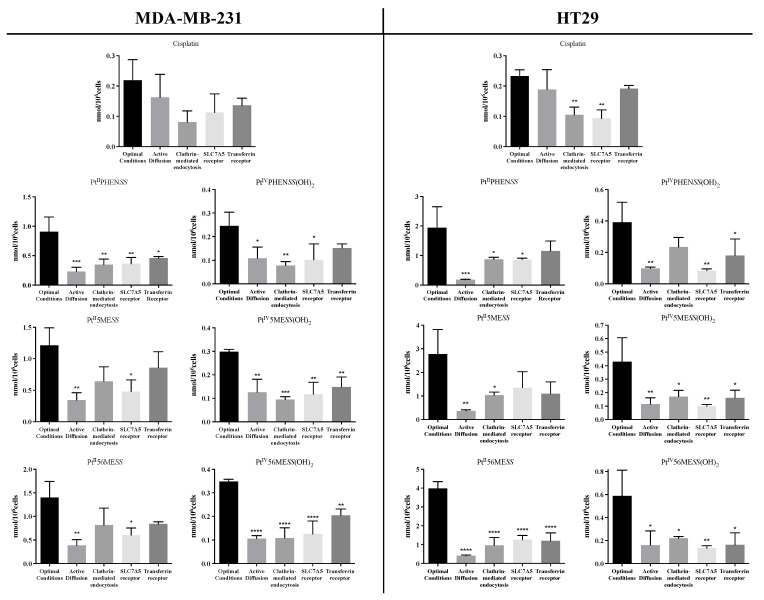
Mode of cellular uptake of platinum in MDA−MB−231 and HT29. The intracellular amount of platinum was measured by ICP-MS after incubation at 37 °C or 4 °C, as well as following inhibition of the SLC7A5, transferrin receptor or clathrin-mediated endocytosis as described in Section 2.6. Data denote mean ± SEM of three independent experiments where samples were run in triplicate. * *p* < 0.05, ** *p* < 0.01, *** *p* < 0.001 and **** *p* < 0.0001 in comparison to the optimal conditions, as measured by one-way ANOVA.

To determine if any essential transport proteins or mechanisms of uptake were involved in the absorption of **Pt^II^** and **Pt^IV^** dihydroxy complexes, the cellular uptake of **Pt^II^PHEN*SS***, **Pt^II^5ME*SS***, **Pt^II^56ME*SS***, **Pt^IV^PHEN*SS*(OH)_2_**, **Pt^IV^5ME*SS*(OH)_2_** and **Pt^IV^56ME*SS*(IV)(OH)_2_** was measured by ICP-MS after blocking the mechanism of interest (Figure 4, Figures S6 and S7) as described in Section 2.6. Reduced intracellular concentrations respectively after inhibition, as explained in Section 2.6, compared to optimal conditions indicated the complex used the route for cell uptake. All investigated platinum complexes exhibited a significant (measured by one-way ANOVA) reduction in cellular uptake after active transport inhibition, as depicted in Figure 4, in both MDA−MB−231 and HT29 cells, exhibiting the primary use of this mechanism for cellular uptake. SLC7A5 was the second most significantly used mechanism of cell uptake by all complexes except for no significance observed by **Pt^II^5ME*SS*** in HT29 cells. Clathrin-mediated endocytosis was another investigated mechanism used by all complexes in both cancers with significance as depicted in Figure 4. Another investigated mechanism was the cellular drug uptake by TfR, which is overexpressed in cancer cells [69,70]. Cellular uptake of all the platinum complexes was reduced after TfR inhibition, as shown in Figure 4. As shown in Figure 4, the cellular uptake of cisplatin was reduced at 4 °C compared to 37 °C (optimal condition) in both cell lines indicating the use of active transport. Clathrin-mediated endocytosis and SLC7A5 were also reduced in both cell lines with a significant reduction of *p* < 0.01 in HT29 cells, as well as TfR in both cell lines. The investigated platinum complexes relative to cisplatin and to each other were notably reduced with **Pt^II^56ME*SS,*** the least available after inhibition with the different mechanisms.

### 3.5. Cellular Localisation of Platinum(II) and Platinum(IV) Complexes

**Figure 5:**
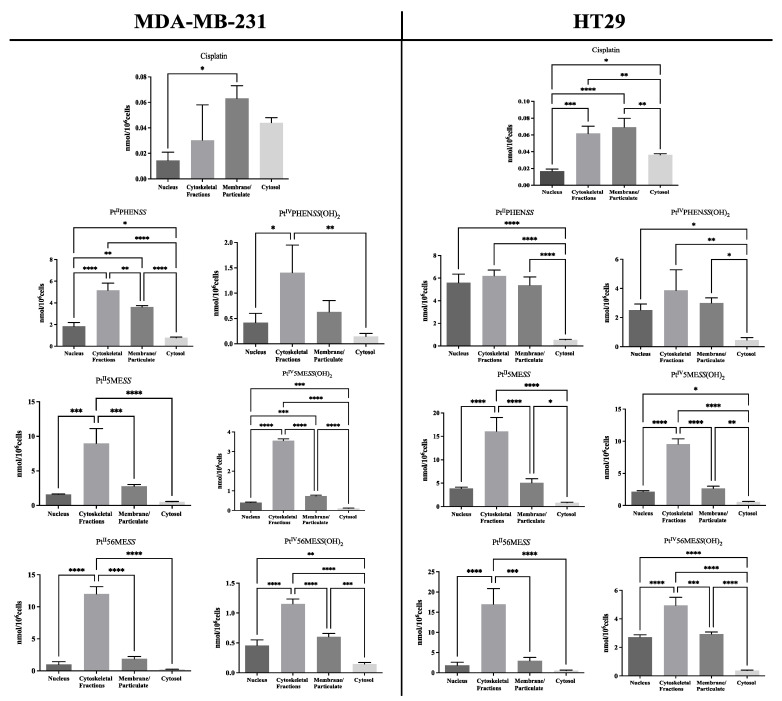
Cellular localisation of platinum in MDA−MB−231 and HT29 post-24 h. The intracellular amount of Pt was measured by ICP-MS after cellular fractionation, as described in Section 2.7. Data denote mean ± SEM of three independent experiments where samples were run in triplicate. * *p* < 0.05, ** *p* < 0.01, *** *p* < 0.001 and **** *p* < 0.0001 in comparison to the fractions, as measured by one-way ANOVA.

Cisplatin has been previously shown to be localised to the cytosol and particulates in A2780 and ADDP [71]. Similar platinum complexes have also been shown to accumulate mostly within the cytoskeleton, yet it cannot be concluded that the complexes’ mechanisms would be alike [26]. Accordingly, the cellular accumulation of platinum(II) and platinum(IV) dihydroxy complexes was studied in subcellular fractions of MDA−MB−231 and HT29 cells post-24 h of treatment and measured by ICP-MS, as shown in Figure 5, Figures S8 and S9. The bar graphs represent the amount of each platinum complex in the nuclear, cytoskeletal, membrane/particulate and cytosolic fraction. All complexes were largely accumulated in the cytoskeletal fraction, while that of cisplatin was mostly localised to the membrane/particulate fraction in both cancers. 

### 3.6. Cell Death Analysis 

**Figure 6:**
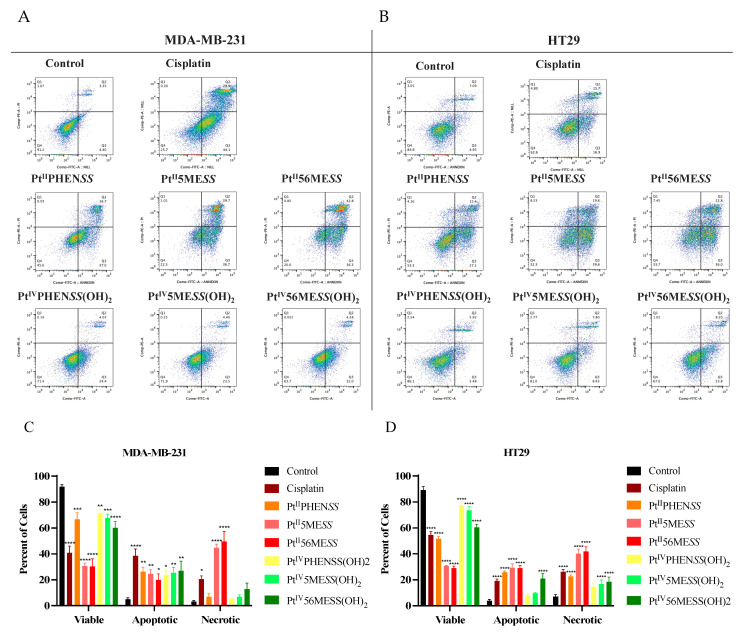
Flow cytometric analysis of cell death mediated by Cisplatin, **Pt^II^PHEN*SS***, **Pt^II^5ME*SS***, **Pt^II^56ME*SS***, **Pt^IV^PHEN*SS*(OH)_2_**, **Pt^IV^5ME*SS*(OH)_2_** and **Pt^IV^56ME*SS*(OH)_2_**. MDA−MB−231 and HT29 cells were treated with the complex and analysed at 72 h, as described in Section 2.8. Representative dot plots: (**A**). MDA−MB−231, (**B**). HT29 and Bar graphs (**C**). MDA−MB−231 and (**D**). HT29, representing percent viable, apoptotic and necrotic cells. Data points denote mean ± SEM. *n* = 3 from three independent experiments where samples were run in triplicate. * *p* < 0.05, ** *p* < 0.01, *** *p* < 0.001 and **** *p* < 0.0001 compared to control, as measured by one-way ANOVA.

Annexin V/PI staining was used to determine the type of cell death. MDA−MB−231 and HT29 cells were treated with **Pt^II^** and **Pt^IV^** dihydroxy complexes, and cisplatin was used as a positive control (Figure 6). Flow cytometry was performed at 72 h post-treatment. Cells treated with **Pt^II^PHEN*SS***, **Pt^II^5ME*SS***, **Pt^II^56ME*SS***, **Pt^IV^PHEN*SS*(OH)_2_**, **Pt^IV^5ME*SS*(OH)_2_** and **Pt^IV^56ME*SS*(OH)_2_** were shown to significantly undergo early apoptosis to late apoptosis/necrosis. All MDA−MB−231-treated cells induced significant (measured by one-way ANOVA) early apoptosis (Figure 6A,C). While, MDA−MB−231 cells treated with cisplatin (*p* < 0.05), **Pt^II^5MESS** (*p* < 0.0001) and **Pt^II^56MESS** (*p* < 0.0001) significantly increased late apoptosis/necrosis. HT29-treated cells showed a significant (measured by one-way ANOVA) increase (*p* < 0.0001) in **Pt^II^PHEN*SS***, **Pt^II^5ME*SS***, **Pt^II^56ME*SS***, **Pt^IV^56ME*SS*(OH)_2_** and cisplatin as well as in late apoptosis/necrosis (*p* < 0.0001), including **Pt^IV^5ME*SS*(OH)_2_** (Figure 6B,D). Comparatively, to cisplatin, **Pt^II^5ME*SS*** and **Pt^II^56ME*SS*** induced greater percent necrotic cell death with a significance of *p* < 0.01 and *p* < 0.001, respectively, in both cancers. 

### 3.7. Cell Cycle Arrest

**Figure 7:**
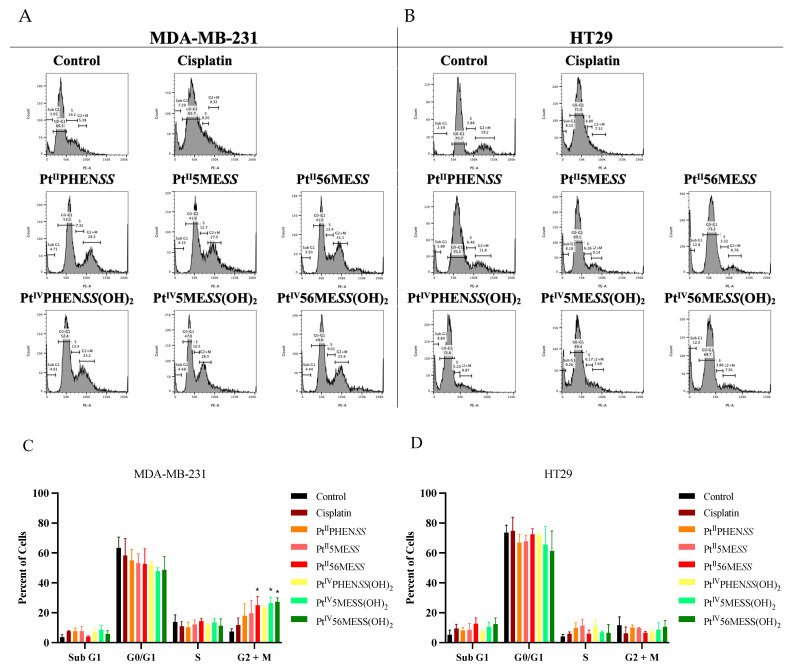
Flow cytometric analysis of cell cycle mediated by Cisplatin, **Pt^II^PHEN*SS***, **Pt^II^5ME*SS***, **Pt^II^56ME*SS***, **Pt^IV^PHEN*SS*(OH)_2_**, **Pt^IV^5ME*SS*(OH)_2_** and **Pt^IV^56ME*SS*(OH)_2_**. MDA−MB−231 and HT29 cells were treated with IC_30_ concentration of each complex and analysed at 72 h, as described in Section 2.9. Representative histogram plots: (**A**). MDA−MB−231, (**B**). HT29 and Bar graphs (**C**). MDA−MB−231 and (**D**). HT29, representing percent Sub G1, G0/G1, S and G2+M phases of cell cycle. Data points denote mean ± SEM. *n* = 3 from three independent experiments where samples were run in triplicate. * *p* < 0.05 compared to control, as measured by one-way ANOVA.

Subsequently, we examined, the cell cycle profiles of MDA−MB−231 and HT29 cells treated for 72 h with **Pt^II^PHEN*SS***, **Pt^II^5ME*SS***, **Pt^II^56ME*SS***, **Pt^IV^PHEN*SS*(OH)_2_**, **Pt^IV^5ME*SS*(OH)_2_**, **Pt^IV^56ME*SS*(OH)_2_** or cisplatin, at their concentrations corresponding to their IC_30_ values at 72 h (Figure 7). In MDA−MB−231-treated cells, a notable decrease in the G0/G1 phase and an increase in the G2+M phase of the cell cycle was observed across all complexes with significance (measured by one-way ANOVA) observed in **Pt^II^56ME*SS***, **Pt^IV^5ME*SS*(OH)_2_**, and **Pt^IV^56ME*SS*(OH)_2_** (*p* < 0.05) (Figure 7A,C). This has been previously reported by **Pt^II^56ME*SS*** in MDA−MB−231 cells [26]. While in HT29-treated cells, a decrease in the G0/G1 phase and an increase in the S phase of the cell cycle were observed across the platinum complexes (Figure 7B,D). Cisplatin remained greatest at the G0/G1 phase and G2+M phase compared to control cells in MDA−MB−231 and the S phase in HT29 cells. 

### 3.8. Reactive Oxygen Species (ROS) Detection Assay 

**Figure 8:**
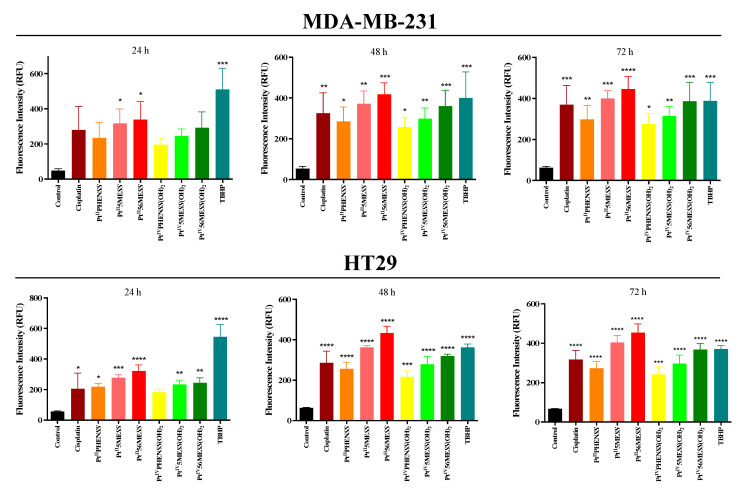
ROS production upon treatment with platinum(II) and (IV) complexes in MDA−MB−231 and HT29 and at 24, 48 and 72 h, as described in Section 2.10. **Pt^II^PHEN*SS***, **Pt^II^5ME*SS***, **Pt^II^56ME*SS***, **Pt^IV^PHEN*SS*(OH)_2_**, **Pt^IV^5ME*SS*(OH)_2_**, **Pt^IV^56ME*SS*(OH)_2_**, Cisplatin and TBHP: t-butyl hydroperoxide. Data points denote mean ± SEM. *n* = 3 from three independent experiments where samples were run in triplicate. * *p* < 0.05, ** *p* < 0.01, *** *p* < 0.001 and **** *p* < 0.0001 compared to control, as measured by one-way ANOVA.

MDA−MB−231 and HT29 cells were treated with **Pt^II^PHEN*SS***, **Pt^II^5ME*SS***, **Pt^II^56ME*SS***, **Pt^IV^PHEN*SS*(OH)_2_**, **Pt^IV^5ME*SS*(OH)_2_**, **Pt^IV^56ME*SS*(OH)_2_** or cisplatin. Upon ROS production, the DCFDA produced a fluorescent product (DCF) in the presence of cellular esterase. The measured fluorescence was therefore proportional to the produced ROS (Figure 8 and Figure S10, and Table S5). MDA−MB−231 and HT29 cells treated with platinum(II) and (IV) complexes exhibited significantly (measured by one-way ANOVA) increased ROS from 24, 48 to 72 h. TBHP (positive control) produced the most ROS at 24 h (*p* < 0.0001) and remained significant at 48 and 72 h with *p* < 0.0001. Relatively to cisplatin, **Pt^II^56ME*SS*** showed a significant increase in ROS in HT29 cells at 48 h (*p* < 0.001) and 72 h (*p* < 0.01). No significant differences were observed in ROS among all complexes in both cancers except in HT29 treated with **Pt^II^5ME*SS*** compared to **Pt^IV^PHEN*SS*(OH)_2_** (*p* < 0.001) and with **Pt^II^56ME*SS*** compared to **Pt^II^PHEN*SS*** (*p* < 0.0001), **Pt^IV^PHEN*SS*(OH)_2_** (*p* < 0.0001), **Pt^IV^5ME*SS*(OH)_2_** (*p* < 0.001) and **Pt^IV^56ME*SS*(OH)_2_** (*p* < 0.01) at 72 h.

### 3.9. Mitochondrial Membrane Potential

**Figure 9:**
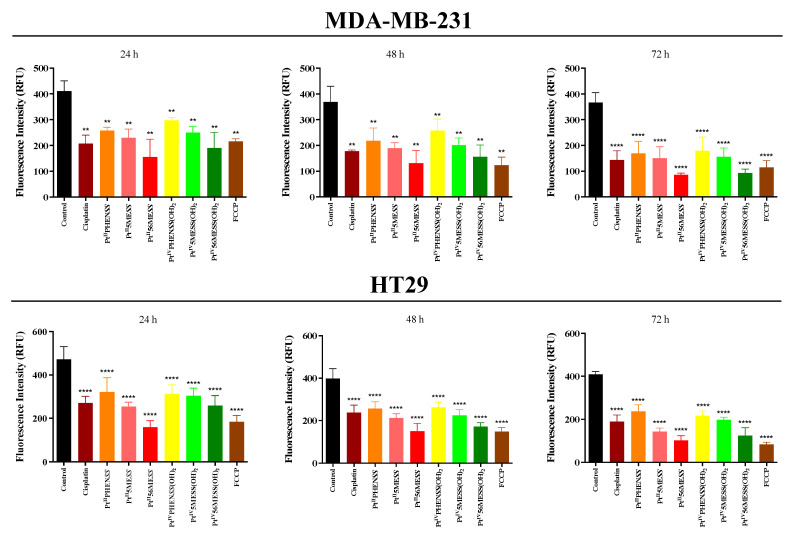
MtMP changes upon treatment with platinum(II) (**Pt^II^PHEN*SS***, **Pt^II^5ME*SS*** and **Pt^II^56ME*SS***) and platinum(IV) (**Pt^IV^PHEN*SS*(OH)_2_**, **Pt^IV^5ME*SS*(OH)_2_** and **Pt^IV^56ME*SS*(OH)_2_**) complexes, as well as cisplatin in MDA−MB−231 and HT29 cells at 24, 48 and 72 h, as described in Section 2.11. Data points denote mean ± SEM. *n* = 3 from three independent experiments where samples were run in triplicate. ** *p* < 0.01 and **** *p* < 0.0001 compared with control, as measured by one-way ANOVA.

The MtMP changes were measured 72 h post-treatment with **Pt^II^PHEN*SS***, **Pt^II^5ME*SS***, **Pt^II^56ME*SS***, **Pt^IV^PHEN*SS*(OH)_2_**, **Pt^IV^5ME*SS*(OH)_2_**, **Pt^IV^56ME*SS*(OH)_2_** or cisplatin. The relative fluorescence units measured are reported in Figure 9 and Table S6. The treatment was performed using each individual complex’s IC_50_ concentration at 72 h. A significant (measured by one-way ANOVA) hypopolarisation in the mitochondrial membrane was induced in MDA−MB−231 cancer cells for all the platinum complexes including FCCP (positive control) at 24 (*p* < 0.01), 48 (*p* < 0.01) and 72 h (*p* < 0.0001) compared to the untreated control group. HT29-treated cells also exhibited significant hypopolarisation across all complexes including FCCP at 24, 48 and 72 h (*p* < 0.0001). Relatively to cisplatin, **Pt^II^56ME*SS*** showed a significant decrease in MtMP in MDA−MB−231 cells at 72 h (*p* < 0.05) and in HT29 cells at 72 h (*p* < 0.0001). Relatively to cisplatin in HT29 cells, a significant decrease in MtMP was observed by **Pt^II^5ME*SS*** (*p* < 0.01), **Pt^II^PHEN*SS*** (*p* < 0.01) and **Pt^IV^56ME*SS*(OH)_2_** (*p* < 0.0001). In HT29 cells, the MtMP was significantly reduced in **Pt^II^56ME*SS*** compared to **Pt^II^5ME*SS*** (*p* < 0.05), **Pt^IV^PHEN*SS*(OH)_2_** (*p* < 0.0001) and **Pt^IV^5ME*SS*(OH)_2_** (*p* < 0.0001).

### 3.10. Morphological Changes in Microtubule Organisation Using Confocal Microscopy 

**Figure 10:**
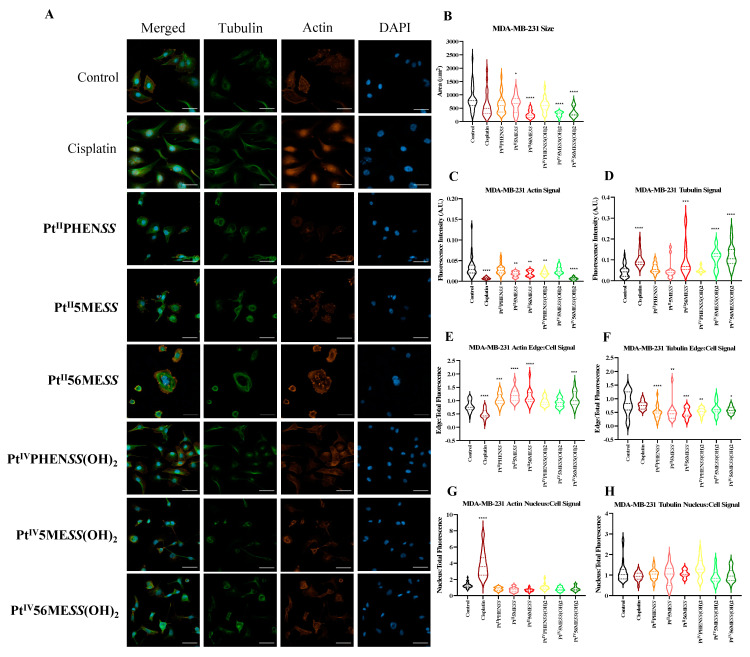

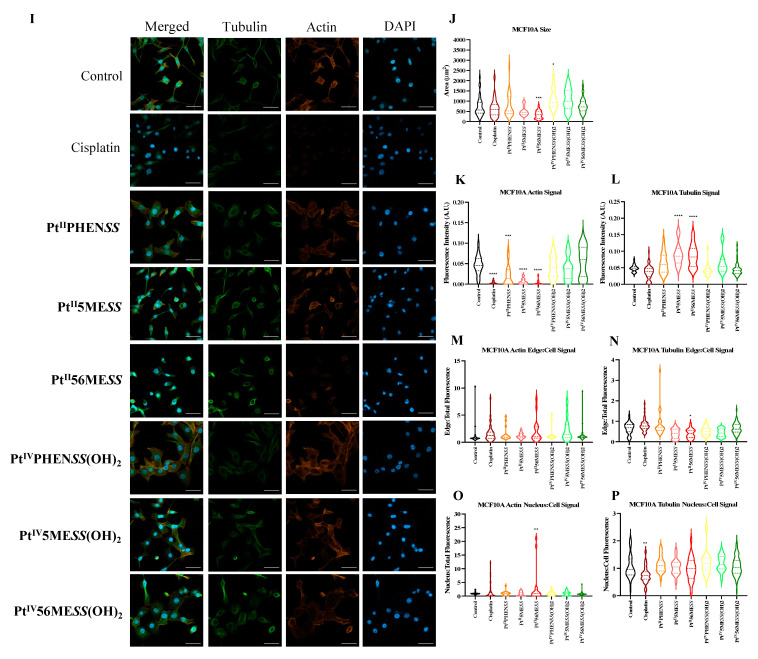

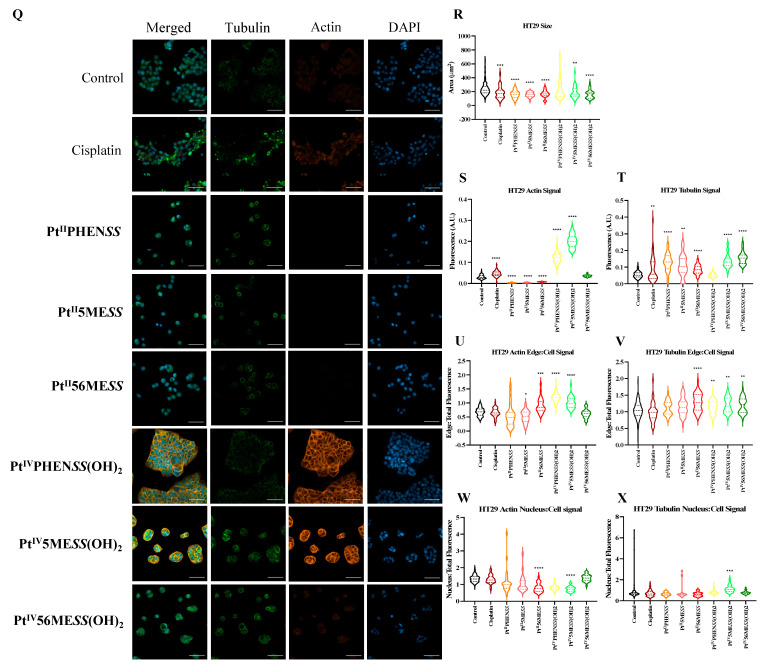
**Effect of platinum complexes and cisplatin on β−tubulin and F−actin.** Immunofluorescence upon treatment with Pt^II^ and Pt^IV^ complexes, as well as cisplatin in MDA−MB−231, MCF10A and HT29 cells at 72 h, as described in Section 2.12: (**A**). MDA−MB−231 airy scan images at 20×. (**B**). MDA−MB−231 cell size (µm^2^) (**C**). Actin expression in MDA−MB−231 (**D**). Tubulin expression in MDA−MB−231 (**E**). Edge/Cell ratio of actin expression in MDA−MB−231 (**F**). Edge/Cell ratio of tubulin expression in MDA−MB−231 (**G**). Nucleus/Cell ratio of actin expression in MDA−MB−231 (**H**). Nucleus/Cell ratio of tubulin expression in MDA−MB−231. (**I**). MCF10A airy scan images at 20×. (**J**). MCF10A cell size (µm^2^) (**K**). Actin expression in MCF10A. (**L**). Tubulin expression in MCF10A. (**M**). Edge/Cell ratio of actin expression in MCF10A. (**N**). Edge/Cell ratio of tubulin expression in MCF10A. (**O**). Nucleus/Cell ratio of actin expression in MCF10A. (**P**). Nucleus/Cell ratio of tubulin expression in MCF10A. (**Q**). HT29 airy scan images at 20×. (**R**). HT29 cell size (µm^2^) (**S**). Actin expression in HT29. (**T**). Tubulin expression in HT29. (**U**). Edge/Cell ratio of actin expression in HT29. (**V**). Edge/Cell ratio of tubulin expression in HT29. (**W**). Nucleus/Cell ratio of actin expression in HT29. (**X**). Nucleus/Cell ratio of tubulin expression in HT29. Data points denote mean ± SEM. *n* = 3 from three independent experiments. * *p* < 0.05, ** *p* < 0.01, *** *p* < 0.001 and **** *p* < 0.0001, as measured by one-way ANOVA.

Cisplatin modulates the cytoskeleton during the induction of apoptosis [72], making morphological changes in actin and tubulin critical indicators of efficacy. To determine whether the platinum(II) and (IV) complexes share this mechanism of action, MDA−MB−231, MCF10A and HT29 cells were incubated with either of the investigated agents, **Pt^II^PHEN*SS***, **Pt^II^5ME*SS***, **Pt^II^56ME*SS***, **Pt^IV^PHEN*SS*(OH)_2_**, **Pt^IV^5ME*SS*(OH)_2_**, **Pt^IV^56ME*SS*(OH)_2_** or cisplatin at IC_50_ concentration (Figure 10 and Figures S11–S13) for 72 h. Cells were fixed, permeabilised, blocked and labelled with phalloidin and β−tubulin antibody and DAPI, before confocal imaging. Morphological evaluations, encompassing cell size, alongside tubulin and phalloidin intensity metrics, showed the impact of these complexes on the cytoskeletal architecture. In MDA−MB−231-treated cells, total actin expression (Figure 10C) was significantly reduced across all treatments with a significant reduction in cisplatin, **Pt^II^5ME*SS***, **Pt^II^56ME*SS***, **Pt^IV^PHEN*SS*(OH)_2_**, and **Pt^IV^56ME*SS*(OH)_2_**. The actin signal on the edge of the cell (Figure 10E) was considerably increased in **Pt^II^PHEN*SS***, **Pt^II^5ME*SS***, **Pt^II^56ME*SS*** and **Pt^IV^56ME*SS*(OH)_2_** whilst reduced in cisplatin. Additionally, the actin nucleus-to-cell ratio was significantly (measured by one-way ANOVA) increased in cisplatin and reduced across all investigated agents compared to the control (Figure 10G), which further suggests a different mechanism of action of the investigated complexes than that of cisplatin. In MCF10A-treated cells, the total actin expression (Figure 10K) was reduced across platinum(II) complex treatments with a significant reduction (*p* < 0.0001) in cisplatin, **Pt^II^PHEN*SS***, **Pt^II^5ME*SS*** and **Pt^II^56ME*SS***, which may suggest reduced selectivity. However, no substantial changes in the actin signal on the edge of the cell (Figure 10M) or actin nucleus-to-cell ratio (Figure 10O) were observed, indicating no significant damage to the MCF10A cells at the IC_50_ treatment concentration required for MDA−MB−231. In HT29-treated cells, actin total cell expression (Figure 10S) was significantly reduced (*p* < 0.0001) in **Pt^II^PHEN*SS***, **Pt^II^5ME*SS*** and **Pt^II^56ME*SS***, while significantly increased in **Pt^IV^PHEN*SS*(OH)_2_**, **Pt^IV^5ME*SS*(OH)_2_** and cisplatin (*p* < 0.0001). The actin signal on the edge of the cell (Figure 10U) was considerably increased in **Pt^II^56ME*SS***, **Pt^IV^PHEN*SS*(OH)_2_**, and **Pt^IV^5ME*SS*(OH)_2_** and reduced in **Pt^II^PHEN*SS*** and **Pt^II^5ME*SS*** treatments, which identifies dysregulation and consequently rapid disassembly of the existing cytoskeleton that subsequently leads to apoptosis [73]. This was additionally not observed by cisplatin-treated cells, which proposes a different mechanism of action by the investigated complexes. The actin nucleus-to-cell ratio was significantly decreased in **Pt^II^56ME*SS*** and **Pt^IV^5ME*SS*(OH)_2_** (Figure 10W), suggesting the sense and mediation of apoptosis [74,75]. Tubulin is a key indicator of cellular division, shape, internal organisation and movement. Its dysregulation can induce mitotic arrest and subsequent cell death. The tubulin total cell expression in MDA−MB−231 cells (Figure 10D) was significantly increased in cisplatin, **Pt^II^56ME*SS***, **Pt^IV^5ME*SS*(OH)_2_** and **Pt^IV^56ME*SS*(OH)_2_**. This significant dysregulation in tubulin at 72 h can be linked to the G2+M arrest observed in Figure 7C. The tubulin signal on the edge of the cell (Figure 10F) was considerably reduced in **Pt^II^PHEN*SS***, **Pt^II^5ME*SS***, **Pt^II^56ME*SS***, **Pt^IV^PHEN*SS*(OH)_2_** and **Pt^IV^56ME*SS*(OH)_2_** with no considerable changes in the tubulin nucleus-to-cell ratio (Figure 10H), indicating its cytoplasmic function. The tubulin total cell expression in MCF10A cells (Figure 10L) was significantly increased in **Pt^II^5ME*SS*** and **Pt^II^56ME*SS***. The tubulin signal on the edge of the cell (Figure 10N) was considerably reduced in **Pt^II^56ME*SS***-treated cells with no considerable changes in the tubulin nucleus-to-cell ratio (Figure 10P). The tubulin total cell expression in HT29 cells (Figure 10T) was significantly increased in cisplatin, **Pt^II^PHEN*SS***, **Pt^II^5ME*SS***, **Pt^II^56ME*SS***, **Pt^IV^5ME*SS*(OH)_2_** and **Pt^IV^56ME*SS*(OH)_2_**. While the tubulin signal on the edge of the cell (Figure 10V) was increased in **Pt^II^56ME*SS***, **Pt^IV^PHEN*SS*(OH)_2_**, **Pt^IV^5ME*SS*(OH)_2_** and **Pt^IV^56ME*SS*(OH)_2_** with considerable changes in the tubulin nucleus-to-cell ratio in **Pt^IV^5ME*SS*(OH)_2_**-treated cells (Figure 10V). This may indicate the interaction of tubulin with nuclear proteins that function during DNA damage checkpoint and repair. In comparison to the control, MDA−MB−231-treated cells exhibited morphological changes in the cytoskeleton, with a characteristic aggregation of actin filaments observed after treatment with **Pt^II^56ME*SS***, as well as membrane blebbing observed after **Pt^II^PHEN*SS***, **Pt^II^5ME*SS*** and **Pt^II^56ME*SS*** treatment (Figure 10A and Figure S11). The cell size was reduced across all treatments and significantly reduced in **Pt^II^56ME*SS***, **Pt^IV^5ME*SS*(OH)_2_** and **Pt^IV^56ME*SS*(OH)_2_** (Figure 10B). Relatively to cisplatin, **Pt^II^56ME*SS*** (*p* < 0.05)- and **Pt^IV^5ME*SS*(OH)_2_**- (*p* < 0.05) treated cells’ sizes were significantly reduced. MCF10A cells treated with MDA−MB−231s IC_50_s did not exhibit the morphological changes (Figure 10I and Figure S12) observed in MDA−MB−231, such as membrane blebbing; however, there were fewer protuberances in treated cells compared to untreated cells (control), which are a normal growing phenomenon of healthy MCF10A cells. The cell area was significantly reduced in **Pt^II^56ME*SS***-treated MCF10A cells (Figure 10J). Relative to cisplatin, **Pt^II^56ME*SS***- (*p* < 0.05) treated cells’ sizes were significantly reduced. HT29-treated cells also showed morphological changes in the cytoskeleton, while untreated (control) cells exhibited the normal filamentous distribution of actin and tubulin. Phalloidin staining showed a characteristic aggregation of actin filaments re-localised to the nucleoli observed after treatment with **Pt^II^5ME*SS*** and **Pt^II^56ME*SS*** (Figure S14), suggesting the presence of DNA damage [76]. Membrane blebbing was also observed after treatment with all investigated agents (Figure 10Q and Figure S13). The HT29 cell size was also reduced across all treatments with a significant decrease in cisplatin, **Pt^II^PHEN*SS***, **Pt^II^5ME*SS***, **Pt^II^56ME*SS***, **Pt^IV^5ME*SS*(OH)_2_** and **Pt^IV^56ME*SS*(OH)_2_** (Figure 10R), all of which further support the presence of apoptotic cell death [77,78,79].

### 3.11. Wound Healing Assay

**Figure 11:**
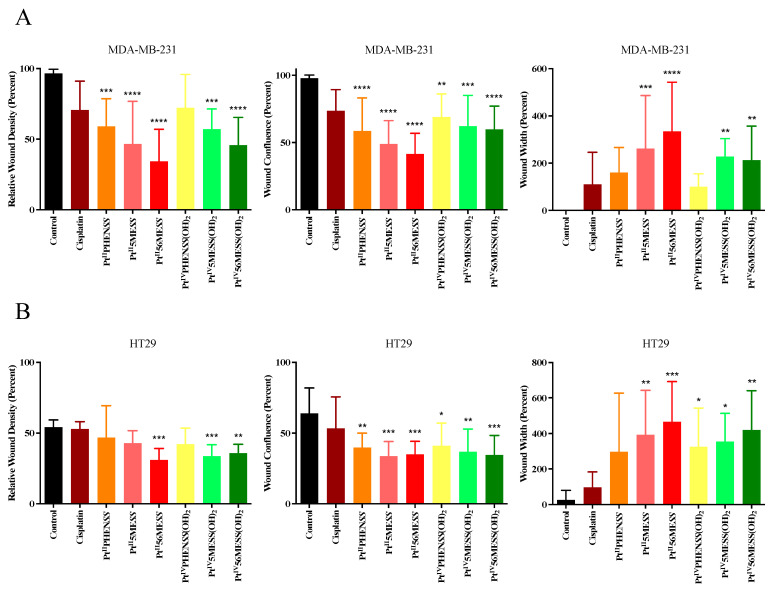
Cell migration (scratch wound healing assay). Percent relative wound density, wound confluence and wound width measured upon treatment with platinum(II) (**Pt^II^PHEN*SS***, **Pt^II^5ME*SS*** and **Pt^II^56ME*SS***) and platinum(IV) (**Pt^IV^PHEN*SS*(OH)_2_**, **Pt^IV^5ME*SS*(OH)_2_** and **Pt^IV^56ME*SS*(OH)_2_**) complexes, as well as cisplatin in (**A**). MDA−MB−231 and (**B**). HT29 cells up to 72 h, as described in Section 2.13. Data points denote mean ± SEM. *n* = 3 from three independent experiments where samples were run in triplicate. * *p* < 0.05, ** *p* < 0.01, *** *p* < 0.001 and **** *p* < 0.0001 compared with control, as measured by one-way ANOVA.

The initial stage of tumour metastasis involves the invasion of cancer cells into nearby tissue and blood vessels, necessitating the chemotactic migration of cancer cells. This is attentively related to their ability to adhere [80,81]. A 2D monolayer was wounded and monitored for 72 h post-treatment with **Pt^II^PHEN*SS***, **Pt^II^5ME*SS***, **Pt^II^56ME*SS***, **Pt^IV^PHEN*SS*(OH)_2_**, **Pt^IV^5ME*SS*(OH)_2_**, **Pt^IV^56ME*SS*(OH)_2_** or cisplatin. The percent relative wound density, wound confluence and wound width were measured (Figure 11 and Figure S15). MDA−MB−231 cells treated with the platinum(II) and platinum(IV) complexes exhibited significantly (measured by one-way ANOVA) reduced wound density and confluence (Figure S16), and a significant increase in wound width was observed in **Pt^II^5ME*SS***, **Pt^II^56ME*SS***, **Pt^IV^5ME*SS*(OH)_2_** and **Pt^IV^56ME*SS*(OH)_2_**. A significant (measured by one-way ANOVA) decrease in wound density was observed in HT29 cells (Figure S17) treated with the **Pt^II^56ME*SS*** (*p* < 0.001), **Pt^IV^5ME*SS*(OH)_2_** (*p* < 0.001) and **Pt^IV^56ME*SS*(OH)_2_** (*p* < 0.01). All platinum(II) and (IV) complexes exhibited significantly reduced wound confluence and a significant increase was observed in **Pt^II^5ME*SS*** (*p* < 0.01), **Pt^II^56ME*SS*** (*p* < 0.001), **Pt^IV^PHEN*SS*(OH)_2_** (*p* < 0.05), **Pt^IV^5ME*SS*(OH)_2_** (*p* < 0.05) and **Pt^IV^56ME*SS*(OH)_2_** (*p* < 0.01). Compared to cisplatin, **Pt^II^56ME*SS*** wound width was significantly increased (*p*< 0.05) in MDA−MB−231 cells as well as in HT29 cells treated with **Pt^II^5ME*SS*** (*p* < 0.05), **Pt^II^56ME*SS*** (*p* < 0.01) and **Pt^IV^56ME*SS*(OH)_2_** (*p* < 0.05) wound width.

### 3.12. Western Blot 

**Figure 12:**
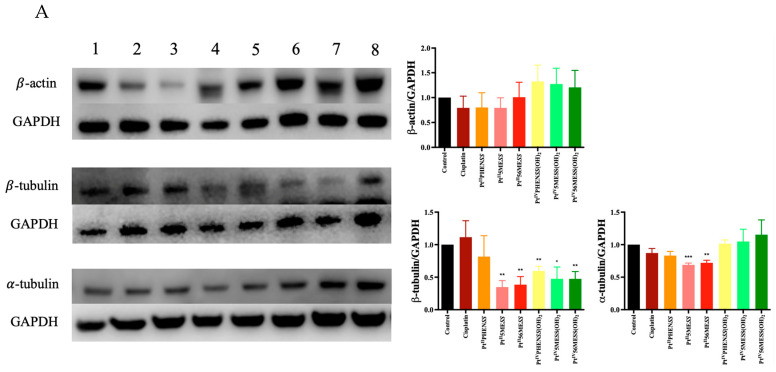

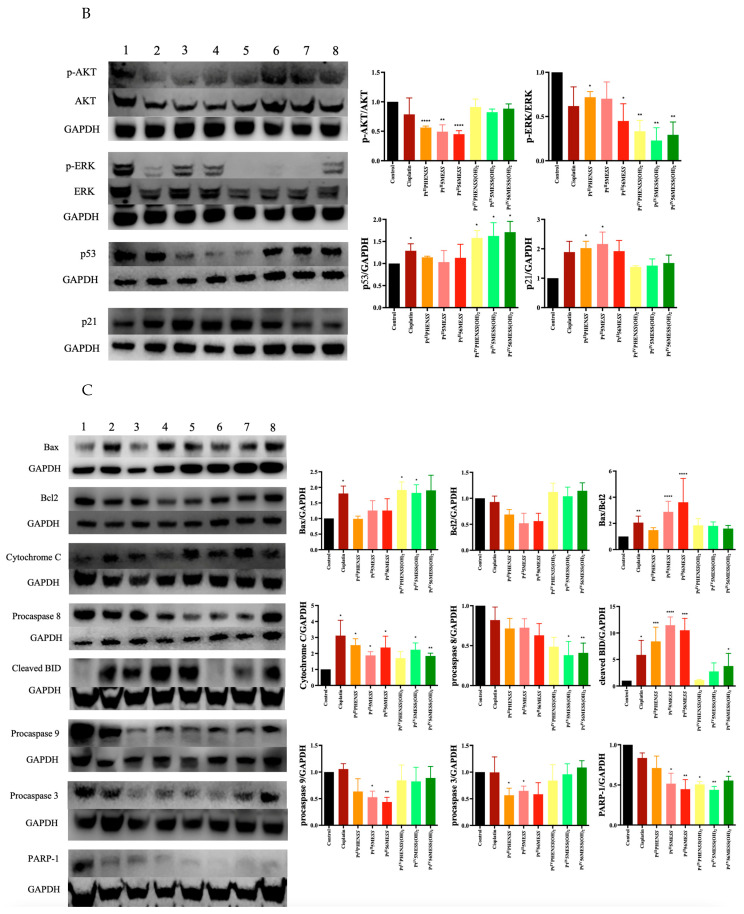

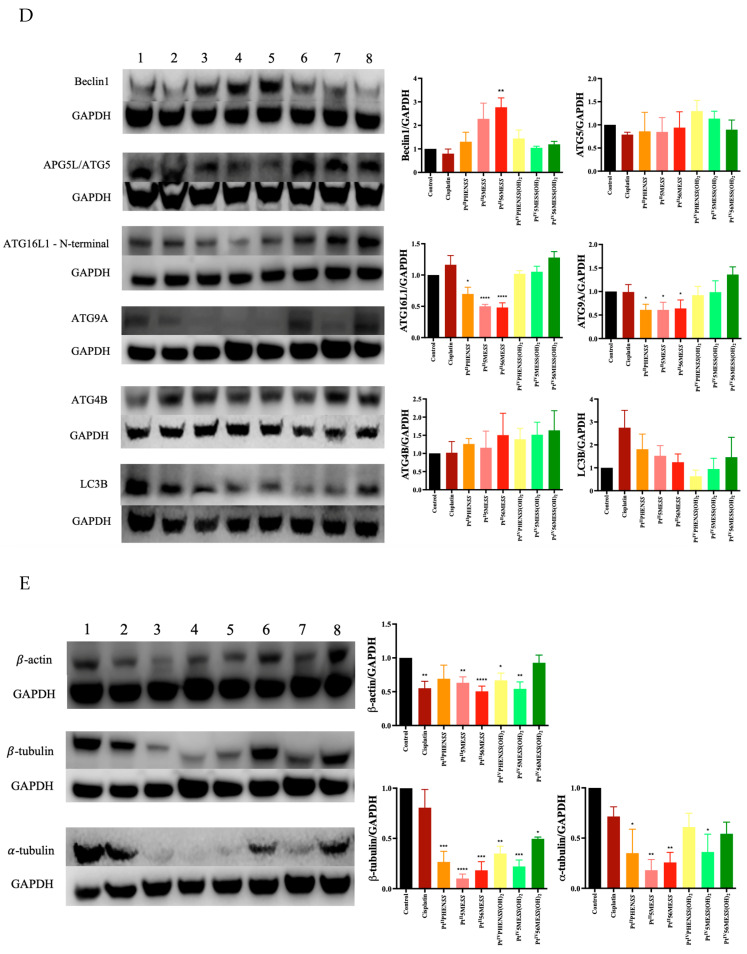

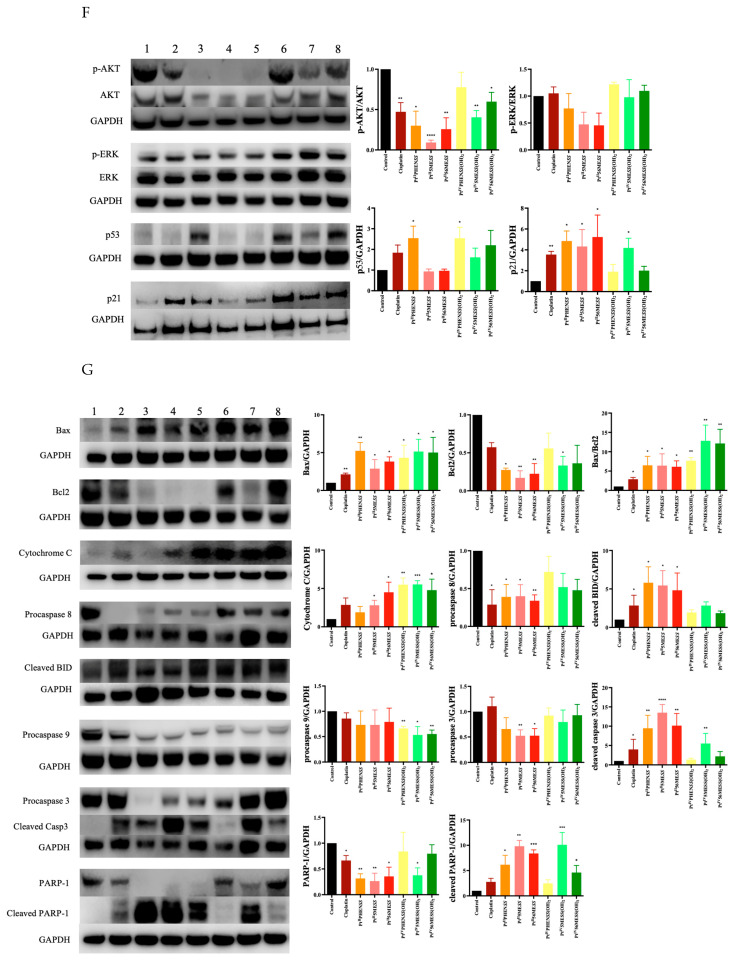

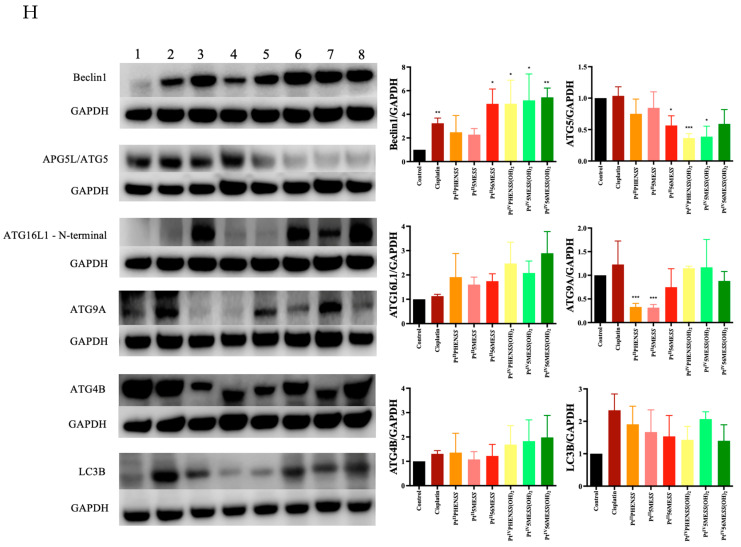
Protein expression upon treatment with platinum(II) (**Pt^II^PHEN*SS*** (lane 3), **Pt^II^5ME*SS*** (lane 4) and **Pt^II^56ME*SS*** (lane 5)) and platinum(IV) (**Pt^II^PHEN*SS*(OH)_2_** (lane 6), **Pt^IV^5ME*SS*(OH)_2_** (lane 7) and **Pt^IV^56ME*SS*(OH)_2_** (lane 8)) complexes, as well as cisplatin (lane 2) in MDA−MB−231 and HT29 cells at 72 h, as described in Section 2.14: (**A**). MDA−MB−231 microtubule cytoskeleton markers (**B**). MDA−MB−231 cell proliferation markers (**C**). MDA−MB−231 intrinsic and extrinsic apoptotic cell death markers (**D**). MDA−MB−231 autophagy markers (**E**). HT29 microtubule cytoskeleton markers (**F**). HT29 cell proliferation markers (**G**). HT29 intrinsic and extrinsic apoptotic cell death markers (**H**). HT29 autophagy markers. Data points denote mean ± SEM. *n* = 3 from three independent experiments. * *p* < 0.05, ** *p* < 0.01, *** *p* < 0.001 and **** *p* < 0.0001 compared with control (lane 1), as measured by unpaired Student’s *t*-test. The full uncropped Western blot, with its corresponding molecular markers, is represented in Figures S24–S31.

The effect of **Pt^II^PHEN*SS***, **Pt^II^5ME*SS***, **Pt^II^56ME*SS***, **Pt^IV^PHEN*SS*(OH)_2_**, **Pt^IV^5ME*SS*(OH)_2_** and **Pt^IV^56ME*SS*(OH)_2_** on the expression of microtubule cytoskeleton, cell proliferation, key apoptotic and autophagic proteins in MDA−MB−231 and HT29 cells was measured (Figure 12). Results have shown some changes to the microtubule cytoskeleton markers in MDA−MB−231-treated cells with a notably reduced expression in β-tubulin in response to **Pt^II^5ME*SS***, **Pt^II^56ME*SS***, **Pt^IV^PHEN*SS*(OH)_2_**, **Pt^IV^5ME*SS*(OH)_2_** and **Pt^IV^56ME*SS*(OH)_2_** treatments as well as α-tubulin with **Pt^II^5ME*SS*** and **Pt^II^56ME*SS*** treatments (Figure 12A). Cellular proliferation markers (Figure 12B) were also affected in MDA−MB−231 cells with a significant (measured by Student’s *t*-test) reduction in Protein Kinase B (AKT) activity, particularly the p-AKT/AKT in **Pt^II^PHEN*SS***, **Pt^II^5ME*SS*** and **Pt^II^56ME*SS*** treatments. The extracellular signal-regulated kinase (ERK) exhibited significant inhibition, particularly in p-ERK/ERK of all investigated agents, with that of the platinum(IV) dihydroxy derivatives reduced more notably than the platinum(II) complexes, including cisplatin. Tumour protein p53 (p53) and cyclin-dependent kinase inhibitor 1 (p21) were also studied and exhibited an increase in expression across all agents compared to the control. The platinum(IV) complexes were increased at p53 significantly, while the platinum(II) complexes were increased significantly at p21. The mitochondrial intrinsic and extrinsic mechanism of cell death was further studied in MDA−MB−231-treated cells (Figure 12C). The levels of the pro-apoptotic protein Bcl-2-associated X-protein (Bax) increased while the expression of the anti-apoptotic factor B-cell CLL/lymphoma 2 (Bcl2) decreased across treatments which exhibited a significant (measured by Student’s *t*-test) increase in Bax/Bcl2 in **Pt^II^5ME*SS*** (*p* < 0.0001), **Pt^II^56ME*SS*** (*p* < 0.0001) and cisplatin (*p* < 0.01) at 72 h, indicating a pro-apoptotic mechanism. Cytochrome c levels were elevated across all agents with a 2–3-fold increase with significance compared to the control. Procaspase 8 was reduced across all treatments with noteworthy significance at **Pt^IV^5ME*SS*(OH)_2_** (*p* < 0.05) and **Pt^IV^56ME*SS*(OH)_2_** (*p* < 0.01) treatments, while cleaved BID was significantly increased across all treatments with **Pt^II^5ME*SS*** (*p* < 0.0001) > **Pt^II^56ME*SS*** (*p* < 0.001) > **Pt^II^PHEN*SS*** (*p* < 0.001) > cisplatin (*p* < 0.05) > **Pt^IV^56ME*SS*(OH)_2_** (*p* < 0.05) indicating the activation of the extrinsic pathway to cell death. The executionary caspases 9 and 3 were also active indicated by a significant reduction in expression of the platinum(II) complexes. The levels of Poly [ADP-ribose] polymerase 1 (PARP-1) were significantly reduced in all platinum(IV) complexes and **Pt^II^5ME*SS*** (*p* < 0.05) and **Pt^II^56ME*SS*** (*p* < 0.01). Autophagy markers were also investigated in MDA−MB−231-treated cells (Figure 12D). Beclin-1 was increased by the platinum(II) complexes with significance (measured by Student’s *t*-test) in **Pt^II^56ME*SS*** (*p* < 0.01). Autophagy-related 5 (ATG5), autophagy-related 16-like 1 (ATG16L1), autophagy-related 9A (ATG9A) and autophagy-related protease 4B (ATG4B) expression levels were increased in **Pt^IV^PHEN*SS*(OH)_2_**, **Pt^IV^5ME*SS*(OH)_2_** and **Pt^IV^56ME*SS*(OH)_2_**. While the levels of ATG16L1 were significantly reduced in all platinum complexes with **Pt^II^56ME*SS*** (*p* < 0.0001) > **Pt^II^5ME*SS*** (*p* < 0.001) > **Pt^II^PHEN*SS*** (*p* < 0.001). ATG9A expression levels were significantly reduced (*p* < 0.05) across **Pt^II^PHEN*SS***, **Pt^II^5ME*SS*** and **Pt^II^56ME*SS***. Microtubule-associated proteins 1A/1B light chain 3B (LC3B) protein expression levels were increased in cisplatin (up to 3 folds). The same mechanisms and markers were investigated in HT29 to better understand the drugs response. Markers of the microtubule cytoskeleton were studied in HT29-treated cells (Figure 10E) with a similar response observed by MDA−MB−231. β−actin was similarly reduced in expression across all treatments. β-tubulin expression was reduced across all treatments with a significant reduction in **Pt^II^5ME*SS*** (*p* < 0.0001) > **Pt^II^56ME*SS*** (*p* < 0.001) > **Pt^IV^5ME*SS*(OH)_2_** (*p* < 0.001) > **Pt^II^PHEN*SS*** (*p* < 0.001) > **Pt^IV^PHEN*SS*(OH)_2_** (*p* < 0.01) > **Pt^IV^56ME*SS*(OH)_2_** (*p* < 0.05). α-tubulin expression was reduced significantly in **Pt^II^PHEN*SS*** (*p* < 0.05), **Pt^II^5ME*SS*** (*p* < 0.01), **Pt^II^56ME*SS*** (*p* < 0.01) and **Pt^IV^5ME*SS*(OH)_2_** (*p* < 0.05). Cellular proliferation markers (Figure 12F) were also affected in HT29 cells with a significant reduction in p-AKT/AKT, similarly to that observed by MDA−MB−231 in **Pt^II^PHEN*SS*** (*p* < 0.05), **Pt^II^5ME*SS*** (*p* < 0.0001) and **Pt^II^56ME*SS*** (*p* < 0.01), **Pt^IV^5ME*SS*(OH)_2_** (*p* < 0.01), **Pt^IV^56ME*SS*(OH)_2_** (*p* < 0.05) treatments and cisplatin (*p* < 0.01). The p-ERK/ERK exhibited a reduction in **Pt^II^5ME*SS***- and **Pt^II^56ME*SS***-treated cells without significance. p53 showed an increase in expression levels with **Pt^II^PHEN*SS*** (*p* < 0.05) and **Pt^IV^PHEN*SS*(OH)_2_** (*p* < 0.05). p21 expression was increased across all investigated agents with a prominent increase in **Pt^II^PHEN*SS*** (*p* < 0.05), **Pt^II^5ME*SS*** (*p* < 0.05), **Pt^II^56ME*SS*** (*p*< 0.05), **Pt^IV^5ME*SS*(OH)_2_** (*p* < 0.05) and cisplatin (*p* < 0.01). The mitochondrial intrinsic and extrinsic mechanism of cell death was also studied in HT29-treated cells (Figure 12G). The protein expression levels of pro-apoptotic protein Bax increased significantly in all treatments. While that of the anti-apoptotic factor Bcl2 decreased significantly in **Pt^II^PHEN*SS*** (*p* < 0.05), **Pt^II^5ME*SS*** (*p* < 0.01), **Pt^II^56ME*SS*** (*p* < 0.01) and **Pt^IV^5ME*SS*(OH)_2_** (*p* < 0.05). This exhibited a significant increase in the ratio of Bax/Bcl2 in all treatments indicating a pro-apoptotic mechanism. Cytochrome c levels were also elevated across all agents. Procaspase 8 was reduced significantly across all platinum(II) treatments, while cleaved BID was increased suggesting the activation of the extrinsic pathway to cell death of the platinum(II) treatments while the platinum(IV) complexes only showed a slight increase. The executionary caspases were also active indicated by a reduction in expression across all treatments in procaspase 9 and 3. Procaspase 9 exhibited a significant reduction by **Pt^IV^PHEN*SS*(OH)_2_** (*p* < 0.01), **Pt^IV^5ME*SS*(OH)_2_** (*p* < 0.05) and **Pt^IV^56ME*SS*(OH)_2_** (*p* < 0.01). Procaspase 3 exhibited significant reduction by **Pt^II^5ME*SS*** (*p* < 0.01) and **Pt^II^5ME*SS*** (*p* < 0.05) suggesting the activity and cleavage of caspase 3. An increase in cleaved caspase 3 was identified in HT29 cells across all investigated agents with significant increase in cisplatin (*p* < 0.05), **Pt^II^PHEN*SS*** (*p* < 0.01), **Pt^II^5ME*SS*** (*p* < 0.0001), **Pt^II^56ME*SS*** (*p* < 0.01) and **Pt^IV^5ME*SS*(OH)_2_** (*p* < 0.01). This suggests the active cleavage of PARP-1, which was reduced in all treatments, with an increase across all investigated agents in cleaved PARP-1 including **Pt^II^PHEN*SS*** (*p* < 0.05), **Pt^II^5ME*SS*** (*p* < 0.01), **Pt^II^56ME*SS*** (*p* < 0.001), **Pt^IV^5ME*SS*(OH)_2_** (*p* < 0.001) and **Pt^IV^56ME*SS*(OH)_2_** (*p* < 0.05). Autophagy markers were also investigated in HT29-treated cells (Figure 12H). Beclin-1 was significantly increased (*p* < 0.05) in **Pt^II^56ME*SS***, **Pt^IV^PHEN*SS*(OH)_2_**, **Pt^IV^5ME*SS*(OH)_2_** and **Pt^IV^56ME*SS*(OH)_2_** treatments. ATG5 levels were reduced with significance in **Pt^II^56ME*SS***, **Pt^IV^PHEN*SS*(OH)_2_**, **Pt^IV^5ME*SS*(OH)_2_** and **Pt^IV^56ME*SS*(OH)_2_**. ATG16L1 and ATG4B levels were increased across all treatments without notable significance. ATG9A expression levels were notably decreased in **Pt^II^PHEN*SS*** and **Pt^II^5ME*SS*** (*p* < 0.001). The protein expression levels of LC3B were increased across all investigated agents.

### 3.13. Proteomics 

**Figure 13:**
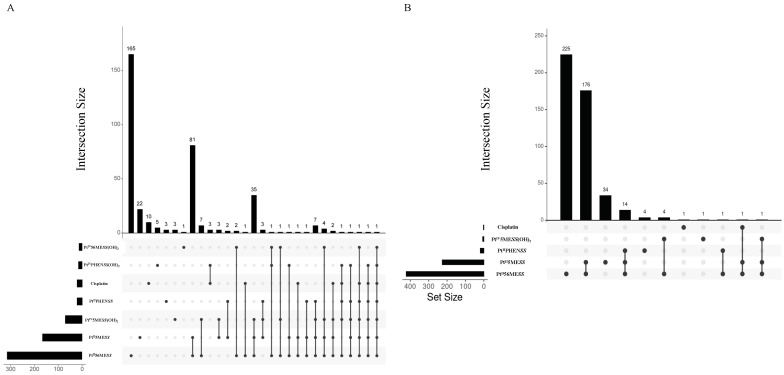
Differential proteins upon treatment with platinum(II) (**Pt^II^PHEN*SS***, **Pt^II^5ME*SS*** and **Pt^II^56ME*SS***) and platinum(IV) (**Pt^IV^PHEN*SS*(OH)_2_**, **Pt^IV^5ME*SS*(OH)_2_** and **Pt^IV^56ME*SS*(OH)_2_**) complexes, as well as cisplatin in (**A**). MDA−MB−231 and (**B**). HT29 cells at 72 h. UpSet plots summarise the differential protein expression analysis for the prodrugs and ligands. The differential protein expression analysis for the prodrugs and ligands is summarised in UpSet plots. Each figure’s bottom-left horizontal bar graph displays the total number of proteins with variations in log 2-fold change in expression for each complex. The same differentially expressed proteins that are shared by the complexes compared on the left are shown by the black circles connected to the right of these bar graphs. The vertical bar graph at the top quantifies the number of proteins with similar log 2-fold change expression differences in the drug comparisons.

**Figure 14:**
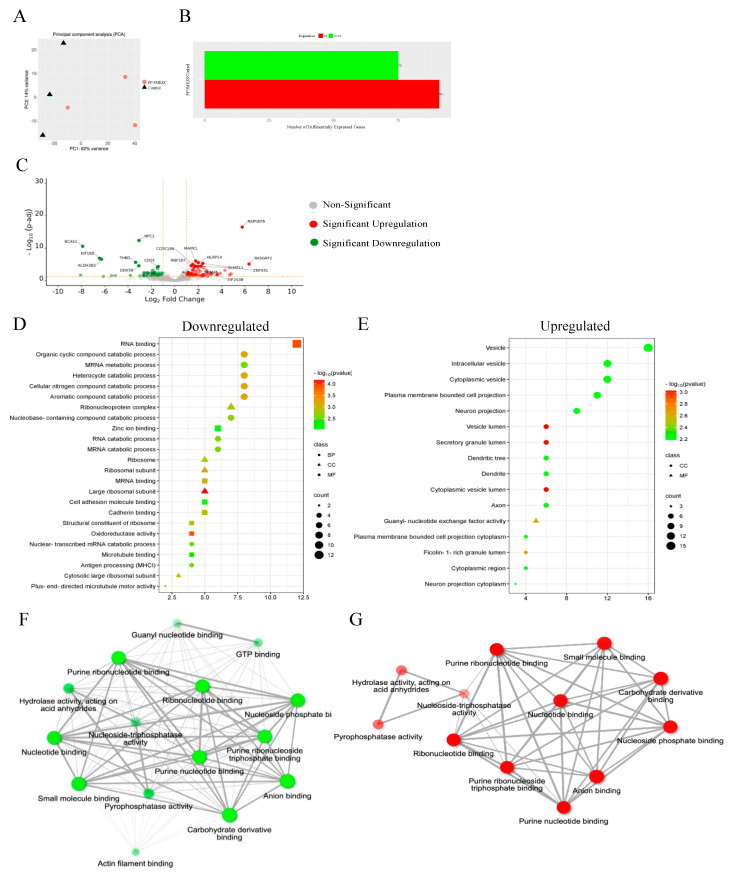

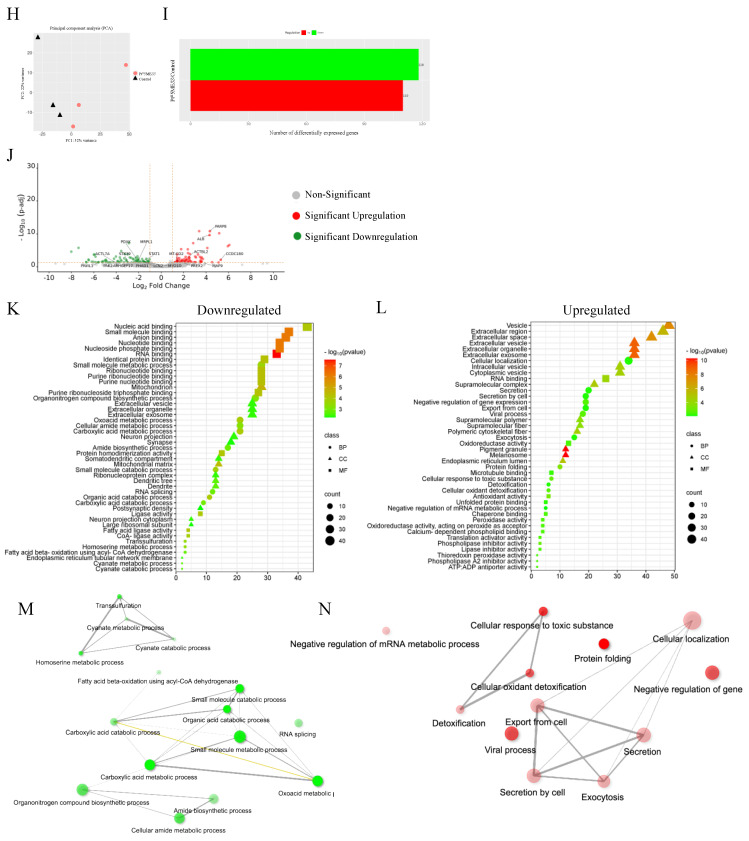
Proteomic analysis of MDA−MB−231 and HT29 upon treatment with **Pt^II^5ME*SS*** as described in Section 2.15: (**A**). MDA−MB−231 principal component analysis. (**B**). MDA−MB−231 number of differentially expressed proteins (DEPs). (**C**). MDA−MB−231 volcano plot of DEPs upregulated (red) and downregulated (green). GO enriched biological processes, cellular components, and molecular function in MDA−MB−231, (**D**). Downregulated and (**E**). Upregulated proteins. MDA−MB−231 pathway enrichment and gene act network analysis with the most significance of (**F**). Downregulated pathways; mRNA metabolic process, GTP binding and nucleoside activity and (**G**). Upregulated pathways; nucleotide binding and pyrophosphatase activity. (**H**). HT29 principal component analysis. (**I**). HT29 number of differentially expressed proteins (DEPs). (**J**). HT29 volcano plot of DEPs upregulated (red) and downregulated (green). GO enriched biological processes, cellular components, and molecular function in HT29, (**K**). Downregulated and (**L**). Upregulated proteins. HT29 pathway enrichment and gene act network analysis with the most significance of (**M**). Downregulated pathways; molecular metabolic and catabolic processes and RNA splicing and (**N**). Upregulated pathways; protein folding, response to toxic substance, detoxification, secretion, and exocytosis activity. Data points denote mean ± SEM. *n* = 3 from three independent experiments. For clarity, a larger representation is provided in the Supplementary Figure S22.

**Figure 15:**
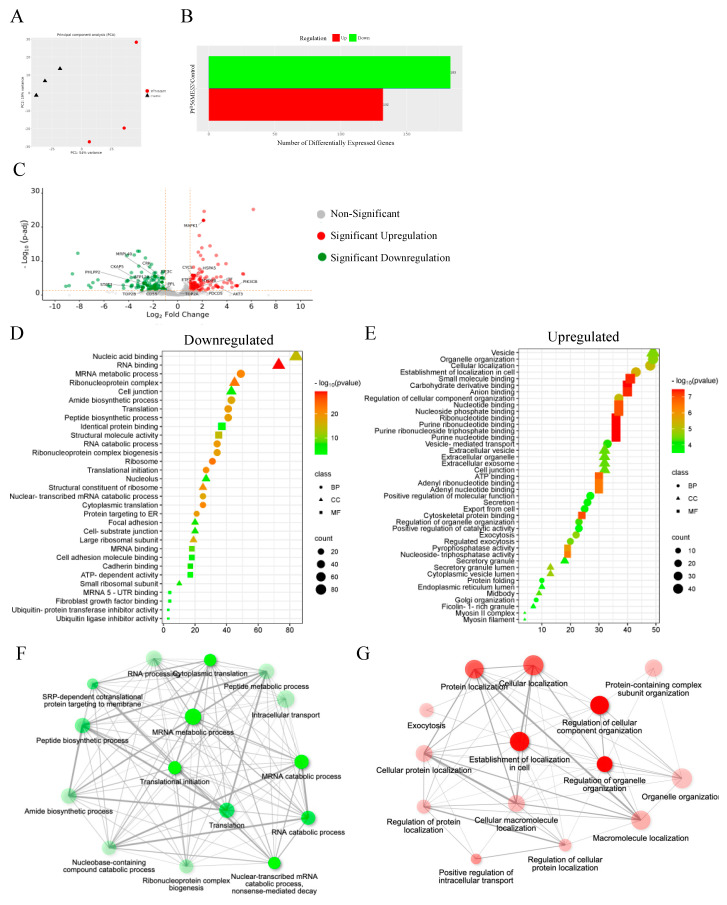

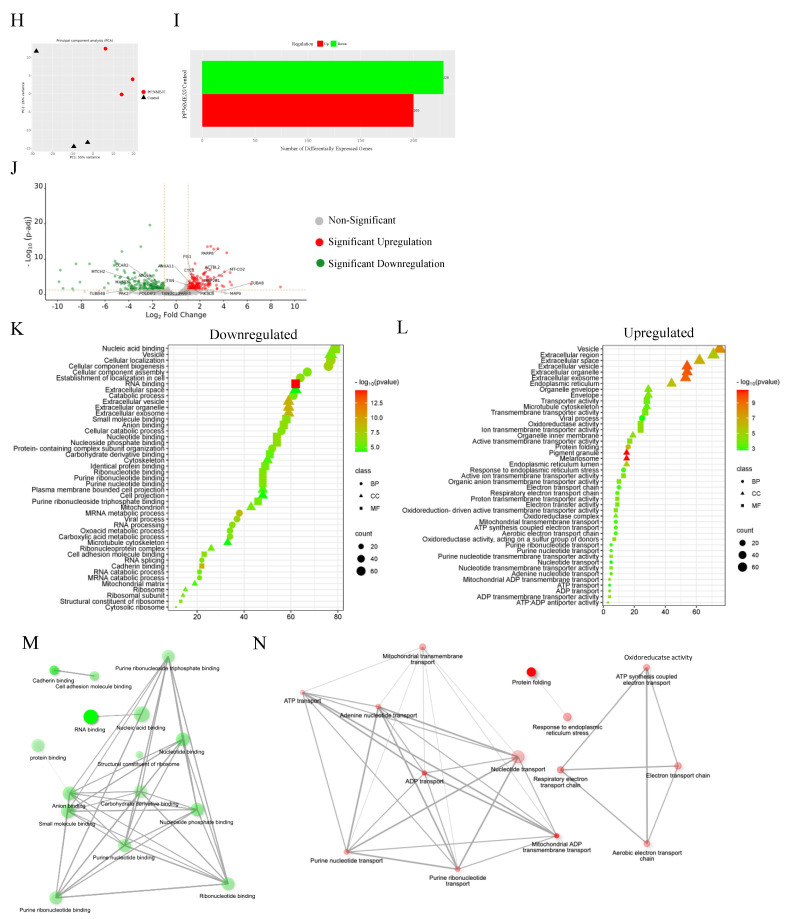
Proteomic analysis of MDA−MB−231 and HT29 upon treatment with **Pt^II^56ME*SS*** as described in Section 2.15: (**A**). MDA−MB−231 principal component analysis. (**B**). MDA−MB−231 number of differentially expressed proteins (DEPs). (**C**). MDA−MB−231 volcano plot of DEPs upregulated (red) and downregulated (green). GO enriched biological processes, cellular components and molecular function in MDA−MB−231 (**D**). Downregulated and (**E**). Upregulated proteins. MDA−MB−231 pathway enrichment and gene act network analysis with the most significance of (**F**). Downregulated pathways; translational initiation and mRNA metabolic process and (**G**). Upregulated pathways; localisation in cell, cellular organisation, protein localisation and exocytosis. (**H**). HT29 principal component analysis. (**I**). HT29 number of differentially expressed proteins (DEPs). (**J**). HT29 volcano plot of DEPs upregulated (red) and downregulated (green). GO enriched biological processes, cellular components and molecular function in HT29, (**K**). Downregulated and (**L**). Upregulated proteins. HT29 pathway enrichment and gene act network analysis with the most significance of (**M**). Downregulated pathways; RNA binding and nucleotide binding and (**N**). Upregulated pathways; ion transmembrane transport, active transport, and oxidoreductase activity. Data points denote mean ± SEM. *n* = 3 from three independent experiments. For clarity, a larger representation is provided in the Supplementary Figure S23.

**Figure 16:**
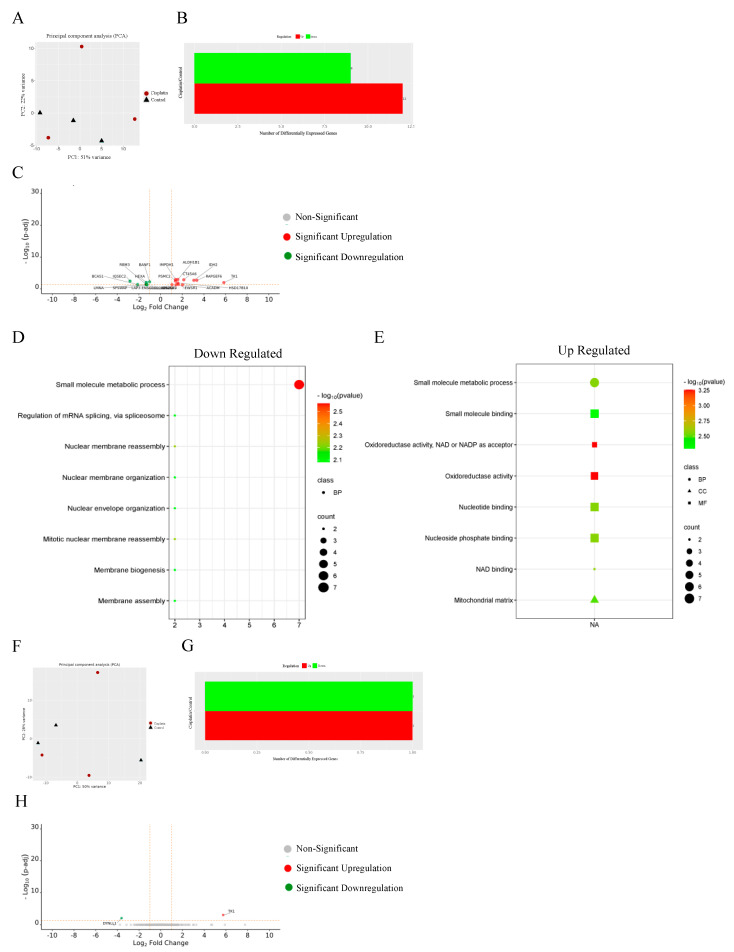
Proteomic analysis of MDA−MB−231 and HT29 upon treatment with cisplatin as described in Section 2.15: (**A**). MDA−MB−231 principal component analysis. (**B**). MDA−MB−231 number of differentially expressed proteins (DEPs). (**C**). MDA−MB−231 volcano plot of DEPs upregulated (red) and downregulated (green). GO enriched biological processes, cellular components and molecular function in MDA−MB−231, (**D**). Downregulated and (**E**). Upregulated proteins. (**F**). HT29 principal component analysis. (**G**). HT29 number of differentially expressed proteins (DEPs). (**H**). HT29 volcano plot of DEPs upregulated (red) and downregulated (green). Data points denote mean ± SEM. *n* = 3 from three independent experiments.

Quantitative proteomic analysis was employed to investigate the global proteome profile and anticancer/resistance effects of the investigated platinum complexes on MDA−MB−231 and HT29 cells treated with IC_50_ concentration of each complex at 72 h (Figure 13, Figure 14, Figure 15 and Figure 16). In total, we identified 1597 MDA−MB−231 and 1859 HT29 proteins across all paired samples that passed quality control in all the three replicates of each drug treatment. First, we performed the principal component analysis (PCA) to screen and compare the proteome profiles among treated vs. untreated cells for further analysis. PCA plots in Figure 14A,H, Figure 15A,H and Figure 16A,F show the treated samples are separated, which suggests that there are overall differences in proteome expression profiles in control vs. treated samples. The number of up- and downregulated proteins are shown across all the treatments and in both the cell line (*t*-test, *p* < 0.05, fold change > 2) (Figure 14B,I, Figure 15B,I and Figure 16B,G). Of all platinum complexes, **Pt^II^5ME*SS*** and **Pt^II^56ME*SS*** expressed the most significant dysregulation in proteins post-treatment in both cancers, suggesting the high potency of these complexes. While the **Pt^II^PHEN*SS*** (Figure S18), **Pt^IV^PHEN*SS*(OH)_2_** (Figure S19), **Pt^IV^5ME*SS*(OH)_2_** (Figure S20), **Pt^IV^56ME*SS*(OH)_2_** (Figure S21) and cisplatin did not exhibit a similar effect as **Pt^II^5ME*SS*** and **Pt^II^56ME*SS***, at 72 h post treatment. Cisplatin treated on HT29 cells did not have several differently expressed proteins; TK1 was commonly upregulated in both cancers (Figure 16C,H). Notably, MAPK1 was upregulated in **Pt^II^5ME*SS***- and **Pt^II^56ME*SS***-treated MDA−MB−231 cells (Figure 14C and Figure 15C). A similar case was noted for PIK3CB in **Pt^II^56ME*SS***-treated MDA−MB−231 cells (Figure 15C). Notably, microtubule-associated proteins, MAP9 and TUBA8, were significantly increased in HT29 **Pt^II^56ME*SS***-treated cells (Figure 15J). Cytochrome C gene (CYC1) was significantly upregulated in both cancers treated with **Pt^II^56ME*SS*** treatment (Figure 15C,J). **Pt^II^PHEN*SS*** differentially expressed 7 down- and 14 upregulated proteins in MDA−MB−231 and 10 down- and 9 upregulated proteins in HT29 cells (Figure S18B,E). **Pt^IV^PHEN*SS*(OH)_2_** had 5 down- and 9 upregulated differential proteins in MDA−MB−231 (Figure S19B), while no detected differentials in HT29 (Figure S19E). This may be linked to the time of treatment, where greater incubation may be required to detect the same effect observed in the platinum(II) precursor. **Pt^II^5ME*SS*** differentially expressed 75 down- and 91 upregulated proteins in MDA−MB−231 (Figure 14B) and 118 down- and 110 upregulated proteins in HT29 cells (Figure 14I). While its derivative, **Pt^IV^5ME*SS*(OH)_2_**, had 33 down- and 38 upregulated differential proteins in MDA−MB−231 (Figure S20B), and 3 detected up and down differentially regulated proteins in HT29 (Figure S20G). This may be linked to the time of treatment, where greater incubation may be required to increase the reduction to the platinum(II) precursor (**Pt^II^5ME*SS***) and activate the same effect observed. **Pt^II^56ME*SS*** differentially expressed 183 down- and 132 upregulated proteins in MDA−MB−231 (Figure 15B) and 228 down- and 200 upregulated proteins in HT29 cells (Figure 15I). While its derivative, **Pt^IV^56ME*SS*(OH)_2_** had only 3 down- and 8 upregulated differential proteins in MDA−MB−231 (Figure S21B), and 3 detected up and down differentially regulated proteins in HT29 (Figure S21F). This may be explained similarly to the effect observed by the **Pt^IV^5ME*SS*(OH)_2_** complex and active effect time. Next, the functional ontology enrichment analysis of drug-specific up- and downregulated proteins based on GO annotations was analysed. The results from all three GO biological processes, molecular functions and cellular components are displayed in Figure 14 and Figure 15. GO analysis identified the upregulated pathways across **Pt^II^5ME*SS*** and **Pt^II^56ME*SS*** treatments in MDA−MB−231 were linked to cell localisation, exocytosis, secretion and uptake (Figure 14E and Figure 15E). Synthesis-linked mechanisms were decreased, which affected glycolysis, DNA and RNA processing and translation initiation (Figure 14D and Figure 15D). While nuclear processes and reassembly were decreased in cisplatin-treated MDA−MB−231 cells (Figure 16D). HT29-treated cells with **Pt^II^5ME*SS*** and **Pt^II^56ME*SS*** exhibited reduced mitochondrial and microtubule matrix of cellular components and RNA binding of metabolic function (Figure 14K and Figure 15K). Additionally, there was an increased biological process of protein folding and response to endoplasmic reticulum stress. Increased cellular oxidant detoxification and peroxidase activity were also observed (Figure 14L and Figure 15L). Differentially expressed proteins (DEPs) considerably identified intersecting across all treatments in MDA−MB−231 and HT29 cells were reported in an UpSet plot (Figure 13A,B). MDA−MB−231 exhibited 1 DEP common to all significantly expressed proteins across all complexes. The second-most identified were 81 DEPs shared between **Pt^II^5ME*SS***- and **Pt^II^56ME*SS***-treated cells. While those that intersected in HT29-treated cells were only 14 DEPs across the **Pt^II^PHEN*SS***, **Pt^II^5ME*SS*** and **Pt^II^56ME*SS***, and the second-most identified were 176 DEPs shared between **Pt^II^5ME*SS***- and **Pt^II^56ME*SS***-treated cells. **Pt^II^56ME*SS*** treatment exceptionally exhibited the most DEPs with 165 in MDA−MB−231 and 225 in HT29 cells. While **Pt^II^5ME*SS*** treatment was the second-most-exhibited DEP with 22 in MDA−MB−231 and 34 in HT29 cells.

## 4. Discussion

The biological activity of the unconventional platinum(II) complexes **Pt^II^PHEN*SS***, **Pt^II^5ME*SS*** and **Pt^II^56ME*SS*** and their novel platinum(IV) di-hydroxido derivatives **Pt^IV^PHEN*SS*(OH)_2_**, **Pt^IV^5ME*SS*(OH)_2_** and **Pt^IV^56ME*SS*(OH)_2_** were studied. The complexes exhibit anticancer potency on several cancer cell lines [17,19]. Exploring the mechanism of action of the complexes relative to cisplatin is an important step in informing future in vivo investigations. The cytotoxicity of each complex was investigated on MDA−MB−231, MCF−7, HT29, A2780 and ADDP cell lines. A dose-dependent response was observed across all cell lines and complexes with their IC_50_s in the low micromolar range (Figure 1 and Table 1) compared to cisplatin, suggesting a potent anti-neoplastic effect with both platinum(II) and (IV) complexes. **Pt^II^56MESS** was the most potent complex with a cytotoxic effect > **Pt^II^5ME*SS*** > **Pt^II^PHEN*SS*** > **Pt^IV^56ME*SS*(OH)_2_** > **Pt^IV^5ME*SS*(OH)_2_** > **Pt^IV^PHEN*SS*(OH)_2_** across all cancer cell lines. Owing to the distinct reducing environment of cancer cells, platinum(IV) complexes are selectively activated intracellularly in hypoxic and low-pH conditions, thereby overcoming the common toxic effects of platinum(II) complexes [82,83,84,85]. The variability in the order of potency of these complexes between cell lines is likely associated with a combination of the properties of the complexes and variation in the genetic profile of each cell line [17,86,87]. To measure the selectivity of all the complexes to cancer cells over genetically stable cells, cytotoxicity was measured on breast epithelial cells (MCF10A) (Table 1 and Table 2). Although the IC_50_ across all the complexes was in the lower micromolar range, the SCI was greater in all complexes than in cisplatin, indicating improved selectivity of the complexes to cancer cells. Higher SCIs were observed with platinum(IV) complexes in MDA−MB−231 and HT29 with a range of 3–7. The cytotoxic effect of the platinum complexes was then tested on 3D-bioprinted MDA−MB−231 and HT29 cells, an in vitro model more closely simulating conditions found in tumours. Platinum complex cytotoxicity decreased from MDA−MB−231 2D to 3D networks to spheroids while maintaining > 11-fold greater potency than cisplatin (Figure 2, Figures S2 and S3, and Table 3). HT29 spheroids remained effective with >39-fold greater potency than cisplatin (Figure 2, Figure S4 and Table 3).

The cellular uptake of platinum complexes was then assessed on MDA−MB−231 and HT29 cells at 3 μM (Figure 3 and Figure S5, Tables S3 and S4). All complexes showed an increase in cell uptake compared to cisplatin up to 30 h. Both the platinum(II) and (IV) complexes exhibited a high intracellular/extracellular ratio (Tables S3 and S4) with **Pt^II^56ME*SS*** exhibiting the most rapid uptake from 0 h of treatment, and the highest mean cell concentration at 30 h of treatment compared to its platinum(IV) derivative and the other investigated platinum agents. Distinctly, the significant toxicity levels of the platinum(II) complexes are mirrored by the increased absorption of **Pt^II^56ME*SS*** compared to **Pt^IV^56ME*SS*(OH)_2_** together with a shortened plateau interval (Figure 3). The difference in cell uptake observed across **Pt^II^PHEN*SS***, **Pt^II^5ME*SS*** and **Pt^II^56ME*SS*** and their platinum(IV) derivatives can be ascribed to the square-planar structure, smaller size and hydrophobicity of the platinum(II) complexes (Scheme 1), factors that facilitate transport across the cell membrane. Cellular uptake is time-dependent, with complexes continuously accumulated within the cell over the 30 h period, with the exception of the HT29 cellular uptake of **Pt^II^5ME*SS*** and **Pt^II^56ME*SS***, which plateaus at later times. This difference in cell accumulation of the platinum(II) vs platinum(IV) complexes up to 30 h can translate to the cytotoxic effect observed in each complex at 72 h. This is consistent with the intracellular reduction and activation requirement of the platinum(IV) complexes to their platinum(II) precursor for activity. Considering the minimal intracellular uptake up to 30 h is comparatively low compared to their platinum(II) precursors, further justifies the reason for mechanism investigation at 72 h of treatment. 

The high intracellular/extracellular ratio suggests all investigated agents use an active mode of transport (Tables S3 and S4). The platinum complexes may in fact be absorbed via several modes of cell uptake. This includes passive, facilitated diffusion or active transport using transport proteins, which require energy in the form of ATP, in addition to via endocytosis [88,89]. Consequently, the mode of cell uptake was further investigated by ICP-MS. Active transport was investigated by studying the drug uptake after inhibiting active mechanisms of transport at 4 °C [90]. Uptake of all agents was significantly reduced compared to optimal conditions at 37 °C confirming the active transport mode is used. Although the concentration intracellularly was significantly low compared to optimal conditions, it remained > 15-fold higher than the extracellular concentration across all agents and both cancers, which indicates that other mechanisms of transport are included [91,92]. A significant decrease in cell uptake also occurred after blocking clathrin-mediated endocytosis [93] for MDA−MB−231 cells treated with **Pt^II^PHEN*SS*** (*p* < 0.01), **Pt^IV^PHEN*SS*(OH)_2_** (*p* < 0.01), **Pt^IV^5ME*SS*(OH)_2_** (*p* < 0.001), **Pt^IV^56ME*SS*(OH)_2_** (*p* < 0.0001) and HT29 cells treated with **Pt^II^PHEN*SS*** (*p* < 0.05), **Pt^II^5ME*SS*** (*p* < 0.05), **Pt^IV^5ME*SS*(OH)_2_** (*p* < 0.05), **Pt^II^56ME*SS*** (*p* < 0.0001), **Pt^IV^56ME*SS*(OH)_2_** (*p* < 0.05) and cisplatin (*p* < 0.01), indicating that the complexes can be internalised by clathrin-mediated endocytosis, as has been previously observed by cisplatin [94]. SLC7A5, also known as the LAT1 receptor, is overexpressed in cancer cells compared to normal cells since cancer proliferation greatly depends on nutrients like essential amino acids [95,96,97]. SLC7A5 receptor uptake was significantly decreased after blocking this route across all investigating agents. Blocking the transferrin receptor also notably resulted in a decrease in cellular uptake of all the complexes with significant decrease observed in **Pt^II^PHEN*SS*** (*p* < 0.05), **Pt^IV^5ME*SS*(OH)_2_** (*p* < 0.01), and **Pt^IV^56ME*SS*(OH)_2_** (*p* < 0.01) in MDA−MB−231 cells and **Pt^IV^PHEN*SS*(OH)_2_** (*p* < 0.05), **Pt^IV^5ME*SS*(OH)_2_** (*p* < 0.05), **Pt^II^56ME*SS*** (*p* < 0.0001) and **Pt^IV^56ME*SS*(OH)_2_** (*p* < 0.05) in HT29 cells indicating that the complexes may use TfR to enter the cells by transferrin-mediated endocytosis. TfR is a membrane receptor that binds to transferrin, the main iron-carrying protein, to regulate the amount of iron that enters the cell, which is a high requirement for the proliferation of cancer cells [98,99,100]. The complexes may be able to bind to transferrin just as iron does. This may contribute to improved selectivity for cancer cells over non-cancer cells since platinum would bind cancer cells more due to the overexpression of TfR, as has been previously observed by cisplatin [94,100]. The study shows the investigated platinum agents may be using multiple routes for transport intracellularly with active transport as the primary mode of cell uptake (Figure 4). These insights accentuate the advanced cellular uptake capabilities and prospective anticancer properties of these platinum complexes with **Pt^II^56ME*SS,*** highlighting its promise in future cancer therapy paradigms.

The cellular localisation experiment (Figure 5) was completed to study the accumulation of platinum in nuclear, cytoskeletal, membrane/particulate and cytosolic fractions. Most of the platinum in the complexes was significantly localised to the cytoskeleton compared to all other fractions in both cell lines. In contrast, cisplatin was found localised in the membrane/particulate fractions, as observed previously in A549 cells [101]. Additionally, **Pt^II^56ME*SS*** has been previously shown to localise significantly in the cytoskeletal fraction in MDA−MB−231 cells [26], with the current data extending this localisation to the other structurally related. This suggests the platinum complexes could bind to fibrous proteins and interrupt the stability of the cell structure contributing to cell death. In addition, some accumulation in the nucleus could suggest a non-covalent interaction with DNA or cross-linking with DNA base pairs.

To assess the mechanism of cell death linked to the cytotoxicity of each complex flow cytometry was used to identify the percent of cell death that was positive or negative for Annexin V and PI staining. This assay measures phosphatidylserine (PS) translocation to the outer membrane leaflet observed in both early and late apoptosis [102]. Additionally, cells in late apoptosis and necrosis lose their membrane integrity, thus becoming more permeable to the PI stain that binds to DNA [103]. The flow cell sorter sorts the cells into four quadrants. The percent viable cells will be negative for both Annexin V and PI in quadrant 4, while cells that have entered early apoptosis in quadrant 3 will be positive for only Annexin V. Cells that have entered late apoptosis will be positive for Annexin V and PI in quadrant 2, and necrotic cells will be Annexin V-negative and PI-positive (Figure 6). At 72 h of treatment, a significant decrease in viable cells across all treatments was observed in both cancer lines (Figure 6C,D). Early apoptotic cells significantly increased across all treatments in MDA−MB−231, while a significant increase in necrotic cells was only observed in cisplatin, **Pt^II^5ME*SS***- and **Pt^II^56ME*SS***-treated cells. In HT29 cells, cisplatin, **Pt^II^PHEN*SS***, **Pt^II^5ME*SS***, **Pt^II^56ME*SS*** and **Pt^IV^56ME*SS*(OH)_2_** showed significant increases in early apoptotic cells and necrotic cells in addition to **Pt^IV^5ME*SS*(OH)_2_** with an observed increase in **Pt^IV^PHEN*SS*(OH)_2_**-treated cells.

The cell cycle arrest profile was then studied (Figure 7). Cisplatin has been previously reported to arrest in the G0/G1, S or G2+M phases of the cell cycle where, if sufficient repairs are not made, the cells ultimately experience aberrant mitosis and cell death by apoptosis [104]. MDA−MB−231 cells treated with the investigated agents showed a notable decrease in the G0/G1 phase with an increase in the G2+M phase, significantly with **Pt^II^56ME*SS***-, **Pt^IV^5ME*SS*(OH)_2_**- and **Pt^IV^56ME*SS*(OH)_2_**-treated cells. Cisplatin-treated MDA−MB−231 cells showed a slight decrease in the G0/G1 phase and a small increase in the G2+M phase [104]. HT29-treated cells showed a notable decrease in the G0/G1 phase with an increase in the S phase for the investigated agents, while cisplatin-treated cells remained predominately in the G0/G1 phase. Additionally, **Pt^II^56ME*SS*** has been previously reported to arrest in the G2+M phase in MDA−MB−231 cells, suggesting its ability to disrupt the microtubule cytoskeleton [26,105,106,107].

Cellular arrest in the S phase suggests cell death may be reliant on signalling for DNA damage [108]. The production of ROS was then investigated. All investigated agents exhibited significant ROS production in both MDA−MB−231 and HT29 cells at 48 and 72 h, while significance was also observed at 24 h with the **Pt^II^5ME*SS*** and **Pt^II^56ME*SS*** treatments (Figure 8). This reinforces the view that the mechanism of cell death is linked to apoptosis, given excess cellular ROS induces damage to proteins, lipids, organelles and nucleic acids [109]. Metal-based complexes have been shown to produce ROS and hydroxyl radicals, which produce oxidative DNA adducts [110,111,112]. As has been previously observed in cisplatin, the stimuli of ROS production have been correlated to mitochondrial membrane potential disturbance and fragmentation [113,114] Numerous chemotherapeutic agents have suggested that the generation of ROS altered mitochondrial morphology. Accordingly, the mitochondrial membrane potential (MtMP) was investigated (Figure 9). Mitochondria are the engine of the cell, responsible for producing energy for metabolic processes that maintain regular cellular operations [115]. Chemotherapeutics (including metallodrugs) induce MtMP disruption, which induces the activation of the intrinsic mitochondrial apoptotic pathway [116]. The electrical potential difference between a cell’s extracellular and intracellular environment is reflected by MtMP, a crucial predictor of mitochondrial activity, which is indicative of bioenergetic cell stress once lost. The MtMP was significantly reduced across all treatments at 24, 48 and 72 h in both MDA−MB−231 and HT29 cells.

The effect of the platinum(II) and (IV) complexes on the microtubule cytoskeleton was further investigated by immunofluorescence using actin and tubulin fluorophore antibodies (Figure 10). Cellular morphological changes were observed in MDA−MB−231- (Figure 10A), MCF10A- (Figure 10I) and HT29- (Figure 10Q) treated cells compared to the control. Changes such as membrane blebbing, cell shrinkage, cytoskeleton collapse, and nuclear DNA breaking into fragments are all characteristic of apoptosis [117], result in cytoskeletal changes. For example, actin is involved in regulating DNA degradation for apoptosis. The actin–myosin complex is associated with membrane blebbing, which is triggered by caspase 3 cleavage [74,118]. This translates to the changes observed in phalloidin (F-actin) expression after treatment with the investigated agents and its increased intensity at the edge of the cell, at the membrane. Actin and tubulin expression dynamics varied, indicating complex-specific impacts on cytoskeletal components and potentially implicating distinct mechanisms of action or cellular responses to these treatments. The noticeable actin re-localisation to the nucleus in HT29-treated cells can be explained by DNA degradation observed after treatment with **Pt^II^5ME*SS*** and **Pt^II^56ME*SS*** at 72 h. As phalloidin intensity increased in the nucleus, the DAPI signal decreased, which indicates DNA damage (Figure S14) [76,119,120]. Microtubules consist of alpha- and beta-tubulin heterodimers that are essential for cell motility, mitosis and intracellular trafficking [121]. Tubulin plays a role in the cell stress response and is a chemotherapeutic target to disrupt the mitotic spindle-inducing mitotic arrest (G2+M) and cell death [121,122,123]. In this experimental framework, the complexes that are effective are expected to destabilise tubulin polymerisation, which exhibits reduced fluorescence compared to the control, while those that stabilise tubulin increase fluorescence. This will reflect the variation in tubulin in polymerised microtubules [26,124]. A filamentous distribution of tubulin was observed in the MCF10A cells, while MDA−MB−231 and HT29 cells exhibited changes in microtubule network architecture with an increase in tubulin aggregates at the perinuclear region and edge of the cell, which contributed to total tubulin expression in treated cells compared to control. This suggests the ability of the platinum(II) and (IV) complexes to disrupt microtubule dynamics reinforcing G2+M arrest, as has been shown previously with microtubule-targeting agents [26,125,126,127,128]. Collectively, these findings reveal that the investigated platinum-based complexes and cisplatin induce significant cytoskeletal alterations in both MDA−MB−231 and HT29 cells, impacting cell morphology, actin and tubulin distribution, and overall cellular integrity. These insights contribute to understanding the cytoskeletal dynamics associated with the anticancer activity of these complexes, offering potential avenues for further exploration in cancer therapy. 

The initial stage to tumour metastasis is the characteristic ability of cancer cells to invade adjacent tissues and blood vessels by chemotaxis, guided by protrusive activity from the cell membrane, its attachment to the extracellular matrix (ECM) and its ability to adhere [129,130,131]. Accordingly, the migrative and adhesive abilities of the invasive MDA−MB−231 [132,133] and HT29 [134,135] cells were studied with the investigated agents compared to the untreated control (Figure 11 and Figures S15–S17). At 72 h post-treatment, the wound width remained significantly wide in **Pt^II^5ME*SS***, **Pt^II^56ME*SS***, **Pt^IV^5ME*SS*(OH)_2_** and **Pt^IV^56ME*SS*(OH)_2_**, with significantly reduced confluency, indicative of the cells’ decreased ability to close the artificial wound in monolayer, which is linked to its reduced metastatic potential.

To study the cell death pathways involved in complex action, the expression of key pro- and anti-apoptotic markers, cell proliferation markers, microtubule markers and autophagy markers was assessed by Western blot analysis in MDA−MB−231 and HT29 cells 72 h post-treatment (Figure 12). All protein markers were normalised to GAPDH and then to their individual no-treatment control run on each gel. Proteins in the Bcl2 family play a critical role in the intrinsic mitochondrial pathway to apoptosis. The pro-apoptotic marker is Bax, and the anti-apoptotic is Bcl2. When this pathway is induced, Bcl2 levels decrease and release Bax, which permeabilises the mitochondrial membrane, releasing cytochrome c, which is another marker that is upregulated in intrinsic apoptosis [9,136]. Treatment with the investigated platinum-based agents in both MDA−MB−231 and HT29 exhibited an increased Bax/Bcl2 ratio indicating a pro-apoptotic mechanism across all treatments in HT29-treated cells and in **Pt^II^5ME*SS*** and **Pt^II^56ME*SS*** in MDA−MB−231-treated cells similar to cisplatin. Cytochrome c was also notably increased across all treatments in both cancers, which further confirms the importance of apoptosis in cell death.

The extrinsic signalling pathway that triggers programmed cell death includes the activation of transmembrane receptors containing death receptor motifs that are a part of the tumour necrosis factor (TNF) family and is measured by protein markers such as caspase 8 [137]. Caspase 8 activation cleaves BID to tBID (cleaved BID), which moves to the mitochondria and activates Bax [138]. Procaspase 8 was observed to decrease across all treatments, with a notable decrease in the platinum(II) complexes in HT29 and platinum(IV) in MDA−MB−231 compared to the control, suggesting the activation of caspase 8 forming cleaved caspase 8, which activates BID. This was confirmed with the notable increase in cleaved BID across the platinum(II) treatments. While the platinum(IV) complexes did increase BID, the increase was not as large. This may suggest a delayed bioreductive activation of the platinum(IV) dihydroxy complexes to their platinum(II) precursors, thus a delayed response in protein dysregulation.

The executionary pathway markers are common for the intrinsic mitochondrial and extrinsic pathway and include executionary caspases 9 and 3, which are activated to their cleaved form, holding effector activity to downstream proteins including activation of PARP-1, whose downstream cleavage is linked to an increase in DNA disintegration in the nucleus to prompt cellular death by apoptosis [9,139,140,141]. Western blot quantification showed a notable decrease in procaspase 3 and 9 in the platinum(II)-treated cancer cells (Figure 12C,G) with observed cleaved caspase 3 in HT29-treated cells across all treatments with a significant increase, confirming the active cleavage of procaspase 3. PARP-1 activity was decreased in both cancers across all treatments with cleaved PARP-1 in HT29-treated cells significantly increasing following **Pt^II^PHEN*SS***, **Pt^II^5ME*SS***, **Pt^II^56ME*SS***, **Pt^IV^5ME*SS*(OH)_2_** and **Pt^IV^56ME*SS*(OH)_2_** treatment. Taken together, these data imply that the platinum(II) and (IV) complexes induce the activation of both the mitochondrial intrinsic and extrinsic signal transduction mechanism of programmed cell death. 

Tumour suppressor proteins p53 and p21 were also studied (Figure 12B,F). In response to DNA damage, p53 is upregulated in the nucleus, triggering p21 transcription together with other response genes. The induction of p21 in the nucleus activates the DNA-damage cell cycle checkpoints at G1/S that induce cell cycle arrest by cyclin-dependent kinases [142,143]. Both p53 and p21 were quantified and showed increased expression in both MDA−MB−231- and HT29-treated cells (Figure 12F). p21 was particularly induced in HT29 cells, which may be linked to the S phase arrest observed by HT29 cells (Figure 7D).

The PI3K-AKT and Ras-ERK cell proliferation pathways have been linked to cell survival and ability to regulate cellular invasion and migration [144,145]. Apoptosis effectors are connected to the downstream effectors of both pathways. ERK controls the following substrates: p21, Bax and Bid. Phosphorylation and activation of ERK (p-ERK) causes it to translocate to the nucleus, where it activates transcription of survival genes [146,147,148]. Accordingly, inhibition of p-ERK is linked to pro-apoptotic protein activity with p-ERK kept localised to the cytoplasm [148,149]. ROS production has been associated with DNA damage through increased p53 and p21 activity, which is controlled by ERK (MAPK pathway) [150,151,152]. The p-ERK/ERK ratio in MDA−MB−231- and HT29-treated cells was reduced with a significant decrease in both platinum(II) and platinum(IV) complexes in MDA−MB−231-treated cells. AKT functions in inhibiting apoptosis and stopping the release of cytochrome c from the mitochondria by phosphorylating the Ser184 site on Bax to impede its pro-apoptotic effect while also phosphorylating Ser136 on BAD (Bcl2-associated death promoter), which dissociates from Bcl2, concomitantly blocking BAD-induced apoptosis [153,154]. Phosphorylation-mediated activation of AKT contributes to the inhibition of caspase 9 activity, in addition to the inhibition of MDM2 (an oncoprotein) which downregulates p53 [155]. The p-AKT/AKT ratio in MDA−MB−231- and HT29-treated cells was reduced across all treatments. A significant decrease in the ratio was observed for both platinum(II) and platinum(IV) complexes in HT29 cells. However, a significant decrease was only observed for the platinum(II) complexes in MDA−MB−231-treated cells (Figure 12B,F).

Microtubule cytoskeletal protein markers were also investigated. Western blot analysis showed reduced actin expression across the complexes compared to the control. α-tubulin and β-tubulin markers were also studied. α-tubulin exhibited a significant decrease across platinum(II)-treated MDA−MB−231 cells while a significant decrease was observed in **Pt^II^PHEN*SS***, **Pt^II^5ME*SS***, **Pt^II^56ME*SS*** and **Pt^IV^5ME*SS*(OH)_2_** in HT29-treated cells. β-tubulin was also reduced significantly across the investigated agents in HT29-treated cells, while MDA−MB−231-treated cells exhibited a significant reduction in expression with **Pt^II^5ME*SS***, **Pt^II^56ME*SS***, **Pt^IV^PHEN*SS*(OH)_2_**, **Pt^IV^5ME*SS*(OH)_2_** and **Pt^IV^56ME*SS*(OH)_2_** (Figure 12A,E), overall correlating with the observed dysregulation identified in immunofluorescence.

Autophagy could be another mechanism of cell death exhibited by the platinum(II) and (IV) complexes. Autophagy is a catabolic cellular process in which lysosomes break down unwanted proteins and organelles. Beclin-1, an autophagy marker, and the Bcl2 complex are related to the inhibition of autophagy-linked cell death. When Bcl2 is downregulated, the complex is free to crosstalk between apoptosis and autophagy [156]. Autophagy signal pathways connect with AKT, which phosphorylates and obstructs TSC1/2, which then activates mTOR and cellular survival. Accordingly, the inhibition of PI3K signal pathways implies a potential inhibition of mTOR, resulting in activated autophagy-linked markers [157,158]. ATG core proteins (autophagy-related proteins) are characterised into functional groups. The class III phosphatidylinositol 3-kinase complex includes Beclin-1, ATG14, PIK3C3/VPS34 and PIK3R4/p150/VPS15. The proteins ATG7, ATG10, ATG12, ATG16L1 and ATG5 are part of the ATG12 conjugation system. The ATG4, ATG7, ATG3 and WIPI2 proteins, and the LC3 protein family are components of the microtubule-associated protein 1 light chain 3 (LC3) conjugation system, as well as the ATG9 trafficking system, including the ATG2A, ATG2B, WIPI4 and the transmembrane protein ATG9A markers [159,160,161]. ATG proteins are key markers of autophagy with ATG5 crosstalk with apoptosis [159,162]. ATG5 is involved in the monitoring of the quality of the mitochondria after oxidative damage and subsequent cellular longevity, as well as vesicular formation during autophagy. ATG5 complexes with ATG16L1, along with other ATGs, instruct the lipidation and formation of other proteins required for autophagy [163]. Additionally, ATG9A controls the subtleties of cell protrusions and directed migration as well as lipid utilisation from lipid droplets [164]. ATG4B interacts with LC3B, which allows the autophagosome to fuse with lysosomes [165,166,167]. The ATG4B and Bcl2 interaction also plays an important role in apoptosis and autophagy crosstalk by upregulating during mitochondrial damage and decreasing Bcl2 expression. ATG4B can also disassociate the Beclin-Bcl2 complex to activate autophagy [168,169]. Beclin-1 expression was notably increased in **Pt^II^56ME*SS***-treated MDA−MB−231 cells while an increase was observed in HT29-treated cells with cisplatin, **Pt^II^56ME*SS***, **Pt^IV^PHEN*SS*(OH)_2_**, **Pt^IV^5ME*SS*(OH)_2_** and **Pt^IV^56ME*SS*(OH)_2_**. While no increase was observed in APG5L/ATG5 protein, ATG16L1 and ATG9A, their activity cannot be ruled out to produce lipids and proteins required for vesicular formations in the presence of autophagy. ATG4B and LC3B exhibited an increase in expression compared to the control, with LC3B expression being more prominently increased in **Pt^II^PHEN*SS***-, **Pt^II^5ME*SS***-, **Pt^II^56ME*SS***- and cisplatin-treated cells (Figure 12D,H). This suggests the presence of the autophagosome and autophagy may be another mechanism targeted by the platinum complexes.

An overall multi-mechanistic potential is observed among our platinum(II) and (IV) complexes, which may be advantageous in limiting drug resistance to cancer. It has been suggested that combination drugs that use different mechanisms of action may increase the efficacy of treatment in the clinic because, for example, if either mechanistic pathway of cell death were to acquire resistance to a drug that activates both pathways, the alternative may still be of benefit [170,171].

The proteome profile of all investigated complexes was studied. Across all treatments, there was a difference relative to the control as observed by each relevant PCA plot (Figure 14, Figure 15 and Figure 16). The most disruptive complexes were identified to be **Pt^II^5ME*SS*** and **Pt^II^56ME*SS***, given their high number of dysregulated proteins. While, **Pt^II^PHEN*SS***, **Pt^IV^PHEN*SS*(OH)_2_**, **Pt^IV^5ME*SS*(OH)_2_**, **Pt^IV^56ME*SS*(OH)_2_** and cisplatin did not exhibit a similar effect at 72 h of treatment. This can be explained by the significant difference in the number of DEPs, suggesting a time course treatment should be further investigated considering the reduced activity required by the platinum(IV) derivatives to lose their axial ligand and act like their platinum(II) precursors [172]. The inhibition of transcription is thought to be the primary mechanism by which traditional platinum complexes cause apoptotic cell death [111]. The commonly upregulated gene observed in cisplatin for both cancers was TK1, which is an indicator of DNA damage [173]. Notably, MAPK1 was upregulated in both **Pt^II^5ME*SS***- and **Pt^II^56ME*SS***-treated MDA−MB−231 cells (Figure 14C and Figure 15C), an unexpected but possible transitionary effect identifying its activation in response to cellular stress [174], comparable to the inhibition noted via Western blot analysis (Figure 12B). The same observation was seen by PIK3CB in **Pt^II^56ME*SS***-treated HT29 cells (Figure 15J), also suggested to be in response to cell stressors, given that the AKT pathway has been suppressed post-treatment as observed by Western blot analysis (Figure 12F). CYC1 was significantly upregulated in both cancers treated by **Pt^II^56ME*SS*** (Figure 15C,J), which was also observed by Western blot analysis in cytochrome c (Figure 12C,G). This confirms the ability of **Pt^II^56ME*SS*** to induce intrinsic apoptotic cell death. Microtubule-Associate Protein 9 (MAP9) and Tubulin Alpha 8 (TUBA8) were significantly increased in HT29 **Pt^II^56ME*SS***-treated cells (Figure 15J), which additionally suggests a blockade at a transitional phase in cell signalling in the presence of cell stressors, while the Western blot analysis exhibited the inhibitory effect of alpha and beta tubulin markers (Figure 12E). The platinum(II) precursors generally exhibited greater activity than their platinum(IV) derivatives. This proposes that the time of treatment may need to be investigated at greater than 72 h for the platinum(IV) derivatives in order to obtain a similar effect after they have released their axial ligand and can act like their platinum(II) precursors [172].

Gene ontology analysis identified the overall up- and downregulated pathways. Upregulated pathways across **Pt^II^5ME*SS*** and **Pt^II^56ME*SS*** treatments in MDA−MB−231 were linked to cell localisation, exocytosis, secretion and uptake (Figure 14E and Figure 15E), which may be correlated to the active transport mechanism identified in Figure 4 across our platinum(II) and (IV) complexes. Downregulated pathways included a synthesis-linked mechanism, which affected glycolysis, DNA translation and RNA processing (Figure 14D and Figure 15D). These findings suggest the ability of **Pt^II^56ME*SS*** to inhibit cellular proliferation. Nuclear processes and reassembly were decreased in cisplatin-treated MDA−MB−231 cells (Figure 16D), proposing cisplatin-induced DNA damage [14]. HT29-treated cells with **Pt^II^5ME*SS*** and **Pt^II^56ME*SS*** exhibited downregulation of mitochondrial and microtubule matrix of cellular components and RNA binding (Figure 14K and Figure 15K) in addition to upregulation in protein folding and response to endoplasmic reticulum stress. Increased cellular oxidant detoxification and peroxidase activity was also observed (Figure 14L and Figure 15L). This suggests induced cellular damage in response to treatment such as ROS-induced DNA damage [175,176]. **Pt^II^56ME*SS*** treatment exhibited the most DEPs with 165 in MDA−MB−231 and 225 in HT29 cells supporting it as the most potent multi-mechanistic complex for chemotherapy. Protein network generation (Figure 14F,G,M,N and Figure 15F,G,M,N) highlighted the most modulated pathways by **Pt^II^5ME*SS*** and **Pt^II^56ME*SS***, which included reduced activity of nucleotide, RNA and actin filament binding and metabolic processes, and an increase in nucleoside phosphate and protein binding, cellular localisation and active ion transmembrane activity.

## 5. Conclusions

To conclude, the platinum(II) complexes and platinum(IV) derivatives exhibited potent anti-neoplastic activity towards MDA−MB−231 and HT29 cancer cells. The mode of cellular uptake was primarily by active transport, with **Pt^II^56ME*SS*** being the most accumulated intracellularly and the primary localisation of all the complexes observed in the cytoskeleton. The complex’s mechanism of action included significant ROS production and reduced MtMP. Morphological changes to the actin and tubulin cytoskeleton were also observed with increased aggregation indicative of cellular damage. They also induced apoptosis via an interplay between the intrinsic and extrinsic pathways and through the inhibition of the MAPK and PI3K pathways. It is also possible that autophagy-dependent cell death is involved. Targeting key pathways of disease and limiting resistance make multi-mechanistic drugs important. Our study has established that these complexes affect multiple pathways; however, it is not clear from this study what the specific molecular target(s) are. While being unable to define a single mechanism of action might be considered a limitation of this study, the use of these complexes with multiple mechanisms of action may be a clinical advantage to circumvent cancer resistance. This study suggests that this class of complex may demonstrate the characteristics of an efficient and potentially safe anticancer prodrug in breast and colorectal cancers. To validate the safety, efficacy and selectivity of the complexes under investigation, future in vivo research is needed. 

## 6. Patents

This work is part of Australian Provisional Patent Application 2022900110, Platinum(IV) complexes, February 2022, Western Sydney University, Sydney, Australia.

## Figures and Tables

**Table 1:** IC_50_ values (µM) of platinum(II) and (IV) complexes and cisplatin on MCF−7, MDA−MB−231, HT29, A2780, ADDP and MCF10A cell lines.

	IC_50_ (µM)					
	MCF−7	MDA−MB−231	HT29	A2780	ADDP	MCF10A
Cisplatin	32.99 ± 1.35 ^a^	13.08 ± 1.36	77.76 ± 1.97	21.50 ± 1.55	112.00 ± 2.02	4.71 ± 1.47
**Pt^II^PHEN*SS***	1.83 ± 2.12 *** ^b^	1.01 ± 1.36 **	0.76 ± 1.47 ****	1.11 ± 1.88 **	2.55 ± 1.48 ****	3.49 ± 1.39 NS
**Pt^II^5ME*SS***	0.86 ± 1.67 ***	0.52 ± 1.94 **	0.55 ± 1.42 ****	0.65 ± 1.71 ***	1.32 ± 1.33 ****	1.18 ± 1.75 NS
**Pt^II^56ME*SS***	0.56 ± 2.08 ***	0.14 ± 1.48 **	0.18 ± 1.49 ****	0.19 ± 1.17 ***	0.76 ± 1.27 ****	0.41 ± 1.52 NS
**Pt^IV^PHEN*SS*(OH)_2_**	9.39 ± 1.26 ***	3.34 ± 1.37 **	4.48 ± 1.77 ****	6.49 ± 1.23 **	13.64 ± 1.57 ****	20.46 ± 1.32 **
**Pt^IV^5ME*SS*(OH)_2_**	2.16 ± 1.53 ***	2.79 ± 1.39 **	3.64 ± 1.45 ****	2.14 ± 1.38 ***	7.71 ± 1.31 ****	12.22 ± 1.48 *
**Pt^IV^56ME*SS*(OH)_2_**	1.36 ± 1.47 ***	0.71 ± 1.22 **	1.25 ± 1.38 ****	1.69 ± 1.36 ***	4.17 ± 1.55 ****	4.91 ± 1.45 *

^a^ Data are mean ± SEM of three independent determinations. ^b^ Significance of IC_50_s compared to cisplatin is identified by * *p* < 0.05, ** *p* < 0.01, *** *p* < 0.001 and **** *p* < 0.0001, as measured by an unpaired Student’s *t*-test. NS = No Significance.

**Table 2:** Selective cytotoxicity index of platinum(II) and (IV) complexes and cisplatin in all cancer cell lines.

	MCF−7	MDA−MB−231	HT29	A2780	ADDP
Cisplatin	0.14	0.36	0.06	0.22	0.04
**Pt^II^PHEN*SS***	1.91	3.45	4.59	3.14	1.37
**Pt^II^5ME*SS***	1.37	2.27	2.15	1.82	0.89
**Pt^II^56ME*SS***	0.73	2.93	2.28	2.16	0.54
**Pt^IV^PHEN*SS*(OH)_2_**	2.18	6.13	4.57	3.15	1.49
**Pt^IV^5ME*SS*(OH)_2_**	5.66	4.38	3.36	5.71	1.58
**Pt^IV^56ME*SS*(OH)_2_**	3.61	6.92	3.93	2.90	1.18

SCI = IC_50_ (normal epithelial cell line, MCF10A)/IC_50_ (cancer cell line).

**Table 3:** IC_50_ values (µM) of platinum(II) and (IV) complexes, and cisplatin on MDA−MB−231 3D networks, MDA−MB−231 spheroids and HT29 spheroids.

${\mathrm{IC}}_{50}\;(\mu$M)	MDA−MB−231 3D Networks	MDA−MB−231 Spheroids	HT29 Spheroids
Cisplatin	15.99 ± 1.49 ^a^	48.47 ± 1.62	444.80 ± 5.09
**Pt^II^PHEN*SS***	2.84 ± 1.56 ** ^b^	5.44 ± 1.35 ****	3.93 ± 1.46 ****
**Pt^II^5ME*SS***	2.04 ± 1.31 **	4.08 ± 1.37 ****	2.91 ± 1.39 ****
**Pt^II^56ME*SS***	0.98 ± 1.21 **	2.49 ± 1.98 ****	0.99 ± 1.29 ****
**Pt^IV^PHEN*SS*(OH)_2_**	6.18 ± 1.62 *	13.49 ± 1.25 ****	11.40 ± 1.31 ****
**Pt^IV^5ME*SS*(OH)_2_**	3.82 ± 1.42 **	7.99 ± 1.30 ****	8.46 ± 1.23 ****
**Pt^IV^56ME*SS*(OH)_2_**	1.40 ± 1.34 **	4.59 ± 1.35 ****	3.16 ± 1.32 ****

^a^ Data are mean ± SEM of three independent determinations. ^b^ Significance of IC_50_s compared to cisplatin is identified by * *p* < 0.05, ** *p* < 0.01 and **** *p* < 0.0001, as measured by an unpaired Student’s *t*-test. NS = No Significance.

## Data Availability

All data relevant to the publication are included.

## Supplementary Materials

The following are available online at https://www.mdpi.com/article/10.3390/cancers16142544/s1, Table S1: Matrix Conditions selected for RASTRUM bioprinting. Figure S1: Calibration curve generated by plotting the peak areas measured by the ICP-MS against known concentrations (^195^Pt(STD)). Table S2: Conditions and parameters selected on the ICP-MS machine. Figure S2: MDA−MB−231 networks. Figure S3: MDA−MB−231 spheroids. Figure S4: HT29 spheroids. Figure S5: ICP-MS analysis for the uptake of Pt in MDA−MB−231 and HT29 at 0, 0.5, 1, 3, 6, 12, 24 and 30 h. Table S3: Kinetic Cellular uptake of Pt complexes by MDA−MB−231. Table S4: Kinetic Cellular uptake of Pt complexes by HT29. Figure S6: Mode of uptake of Platinum in MDA−MB−231. Figure S7: Mode of uptake of Platinum in HT29. Figure S8: Cellular Localisation of Platinum in MDA−MB−231. Figure S9: Cellular Localisation of Platinum in HT29. Figure: S10: ROS production upon treatment with platinum(II) and (IV) complexes in MDA−MB−231 and HT29 at 0, 0.25, 0.5, 1, 3, 6, 12, 24, 48 and 72 h. Table S5: ROS production upon treatment with complexes in MDA−MB−231 and HT29 cells at 24, 48 and 72 h. Table S6: Mitochondrial membrane potential upon treatment with platinum(II) and platinum (IV) complexes, and cisplatin in MDA−MB−231 and HT29 cells at 24, 48 and 72 h. Figure S11: Effect of platinum complexes and cisplatin on β−tubulin and F−actin in MDA−MB−231. Figure S12: Effect of platinum complexes and cisplatin on β−tubulin and F−actin in MCF10A. Figure S13: Effect of platinum complexes and cisplatin on β−tubulin and F−actin in HT29. Figure S14: Relation between phalloidin and DAPI after **Pt^II^5ME*SS*** and **Pt^II^56ME*SS*** treatment. Figure S15: Cell migration. Figure S16: Cell migration in MDA−MB−231. Figure S17: Cell migration in HT29. Figure S18: Proteomic analysis of MDA−MB−231 and HT29 upon treatment with Pt**^II^PHEN*SS***. Figure S19: Proteomic analysis of MDA−MB−231 and HT29 upon treatment with **Pt^IV^PHEN*SS*(OH)_2_**. Figure S20: Proteomic analysis of MDA−MB−231 and HT29 upon treatment with **Pt^IV^5ME*SS*(OH)_2_**. Figure S21: Proteomic analysis of MDA−MB−231 and HT29 upon treatment with **Pt^IV^56ME*SS*(OH)_2_**. Figure S22: Proteomic analysis of MDA−MB−231 and HT29 upon treatment with **Pt^II^5ME*SS***. Figure S23: Proteomic analysis of MDA−MB−231 and HT29 upon treatment with **Pt^II^56ME*SS***. Table S7: Antibody Concentrations used in Western Blot. Figure S24: Full representative western blot of microtubule cytoskeleton protein markers in MDA−MB−231. Figure S25: Full representative western blot of cell proliferation protein markers in MDA−MB−231. Figure S26: Full representative western blot of intrinsic and extrinsic apoptotic cell death markers in MDA-MB-231. Figure S27: Full representative western blot of autophagy markers in MDA−MB−231. Figure S28. Full representative western blot of microtubule cytoskeleton protein markers in HT29. Figure S29: Full representative western blot of cell proliferation protein markers in HT29. Figure S30: Full representative western blot of intrinsic and extrinsic apoptotic cell death markers in HT29. Figure S31: Full representative western blot of autophagy markers in HT29.
