# Supplementary material for: Anticancer Effect of PtIIPHENSS, PtII5MESS, PtII56MESS and Their Platinum(IV)-Dihydroxy Derivatives against Triple-Negative Breast Cancer and Cisplatin-Resistant Colorectal Cancer
Source: Cancers (Basel). 2024 Jul 15;16(14):2544. doi: 10.3390/cancers16142544 (PMC11274883; doi:10.3390/cancers16142544)
Supplement: Supplementary file 1 [file cancers-16-02544-s001.zip › cancers-3060979-supplementary.pdf]

Supplementary

Anticancer Effect of Pt^{II}PHEN*SS*, Pt^{II}5ME*SS*, Pt^{II}56ME*SS* and Their Platinum(IV)-Dihydroxy Derivatives against Triple-Negative Breast Cancer and Cisplatin-Resistant Colorectal Cancer

Maria George Elias [1,2], Shadma Fatima [2,3], Timothy J. Mann [2,3], Shawan Karan [1], Meena Mikhael [1], Paul de Souza [4], Christopher P. Gordon [1], Kieran F. Scott [2,3,*] and Janice R. Aldrich-Wright [1,3,*]

1 School of Science, Western Sydney University, Sydney, NSW 2751, Australia; m.elias3@westernsydney.edu.au (M.G.E.); shawan.karan@westernsydney.edu.au (S.K.); m.mikhael@westernsydney.edu.au (M.M.); c.gordon@westernsydney.edu.au (C.P.G.)

2 Medical Oncology, Ingham Institute for Applied Medical Research, Liverpool, NSW 2170, Australia; s.fatima@westernsydney.edu.au (S.F.); tim.mann@westernsydney.edu.au (T.J.M.)

3 School of Medicine, Western Sydney University, Sydney, NSW 2751, Australia

4 Nepean Clinical School, Faculty of Medicine and Health, University of Sydney, Kingswood, NSW 2747, Australia; paul.desouza@sydney.edu.au

* Correspondence: kieran.scott@westernsydney.edu.au (K.F.S.); j.aldrich-wright@westernsydney.edu.au (J.R.A.-W.); Tel.: +61-246203218 (J.R.A.-W.)

Method S1. Nano Proteomics Method

The digested protein samples were analyzed by UPLC-MS using a Waters nanoAcquity UPLC sample manager fitted with a binary solvent manager. Mass spectrometric detection was conducted using a Waters Synapt G2-Si. Separation consisted of two mobile phases. Mobile Phase A (0.1% formic acid in Milli-Q water) and Mobile Phase B (0.1% formic acid in ACN). The trapping column was a Waters nanoEase M/Z Symmetry C18 trap column (180 µm x 20 mm) and the analytical column was a Waters nanoAcquity UPLC 1.7um BEH130 C18 column (75 µm x 100 mm) thermostatted to 40 °C. Elution was achieved at a flow rate of 0.3 µL/min with each sample run for 55 minutes. The gradient was 0 min 1% B; 2 min 10% B; 40 min 40% B; 42 min 85% B and 50 min 85% B. System specific cleaning and equilibration protocols were run before each sample.
Mass spectrometry was conducted in positive ion mode with a capillary voltage of 3 kV and a sampling cone voltage of 30 V as well as a source offset of 30 V for electrospray ionizations. The source temperature was set at 80 °C. A desolvation source of nitrogen gas at 20 L/h and a desolvation temperature of 350 °C was used. Lock spray ion acquisition was conducted every 300 seconds with [Glu1]-fibrinopeptide B as the reference compound. Data acquisition was conducted over the mass to charge range of 50–2000. The data independent acquisition used an HDMSe experiment employing both low and high energy collision-induced dissociation of parent ions. Low energy collision was done at 6 V in the trap collision cell and at 4 V in the transfer collision cell. High energy collision used a collision energy ramp from 17 to 60 V in the transfer collision cell. Scan time was 0.5 seconds and after each scan the system would switch from high to low energy collision.
Protein identification was carried out using Progenesis QI for Proteomics and the UniProt Homo Sapiens reference database with the following conditions. The parent and fragment ion tolerances were set to automatic, the allowed maximum missed cleavages was set to 1, the allowed false discovery rate was set to 4% and the maximum protein size was set to 250 kDa. The peptide modifications were carbamidomethyl C (fixed) and oxidation M (variable). The ion matching requirements were fragments/peptide of 1 or more, fragments/protein of 3 or more and peptides/protein of 1 or more. Relative protein quantitation was performed with the Hi-N method using the top 3 peptides. Progenesis QI automatically selects unique reporter peptides to quantify a protein and measures their UPLC peak areas in the total ion chromatogram.

Method S2. Syntheses of Platinum(II) Complexes and Platinum(IV) Dihydroxy Complexes

The synthesis of [Pt(1*S*,2*S*-diaminocyclohexane)]Cl_2

Potassium tetrachloroplatinate (K_2PtCl_4; 500.8 mg, 1.21 mmol) and 1*S*,2*S*-diaminocyclohexane (1*S*,2*S*-dach, 1 mol eq.) were dissolved in deionised water (d.i.H_2O). The mixture was stored at 4 ºC for 48 h. The solution turns opaque as the yellow precipitate forms. The solution was filtered and the product, [Pt(1*S*,2*S*-dach)Cl_2], was washed sequentially with d.i.H_2O, ethanol, ether, and then air dried. The product, [Pt(1*S*,2*S*-dach)Cl_2], was stored in a desiccator [1-5].

The general synthesis of [Pt(P_L)(1*S*,2*S*-diaminocyclohexane)]$(NO_3)_2$,
[Pt(1*S*,2*S*-dach)Cl_2] (490.5 mg, 1.42 mmol) and a polyaromatic ligand P_L (Phen, 5Mephen or 56Me_2phen) (P_L; 1.1 mol eq.) in d.i.H_2O (100 mL) were refluxed for 24 h at 100 ºC. The refluxed solution was an opaque yellow mixture and became a clear yellow solution when the

reaction was completed. The reaction solution volume was reduced to roughly 20 mL *via* rotary evaporation for purification *via* a Vac 20cc (5 g) C_{18} Sep-Pak© column connected to a Bio-Rad pump with UV detector (Bio-Rad, EM-1 Econo™ UV Monitor). The C_{18} Sep-Pak© column was activated by eluting initially with methanol (20 mL) and subsequently flushing with d.i.H_2O (20 mL) until the UV absorbance had equilibrated. The concentrated reaction product was loaded onto the column and eluted with H_2O. Fractions of the eluting yellow band were collected. The fractions, were reduced under vacuum using a rotary evaporator and then lyophilised, producing pale yellow solids.

To convert each platinum(II) complex from chloride to the nitrate salt, each solid was dissolved in a minimal volume of d.i.H_2O and silver nitrate ($AgNO_3$; 2 mol eq.) was then added. The solution was stirred in the dark overnight, and the resulting silver chloride (AgCl) precipitate was syringe filtered to isolate the solution containing the platinum(II) complex nitrate salt. The nitrate solutions of **Pt^{II}PHEN*SS***, **Pt^{II}5ME*SS*** or **Pt^{II}56ME*SS*** (Scheme 1) were then reduced under vacuum and lyophilised to produce pale yellow solid.

The successful synthesis and purity of the platinum(II) complexes was confirmed by by high performance liquid chromatography (HPLC), ^{1}H nuclear magnetic resonance (NMR), ^{1}H-^{195}Pt heteronuclear multiple quantum coherence (HMQC) NMR and circular dichroism (CD) spectroscopy [1-5]. HPLC chromatograms were determined by eluting the platinum complexes through an Agilent Technologies Infinity HPLC machine, equipped with a Phenomenx Onyx™ Monolithic C_{18}-reverse phase column (100 × 4.6 mm, 5 µm pore size). The mobile phase consisted of solvents, A (0.06% trifluoroacetic acid (TFA) in d.i.H_2O) and B (0.06% TFA in ACN/d.i.H_2O (90:10)). An injection volume of 5 µL was utilised and eluted with a 0 – 100% linear gradient over 15 min with a flow rate of 1 mL/min, within the set wavelengths of 214 and 254 nm. ^{1}H-MR was determined for each complex by dissolving the complex (5 mg) in deuterium oxide (D_2O) (500 µL). ^{1}H NMR was carried out on a 400 MHz Bruker Avance Spectrometer at 298 K. All samples were prepared to a concentration of 10 – 20 mM in 450 – 600 µL using D_2O. ^{1}H NMR was set to 10 ppm and 16 scans with a spectral width of 8250 Hz and 65536 data points. ^{1}H-^{195}Pt HMQC was carried out using a spectral width of 214436 Hz and 256 data points for ^{195}Pt nucleus, F1 dimension, also a spectral width of 4808 Hz with 2048 data points for ^{1}H nucleus, F2 dimension. All resonance recorded were presented as chemical shifts in parts per million (δ ppm). A Jasco J-810 CD spectrophotometer was used to measure the CD spectra of the purified platinum(II) and platinum(IV) complexes. The samples were prepared in d.i.H_2O, with a concentration of 0.05 mM and a 1 mm optical glass cuvette was used. CD experiments were performed in the range of 350–200 nm (20 accumulations) with a bandwidth of 1 nm, data pitch of 0.5 nm, a response time of 1 sec, and a 100 nm/min scan speed. The flowrate of nitrogen gas was 8 L/min. Excel) was used to process the spectral data.

The general synthesis of $[Pt(P_L)(1S,2S\text{-diaminocyclohexane})(OH)_2](NO_3)_2$

Platinum(IV) complexes of the type $[Pt^{IV}(P_L)(1S,2S\text{-dach})(OH)_2]^{2+}$ were synthesised using platinum(II) products (**Pt^{II}PHENSS**, **Pt^{II}5MESS** or **Pt^{II}56MESS**) obtained previously after the nitrate conversion step. Oxidation was undertaken by adding 8 mL of 30% hydrogen peroxide to the solution, which was then heated at 70 ºC for 3 h in the dark to obtain, **Pt^{IV}PHEN*SS* $(OH)_2$**, **Pt^{IV}5ME*SS* $(OH)_2$** or **Pt^{IV}56ME*SS*$(OH)_2$** derivatives (Scheme 1). The resulting reaction solution was lyophilised overnight to obtain a yellow to brown precipitate. The crude lyophilised platinum(IV) product was dissolved in minimal amount of d.i.H_2O (3–5 mL) and excess ACN added to precipitate out the crude product which appears as a fine almost white powder when dried. The successful synthesis and purity of the platinum(IV) dihydroxy

complexes was confirmed with previously reported literature using HPLC and NMR [1-5]. Yields are reported in Table H.S1.

Result S2. Characterisation and Yields of Platinum(II) Complexes and Platinum(IV) Dihydroxy Complexes.

HPLC, NMR and CD were used to assess the structure and purity of the complexes for biological testing, passing for greater than 95% purity. The obtained yield and purity of the platinum (II) and their platinum(IV) dihydroxy derivatives were reported in Table H.S1 and chromatograms, which include the retention time (t_R) are reported in Figures HS1-HS6.The obtained ^1H and ^1H-^{195}Pt-HMQC NMR spectra are tabulated in Table N.S1 and represented in Figures N.S7–S18. The obtained HPLC chromatograms (Figure H.S-S6) and NMR spectra (Figure N.S7–S18) matched those of previously published data, which confirmed the successful synthesis and isolation of our product of interest [1-3,5]. The CD experiments confirm the retention of chirality of the 1*S*,2*S*-dach. The obtained results for the platinum(II) and platinum(IV) complexes were comparable to what is reported in the literature [3]. All the characteristic peaks from CD spectra of the purified complexes are summarised in Table H.S1.

Table H.S1. Summary of the characterisation data of the platinum(II) and (IV) complexes.

Complexes	Yields [mg]	Yields [%]	t_R [min]	Purity [%]	CD λ_{max} [nm] (mdeg.mol / L × 10^1)
**Pt^{II}PHEN*SS***	550	89	5.152	99.59	207 (–210.46), 228 (+168.45), 247 (–52.97), 257 (+15.56), 309 (–33.44), 334 (+127.32)
**Pt^{II}5ME*SS***	620	84	5.599	99.03	208 (+361.54), 226 (+416.85), 247 (+296.63), 261 (+300.04), 290 (–92.00), 335 (+144.51)
**Pt^{II}56ME*SS***	490	75	5.990	99.93	209 (–137.92), 228 (+150.21), 241 (–49.52), 268 (+71.07), 291 (–46.77), 310 (+42.38), 338 (+126.86)
**Pt^{IV}PHEN*SS*(OH)$_2$**	307	90	4.092	99.99	202 (–596.81), 269 (–44.98), 286 (+45.70)
**Pt^{IV}5ME*SS*(OH)$_2$**	380	88	4.132	99.43	205 (– 463.98), 208 (–503.54), 253 (+9.05), 269 (–41.70), 298 (+57.97)
**Pt^{IV}56ME*SS*(OH)$_2$**	257	92	4.441	99.55	202 (–517.15), 212 (–425.03), 255 (+28.35), 284 (–27.59)

Table N.S1. Summary of NMR spectroscopy data of platinum(II) and (IV) complexes, showing the chemical shifts (ppm), integration, multiplicity and coupling constants. [a]

**Proton Labels**	**Complexes**					
	**Pt^{II}PHEN*SS***	**Pt^{II}5ME*SS***	**Pt^{II}56ME*SS***	**Pt^{IV}PHEN*SS*(OH)$_2$**	**Pt^{IV}5ME*SS*(OH)$_2$**	**Pt^{IV}56ME*SS*(OH)$_2$**
H2/H9	8.8 (d, 2H, *J* = 5.4 Hz)	8.80 (m, 3H)	8.8 (d, 2H, *J* = 8.5 Hz)	9.2 (d, 2H, *J* = 6.2 Hz)	9.1 (d, 2H, *J* = 8.3 Hz)	9.2 (d, 2H, *J* = 8.6 Hz)
H4/H7	8.8 (d, 2H, *J* = 8.3 Hz)	9.1 (d, 2H, *J* = 8.3Hz),	9.1 (d, 2H, *J* = 5.3 Hz)	9.1 (d, 2H, *J* = 8.9 Hz)	9.1 (d, 2H *J* = 8.5 Hz)	9.1 (d, 2H, *J* = 5.5 Hz)
H5/H6	8.0 (s, 2H)	7.59 (s, H6, 1H)	–	8.3 (s, 2H)	8.1 (s, H6, 1H),	–
H3/H8	7.9 (dd, 2H, J_1 = 5.4 Hz, J_2 = 8.3 Hz)	7.9 (m, 2H, J_1 = 5.4 Hz, J_2 = 8.3 Hz)	7.9 (dd, 2H, J_1 = 5.4 Hz, J_2 = 8.6 Hz)	8.2 (dd, 2H, J_1 = 5.5 Hz, J_2 = 8.3 Hz)	8.2 (dd, 2H, J_1 = 5.5 Hz, J_2 = 8.1Hz)	8.2 (dd, 2H, J_1 = 8.6 Hz J_2 = 5.5 Hz)
CH_3	–	2.65 (s, CH_3, 3H)	2.59 (s, 6H)	–	2.9 (s, CH_3, 3H)	2.8 (s, 2 × CH_3, 6H),
H1'/H2'	2.7 (m, 2H)	2.7 (m, 2H)	2.6 (m, 2H)	3.2 (m, 2H)	3.1 (m, 2H)	3.1 (m, 2H)
H3'/H6' equatorial	2.2 (d, 2H, *J* = 13.3 Hz)	2.2 (d, 2H),	2.22 (d, 2H, *J* = 13.3 Hz)	2.39 (d, 2H, J = 10.8 Hz)	2.38 (m, 2H; CH_2)	2.38 (d, 2H, *J* = 11 Hz)
H4'/H5' equatorial and H3'/H6' axial	1.64 (d, 2H, *J* = 8.6 Hz)	1.65 (d, 2H), 1.49 (d, 2H)	1.66 (d, 2H, *J* = 8.6 Hz)	1.69 (m, 4H)	1.69 (m, 4H)	1.68 (m, 4H)
H4'/H5' axial	1.23 (m, 2H)	1.24 (m, 2H).	1.03 (m, 2H)	1.30 (m, 2H)	1.31 (m, 2H)	1.30 (m, 2H)
$^1H/^{195}Pt$	8.88/–2821, 8.09/–2821	8.80/–2810, 7.95/–2810	8.87/–2822, 8.87/–2822	9.32/440; 8.27/440	9.10/422, 8.25/422	9.20/410, 8.23/410

[a] Experiments were performed in D_2O. Accordingly, ammine or hydroxido resonances are not observed due to proton exchange. [b] Symmetrical complexes, thus H2/9, H3/8, H4/7 and H5/6 are equivalent protons.

HPLC Chromatograms

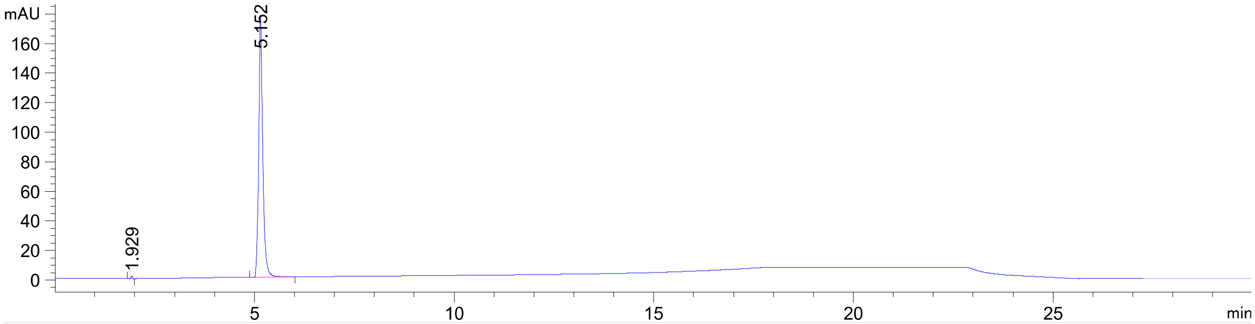

Figure H.S1. The HPLC chromatogram of [**Pt^{II}PHEN*SS***]$(NO_3)_2$, at 254 nm and 298K was acquired by a Phenomenex Onyx^{TM} Monolithic C18-reverse phase column (100 × 4.6 mm, 5 μm pore size). t_R at 5.152

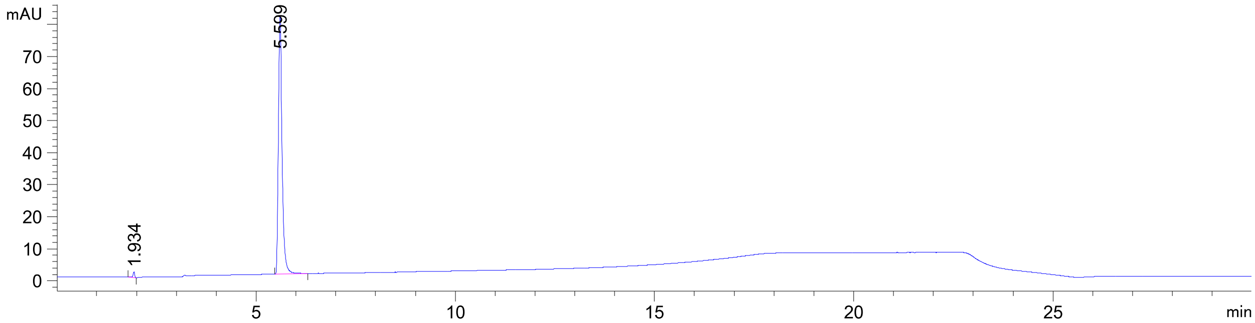

Figure H.S2. The HPLC chromatogram of [**Pt^{II}5ME*SS***]$(NO_3)_2$, at 254 nm and 298K was acquired by a Phenomenex Onyx^{TM} Monolithic C18-reverse phase column (100 × 4.6 mm, 5 μm pore size). t_R at 5.599 obtained.

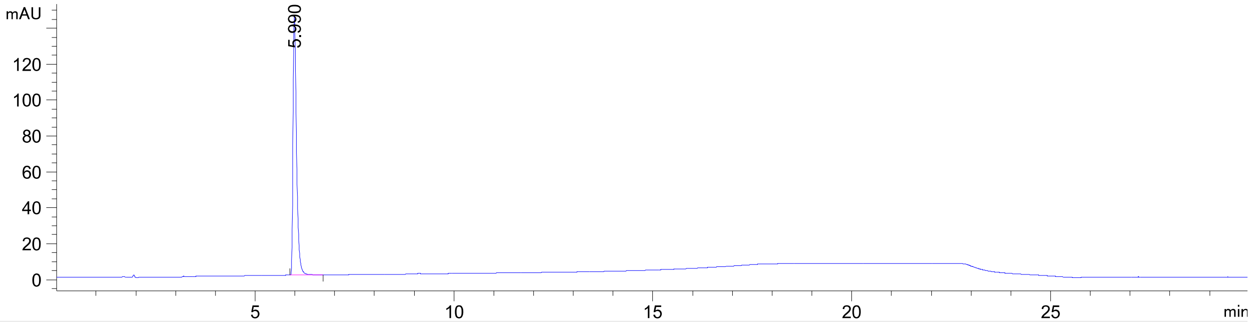

Figure H.S3. The HPLC chromatogram of [**Pt^{II}56ME*SS***]$(NO_3)_2$, at 254 nm and 298K was acquired by a Phenomenex Onyx^{TM} Monolithic C18-reverse phase column (100 × 4.6 mm, 5 μm pore size). t_R at 5.990.

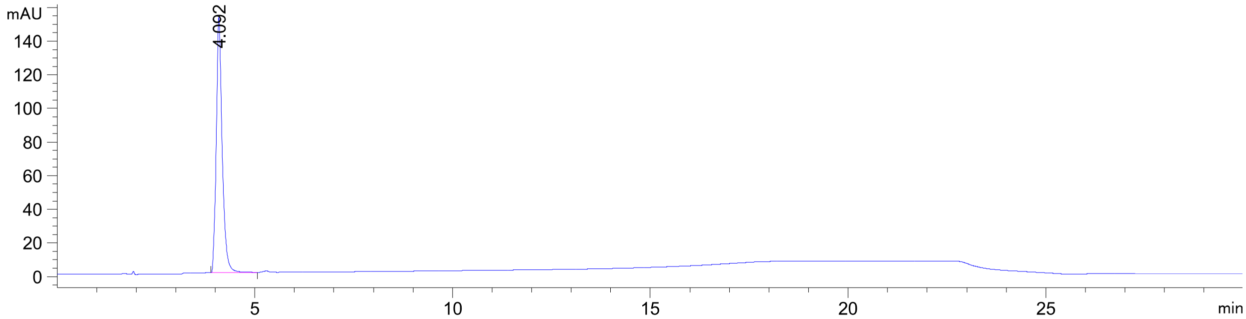

Figure H.S4. The HPLC chromatogram of [**Pt^{IV}PHEN*SS*(OH)$_2$**]$(NO_3)_2$, at 254 nm and 298K was acquired by a Phenomenex Onyx^{TM} Monolithic C18-reverse phase column (100 × 4.6 mm, 5 μm pore size). t_R at 4.092.

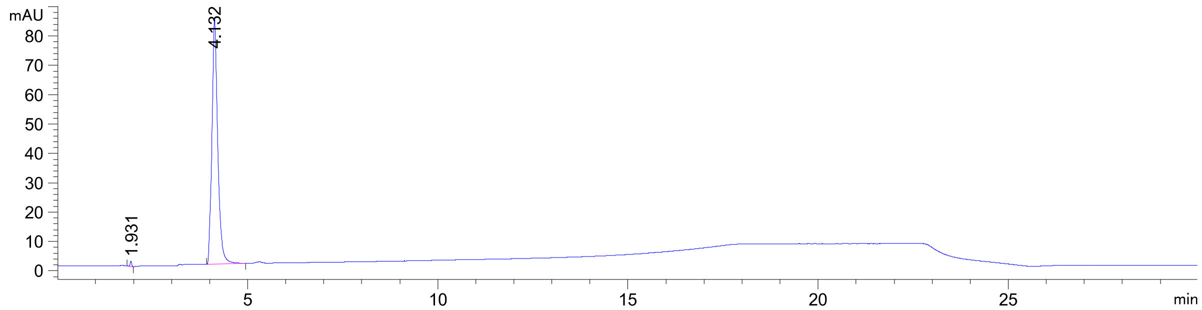

Figure H.S5. The HPLC chromatogram of [**Pt^{IV}5ME*SS*(OH)$_2$**]$(NO_3)_2$, at 254 nm and 298K was acquired by a Phenomenex Onyx^{TM} Monolithic C18-reverse phase column (100 × 4.6 mm, 5 µm pore size). t_R at 4.132.

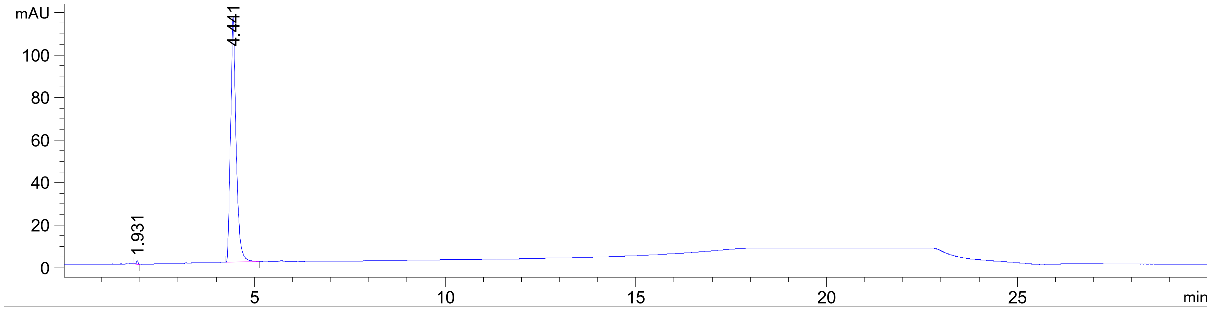

Figure H.S6. The HPLC chromatogram of [**Pt^{IV}56ME*SS*(OH)$_2$**]$(NO_3)_2$, at 254 nm and 298K was acquired by a Phenomenex Onyx^{TM} Monolithic C18-reverse phase column (100 × 4.6 mm, 5 µm pore size). t_R at 4.441.

^{1}H NMR and ^{1}H-^{195}Pt HMQC Spectra

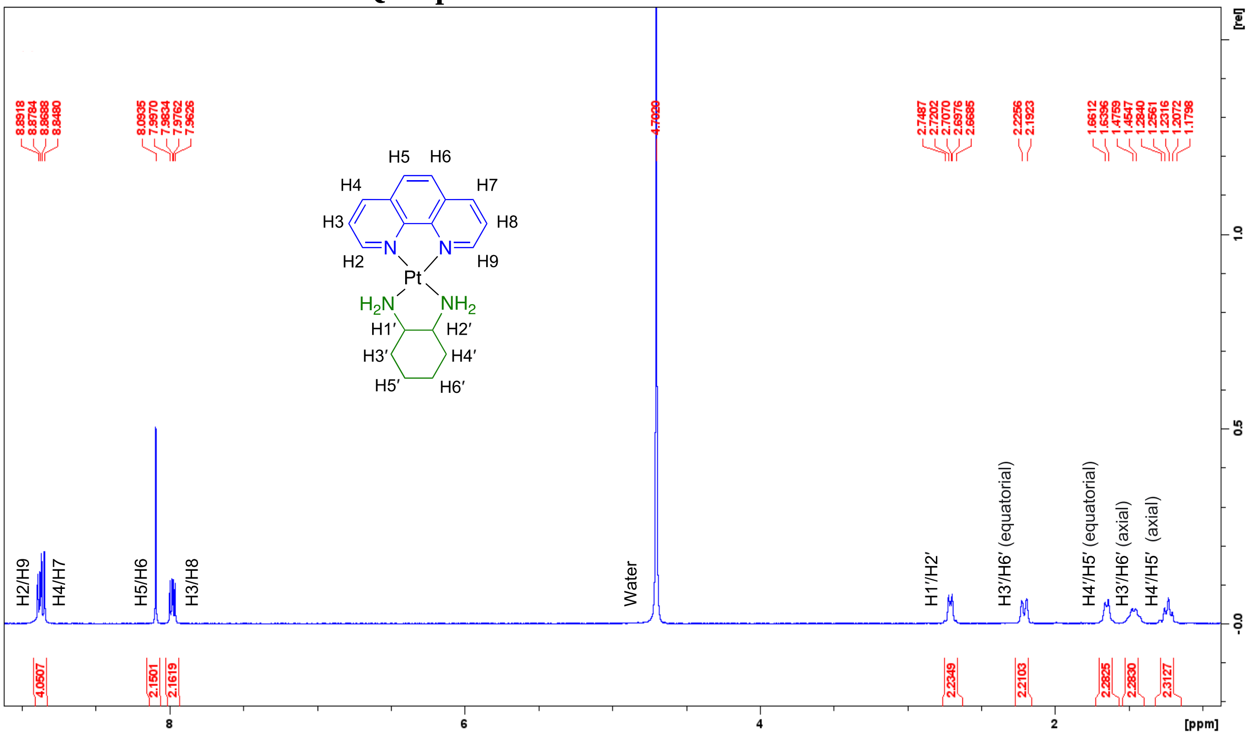

Figure N.S7. ^{1}H NMR spectra of **Pt^{II}PHENSS** in D_2O obtained at 298 K. Inset: structure of **Pt^{II}PHENSS**, with proton labelling system.

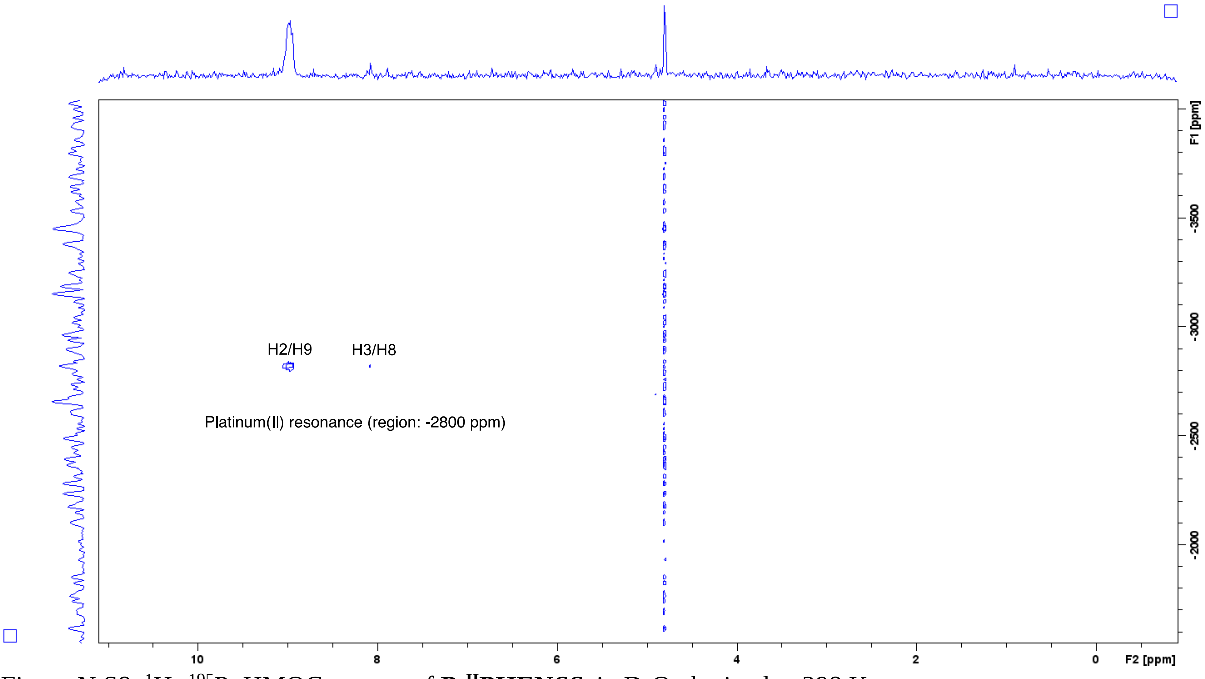

Figure N.S8. ^{1}H -^{195}Pt HMQC spectra of **Pt^{II}PHENSS**, in D_2O obtained at 298 K.

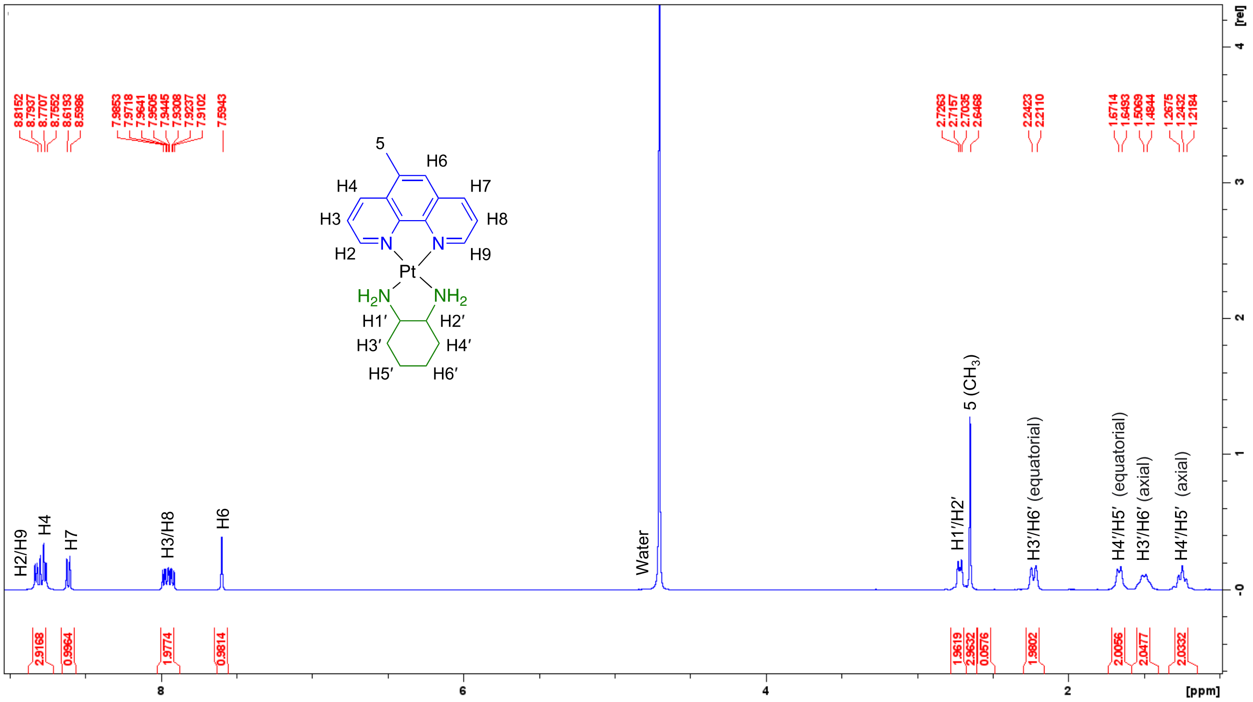

Figure N.S9. ^{1}H NMR spectra of **Pt^{II}5ME*SS*** in D_2O obtained at 298 K. Inset: structure of **Pt^{II}5ME*SS***, with proton labelling system.

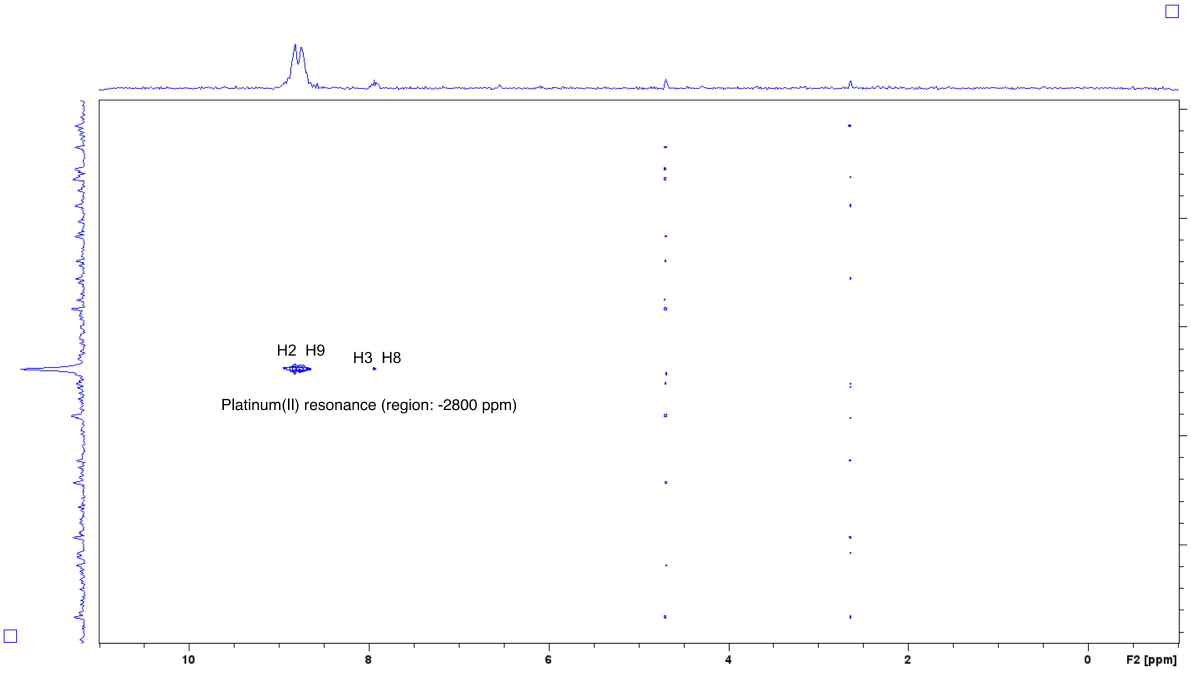

Figure N.S10. ^{1}H -^{195}Pt HMQC spectra of **Pt^{II}5ME*SS*** in D_2O obtained at 298 K.

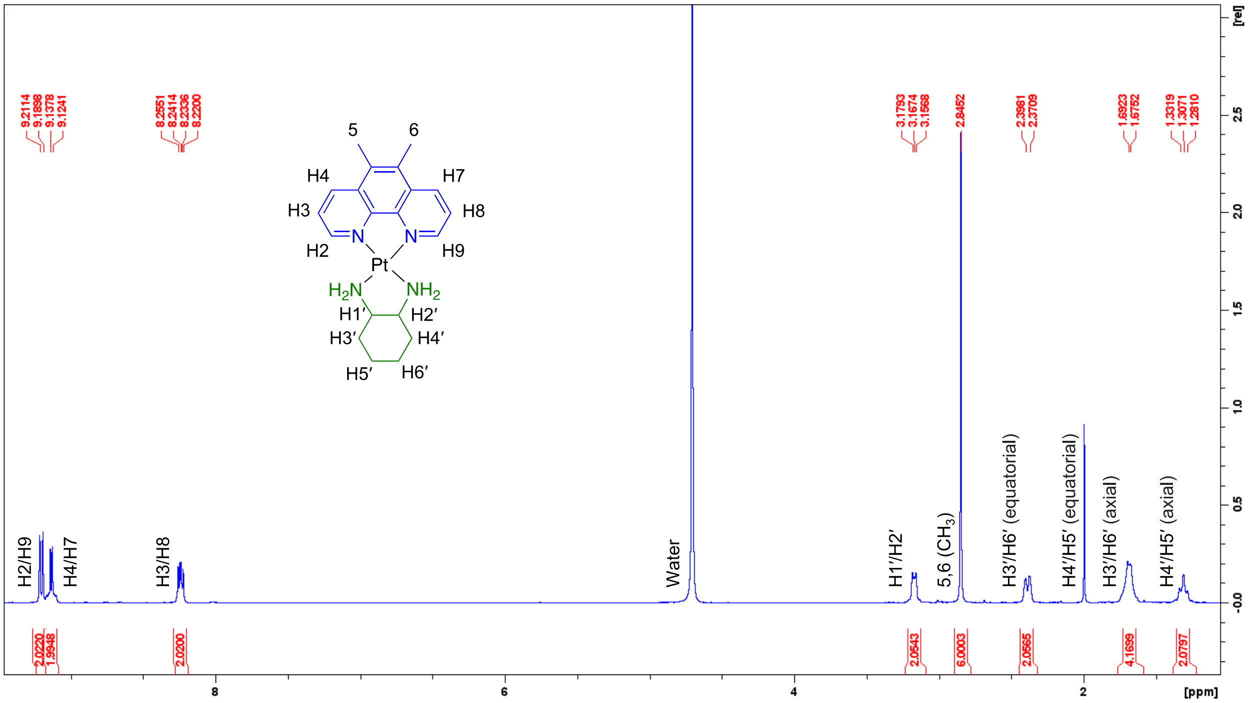

Figure N.S11. ^{1}H NMR spectra of **Pt^{II}56ME*SS*** in D_2O obtained at 298 K. Inset: structure of **Pt^{II}56ME*SS***, with proton labelling system.

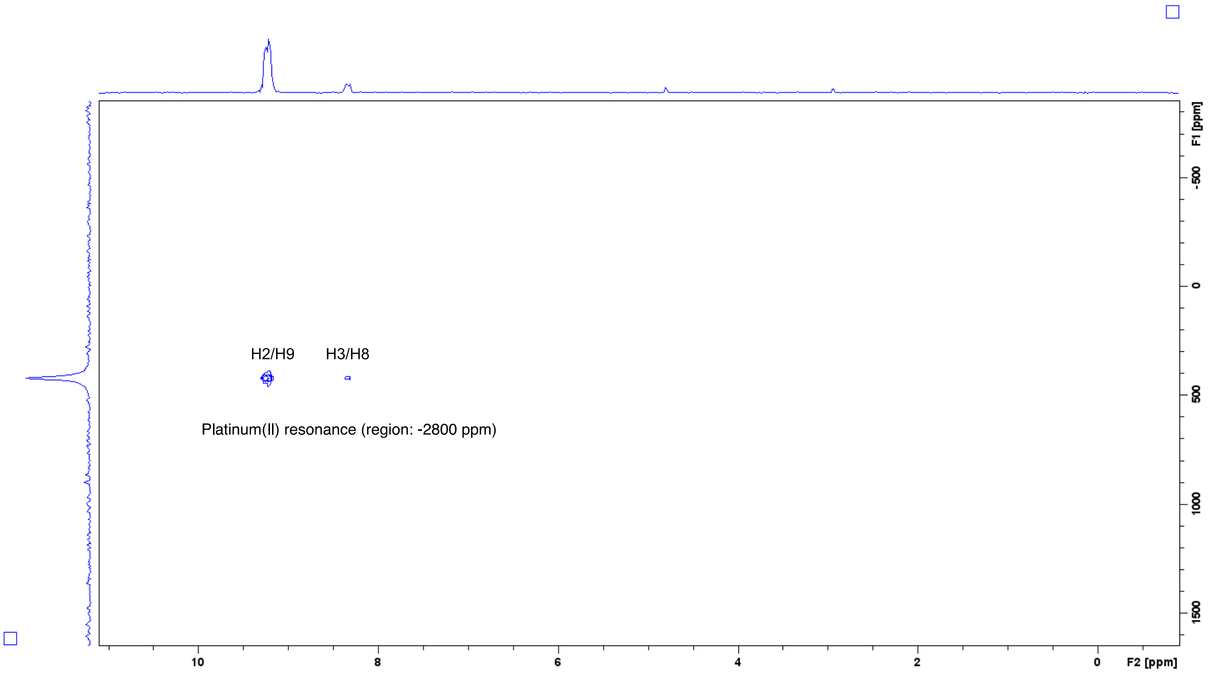

Figure N.S12. ^{1}H -^{195}Pt HMQC spectra of **Pt^{II}56ME*SS*** in D_2O obtained at 298 K.

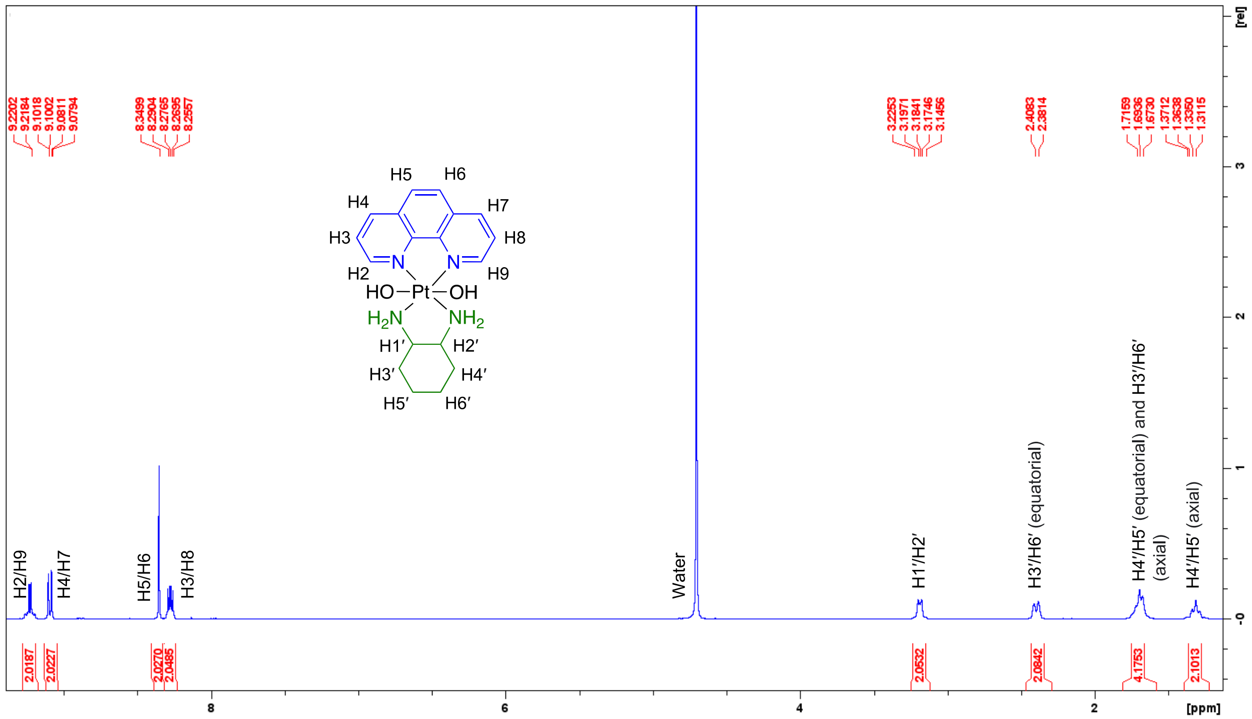

Figure N.S13. ^{1}H NMR spectra of **Pt^{IV}PHEN*SS*(OH)$_2$** in D_2O obtained at 298 K. Inset: structure of **Pt^{IV}PHEN*SS*(OH)$_2$**, with proton labelling system.

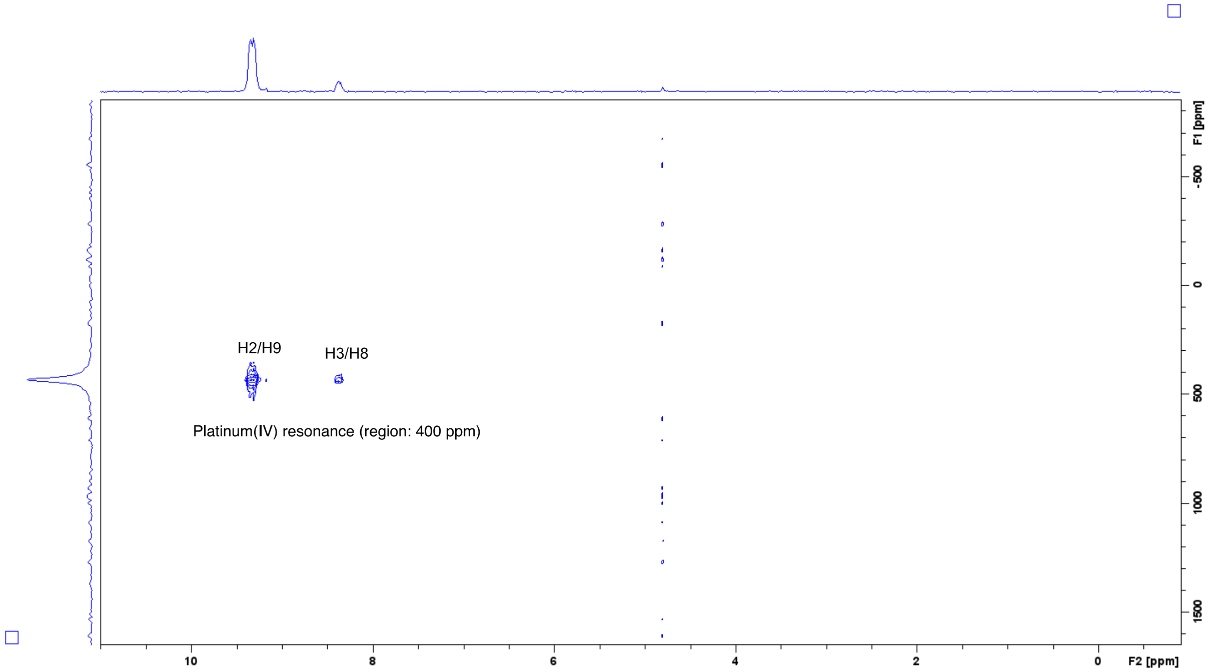

Figure N.S14. ^{1}H -^{195}Pt HMQC spectra of **Pt^{IV}PHEN*SS*(OH)$_2$** in D_2O obtained at 298 K.

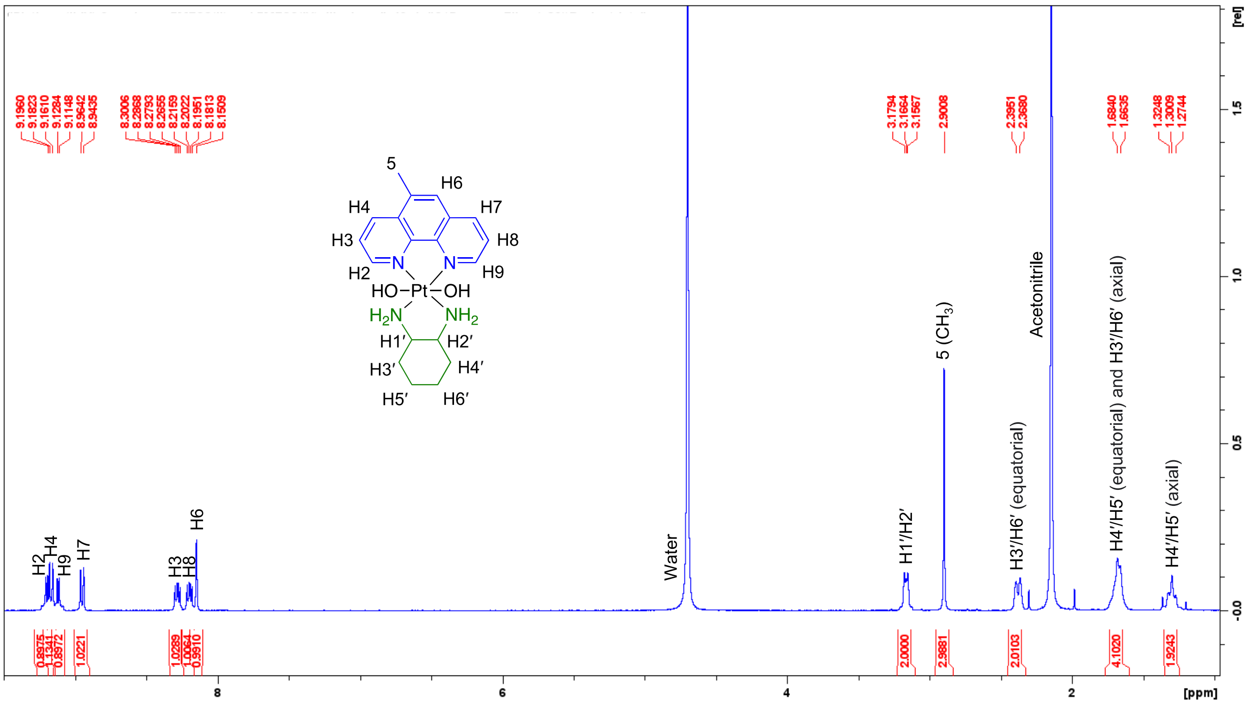

Figure N.S15. ^{1}H NMR spectra of **Pt^{IV}5MESS(OH)$_2$** in D_2O obtained at 298 K. Inset: structure of **Pt^{IV}5MESS(OH)$_2$**, with proton labelling system.

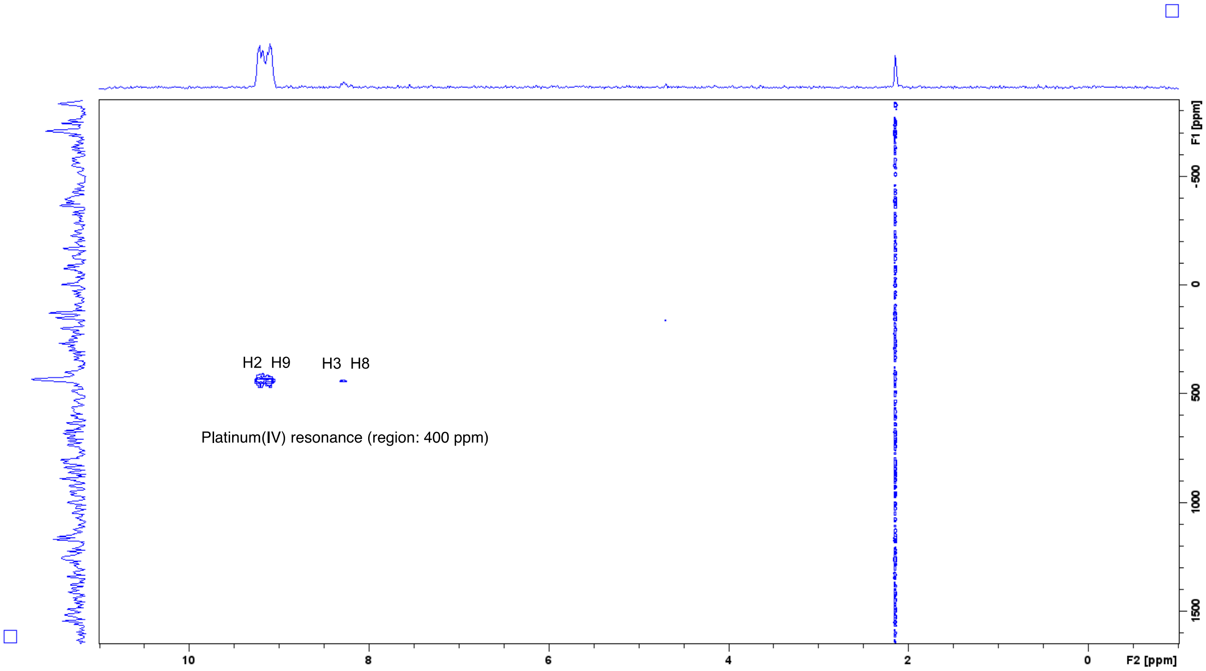

Figure N.S16. ^{1}H -^{195}Pt HMQC spectra of **Pt^{IV}5MESS(OH)$_2$** in D_2O obtained at 298 K.

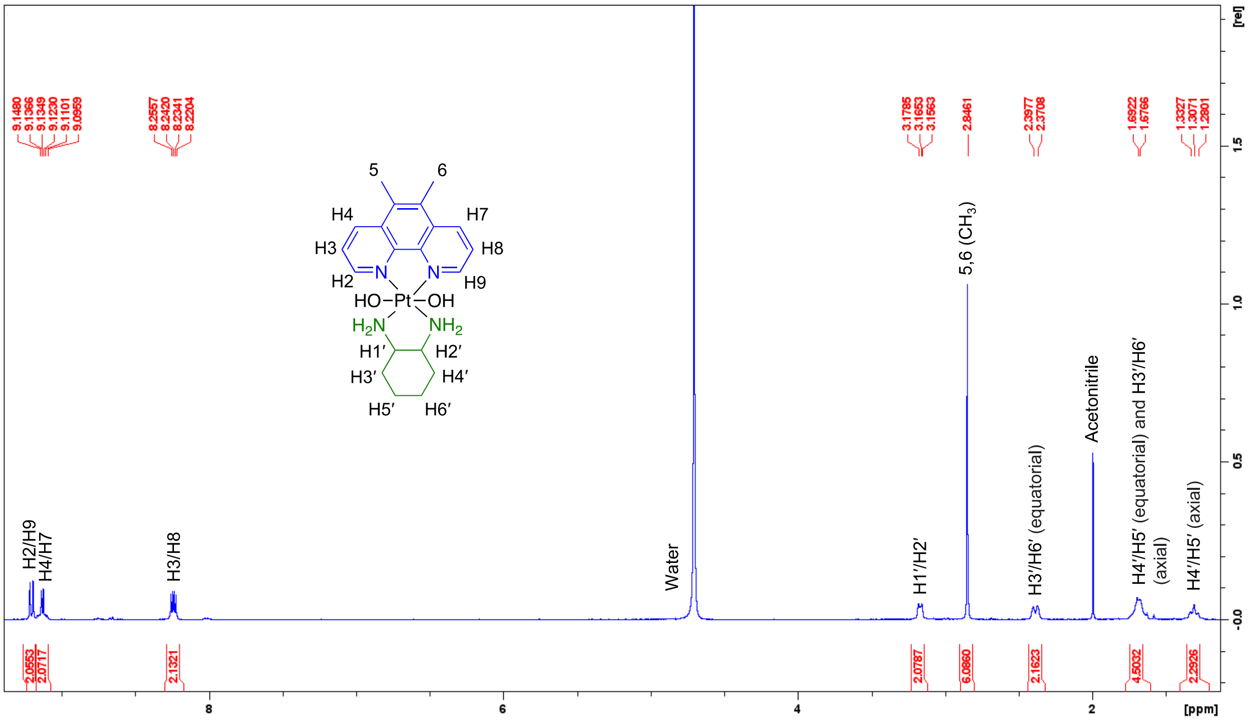

Figure N.S17. ^1H NMR spectra of **Pt^{IV}56ME*SS*(OH)$_2$** in D_2O obtained at 298 K. Inset: structure of **Pt^{IV}56ME*SS*(OH)$_2$**, with proton labelling system.

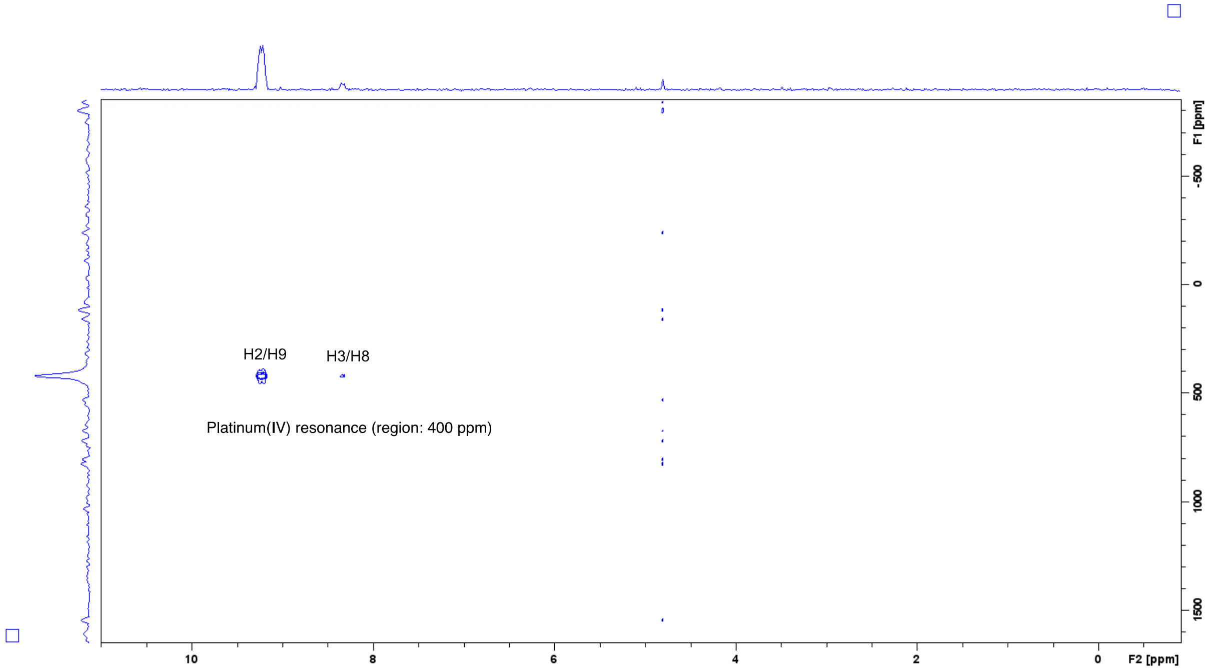

Figure N.S18. ^1H -^{195}Pt HMQC spectra of **Pt^{IV}56ME*SS*(OH)$_2$** in D_2O obtained at 298 K.

Method S3. Cellular Uptake of Platinum(II) and (IV) complexes

A final concentration of 10^6 cells/well of MDA−MB−231 and HT29 cells were seeded in 6-well plates and incubated overnight to adhere. The cells were then treated with a final concentration of 3 µM of either Pt^{II} or Pt^{IV} complexes. After 0, 0.5, 1, 3, 6, 12, 24 or 30 h, the medium was removed, and the cells were washed three times with cold PBS and allowed to dry. Then, 400 µL of 69% HNO_3 (Baseline grade nitric acid with Instrument Quality Grade Nitric acid with ppb level reported impurities, Seastar Chemicals, Sidney, Canada) was added to each well for 90 mins to ensure complete digestion. The digests were then moved to trace-metal free 15 mL centrifuge tubes (Labcon CA, USA), to which 7 mL milliQ water was then added which resulted in a final acid concentration of 3.5-4%. Iridium 193 (^{193}Ir), selected as internal standard, has similar first ionisation potential and mass to Platinum 195 (^{195}Pt). A concentration of 50 ppb ^{193}Ir in 2% HNO_3 solution was chosen for the Perkin Elmer NexION® Inductively Coupled Plasma Mass Spectrometer (ICP-MS) as it produced a steady state signal intensity of 600-800K counts per second. Mixing and injection of sample and internal standard solution was carried out using an ESI 7 port Valve system (Elemental Scientific, USA). A high-quality Solvent Flex 2 stop pump with 0.38 mm ID tubing was used in conjunction with the ICP-MS's Peristaltic pump set at 20 RPM (anti-clockwise direction) which resulted in the total Flow to Nebuliser (sample + internal standard both, at 0.25 mL/min) being 0.5 mL/min. The ICP-MS was tuned to high sensitivity settings to facilitate detection of low Pt concentrations in samples. This was done by adjusting the nebuliser gas flow between 0.92-0.94 mL/min to achieve maximum Indium 115 intensity whilst maintaining an acceptable average oxide and doubly charged ratio of 1.64-2.24% and 1.17-1.69% respectively. The analysis method was set to peak hopping mode with a dwell time of 50 milliseconds and 45 sweeps through the mass spectrum per sample read. This setting resulted in a peak integration time of 4500 milliseconds and enabled the collection of numerous data points for peak integration thereby increasing accuracy. The ICP-MS was tuned daily, and its performance optimised to a set of suppliers recommended settings. Analysis was only carried out when optimal oxide and doubly charged ratios with high ionic sensitivity was achieved. All ICP-MS parameters are described in Table S2. The results represent an average of three different experiments run in triplicates (± SEM) and expressed as nmol/10^6 cells or µM/cell. Quantification of the cellular uptake of Pt was based on external standards (Certified Standard from Sigma-Aldrich NSW, Australia) containing Internal standard Ir. The calibration curve is shown in Figure S1.

Table S1. Matrix Conditions selected for RASTRUM bioprinting.

Matrix Code	Formulation	Stiffness (kPa, storage modulus	F-code (Bioink)	F-code (Activator)
Px02.09	GFOGER, RGD	1.1	F236	F177
Px02.31	RGD	1.1	F242	F177
Px02.00	N/A	1.1	F119	F177

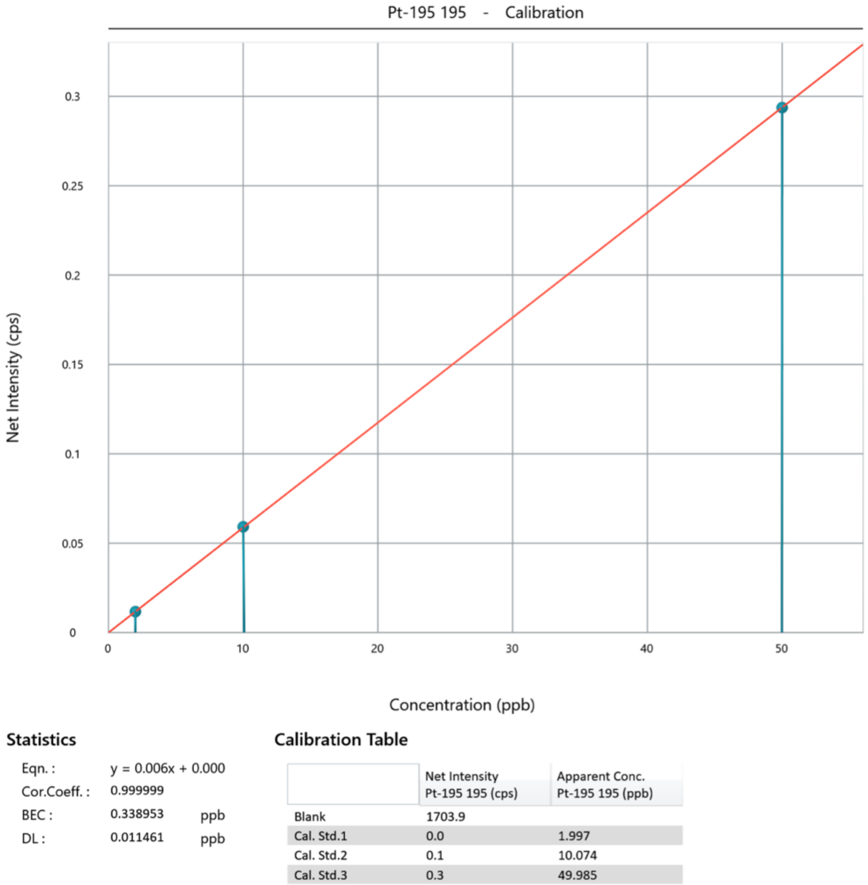

Figure S1. Calibration curve generated by plotting the peak areas measured by the ICP-MS against known concentrations (^{195}Pt(STD)). This curve was used to quantify the cellular uptake of the Pt complexes; y=0.006x + 0.000 and R^2= 0.999999

Table S2. Conditions and parameters selected on the ICP-MS machine.

**Parameter**	**Value**
Plasma RF power	1500 W
Nebulizer gas flow rate	0.92-0.94 $L.min^{-1}$
Auxiliary gas flow rate	1.2 $L.min^{-1}$
Collision gas flow rate (He)	4.5 $L.min^{-1}$
KED Cell Entrance/Exit voltage	-8/-25V
KED CRO/QRO Voltage	-15/-12V
Deflector Voltage	-9V
Isotope monitored	^{195}Pt
Dwell times	50ms
Integration Time	4500ms

RF, radio frequency; He, helium; KED, kinetic energy discrimination

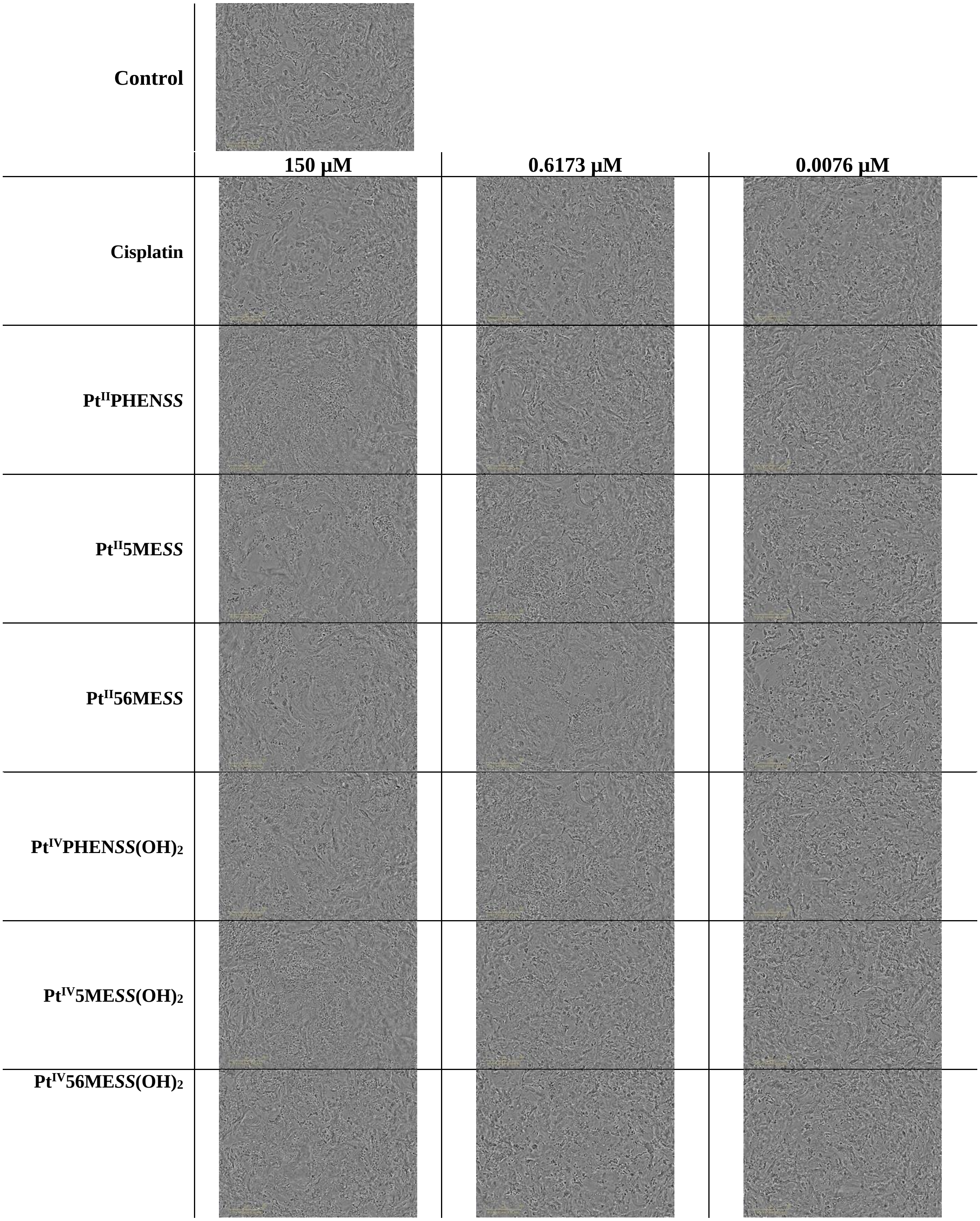

Figure S2. MDA−MB−231 networks. Cell viability upon treatment with platinum(II) (**Pt^{II}PHEN*SS***, **Pt^{II}5ME*SS*** and **Pt^{II}56ME*SS***) and platinum(IV) (**Pt^{IV}PHEN*SS*(OH)$_2$**, **Pt^{IV}5ME*SS*(OH)$_2$** and **Pt^{IV}56ME*SS*(OH)$_2$**) complexes, as well as cisplatin in MDA−MB−231 networks at 72 h. Incucyte® phase contrast microscope used to collect bright-field live images using 10× objective.

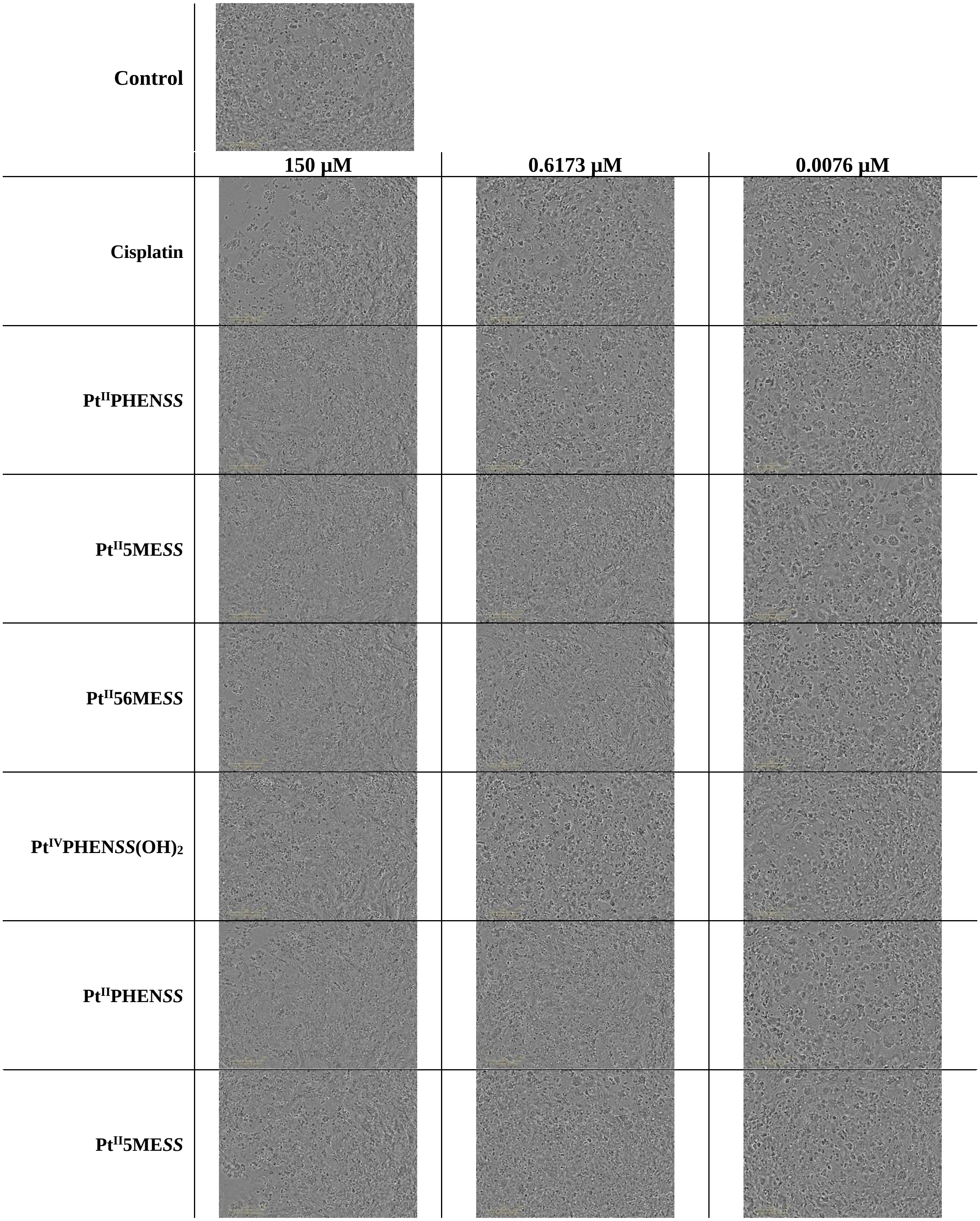

Figure S3. MDA−MB−231 spheroids. Cell viability upon treatment with platinum(II) (**Pt^{II}PHEN*SS***, **Pt^{II}5ME*SS*** and **Pt^{II}56ME*SS***) and platinum(IV) (**Pt^{IV}PHEN*SS*(OH)$_2$**, **Pt^{IV}5ME*SS*(OH)$_2$** and **Pt^{IV}56ME*SS*(OH)$_2$**) complexes, as well as cisplatin in MDA−MB−231 spheroids at 72 h. Incucyte® phase contrast microscope used to collect bright-field live images using 10× objective.

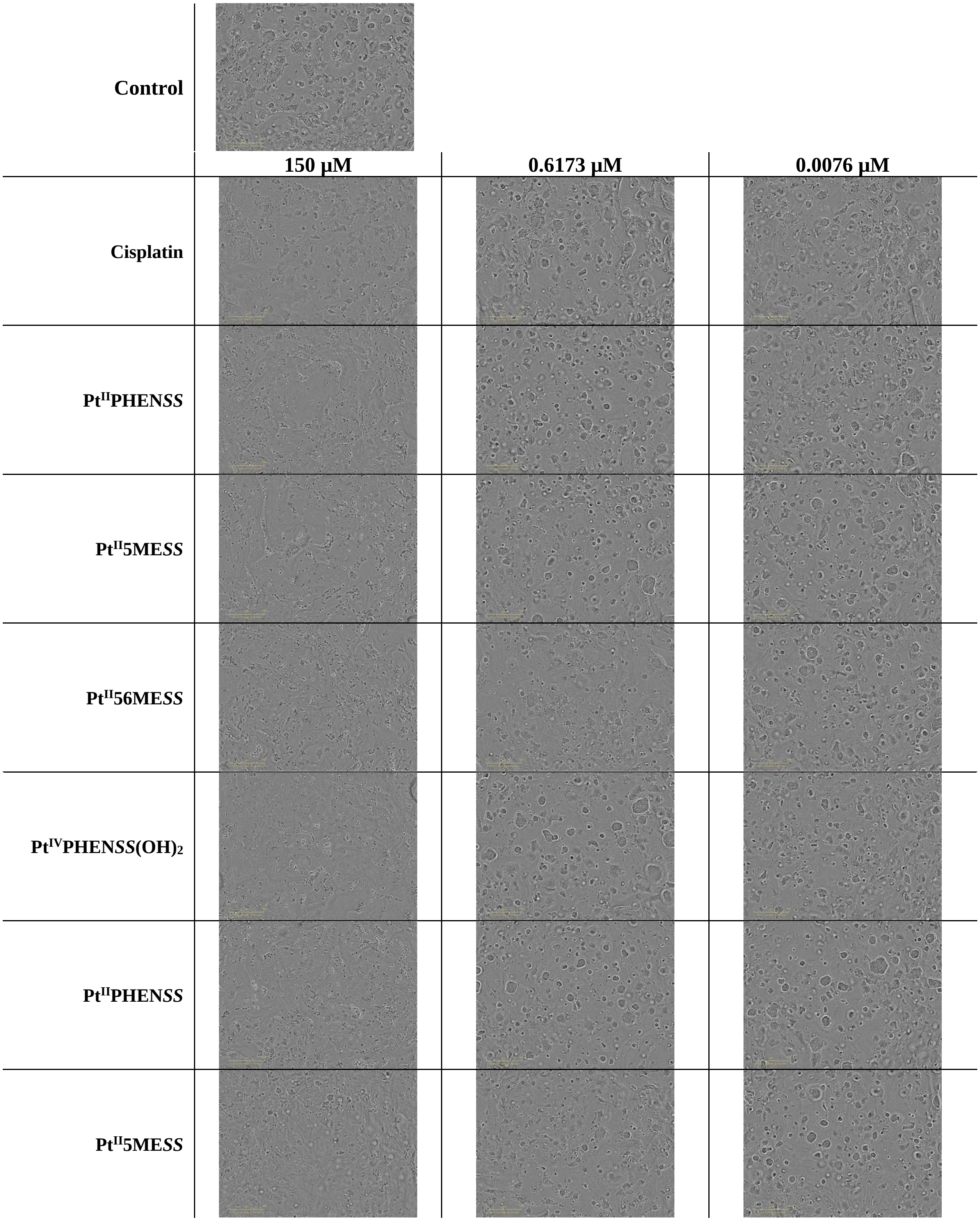

Figure S4. HT29 spheroids. Cell viability upon treatment with platinum(II) (**Pt^{II}PHEN*SS***, **Pt^{II}5ME*SS*** and **Pt^{II}56ME*SS***) and platinum(IV) (**Pt^{IV}PHEN*SS*(OH)$_2$**, **Pt^{IV}5ME*SS*(OH)$_2$** and **Pt^{IV}56ME*SS*(OH)$_2$**) complexes, as well as cisplatin in HT29 spheroids at 72 h. Incucyte® phase contrast microscope used to collect bright-field live images using 10× objective.

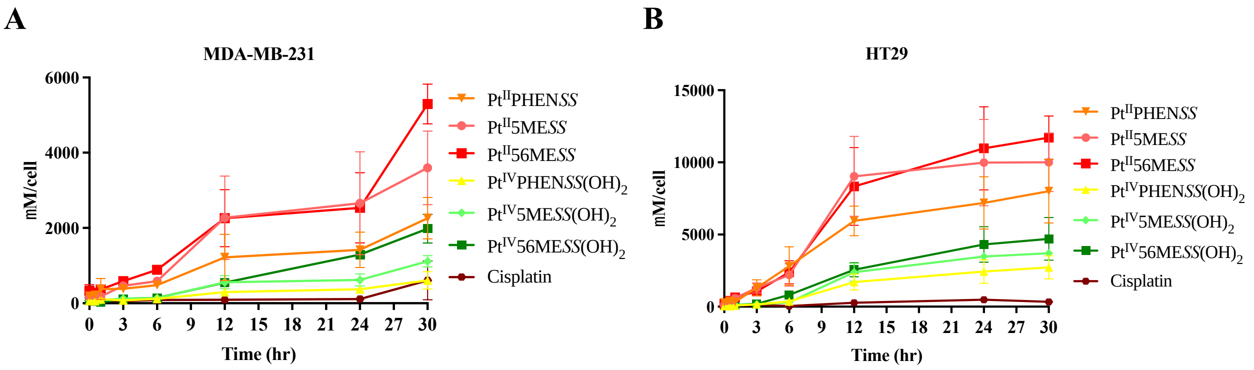

Figure S5. ICP-MS analysis for the uptake of Pt in MDA−MB−231 and HT29 at 0, 0.5, 1, 3, 6, 12, 24 and 30 h. The **Pt^{II}56ME*SS*** precursor has the greatest cellular uptake in both A. MDA−MB−231 and B. HT29. *n* = 3 from three independent experiments where samples were run in triplicates. Data points denote mean ± SEM and expressed in µM/ cell.

Table S3. Kinetic Cellular uptake of Pt complexes by MDA−MB−231. Table shows the average value of cellular concentration in nmol/10^6 cells (top) and the ratio of intracellular concentration to extracellular concentrations (bottom) for each complex. Data points denote mean ± SEM. *n* = 3 from three independent experiments where samples were run in triplicates.

		Time (h)							
	**Complex**	**0**	**0.5**	**1**	**3**	**6**	**12**	**24**	**30**
**Intracellular Concentration (nmol/10^6 cells)**	**Pt^{II}PHENSS**	0.40 ± 0.22	0.46 ± 0.23	0.80 ± 0.59	0.81 ± 0.14	1.03 ± 0.14	2.59 ± 1.29	3.01 ± 0.99	4.79 ± 1.18
	**Pt^{II}5ME*SS***	0.37 ± 0.17	0.44 ± 0.18	0.39 ± 0.05	0.98 ± 0.19	1.25 ± 0.20	4.82 ± 2.35	5.64 ± 2.90	7.63 ± 2.07
	**Pt^{II}56ME*SS***	0.68 ± 0.36	0.58 ± 0.18	0.78 ± 0.04	1.24 ± 0.13	1.88 ± 0.10	4.80 ± 1.61	5.38 ± 1.97	11.24 ± 1.12
	**Pt^{IV}PHENSS(OH)$_2$**	0.13 ± 0.01	0.13 ± 0.03	0.223 ± 0.04	0.16 ± 0.01	0.25 ± 0.06	0.63 ± 0.25	0.78 ± 0.16	1.29 ± 0.49
	**Pt^{IV}5ME*SS*(OH)$_2$**	0.16 ± 0.03	0.22 ± 0.04	0.17 ± 0.07	0.25 ± 0.03	0.29 ± 0.09	1.19 ± 0.37	1.31 ± 0.34	2.37 ± 0.32
	**Pt^{IV}56ME*SS*(OH)$_2$**	0.14 ± 0.04	0.11 ± 0.02	0.05 ± 0.02	0.25 ± 0.04	0.30 ± 0.01	1.16 ± 0.11	2.74 ± 0.07	4.19 ± 0.81
	**Cisplatin**	0.56 ± 0.40	0.15 ± 0.06	0.14 ± 0.05	0.12 ± 0.02	0.18 ± 0.07	0.19 ± 0.01	0.24 ± 0.04	1.29 ± 1.09
**Intracellular/ extracellular ratio**	**Pt^{II}PHEN*SS***	63.45 ± 101.96	72.01 ± 107.27	126.21 ± 279.24	127.55 ± 65.28	161.09 ± 64.41	407.32 ± 612.67	473.33 ± 468.94	752.93 ± 554.54
	**Pt^{II}5ME*SS***	57.65 ± 80.43	69.63 ± 88.74	61.30 ± 24.80	154.59 ± 91.37	196.09 ± 95.27	757.65 ± 1106.77	885.63 ± 1367.55	1199.77 ± 977.40
	**Pt^{II}56ME*SS***	107.16 ± 170.69	92.36 ± 84.69	122.42 ± 23.52	195.59 ± 63.55	296.93 ± 48.82	754.52 ± 758.66	845.27 ± 931.54	1765.95 ± 526.75
	**Pt^{IV}PHEN*SS*(OH)$_2$**	21.21 ± 4.95	20.07 ± 12.01	35.47 ± 19.97	26.14 ± 2.33	38.82 ± 29.09	98.56 ±116.92	123.87 ± 76.45	203.38 ± 233.85
	**Pt^{IV}5ME*SS*(OH)$_2$**	26.14 ± 14.63	35.23 ± 18.90	27.69 ± 34.32	40.45 ± 15.98	46.47 ± 42.96	186.97 ± 177.03	205.97 ± 161.43	371.97 ± 153.15
	**Pt^{IV}56ME*SS*(OH)$_2$**	64.92 ± 18.45	50.71 ± 7.93	26.14 ± 8.97	117.57 ± 16.99	142.93 ± 3.92	548.82 ± 52.42	1293.81 ± 33.95	1978.51 ± 381.47
	**Cisplatin**	88.32 ± 189.97	23.57 ± 29.64	22.36 ± 25.77	19.47 ± 11.33	28.65 ± 30.59	30.78 ± 7.28	38.28 ± 22.01	202.79 ± 518.14

Table S4. Kinetic Cellular uptake of Pt complexes by HT29. Table shows the average value of cellular concentration in nmol/10^6 cells (top) and the ratio of intracellular concentration to extracellular concentrations (bottom) for each complex. Data points denote mean ± SEM. *n* = 3 from three independent experiments where samples were run in triplicates.

	Complex	Time (h)							
		**0**	**0.5**	**1**	**3**	**6**	**12**	**24**	**30**
**Intracellular Concentration (nmol/10^6 cells)**	**Pt^{II}PHEN*SS***	0.61 ± 0.33	0.85 ± 0.25	0.72 ± 0.23	2.77 ± 1.16	5.95 ± 2.84	12.59 ± 2.18	15.26 ± 3.85	16.99 ± 4.69
	**Pt^{II}5ME*SS***	0.28 ± 0.06	0.79 ± 0.50	1.26 ± 0.22	2.75 ± 0.74	4.65 ± 1.64	19.18 ± 5.88	21.18 ± 6.35	21.24 ± 4.31
	**Pt^{II}56ME*SS***	0.45 ± 0.02	0.87 ± 0.27	1.38 ± 0.19	2.23 ± 0.45	5.05 ± 1.70	17.67 ± 5.72	23.28 ± 6.12	24.85 ± 3.20
	**Pt^{IV}PHEN*SS*(OH)$_2$**	0.10 ± 0.02	0.10 ± 0.01	0.18 ± 0.02	0.33 ± 0.14	0.77 ± 0.53	3.64 ± 1.18	5.18 ± 1.73	5.79 ± 1.72
	**Pt^{IV}5ME*SS*(OH)$_2$**	0.15 ± 0.02	0.17 ± 0.02	0.28 ± 0.05	0.33 ± 0.12	0.66 ± 0.32	5.05 ± 1.32	7.37 ± 0.10	7.85 ± 0.55
	**Pt^{IV}56ME*SS*(OH)$_2$**	0.14 ± 0.01	0.18 ± 0.01	0.28 ± 0.01	0.42 ± 0.03	1.74 ± 0.17	5.43 ± 1.04	9.14 ± 2.61	9.98 ± 3.14
	**Cisplatin**	0.36 ± 0.04	0.94 ± 0.55	0.14 ± 0.02	0.28 ± 0.09	0.10 ± 0.01	0.57 ± 0.23	1.02 ± 0.57	0.71 ± 0.18
**Intracellular/ extracellular ratio**	**Pt^{II}PHEN*SS***	95.39 ±157.34	133.55 ±119.40	112.44 ± 110.62	435.59 ± 544.47	935.29 ±1340.57	1979.17 ±1025.61	2397.59 ± 1814.7	26698.41 ±2208.68
	**Pt^{II}5ME*SS***	44.37 ± 26.94	125.00 ±236.39	197.63± 102.35	431.44 ± 348.12	730.89 ± 774.95	3013.21 ±2773.67	3327.94 ±2994.12	3337.20 ±2030.29
	**Pt^{II}56ME*SS***	70.93 ± 9.69	136.74 ±131.83	216.82 ± 89.98	350.73 ± 213.21	793.80 ± 803.03	2776.28 ±2694.76	3657.59 ±2886.20	3903.51 ±1509.01
	**Pt^{IV}PHEN*SS*(OH)$_2$**	16.45 ± 8.04	15.93 ± 3.81	28.08 ± 8.54	51.34 ± 67.56	121.19 ± 249.85	571.26 ± 554.55	813.09 ± 817.45	909.81 ± 808.35
	**Pt^{IV}5ME*SS*(OH)$_2$**	25.03 ± 9.01	26.29 ± 8.01	44.59 ± 25.24	52.25 ± 56.49	104.08 ± 151.75	793.61 ± 622.21	1158.37 ± 47.58	1234.08 ± 258.11
	**Pt^{IV}56ME*SS*(OH)$_2$**	21.81 ± 4.03	28.52 ± 6.13	43.51 ± 3.21	66.77 ± 12.84	273.54 ± 78.68	854.01 ± 488.39	1435.74 ±1229.28	1568.28 ±1478.83
	**Cisplatin**	57.31 ± 20.91	147.74 ±258.72	22.65 ± 9.82	43.31 ± 46.90	16.07 ± 6.78	89.42 ± 106.10	160.54 ± 271.72	112.14 ± 83.99

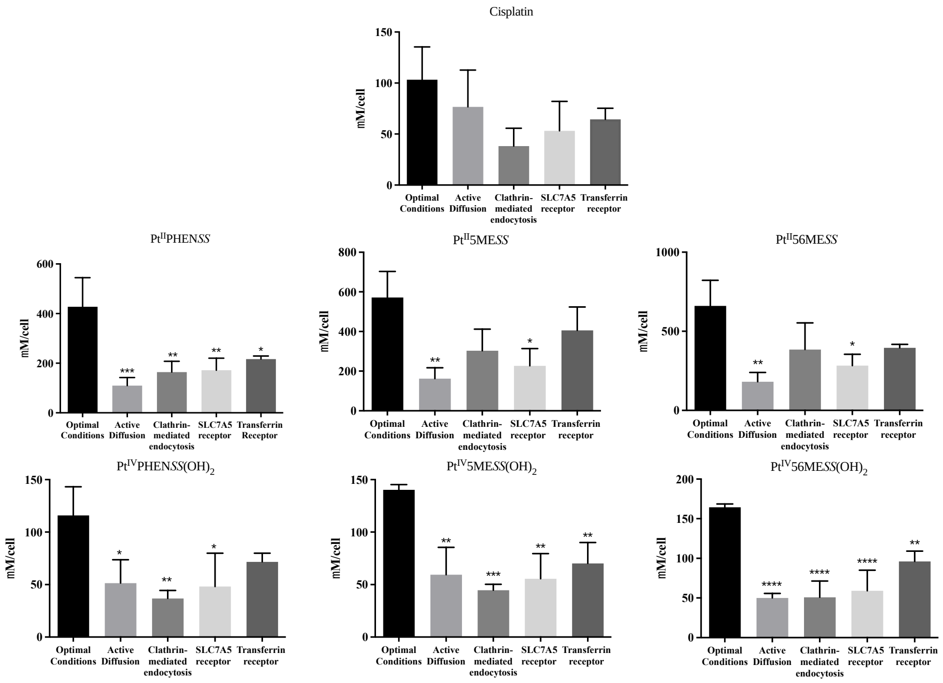

Figure S6. Mode of uptake of Platinum in MDA−MB−231. The intracellular amount of Pt was measured by ICP-MS after incubation at 37 °C or 4 °C, as well as following inhibition of the SLC7A5, transferrin receptor or clathrin-mediated endocytosis. Data denote mean ± SEM of three independent experiments where samples were run in triplicates. **p* < 0.05, ***p* < 0.01, ****p* < 0.001 and *****p* < 0.0001 in comparison to the optimal conditions.

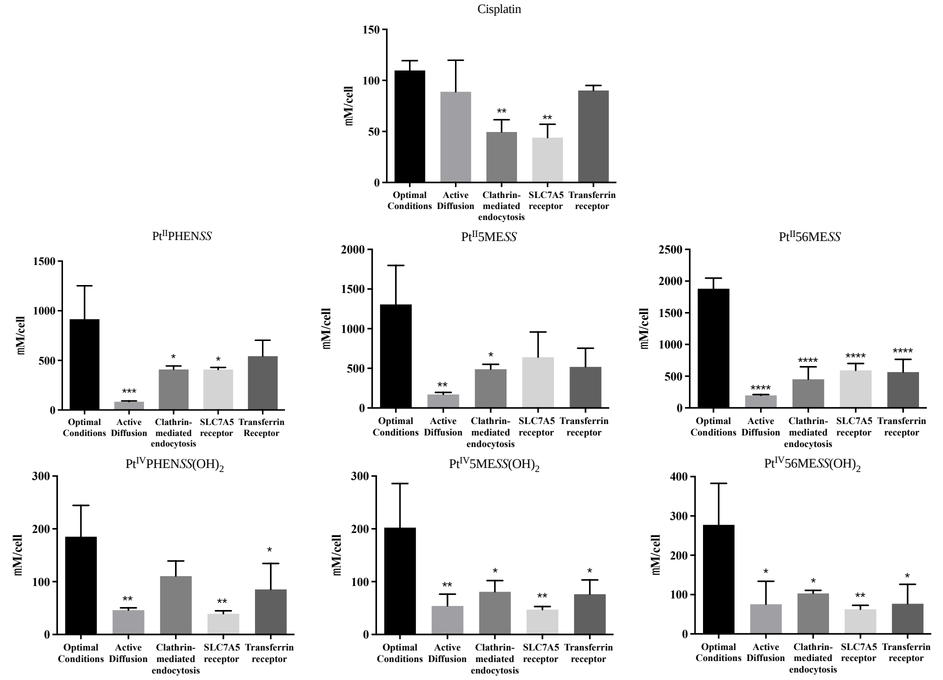

Figure S7. Mode of uptake of Platinum in HT29. The intracellular amount of Pt was measured by ICP-MS after incubation at 37 °C or 4 °C, as well as following inhibition of the SLC7A5, transferrin receptor or clathrin-mediated endocytosis. Data denote mean ± SEM of three independent experiments where samples were run in triplicates. **p* < 0.05, ***p* < 0.01, ****p* < 0.001 and *****p* < 0.0001 in comparison to the optimal conditions.

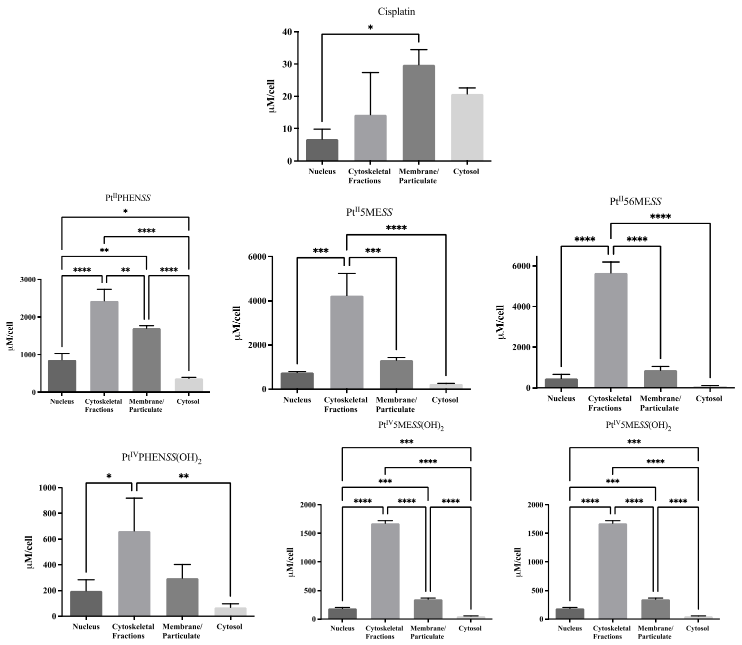

Figure S8. Cellular Localisation of Platinum in MDA−MB−231. The intracellular amount of Pt (µM/cell) was measured by ICP-MS after cellular fractionation. Data denote mean ± SEM of three independent experiments where samples were run in triplicates. *$p < 0.05$, **$p < 0.01$, ***$p < 0.001$ and ****$p < 0.0001$ in comparison to the fractions.

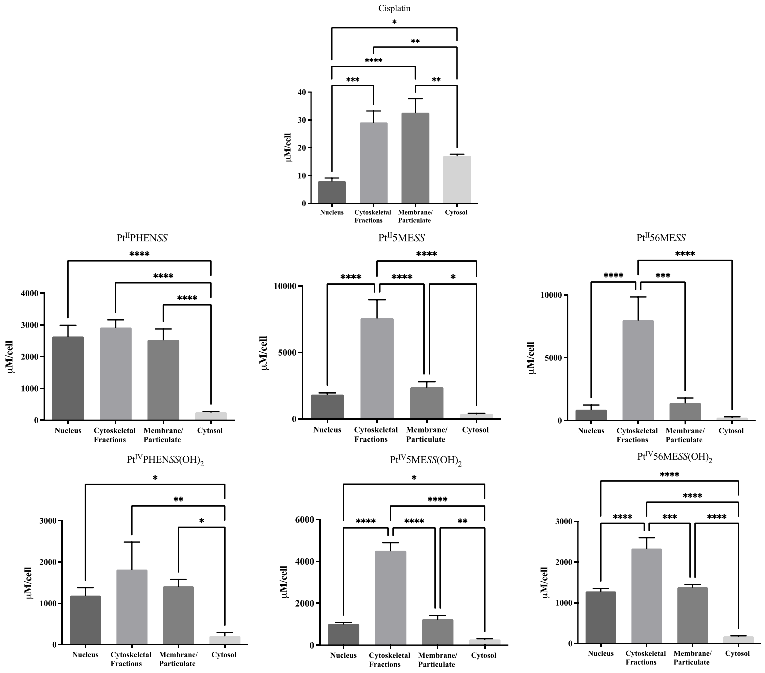

Figure S9. Cellular Localisation of Platinum in HT29. The intracellular amount of Pt (µM/cell) was measured by ICP-MS after cellular fractionation. Data denote mean ± SEM of three independent experiments where samples were run in triplicates. *$p < 0.05$, **$p < 0.01$, ***$p < 0.001$ and ****$p < 0.0001$ in comparison to the fractions.

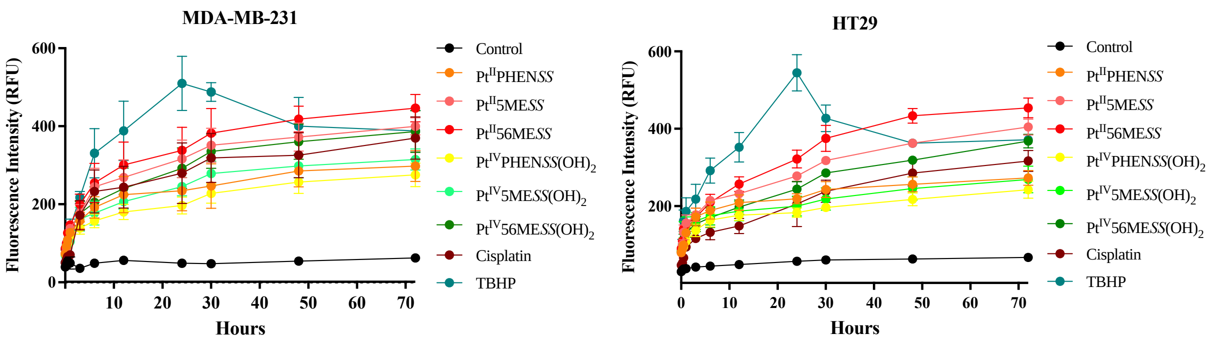

Figure. S10. ROS production upon treatment with platinum(II) and (IV) complexes in MDA−MB−231 and HT29 at 0, 0.25, 0.5, 1, 3, 6, 12, 24, 48 and 72 h. Pt^{II}PHEN*SS*, Pt^{II}5ME*SS*, Pt^{II}56ME*SS*, Pt^{IV}PHEN*SS*(OH)$_2$, Pt^{IV}5ME*SS*(OH)$_2$, Pt^{IV}56ME*SS*(OH)$_2$, cisplatin and TBHP: t-butyl hydroperoxide. Data points denote mean ± SEM. *n* = 3 from three independent experiments where samples were run in triplicate.

Table S5. ROS production upon treatment with complexes Pt^{II}PHEN*SS*, Pt^{II}5ME*SS*, Pt^{II}56ME*SS*, Pt^{IV}PHEN*SS*(OH)$_2$, Pt^{IV}5ME*SS*(OH)$_2$, Pt^{IV}56ME*SS*(OH)$_2$, Cisplatin and TBHP: t-butyl hydroperoxide, in MDA−MB−231 and HT29 cells at 24, 48 and 72 h.

Complex	ROS production in different time intervals (RFU)					
	MDA−MB−231			HT29		
	24 h	48 h	72 h	24 h	48 h	72 h
Control	48.77 ± 1.16	54.55 ± 1.14	62.33 ± 0.69	57.22 ± 0.34	63.22 ± 0.30	67.33 ± 0.21
Cisplatin	279.82 ± 14.88	325.96 ± 11.09	369.97 ± 10.43	205.39 ± 11.26	285.11 ± 6.49	316.55 ± 5.21
TBHP	509.88 ± 13.38	400.11 ± 14.21	388.41 ± 10.04	544.67 ± 9.04	362.55 ± 1.84	371.44 ± 1.74
Pt^{II}PHEN*SS*	234.82 ± 9.88	285.31 ± 7.84	297.81 ± 7.62	219.00 ± 2.52	256.22 ± 3.67	273.00 ± 3.71
Pt^{II}5ME*SS*	316.23 ± 9.22	372.49 ± 6.94	399.97 ± 4.28	278.00 ± 2.18	362.89 ± 0.88	404.44 ± 3.93
Pt^{II}56ME*SS*	338.49 ± 11.45	418.08 ± 6.35	446.09 ± 6.81	321.56 ± 4.44	433.67 ± 3.65	453.89 ± 4.91
Pt^{IV}PHEN*SS*(OH)$_2$	196.62 ± 4.17	256.83 ± 5.57	275.09 ± 5.81	182.77 ± 2.29	217.33 ± 3.18	242.44 ± 4.28
Pt^{IV}5ME*SS*(OH)$_2$	245.26 ± 4.55	264.00 ± 5.91	314.78 ± 5.21	234.55 ± 3.09	278.33 ± 4.39	296.67 ± 4.85
Pt^{IV}56ME*SS*(OH)$_2$	292.21 ± 10.00	360.12 ± 8.69	386.28 ± 10.27	244 ± 3.67	318.56 ± 1.15	368.11 ± 3.38

Table S6. Mitochondrial membrane potential upon treatment with platinum(II) (Pt^{II}PHEN*SS*, Pt^{II}5ME*SS*, Pt^{II}56ME*SS*) and platinum (IV) (Pt^{IV}PHEN*SS*(OH)$_2$, Pt^{IV}5ME*SS*(OH)$_2$, Pt^{IV}56ME*SS*(OH)$_2$,) complexes, and cisplatin in MDA−MB−231 and HT29 cells at 24, 48 and 72 h.

Complex	MtMp in different time intervals (RFU)					
	MDA−MB−231			HT29		
	24 h	48 h	72 h	24 h	48 h	72 h
Control	411.10 ± 13.62	368.86 ± 19.21	367.15 ± 12.30	471.52 ± 19.62	398.14 ± 15.77	409.06 ± 4.52
Cisplatin	208.24 ± 11.41	177.91 ± 11.37	154.83 ± 15.32	270.02 ± 10.56	237.68 ± 4.61	188.59 ± 10.43
FCCP	215.89 ± 6.12	123.38 ± 13.15	99.10 ± 8.58	184.32 ± 9.51	143.31 ± 4.61	83.58 ± 3.54
Pt^{II}PHEN*SS*	258.17 ± 8.25	218.19 ± 16.07	169.95 ± 14.30	321.85 ± 21.92	256.63 ± 8.58	236.73 ± 10.29
Pt^{II}5ME*SS*	230.62 ± 12.03	189.85 ± 7.61	150.71 ± 14.76	253.81 ± 6.85	211.88 ± 6.22	143.93 ± 5.49
Pt^{II}56ME*SS*	155.45 ± 21.62	131.53 ± 18.06	86.51 ± 2.83	159.93 ± 9.67	151.44 ± 13.23	102.76 ± 7.13
Pt^{IV}PHEN*SS*(OH)$_2$	297.56 ± 7.26	257.95 ± 17.97	179.38 ± 16.49	312.19 ± 14.04	261.95 ± 4.88	215.58 ± 7.74
Pt^{IV}5ME*SS*(OH)$_2$	250.80 ± 11.01	201.07 ± 11.03	155.85 ± 10.56	304.45 ± 1.60	224.23 ± 8.71	198.24 ± 3.77
Pt^{IV}56ME*SS*(OH)$_2$	190.39 ± 20.15	156.37 ± 16.19	92.94 ± 5.17	258.69 ± 15.37	172.39 ± 5.78	124.84 ± 12.34

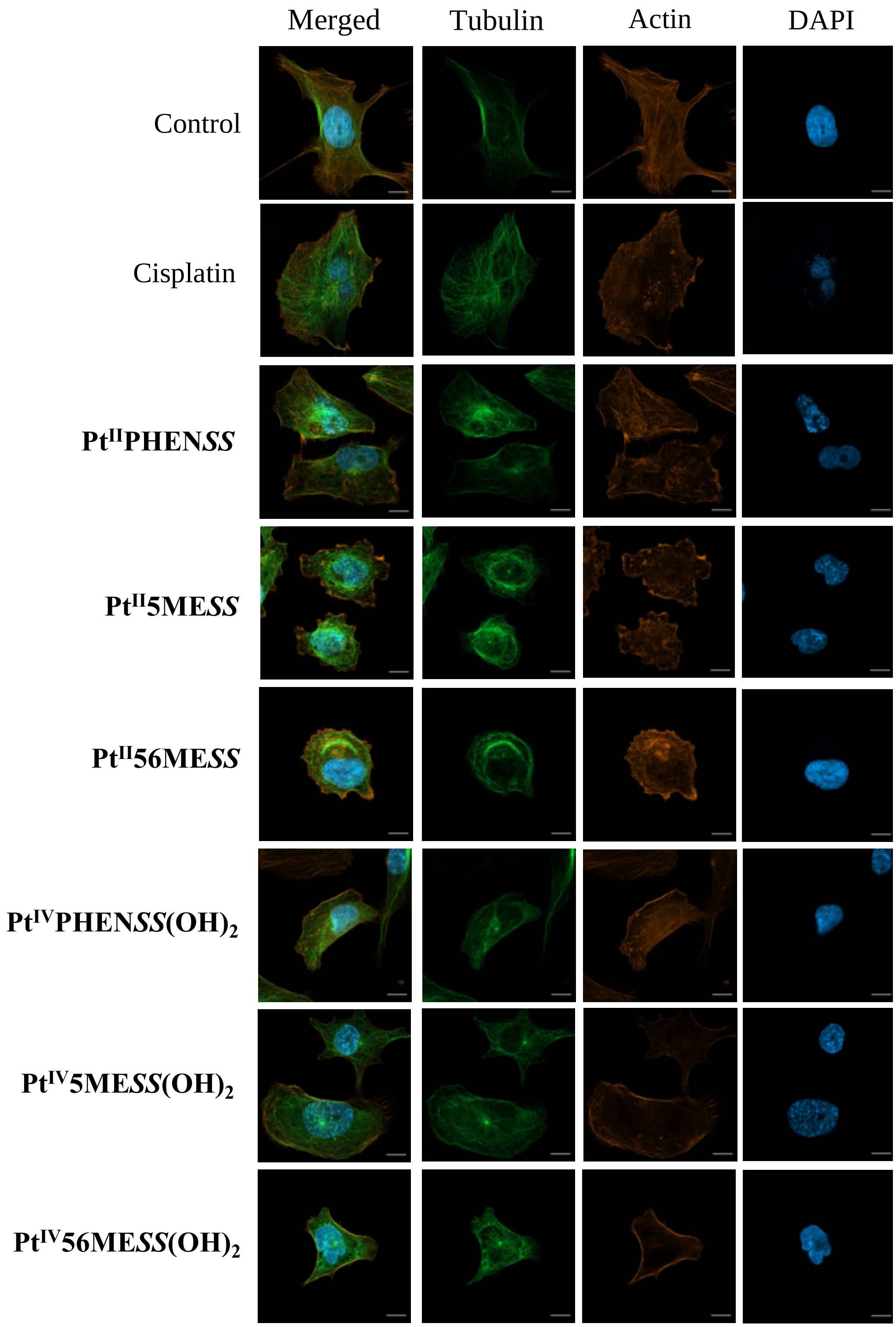

Figure S11. Effect of platinum complexes and cisplatin on β-tubulin and F-actin. Immunofluorescence upon treatment with platinum(II) and platinum(IV) complexes, as well as cisplatin in MDA−MB−231. Airyscan images were collected at 63×. Confocal microscope paramters were constant across all treatments for comparison in expression. *n* = 50 cells.

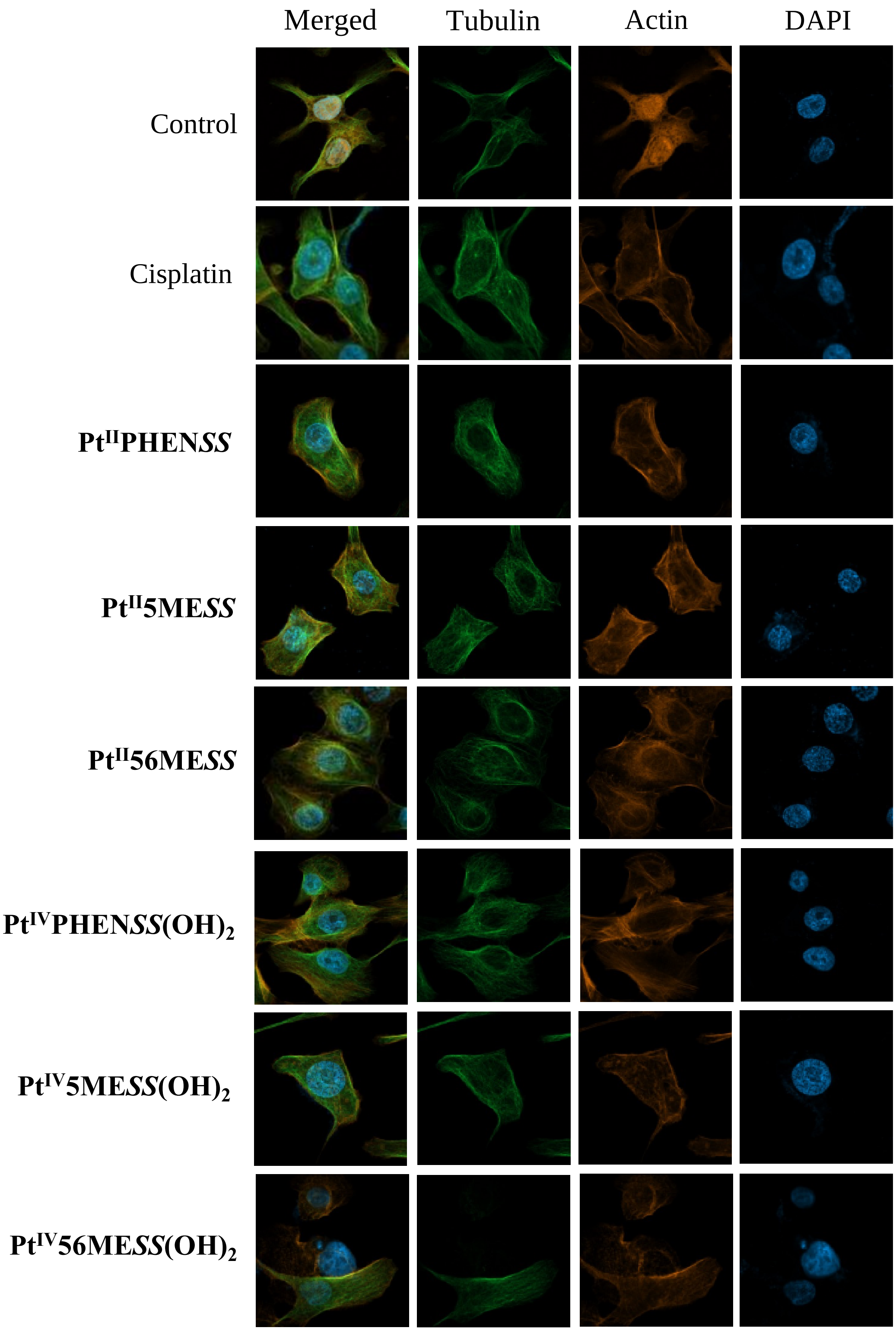

Figure S12. Effect of platinum complexes and cisplatin on β-tubulin and F-actin. Immunofluorescence upon treatment with platinum(II) and platinum(IV) complexes, as well as cisplatin in MCF10A. Airyscan images were collected at 63×. Confocal microscope paramters were constant across all treatments for comparison in expression. *n* = 50 cells.

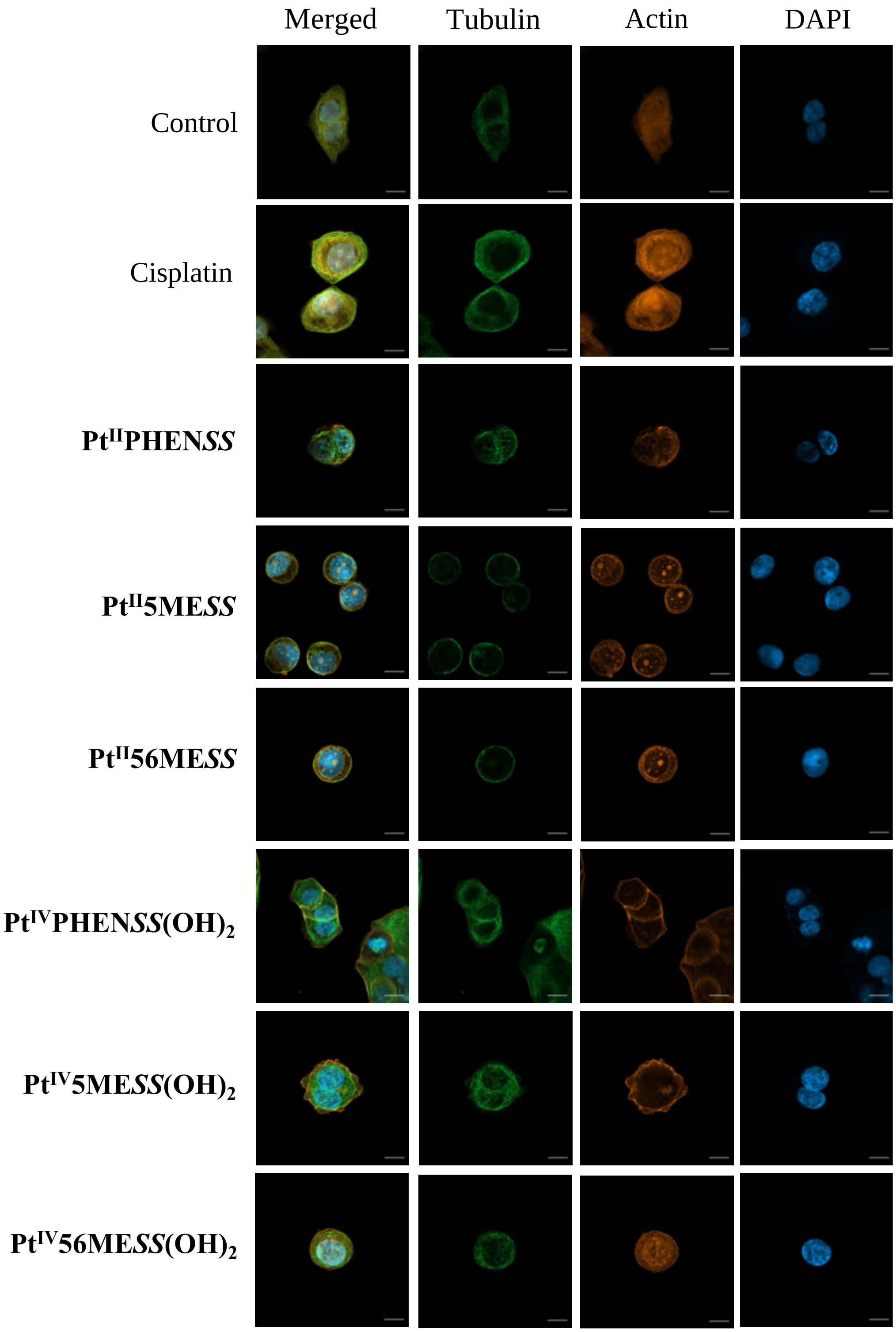

Figure S13. Effect of platinum complexes and cisplatin on β-tubulin and F-actin. Immunofluorescence upon treatment with platinum(II) and platinum(IV) complexes, as well as cisplatin in HT29. Airyscan images were collected at 63×. Confocal microscope paramters were constant across all treatments for comparison in expression. $n = 50$ cells.

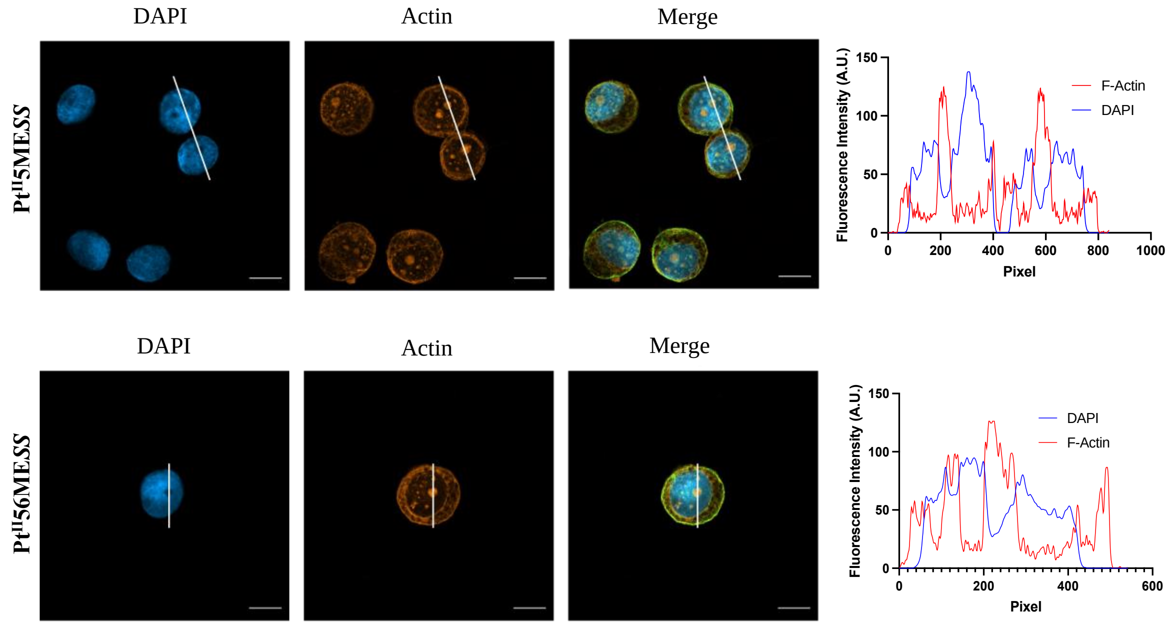

Figure S14. Relation between phalloidin and DAPI after Pt^{II}5ME*SS* and Pt^{II}56ME*SS* treatment. Immunofluorescence upon treatment with platinum(II) in HT29. Airyscan images were collected at 63×. Fluorescent Intensity profile of Actin (red) and DAPI (blue) over line selection (white).

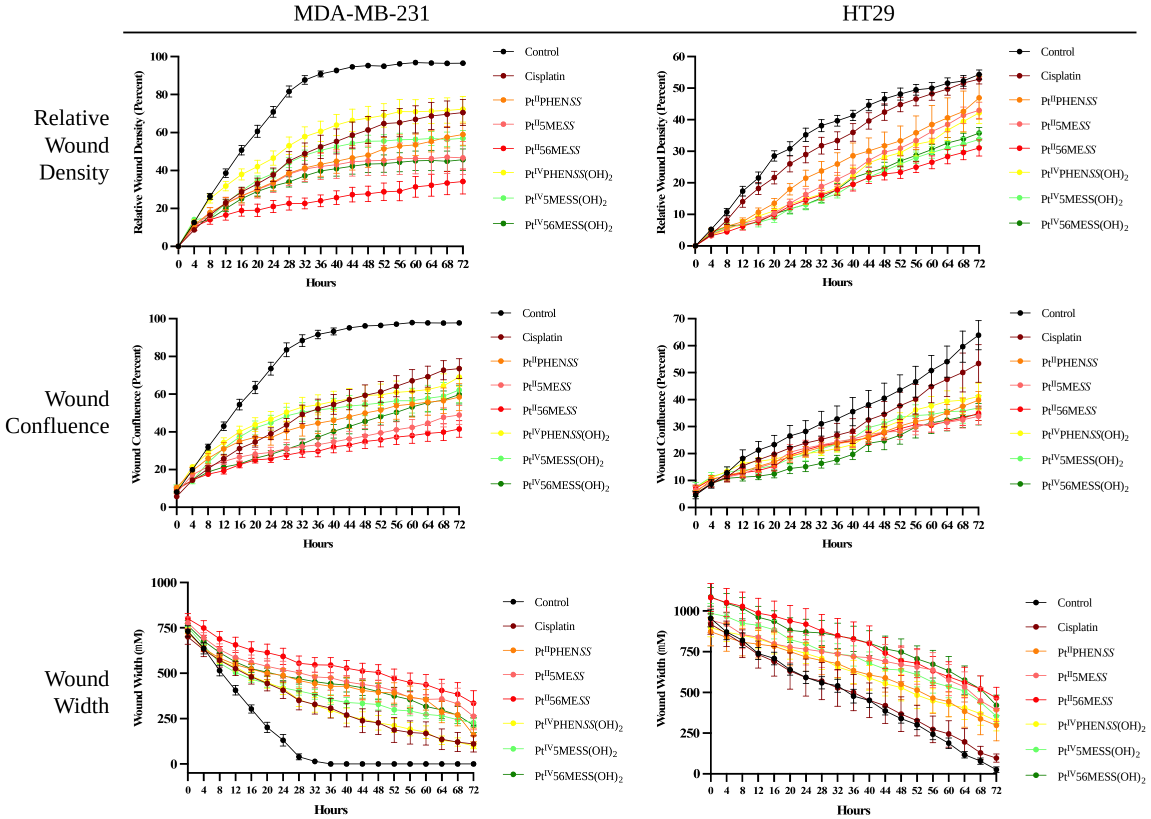

Figure S15. Cell migration (scratch wound healing assay). Cell Migration upon treatment with platinum(II) (**Pt^{II}PHEN*SS***, **Pt^{II}5ME*SS*** and **Pt^{II}56ME*SS***) and platinum(IV) (**Pt^{IV}PHEN*SS*(OH)$_2$**, **Pt^{IV}5ME*SS*(OH)$_2$** and **Pt^{IV}56ME*SS*(OH)$_2$**) complexes, as well as cisplatin in MDA−MB−231 and HT29 cells quantified every 4 h for up to 72 h.

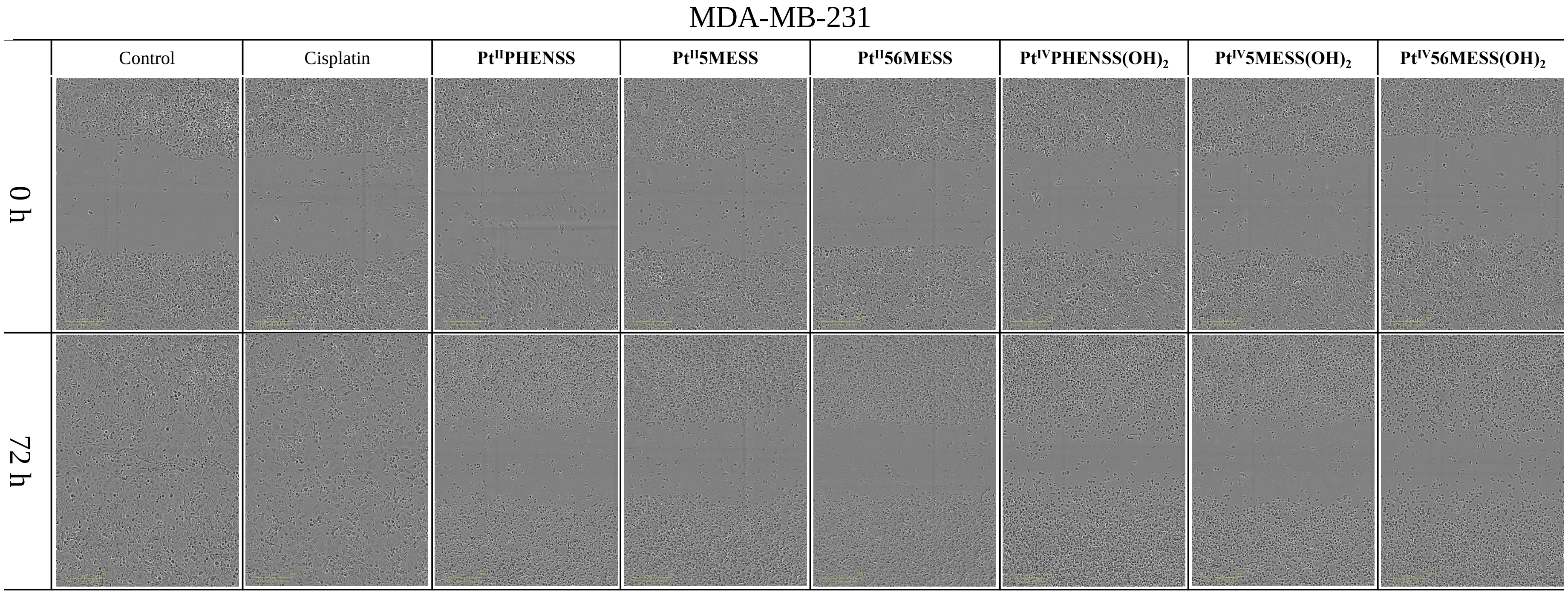

Figure S16. Cell migration. Cell Migration upon treatment with platinum(II) (**Pt^{II}PHEN*SS***, **Pt^{II}5ME*SS*** and **Pt^{II}56ME*SS***) and platinum(IV) (**Pt^{IV}PHEN*SS*(OH)$_2$**, **Pt^{IV}5ME*SS*(OH)$_2$** and **Pt^{IV}56ME*SS*(OH)$_2$**) complexes, as well as cisplatin in MDA−MB−231 cells at 0 and 72 h. Incucyte® phase contrast microscope used to collect bright-field live images using 10× objective.

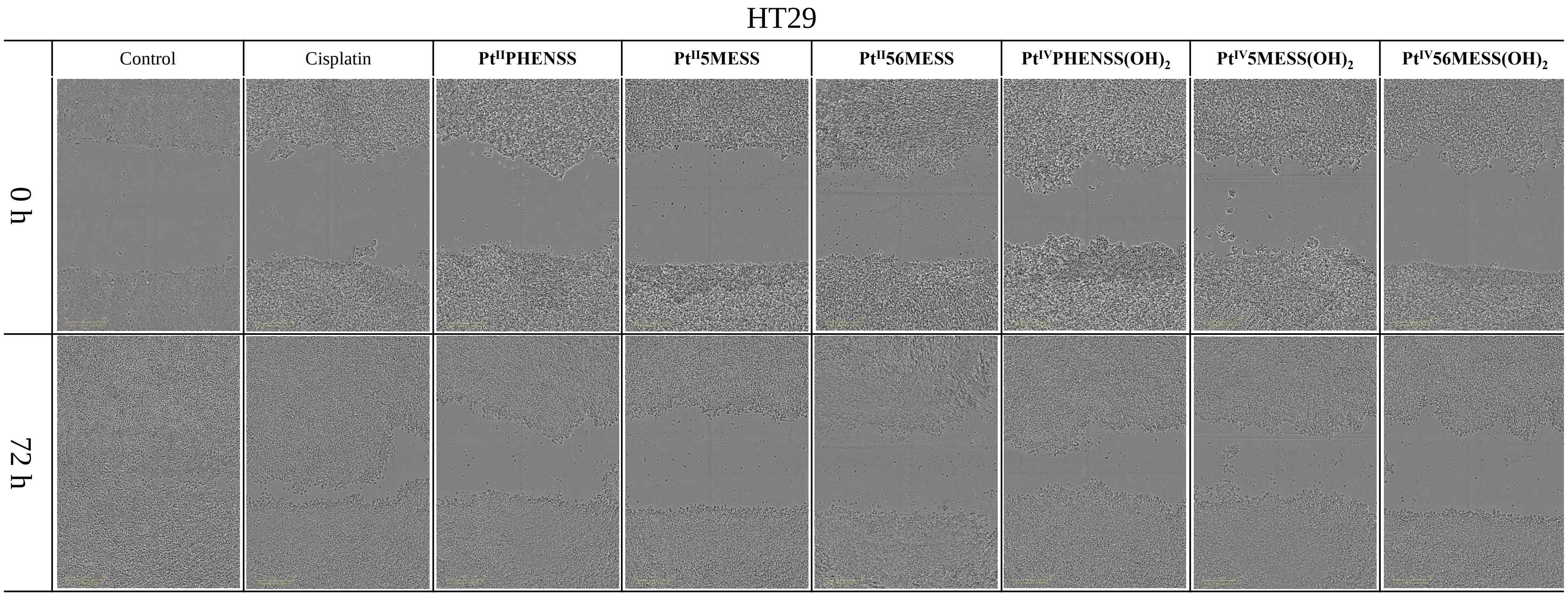

Figure S17. Cell migration. Cell Migration upon treatment with platinum(II) (**Pt^{II}PHEN*SS***, **Pt^{II}5ME*SS*** and **Pt^{II}56ME*SS***) and platinum(IV) (**Pt^{IV}PHEN*SS*(OH)$_2$**, **Pt^{IV}5ME*SS*(OH)$_2$** and **Pt^{IV}56ME*SS*(OH)$_2$**) complexes, as well as cisplatin in HT29 cells at 0 and 72 h. Incucyte® phase contrast microscope used to collect bright-field live images using 10× objective.

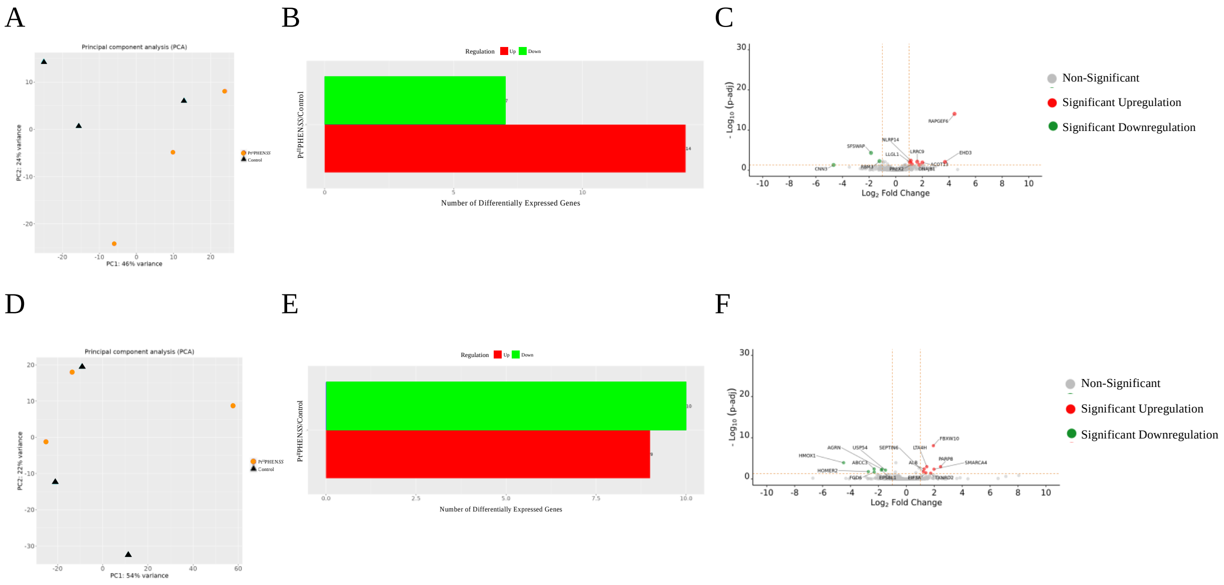

Figure S18. Proteomic analysis of MDA−MB−231 and HT29 upon treatment with Pt^{II}PHEN*SS*. A. MDA−MB−231 principal component analysis. B. Number of differentially expressed proteins (DEPs) in MDA−MB−231. C. Volcano plot of DEPs upregulated (red) and down regulated (green) proteins in MDA−MB−231. D. HT29 principal component analysis. E. Number of differentially expressed proteins (DEPs) in HT29. F. Volcano plot of DEPs upregulated (red) and down regulated (green) proteins in HT29.

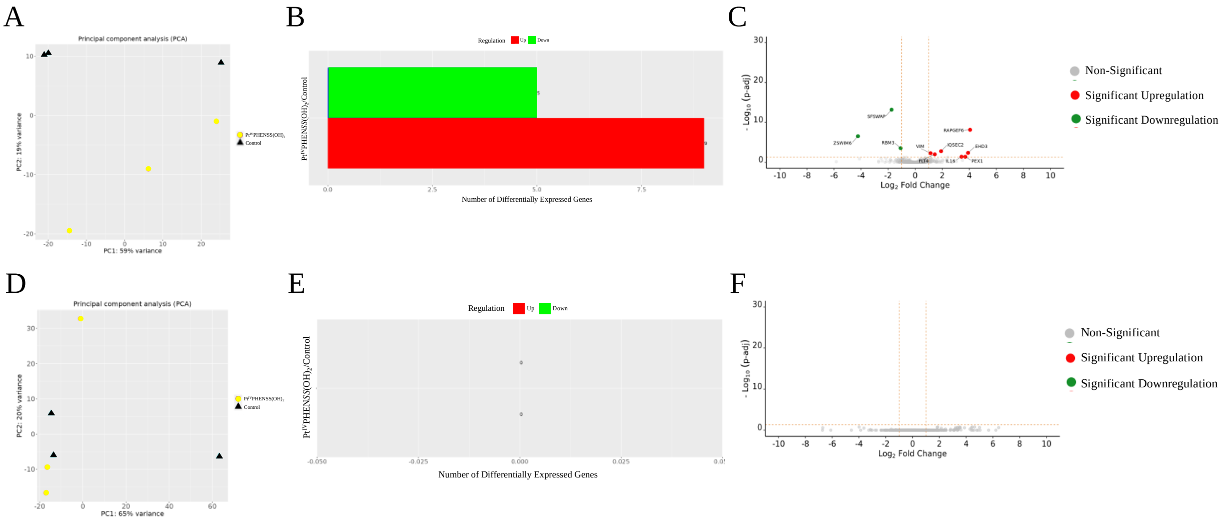

Figure S19. Proteomic analysis of MDA−MB−231 and HT29 upon treatment with Pt^{IV}PHEN*SS*$(OH)_2$. A. MDA−MB−231 principal component analysis. B. Number of differentially expressed proteins (DEPs) in MDA−MB−231. C. Volcano plot of DEPs upregulated (red) and down regulated (green) proteins in MDA−MB−231. D. HT29 principal component analysis. E. Number of differentially expressed proteins (DEPs) in HT29. F. Volcano plot of DEPs upregulated (red) and down regulated (green) proteins in HT29.

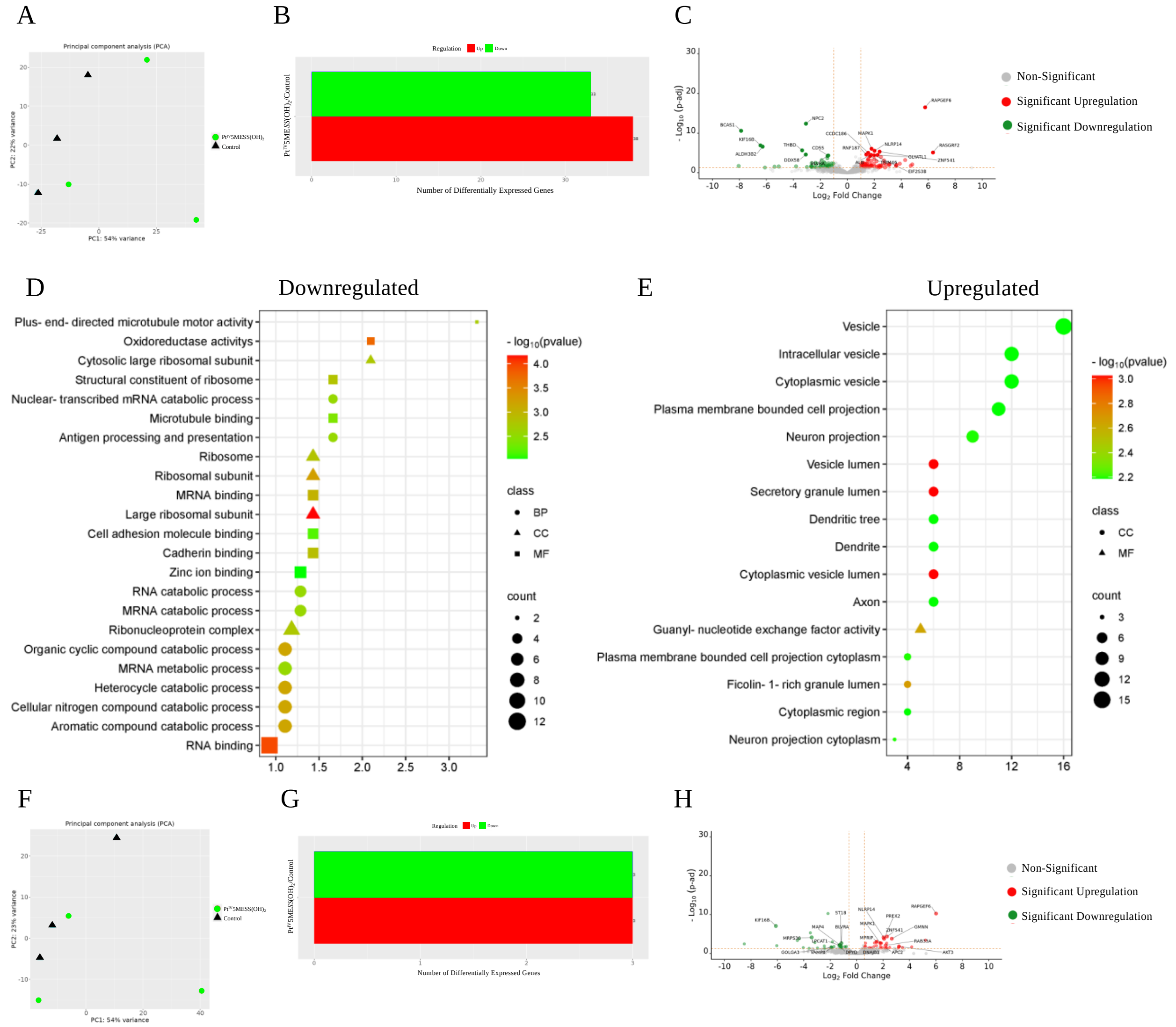

Figure S20. Proteomic analysis of MDA−MB−231 and HT29 upon treatment with Pt^{IV}5MES*SS*$(OH)_2$. A. MDA−MB−231 principal component analysis. B. Number of differentially expressed proteins (DEPs) in MDA−MB−231. C. Volcano plot of DEPs upregulated (red) and down regulated (green) proteins in MDA−MB−231. MDA−MB−231 GO enriched biological processes, cellular components, and molecular function in MDA−MB−231, D. downregulated and E. upregulated proteins. F. HT29 principal component analysis. G. Number of differentially expressed proteins (DEPs) in HT29. H. Volcano plot of DEPs upregulated (red) and down regulated (green) proteins in HT29.

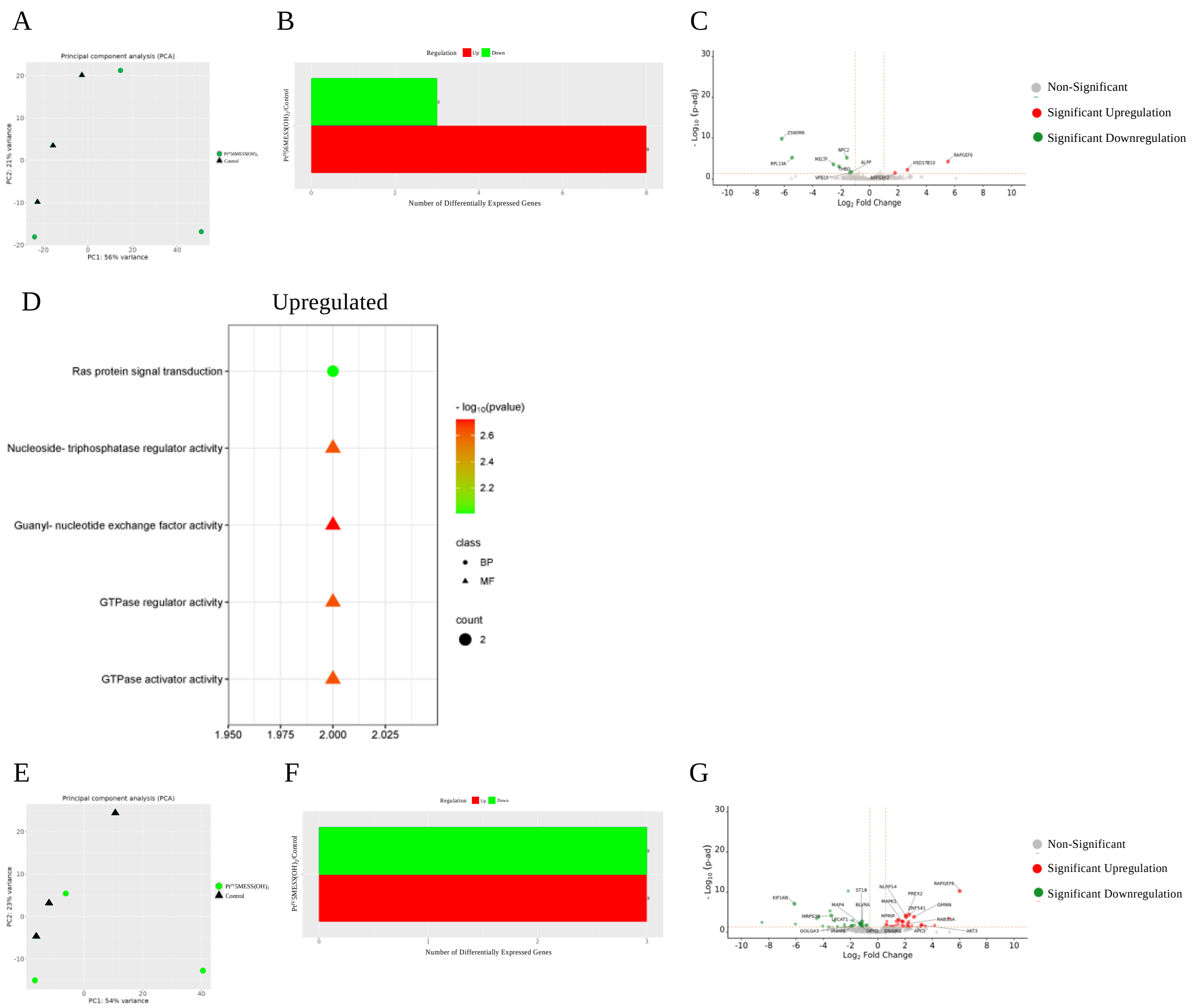

Figure S21. Proteomic analysis of MDA−MB−231 and HT29 upon treatment with Pt^{IV}56MES*S*(OH)$_2$. A. MDA−MB−231 principal component analysis. B. Number of differentially expressed proteins (DEPs) in MDA−MB−231. C. Volcano plot of DEPs upregulated (red) and down regulated (green) proteins in MDA−MB−231. D. MDA−MB−231 GO enriched biological processes, cellular components, and molecular function in upregulated proteins. E. HT29 principal component analysis. F. Number of differentially expressed proteins (DEPs) in HT29. G. Volcano plot of DEPs upregulated (red) and down regulated (green) proteins in HT29.

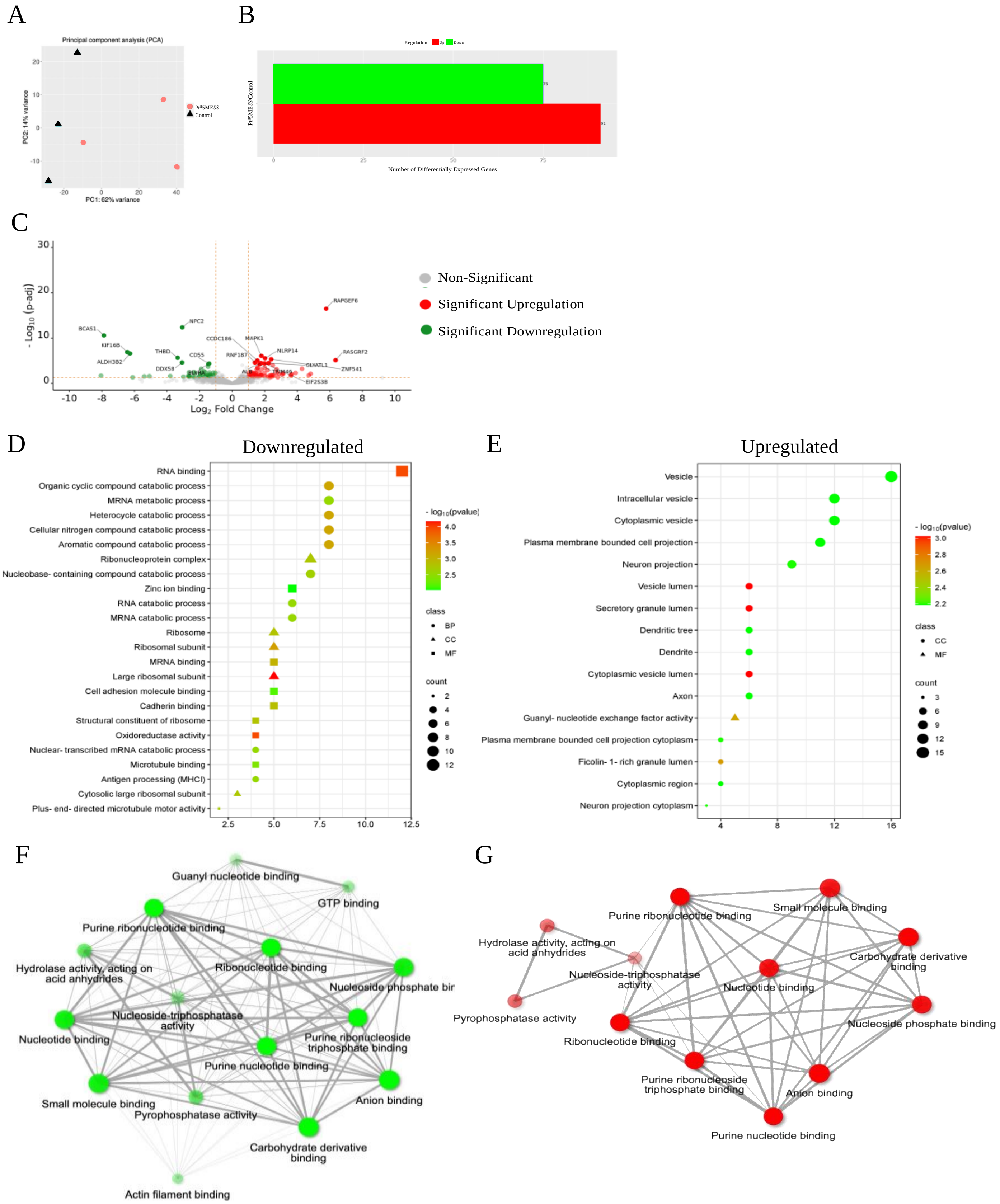
A
Principal component analysis (PCA)
PC1: 62% variance
PC2: 14% variance
Control
B
Regulation
Up
Down
Number of Differentially Expressed Genes
C
Non-Significant
Significant Upregulation
Significant Downregulation
Log2 Fold Change
- Log10 (p-adj)
RAPGEF6
NPC2
BCAS1
KIF16B
ALDH3B2
THBD
CD55
DDX58
CCDC186
MAPK1
RNF187
NLRP14
RASGRF2
GLYATL1
ZNF541
EIF2S3B
D
Downregulated
RNA binding
Organic cyclic compound catabolic process
MRNA metabolic process
Heterocycle catabolic process
Cellular nitrogen compound catabolic process
Aromatic compound catabolic process
Ribonucleoprotein complex
Nucleobase- containing compound catabolic process
Zinc ion binding
RNA catabolic process
MRNA catabolic process
Ribosome
Ribosomal subunit
MRNA binding
Large ribosomal subunit
Cell adhesion molecule binding
Cadherin binding
Structural constituent of ribosome
Oxidoreductase activity
Nuclear- transcribed mRNA catabolic process
Microtubule binding
Antigen processing (MHCI)
Cytosolic large ribosomal subunit
Plus- end- directed microtubule motor activity
class
BP
CC
MF
count
E
Upregulated
Vesicle
Intracellular vesicle
Cytoplasmic vesicle
Plasma membrane bounded cell projection
Neuron projection
Vesicle lumen
Secretory granule lumen
Dendritic tree
Dendrite
Cytoplasmic vesicle lumen
Axon
Guanyl- nucleotide exchange factor activity
Plasma membrane bounded cell projection cytoplasm
Ficolin- 1- rich granule lumen
Cytoplasmic region
Neuron projection cytoplasm
class
CC
MF
count
F
Guanyl nucleotide binding
GTP binding
Purine ribonucleotide binding
Hydrolase activity, acting on acid anhydrides
Ribonucleotide binding
Nucleoside phosphate bir
Nucleoside-triphosphatase activity
Nucleotide binding
Purine ribonucleoside triphosphate binding
Purine nucleotide binding
Anion binding
Small molecule binding
Pyrophosphatase activity
Carbohydrate derivative binding
Actin filament binding
G
Purine ribonucleotide binding
Small molecule binding
Hydrolase activity, acting on acid anhydrides
Carbohydrate derivative binding
Nucleoside-triphosphatase activity
Nucleotide binding
Pyrophosphatase activity
Nucleoside phosphate binding
Ribonucleotide binding
Purine ribonucleoside triphosphate binding
Anion binding
Purine nucleotide binding

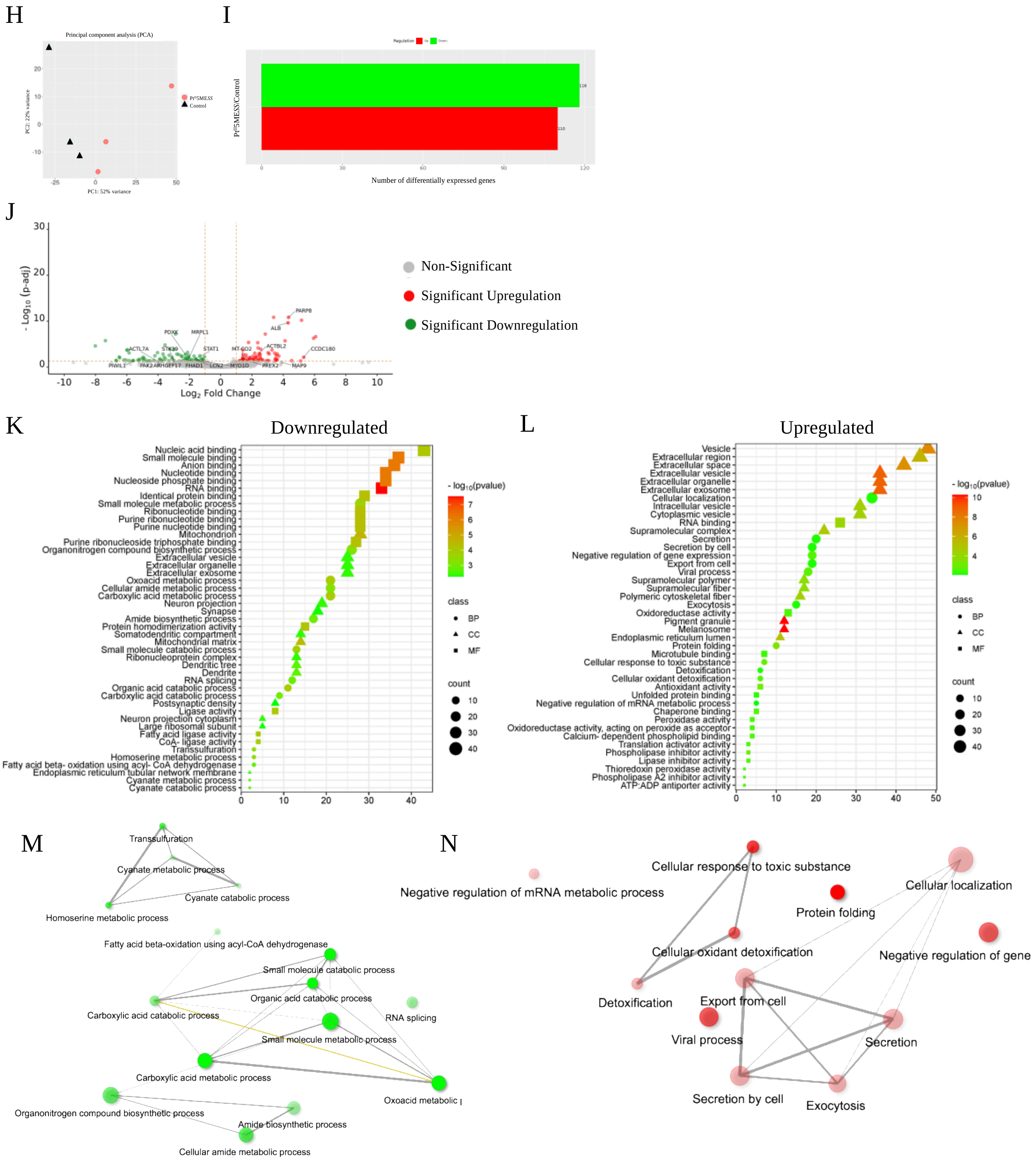

Figure S22. Proteomic analysis of MDA−MB−231 and HT29 upon treatment with **Pt^{II}5ME*SS*** as described in Section 2.15. A. MDA−MB−231 principal component analysis. B. MDA−MB−231 number of differentially expressed proteins (DEPs). C. MDA−MB−231 volcano plot of DEPs upregulated (red) and down regulated (green). GO enriched biological processes, cellular components, and molecular function in MDA−MB−231, D. Downregulated and E. Upregulated proteins. MDA−MB−231 pathway enrichment and gene act network analysis in most significance of F.

Downregulated pathways; mRNA metabolic process, GTP binding and nucleoside activity and G. Upregulated pathways; nucleotide binding and pyrophosphatase activity. H. HT29 principal component analysis. I. HT29 number of differentially expressed proteins (DEPs). J. HT29 volcano plot of DEPs upregulated (red) and down regulated (green). GO enriched biological processes, cellular components, and molecular function in HT29, K. Downregulated and L. Upregulated proteins. HT29 pathway enrichment and gene act network analysis in most significance of M. Downregulated pathways; molecular metabolic and catabolic processes and RNA splicing and N. Upregulated pathways; protein folding, response to toxic substance, detoxification, secretion, and exocytosis activity. Data points denote mean ± SEM. n = 3 from three independent experiments.

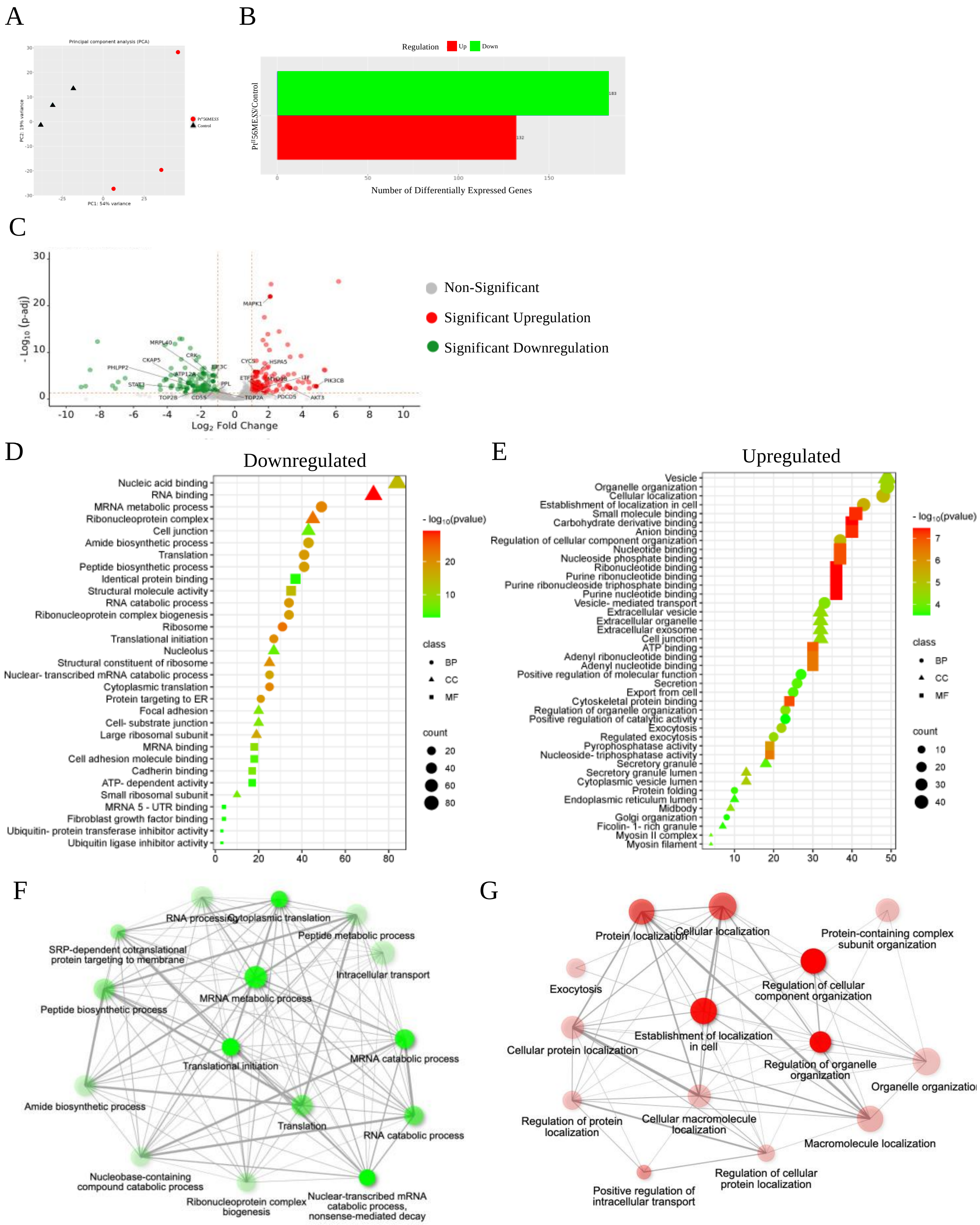

A
Principal component analysis (PCA)
PC1: 54% variance
PC2: 19% variance
Control
B
Regulation
Up
Down
183
132
Number of Differentially Expressed Genes
C
Non-Significant
Significant Upregulation
Significant Downregulation
- Log10 (p-adj)
Log2 Fold Change
MAPK1
MRPL40
CKAP5
CRK
PHLPP2
ATP12A
EIF3C
STAT1
TOP2B
CD55
PPL
CYCS
HSPA5
TOP2A
PDCD5
AKT3
PIK3CB
D
Downregulated
Nucleic acid binding
RNA binding
MRNA metabolic process
Ribonucleoprotein complex
Cell junction
Amide biosynthetic process
Translation
Peptide biosynthetic process
Identical protein binding
Structural molecule activity
RNA catabolic process
Ribonucleoprotein complex biogenesis
Ribosome
Translational initiation
Nucleolus
Structural constituent of ribosome
Nuclear- transcribed mRNA catabolic process
Cytoplasmic translation
Protein targeting to ER
Focal adhesion
Cell- substrate junction
Large ribosomal subunit
MRNA binding
Cell adhesion molecule binding
Cadherin binding
ATP- dependent activity
Small ribosomal subunit
MRNA 5 - UTR binding
Fibroblast growth factor binding
Ubiquitin- protein transferase inhibitor activity
Ubiquitin ligase inhibitor activity
- log10(pvalue)
class
BP
CC
MF
count
E
Upregulated
Vesicle
Organelle organization
Cellular localization
Establishment of localization in cell
Small molecule binding
Carbohydrate derivative binding
Anion binding
Regulation of cellular component organization
Nucleotide binding
Nucleoside phosphate binding
Ribonucleotide binding
Purine ribonucleotide binding
Purine ribonucleoside triphosphate binding
Purine nucleotide binding
Vesicle- mediated transport
Extracellular vesicle
Extracellular organelle
Extracellular exosome
Cell junction
ATP binding
Adenyl ribonucleotide binding
Adenyl nucleotide binding
Positive regulation of molecular function
Secretion
Export from cell
Cytoskeletal protein binding
Regulation of organelle organization
Positive regulation of catalytic activity
Exocytosis
Regulated exocytosis
Pyrophosphatase activity
Nucleoside- triphosphatase activity
Secretory granule
Secretory granule lumen
Cytoplasmic vesicle lumen
Protein folding
Endoplasmic reticulum lumen
Midbody
Golgi organization
Ficolin- 1- rich granule
Myosin II complex
Myosin filament
- log10(pvalue)
class
BP
CC
MF
count
F
RNA processing
Cytoplasmic translation
Peptide metabolic process
SRP-dependent cotranslational protein targeting to membrane
Intracellular transport
MRNA metabolic process
Peptide biosynthetic process
Translational initiation
MRNA catabolic process
Amide biosynthetic process
Translation
RNA catabolic process
Nucleobase-containing compound catabolic process
Ribonucleoprotein complex biogenesis
Nuclear-transcribed mRNA catabolic process, nonsense-mediated decay
G
Protein localization
Cellular localization
Protein-containing complex subunit organization
Exocytosis
Regulation of cellular component organization
Establishment of localization in cell
Cellular protein localization
Regulation of organelle organization
Organelle organizatio
Regulation of protein localization
Cellular macromolecule localization
Macromolecule localization
Regulation of cellular protein localization
Positive regulation of intracellular transport

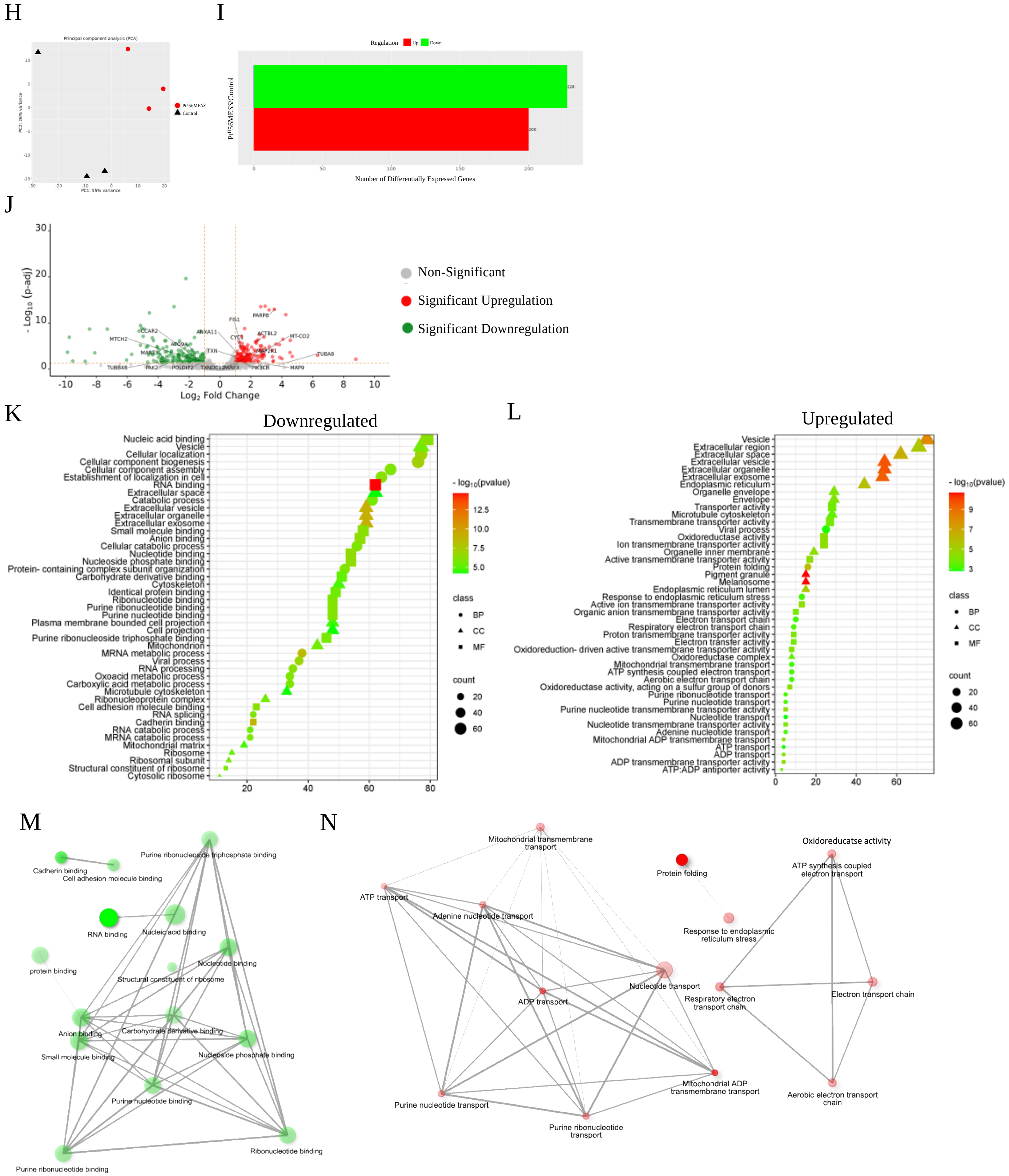

Figure S23. Proteomic analysis of MDA−MB−231 and HT29 upon treatment with **Pt^{II}56ME*SS*** as described in Section 2.15. A. MDA−MB−231 principal component analysis. B. MDA−MB−231 number of differentially expressed proteins (DEPs). C. MDA−MB−231 volcano plot of DEPs upregulated (red) and down regulated (green). GO enriched

biological processes, cellular components, and molecular function in MDA−MB−231, D. Downregulated and E. Upregulated proteins. MDA−MB−231 pathway enrichment and gene act network analysis in most significance of F. Downregulated pathways; translational initiation and mRNA metabolic process and G. Upregulated pathways; localisation in cell, cellular organisation, protein localisation and exocytosis. H. HT29 principal component analysis. I. HT29 number of differentially expressed proteins (DEPs). J. HT29 volcano plot of DEPs upregulated (red) and down regulated (green). GO enriched biological processes, cellular components, and molecular function in HT29, K. Downregulated and L. Upregulated proteins. HT29 pathway enrichment and gene act network analysis in most significance of M. Downregulated pathways; RNA binding and nucleotide binding and N. Upregulated pathways; ion transmembrane transport, active transport, and oxidoreductase activity. Data points denote mean ± SEM. n = 3 from three independent experiments.

Table S7. Antibody Concentrations used in Western Blot

Supplier	Clone no.	Clone	Primary Antibody Target	Molecular Weight
Abcam	ab32503	Rabbit monoclonal	BAX	21 KDa
Abcam	ab196495	Rabbit polyclonal	Bcl2	26 KDa
Abcam	ab32138	Rabbit monoclonal	PARP-1	pro-form 113 Kda and 25 Kda (N-terminal catalytic domain) cleaved form of PARP1
Abcam	ab133504	Rabbit monoclonal	Cytochrome C	11 or 14 KDa
Abcam	ab214430	Rabbit monoclonal	Caspase 3	32 Kda and cleaved fragments at 17, 19 or 24 Kda
Abcam	ab108333	Rabbit monoclonal	Caspase 8	55 KDa
Abcam	ab202068	Rabbit monoclonal	Caspase 9	46, KDa
Abcam	ab8227	Rabbit polyclonal	Beta Actin	42 KDa
Abcam	ab7291	Mouse monoclonal	Alpha Tubulin	50 KDa
Abcam	ab6046	Rabbit Polyclonal	Beta Tubulin	50 KDa
Abcam	ab32389	Rabbit monoclonal	p53	44 KDa
Abcam	ab109520	Rabbit monoclonal	p21	21 KDa
Abcam	ab192591	Rabbit polyclonal	p-ERK	41-44 KDa
Abcam	ab184699	Rabbit monoclonal	ERK	42 KDa
Abcam	ab38449	Rabbit polyclonal	p-AKT	56 KDa
Abcam	ab8805	Rabbit polyclonal	AKT	56 KDa
Abcam	ab8245	Mouse monoclonal	GAPDH	36 or 40 KDa
Abcam	ab228668	Rabbit polyclonal	APG5L/ATG5	predicted at 32 KDA but seen at 55 Kda
Abcam	ab228525	Rabbit polyclonal	ATG16L1 - N-terminal	68-70 Kda
			ATG4B	44 Kda
			ATG9A	94-100 Kda
			Beclin-1	52 Kda
			LC3B	14-16 Kda
Abcam	ab10640	Rabbit polyclonal	Bid Cleavage Site	15 KDa

The representative full blots below include proteins from several projects run in parallel. Proteins related to this study are labelled lane 1-8 for the relevant treatment.

A

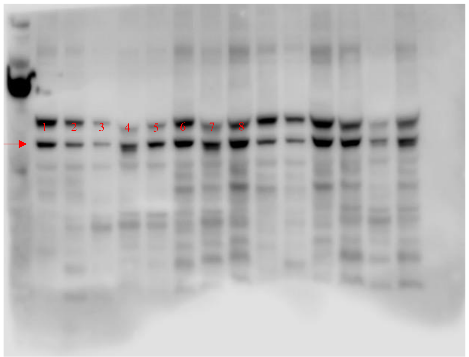

β-actin

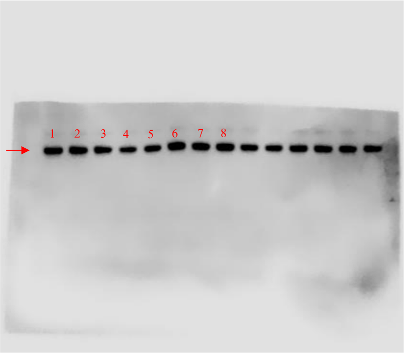

GAPDH

β-actin/ GAPDH normalized to control (1)	**1**	**2**	**3**	**4**	**5**	**6**	**7**	**8**
	1	0.58050004	0.47443239	0.70824343	0.59555736	0.83501972	0.87880796	0.66714398

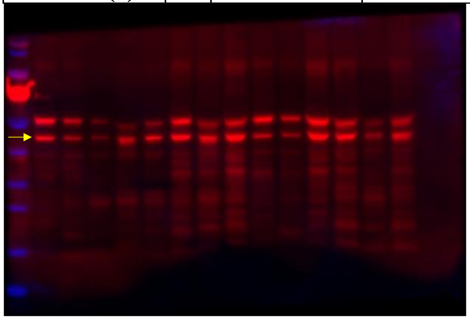

β-actin

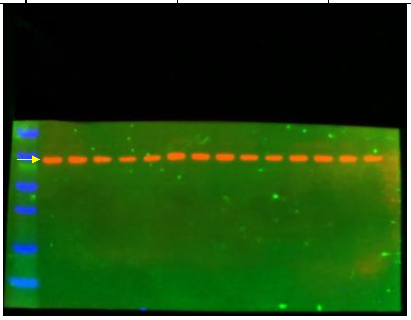

GAPDH

Representative uncropped western blot is presented with protein of interest directed with a red arrow and lanes marked 1-8 in red, on the chemiluminescence blot. Representative fluorescent western blot with molecular marker (PageRuler Prestained Protein Ladder (Invitrogen #26617)) was detected using laser 600 and 700 on the Odyssey® FC imaging system and protein of interest directed with a yellow arrow.

B

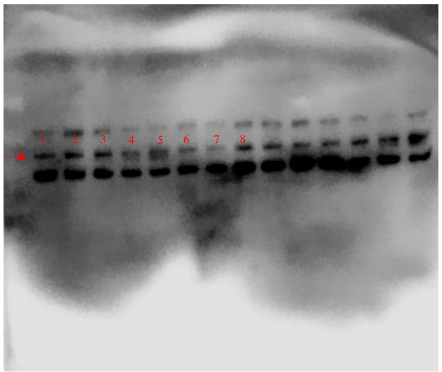

β-tubulin

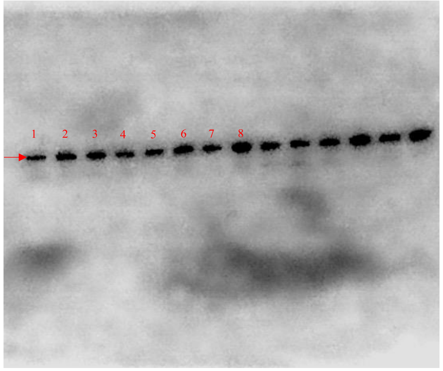

GAPDH

β-tubulin/ GAPDH normalized to control (1)	**1**	**2**	**3**	**4**	**5**	**6**	**7**	**8**
	1	1.19453363	1.15028482	0.41633332	0.59777244	0.65519285	0.39846695	0.42888854

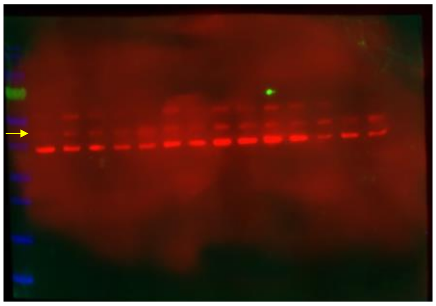

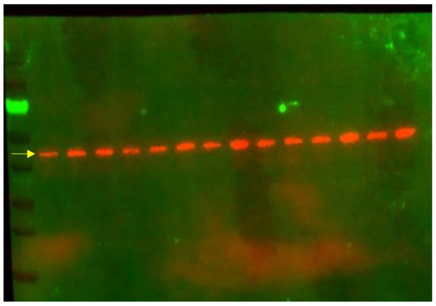

β-tubulin **GAPDH**

Representative uncropped western blot is presented with protein of interest directed with a red arrow and lanes marked 1-8 in red, on the chemiluminescence blot. Representative fluorescent western blot with molecular marker (PageRuler Prestained Protein Ladder (Invitrogen #26617)) was detected using laser 600 and 700 on the Odyssey® FC imaging system and protein of interest directed with a yellow arrow.

C

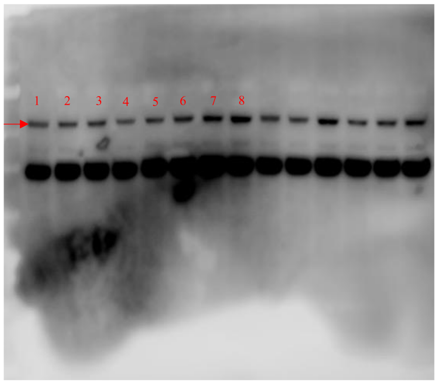

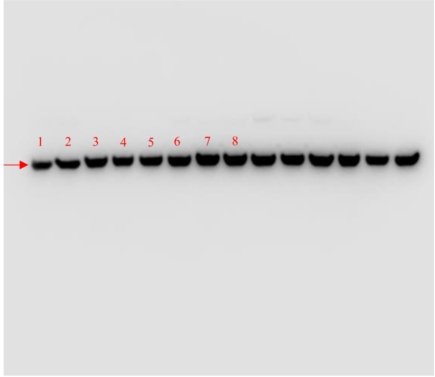

α-tubulin **GAPDH**

α-tubulin/ GAPDH normalized to control (1)	**1**	**2**	**3**	**4**	**5**	**6**	**7**	**8**
	1	0.94863864	0.93546771	0.63992484	0.7886653	0.92790063	1.17680214	1.35918472

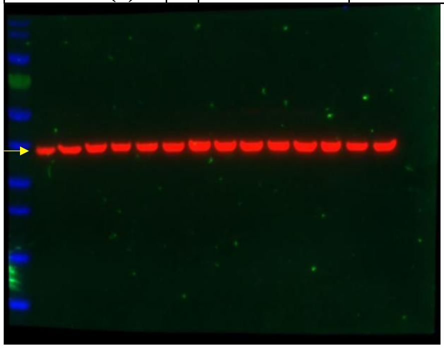

GAPDH

Representative uncropped western blot is presented with protein of interest directed with a red arrow and lanes marked 1-8 in red, on the chemiluminescence blot. Representative fluorescent western blot of GAPDH with molecular marker (PageRuler Prestained Protein Ladder (Invitrogen #26617)) was detected using laser 600 and 700 on the Odyssey® FC imaging system and protein of interest directed with a yellow arrow. α-tubulin molecular marker can be seen on the chemiluminescence blot and tracked using the GAPDH fluorescent blot, given α-tubulin falls above GAPDH.

Figure S24. Full representative western blot of microtubule cytoskeleton protein markers in MDA−MB−231. Represented data normalized to GAPDH relative to control (lane 1). A. β-actin, B. β-tubulin and C. α-tubulin. Protein expression upon treatment with platinum(II) (**Pt^{II}PHEN*SS*** (lane 3), **Pt^{II}5ME*SS*** (lane 4) and **Pt^{II}56ME*SS*** (lane 5))

and platinum(IV) (**Pt^{II}PHEN*SS*(OH)$_2$** (lane 6), **Pt^{IV}5ME*SS*(OH)$_2$** (lane 7) and **Pt^{IV}56ME*SS*(OH)$_2$** (lane 8)) complexes, as well as cisplatin (lane 2) in MDA−MB−231 cells at 72 h compared with control (lane 1).

A

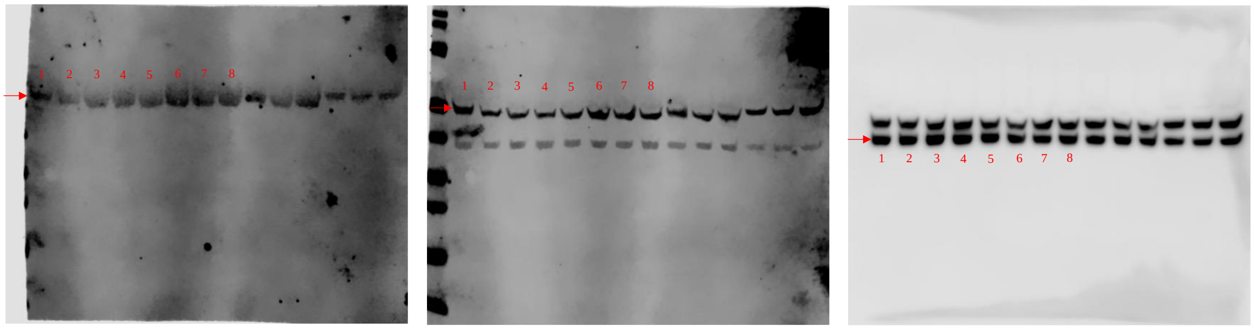

p-AKT **AKT** **GAPDH**

p-AKT/AKT /GAPDH normalized to control (1)	**1**	**2**	**3**	**4**	**5**	**6**	**7**	**8**
	1	0.6180738	0.60416678	0.53879108	0.52290527	1.06325105	0.93449426	0.79004776

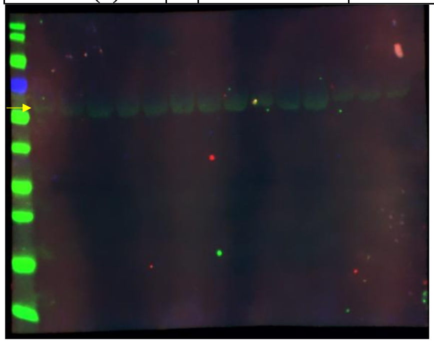

p-AKT

Representative uncropped western blot is presented with protein of interest directed with a red arrow and lanes marked 1-8 in red, on the chemiluminescence blot. Representative fluorescent western blot of p-AKT with molecular marker (PageRuler Prestained Protein Ladder (Invitrogen #26617)) was detected using laser 600 and 700 on the Odyssey® FC imaging system and protein of interest directed with a yellow arrow. AKT and GAPDH molecular marker can be seen on the chemiluminescence blot and tracked using the p-AKT fluorescent blot, given AKT will fall at the same molecular weight and GAPDH will fall below.

B

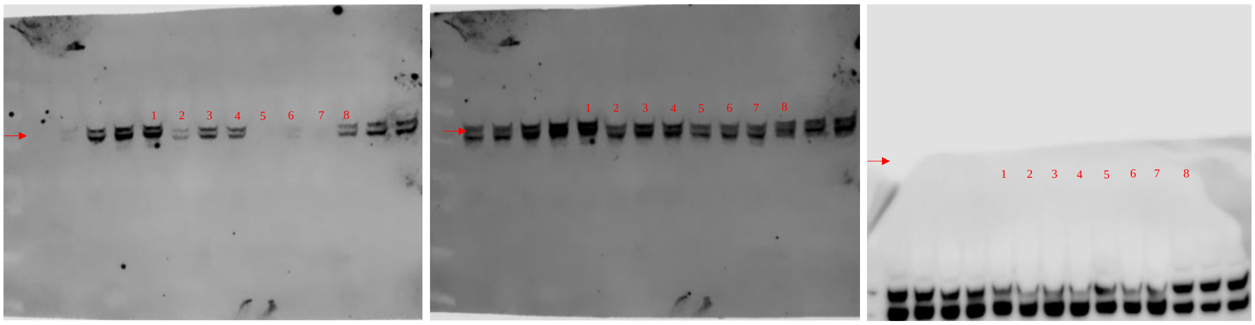

p-ERK **ERK** **GAPDH**

p-ERK/ERK /GAPDH normalized to control (1)	**1**	**2**	**3**	**4**	**5**	**6**	**7**	**8**
	1	0.21229775	0.67705437	0.59471979	0.06080849	0.12062363	0.14403014	0.28275594

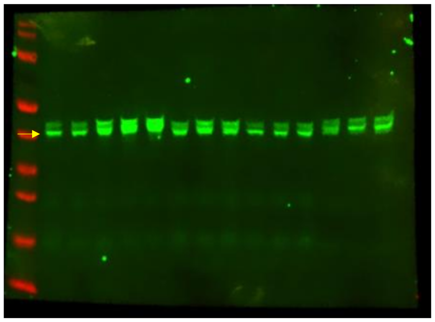

ERK

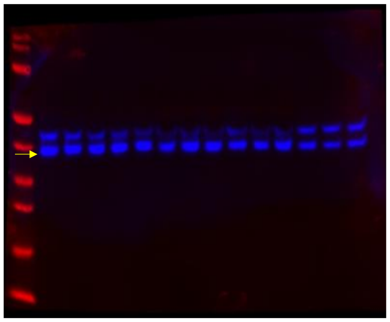

GAPDH

Representative uncropped western blot is presented with protein of interest directed with a red arrow and lanes marked 1-8 in red, on the chemiluminescence blot. Representative fluorescent western blot of ERK and GAPDH with molecular marker (PageRuler Prestained Protein Ladder (Invitrogen #26617)) was detected using laser 600 on the Odyssey® FC imaging system and protein of interest directed with a yellow arrow. p-ERK molecular marker can be seen on the chemiluminescence blot and tracked using the ERK fluorescent blot, given p-ERK will fall at the same molecular weight.

C

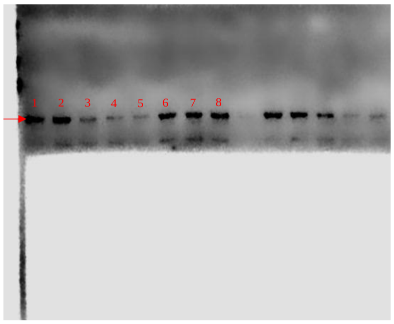

p53

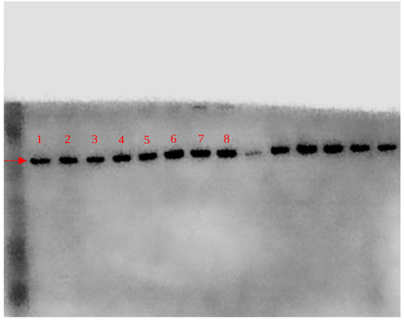

GAPDH

p53/GAPDH normalized to control (1)	**1**	**2**	**3**	**4**	**5**	**6**	**7**	**8**
	1	1.58464538	1.10500528	0.78093946	0.61872139	1.17637615	1.18271952	1.18294515

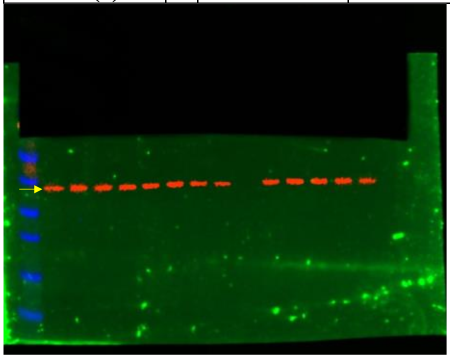

GAPDH

Representative uncropped western blot is presented with protein of interest directed with a red arrow and lanes marked 1-8 in red, on the chemiluminescence blot. Representative fluorescent western blot of GAPDH with molecular marker (PageRuler Prestained Protein Ladder (Invitrogen #26617)) was detected using laser 600 on the Odyssey® FC imaging system and protein of interest directed with a yellow arrow. p53 molecular marker can be tracked on the chemiluminescence blot using the GAPDH fluorescent blot, given p53 will fall above GAPDH.

D

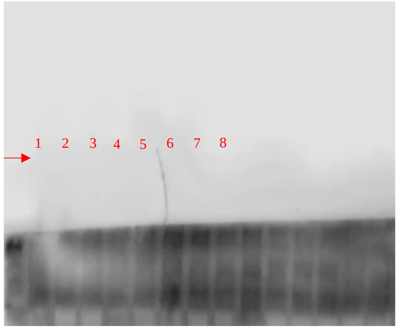

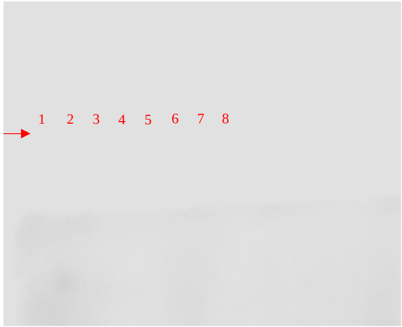

p21 **GAPDH**

p21/GAPDH normalized to control (1)	**1**	**2**	**3**	**4**	**5**	**6**	**7**	**8**
	1	1.35060326	1.81212007	2.47783829	2.59969343	1.34603696	1.0418355	1.0525943

Representative uncropped western blot is presented with protein of interest directed with a red arrow and lanes marked 1-8 in red, on the chemiluminescence blot. Representative fluorescent western blot could not be detected with fault in lasers 600 and 700 of Odyssey® FC imaging system requiring replacement. Molecular marker (PageRuler Prestained Protein Ladder (Invitrogen #26617)) was still loaded to the left of the gel and can still be observed in p21 chemiluminescent blot. Protein markers p21 and GAPDH were matched for confirmation with the molecular marker and antibodies reference in Table S7, given previous observational knowledge of GAPDH fluorescent blots.

Figure S25. Full representative western blot of cell proliferation protein markers in MDA−MB−231. Represented data normalized to GAPDH relative to control (lane 1). A. p-AKT/AKT, B. p-ERK/ERK, C. p53 and D. p21. Protein expression upon treatment with platinum(II) (**Pt^{II}PHEN*SS*** (lane 3), **Pt^{II}5ME*SS*** (lane 4) and **Pt^{II}56ME*SS*** (lane 5)) and platinum(IV) (**Pt^{II}PHEN*SS*(OH)$_2$** (lane 6), **Pt^{IV}5ME*SS*(OH)$_2$** (lane 7) and **Pt^{IV}56ME*SS*(OH)$_2$** (8)) complexes, as well as cisplatin (lane 2) in MDA−MB−231 cells at 72 h compared with control (lane 1).

A

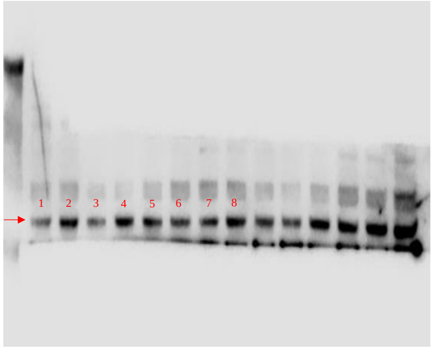

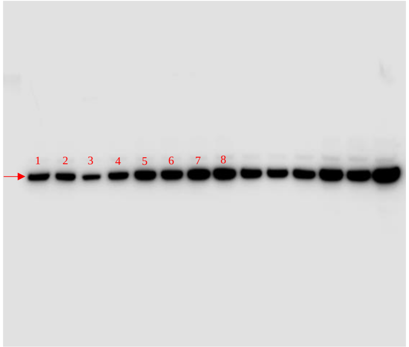

Bax **GAPDH**

Bax/GAPDH normalized to control (1)	**1**	**2**	**3**	**4**	**5**	**6**	**7**	**8**
	1	1.83635249	1.16069247	1.88631075	1.27646318	1.39794735	1.84635944	2.60047266

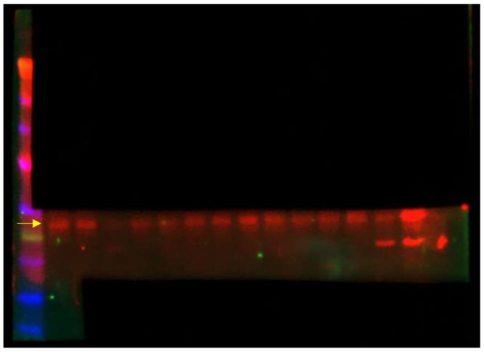

Bax

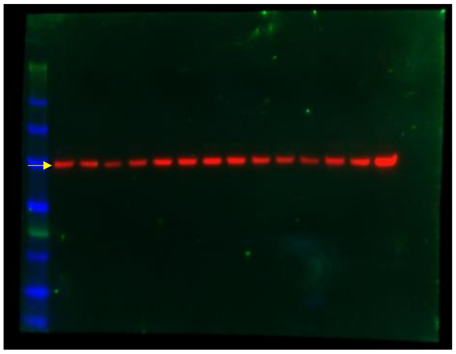

GAPDH

Representative uncropped western blot is presented with protein of interest directed with a red arrow and lanes marked 1-8 in red, on the chemiluminescence blot. Representative fluorescent western blot of Bax and GAPDH with molecular marker (SeeBlue Prestained Protein Standard (Invitrogen #LC5625)) was detected using laser 600 and 700 on the Odyssey® FC imaging system and protein of interest directed with a yellow arrow.

B

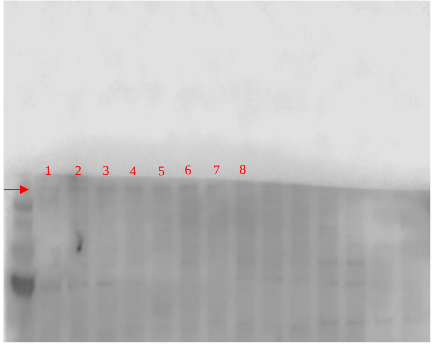

Bcl2

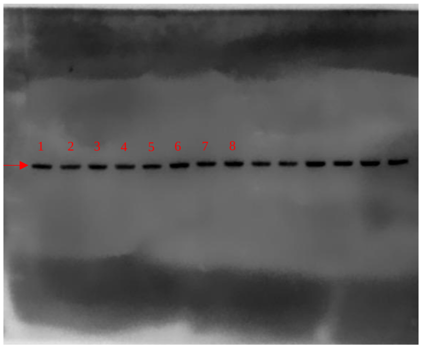

GAPDH

Bcl2/GAPDH normalized to control (1)	**1**	**2**	**3**	**4**	**5**	**6**	**7**	**8**
	1	0.75285082	0.8361108	0.50739129	0.68460454	0.78430242	0.79136982	0.8651495

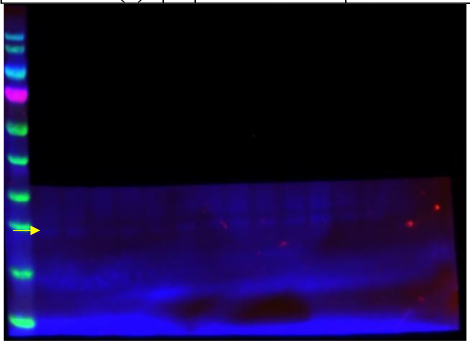

Bcl2

Representative uncropped western blot is presented with protein of interest directed with a red arrow and lanes marked 1-8 in red, on the chemiluminescence blot. Representative fluorescent western blot of Bcl2 with molecular marker (PageRuler Prestained Protein Ladder (Invitrogen #26617)) was detected using laser 600 and 700 on the Odyssey® FC imaging system and protein of interest directed with a yellow arrow. GAPDH molecular marker fluorescent blot was not obtained but can be seen on the chemiluminescence blot and tracked using the Bcl2 fluorescent blot, given GAPDH will fall above.

C

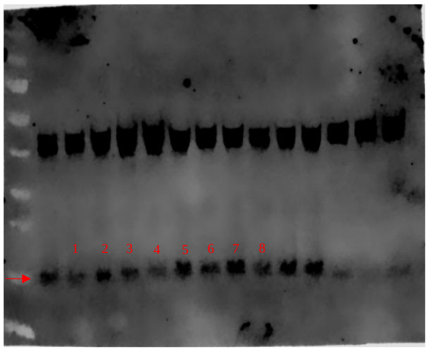

Cytochrome C

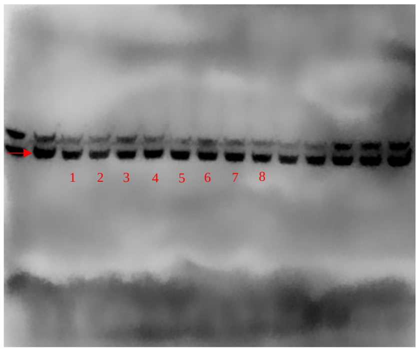

GAPDH

Cytochrome C /GAPDH normalized to control (1)	1	2	3	4	5	6	7	8
	1	2.37118767	1.82721713	1.51269724	3.10869364	2.37833562	2.79548123	2.18464815

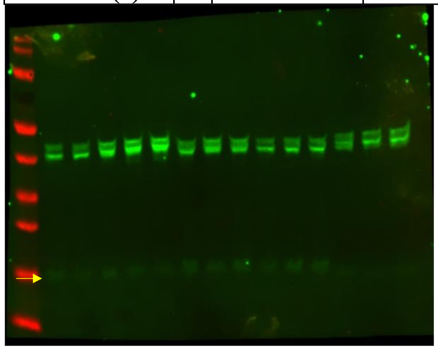

Cytochrome C

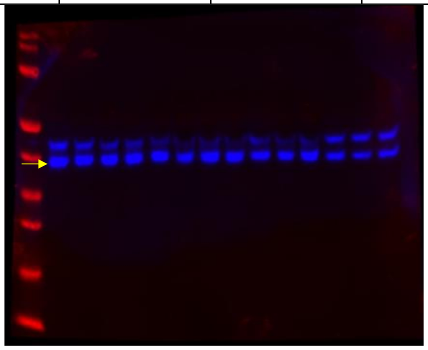

GAPDH

Representative uncropped western blot is presented with protein of interest directed with a red arrow and lanes marked 1-8 in red, on the chemiluminescence blot. Representative fluorescent western blot with molecular marker (PageRuler Prestained Protein Ladder (Invitrogen #26617)) was detected using laser 600 on the Odyssey® FC imaging system and protein of interest directed with a yellow arrow.

D

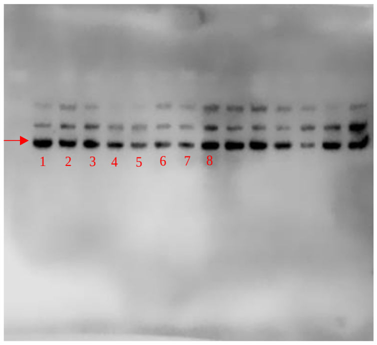

Procaspase 8

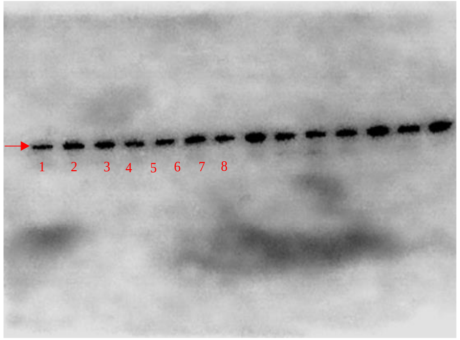

GAPDH

Procaspase 8 /GAPDH normalized to control (1)	1	2	3	4	5	6	7	8
	1	0.56815789	0.51949587	0.50994632	0.35839375	0.28884874	0.35460896	0.56804293

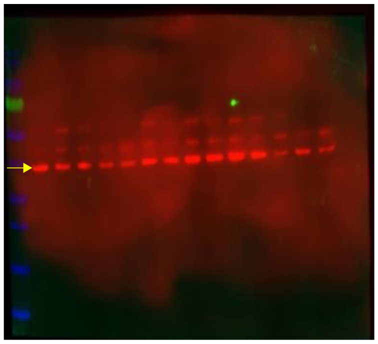

Procaspase 8

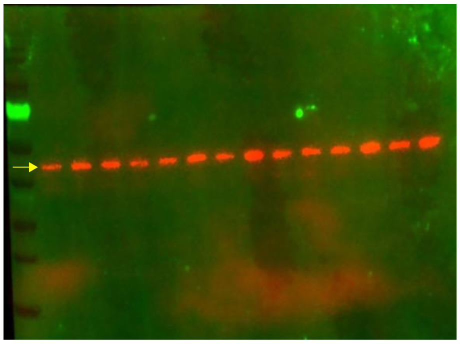

GAPDH

Representative uncropped western blot is presented with protein of interest directed with a red arrow and lanes marked 1-8 in red, on the chemiluminescence blot. Representative fluorescent western blot with molecular marker (PageRuler Prestained Protein Ladder (Invitrogen #26617)) was detected using laser 600 and 700 on the Odyssey® FC imaging system and protein of interest directed with a yellow arrow.

E

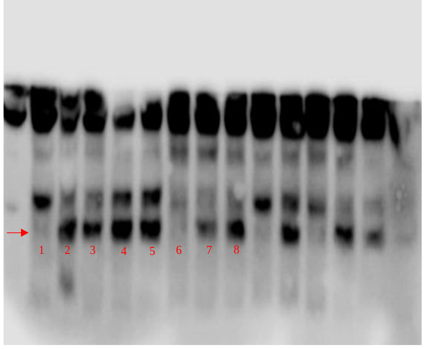

Cleaved BID

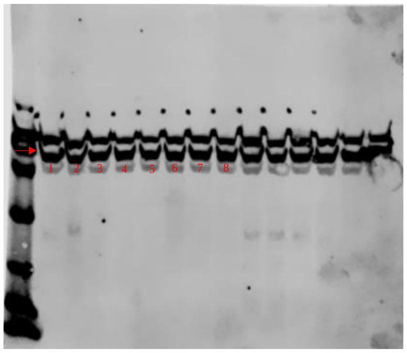

GAPDH

Cleaved BID /GAPDH normalized to control (1)	**1**	**2**	**3**	**4**	**5**	**6**	**7**	**8**
	1	11.0692064	13.3905609	14.2890745	14.7401877	1.3298651	5.9481588	8.5326365

Representative uncropped western blot is presented with protein of interest directed with a red arrow and lanes marked 1-8 in red, on the chemiluminescence blot. Representative fluorescent western blot could not be detected with fault in lasers 600 and 700 of Odyssey® FC imaging system requiring replacement. However, the appearance of the molecular marker (SeeBlue Prestained Protein Standard (Invitrogen #LC5625)) on the chemiluminescence blot in cleaved BID and GAPDH blots confirm the detected band position. Protein markers were matched for confirmation with the molecular marker and antibodies reference in Table S7, given previous observational knowledge of GAPDH fluorescent blots.

F

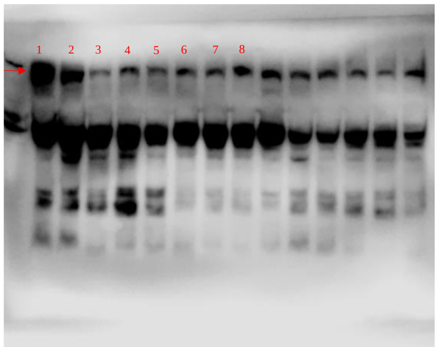

Procaspase 9

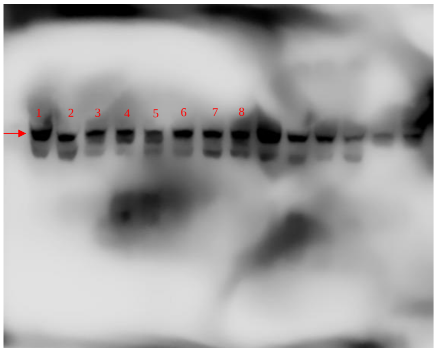

GAPDH

Procaspase 9 /GAPDH normalized to control (1)	**1**	**2**	**3**	**4**	**5**	**6**	**7**	**8**
	1	1.12199504	0.20416262	0.31011077	0.47547078	0.26748363	0.30218544	0.45565747

Representative uncropped western blot is presented with protein of interest directed with a red arrow and lanes marked 1-8 in red, on the chemiluminescence blot. Representative fluorescent western blot could not be detected with fault in lasers 600 and 700 of Odyssey® FC imaging system requiring replacement. Molecular marker (PageRuler Prestained Protein Ladder (Invitrogen #26617)) was still loaded to the left of the gel and can still be somewhat observed in procaspase 9 chemiluminescent blot. Protein markers procaspase 9 and GAPDH were matched for confirmation with the molecular marker and antibodies reference in Table S7, given previous observational knowledge of GAPDH fluorescent blots.

G

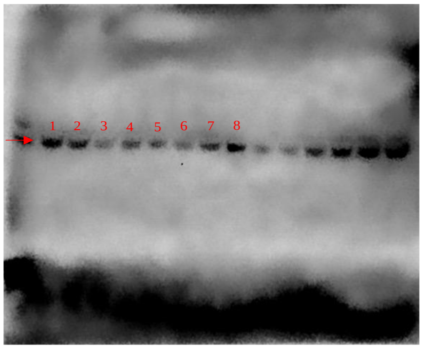

Procaspase 3

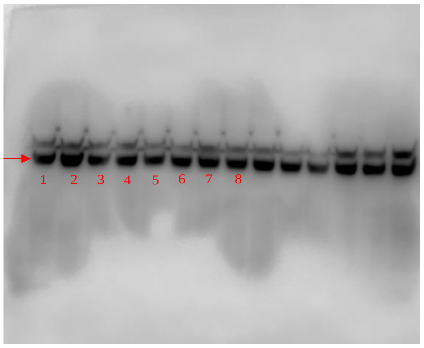

GAPDH

Procaspase 3 /GAPDH normalized to control (1)	**1**	**2**	**3**	**4**	**5**	**6**	**7**	**8**
	1	0.8252645	0.32070402	0.54050899	0.4243036	0.40928799	0.71667593	0.94708058

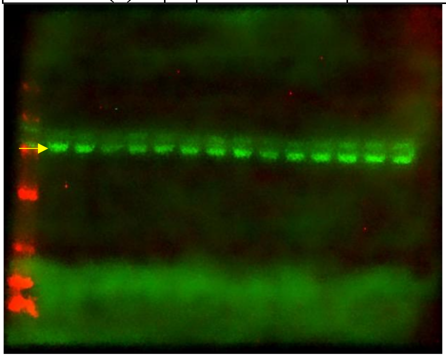

Procaspase 3

Representative uncropped western blot is presented with protein of interest directed with a red arrow and lanes marked 1-8 in red, on the chemiluminescence blot. Representative fluorescent western blot of Procaspase 3 with molecular marker (SeeBlue Prestained Protein Standard (Invitrogen #LC5625)) was detected using laser 600 on the Odyssey® FC imaging system and protein of interest directed with a yellow arrow. GAPDH molecular marker fluorescent blot was not obtained but can be seen on the chemiluminescence blot and tracked using the Procaspase 3 fluorescent blot, given GAPDH will in the same molecular range.

H

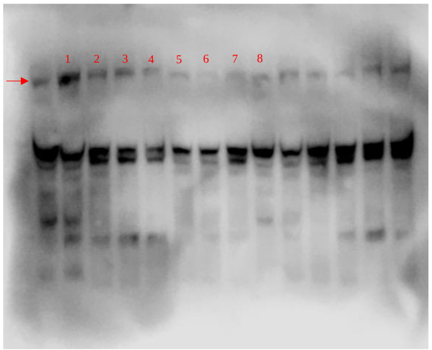

PARP-1

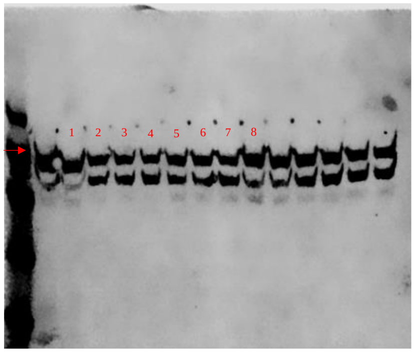

GAPDH

PARP-1 /GAPDH normalized to control (1)	**1**	**2**	**3**	**4**	**5**	**6**	**7**	**8**
	1	0.78666175	0.77749498	0.76929938	0.63398891	0.57165935	0.51867466	0.61815111

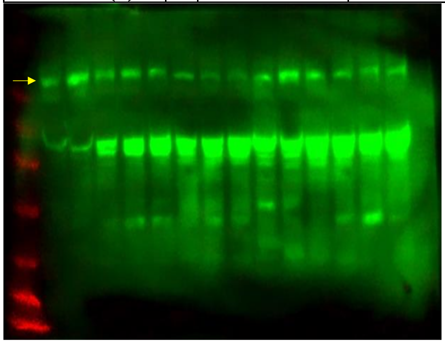

PARP-1

Representative uncropped western blot is presented with protein of interest directed with a red arrow and lanes marked 1-8 in red, on the chemiluminescence blot. Representative fluorescent western blot of PARP-1 with molecular marker (SeeBlue Prestained Protein Standard (Invitrogen #LC5625)) was detected using laser 600 on the Odyssey® FC imaging system and protein of interest directed with a yellow arrow. GAPDH molecular marker fluorescent blot was not obtained but can be seen on the chemiluminescence blot and tracked using the PARP-1 fluorescent blot for marker reference, given previous observational knowledge of GAPDH.

Figure S26. Full representative western blot of intrinsic and extrinsic apoptotic cell death markers in MDA−MB−231. Represented data normalized to GAPDH relative to control (lane 1). A. Bax, B. Bcl2, C. Cytochrome C, D.Procaspase 8, E. Cleaved BID, F. Procaspase 9, G. Procaspase 3 and H. PARP-1. Protein expression upon treatment with platinum(II) (**Pt^{II}PHEN*SS*** (lane 3), **Pt^{II}5ME*SS*** (lane 4) and **Pt^{II}56ME*SS*** (lane 5)) and platinum(IV) (**Pt^{II}PHEN*SS*(OH)$_2$** (lane 6), **Pt^{IV}5ME*SS*(OH)$_2$** (lane 7) and **Pt^{IV}56ME*SS*(OH)$_2$** (lane 8)) complexes, as well as cisplatin (lane 2) in MDA−MB−231 cells at 72 h compared with control (lane 1).

A

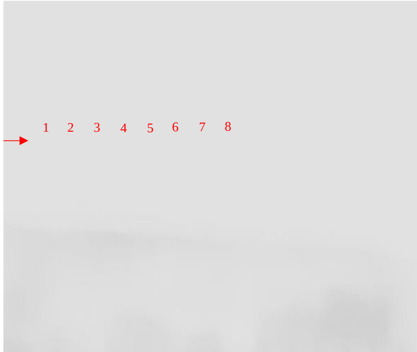

Beclin1

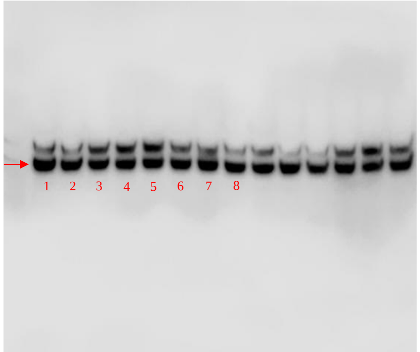

GAPDH

Beclin1 /GAPDH normalized to control (1)	1	2	3	4	5	6	7	8
	1	0.92112095	1.91053049	2.58803175	3.1497487	1.40825406	0.93739607	0.95917922

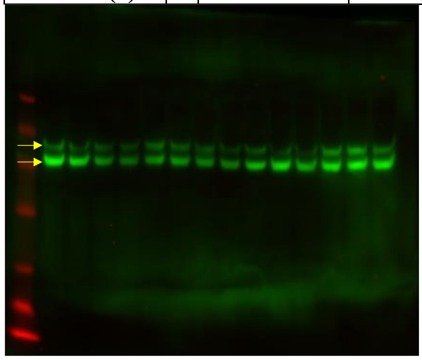

Beclin1 -top
GAPDH -bottom

Representative uncropped western blot is presented with protein of interest directed with a red arrow and lanes marked 1-8 in red, on the chemiluminescence blot. Representative fluorescent western blot with molecular marker (PageRuler Prestained Protein Ladder (Invitrogen #26617)) was detected using laser 600 on the Odyssey® FC imaging system and protein of interest directed with a yellow arrow.

B

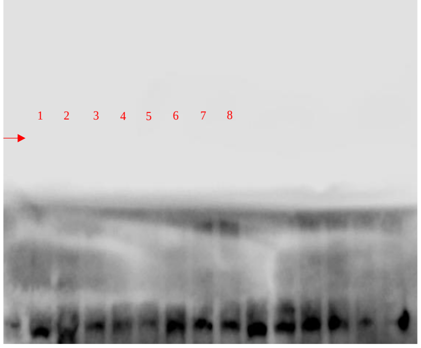

APG5L/ATG5

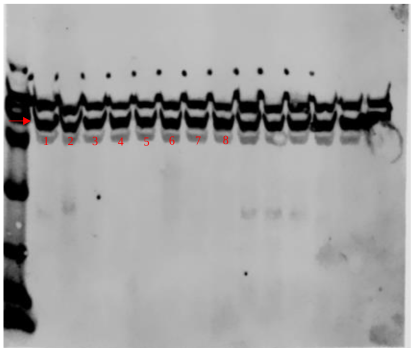

GAPDH

ATG5 /GAPDH normalized to control (1)	1	2	3	4	5	6	7	8
	1	0.82158927	0.7761884	0.5948127	0.40117138	1.12853361	1.06548285	0.89236817

Representative uncropped western blot is presented with protein of interest directed with a red arrow and lanes marked 1-8 in red, on the chemiluminescence blot. Representative fluorescent western blot could not be detected with fault in lasers 600 and 700 of Odyssey® FC imaging system requiring replacement. However, the appearance of the molecular

marker (SeeBlue Prestained Protein Standard (Invitrogen #LC5625)) on the chemiluminescence blot in APG5L/ATG5 and GAPDH blots confirm the detected band position. Protein markers were matched for confirmation with the molecular marker and antibodies reference in Table S7, given previous observational knowledge of GAPDH fluorescent blots.

C

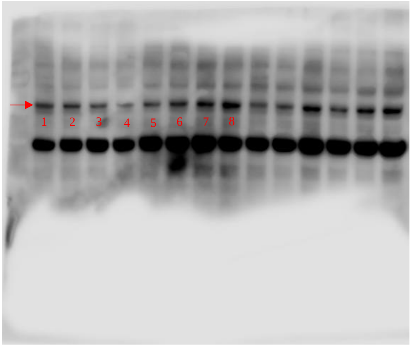

ATG16L1

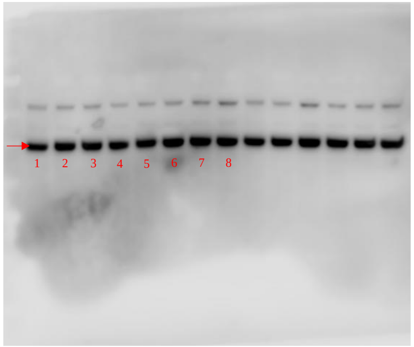

GAPDH

ATG16L1 /GAPDH normalized to control (1)	1	2	3	4	5	6	7	8
	1	0.95116116	0.6757997	0.47416701	0.57264767	0.99866099	1.20282487	1.45636448

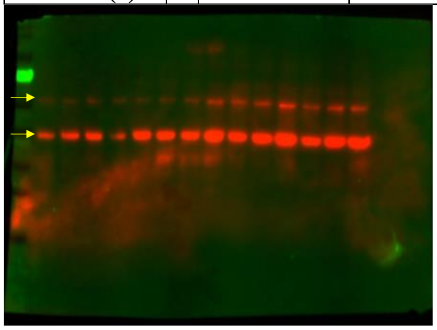

ATG16L1 -top
GAPDH -bottom

Representative uncropped western blot is presented with protein of interest directed with a red arrow and lanes marked 1-8 in red, on the chemiluminescence blot. Representative fluorescent western blot with molecular marker (PageRuler Prestained Protein Ladder (Invitrogen #26617)) was detected using laser 600 and 700 on the Odyssey® FC imaging system and protein of interest directed with a yellow arrow.

D

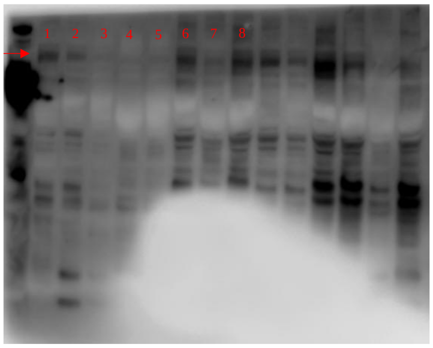

ATG9A

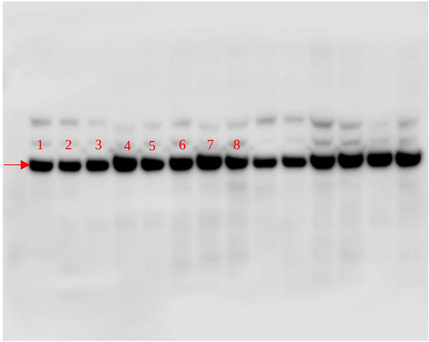

GAPDH

ATG9A/ GAPDH	1	2	3	4	5	6	7	8
	1	0.65513002	0.36100555	0.28820224	0.28482081	0.77401735	0.39841324	0.88224084

normalized to control (1)								

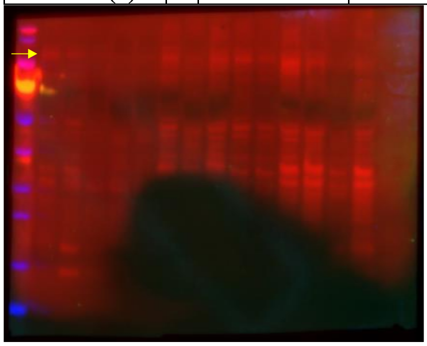

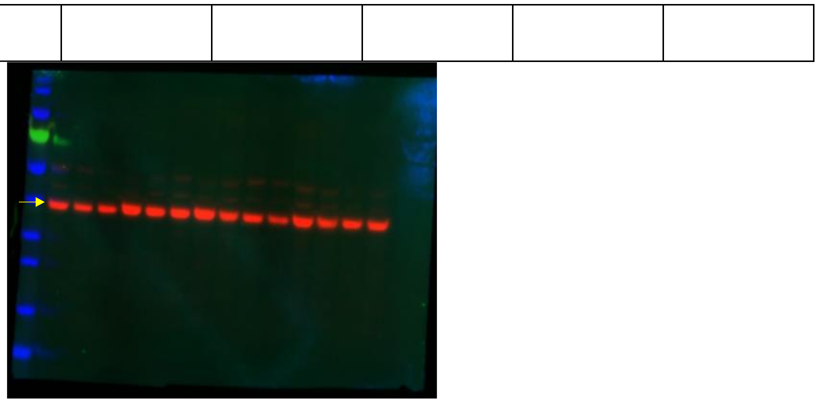

ATG9A **GAPDH**

Representative uncropped western blot is presented with protein of interest directed with a red arrow and lanes marked 1-8 in red, on the chemiluminescence blot. Representative fluorescent western blot with molecular marker (PageRuler Prestained Protein Ladder (Invitrogen #26617)) was detected using laser 600 and 700 on the Odyssey® FC imaging system and protein of interest directed with a yellow arrow.

E

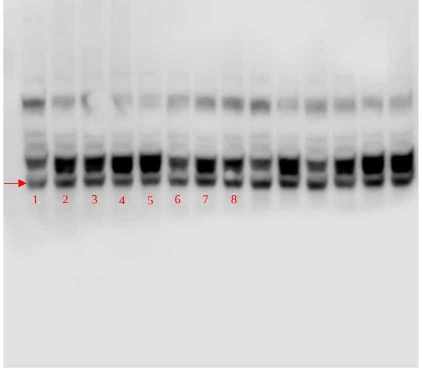

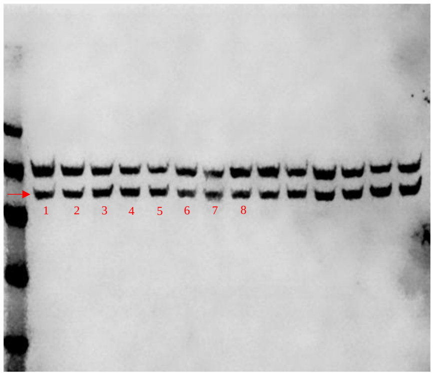

ATG4B **GAPDH**

ATG4B/ GAPDH normalized to control (1)	**1**	**2**	**3**	**4**	**5**	**6**	**7**	**8**
	1	1.55113158	1.54556964	1.83877154	2.10690916	1.58129772	2.7974247	2.17850114

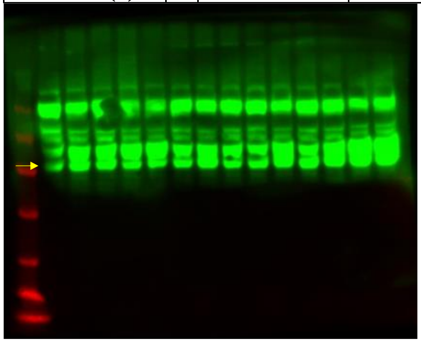

ATG4B

Representative uncropped western blot is presented with protein of interest directed with a red arrow and lanes marked 1-8 in red, on the chemiluminescence blot. Representative fluorescent western blot with molecular marker (PageRuler Prestained Protein Ladder (Invitrogen #26617)) was detected using laser 600 on the Odyssey® FC imaging system and protein of interest directed with a yellow arrow. Fluorescent western blot has not been obtained for GAPDH, however, the appearance of the molecular marker in GAPDH chemiluminescence blot is observed.

F

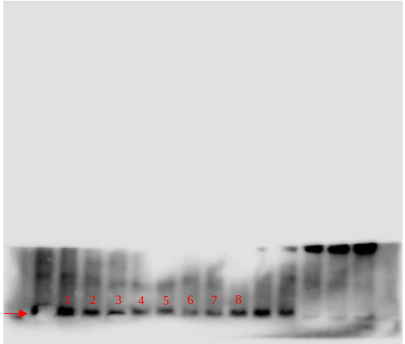

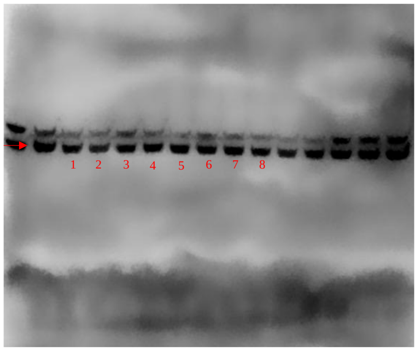

LC3B **GAPDH**

LC3B/ GAPDH normalized to control (1)	**1**	**2**	**3**	**4**	**5**	**6**	**7**	**8**
	1	1.36804873	0.99689688	0.93663875	0.84782246	0.60763292	0.6059369	0.7501472

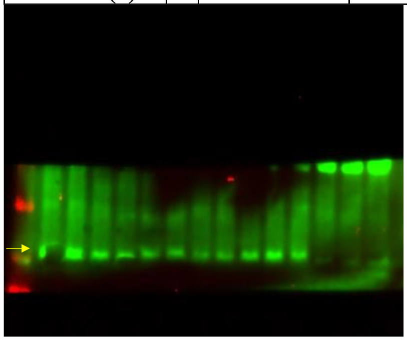

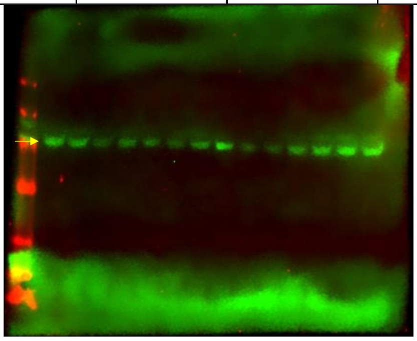

LC3B **GAPDH**

Representative uncropped western blot is presented with protein of interest directed with a red arrow and lanes marked 1-8 in red, on the chemiluminescence blot. Representative fluorescent western blot with molecular marker (PageRuler Prestained Protein Ladder (Invitrogen #26617)) was detected using laser 600 on the Odyssey® FC imaging system and protein of interest directed with a yellow arrow.

Figure S27. Full representative western blot of autophagy markers in MDA−MB−231. Represented data normalized to GAPDH relative to control (lane 1). A. Beclin1, B. APG5L/ATG5, C. ATG16L1, D. ATG9A, E. ATG4B and F. LC3B. Protein expression upon treatment with platinum(II) (**Pt^{II}PHEN*SS*** (lane 3), **Pt^{II}5ME*SS*** (lane 4) and **Pt^{II}56ME*SS*** (lane 5)) and platinum(IV) (**Pt^{II}PHEN*SS*(OH)$_2$** (lane 6), **Pt^{IV}5ME*SS*(OH)$_2$** (lane 7) and **Pt^{IV}56ME*SS*(OH)$_2$** (lane 8)) complexes, as well as cisplatin (lane 2) in MDA−MB−231 cells at 72 h compared with control (lane 1).

A

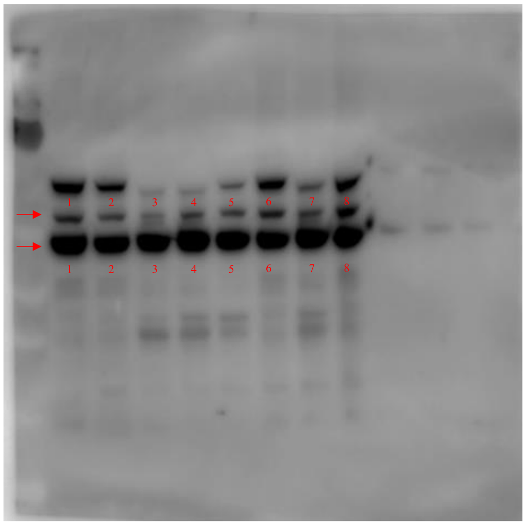

β-actin -top

GAPDH -bottom

β-actin/ GAPDH normalized to control (1)	**1**	**2**	**3**	**4**	**5**	**6**	**7**	**8**
	1	0.63447655	0.12673082	0.2979228	0.49425744	0.89432236	0.29479307	1.20892896

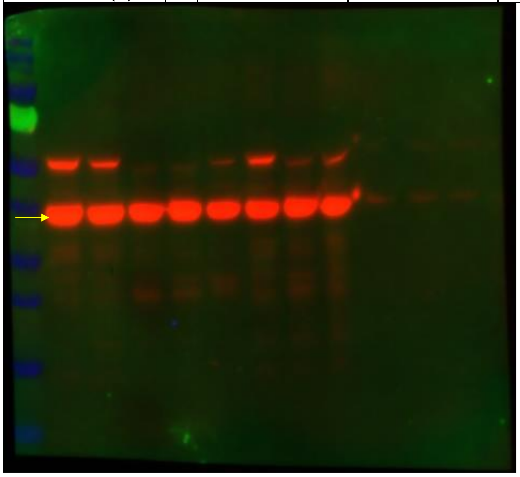

GAPDH

Representative uncropped western blot is presented with protein of interest directed with a red arrow and lanes marked 1-8 in red, on the chemiluminescence blot. Representative fluorescent western blot with molecular marker (PageRuler Prestained Protein Ladder (Invitrogen #26617)) was detected using laser 600 and 700 on the Odyssey® FC imaging system and protein of interest directed with a yellow arrow. Fluorescent western blot has not been obtained for β-actin, however, the appearance of the molecular marker in GAPDH chemiluminescence blot is observed. While the top observed protein marker (above GAPDH) is procaspase 9, β-actin can be seen to fall between procaspase 9 and GAPDH on the chemiluminescence blot and accordingly can be estimated and confirmed on the fluorescence blot.

B

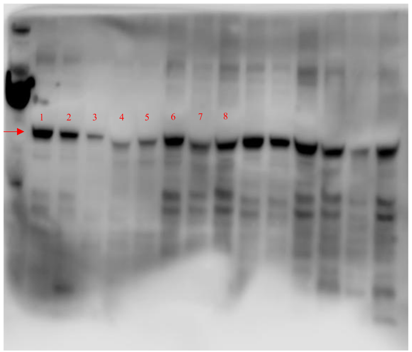

***β*-tubulin**

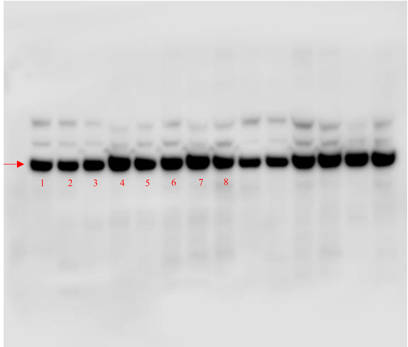

GAPDH

β-tubulin/ GAPDH normalized to control (1)	**1**	**2**	**3**	**4**	**5**	**6**	**7**	**8**
	1	0.80027005	0.23201888	0.123819852	0.21816259	0.81366556	0.16113688	0.70747627

Representative uncropped western blot is presented with protein of interest directed with a red arrow and lanes marked 1-8 in red, on the chemiluminescence blot. Representative fluorescent western blot could not be detected with fault in lasers 600 and 700 of Odyssey® FC imaging system requiring replacement. Molecular marker (PageRuler Prestained Protein Ladder (Invitrogen #26617)) was still loaded to the left of the gel and can be observed in β-tubulin chemiluminescence blot. Protein markers β-tubulin and GAPDH were matched for confirmation with the molecular marker and antibodies reference in Table S7, given previous observational knowledge of GAPDH fluorescent blots.

C

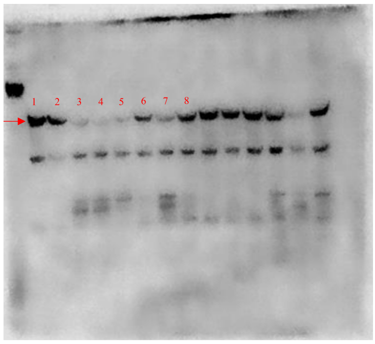

***α*-tubulin**

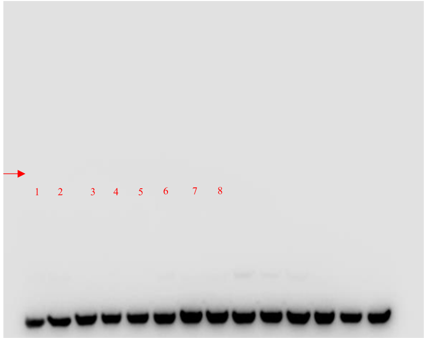

GAPDH

α-tubulin/ GAPDH normalized to control (1)	**1**	**2**	**3**	**4**	**5**	**6**	**7**	**8**
	1	0.82006592	0.09086078	0.067885741	0.049057164	0.40539325	0.15181101	0.57191859

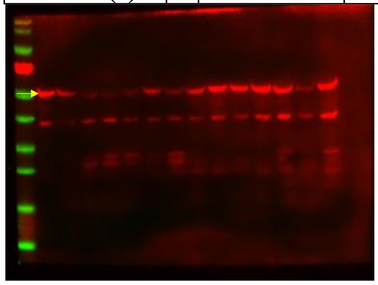

***α*-tubulin**

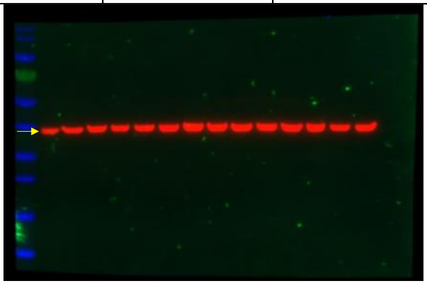

GAPDH

Representative uncropped western blot is presented with protein of interest directed with a red arrow and lanes marked 1-8 in red, on the chemiluminescence blot. Representative fluorescent western blot with molecular marker (PageRuler Prestained Protein Ladder (Invitrogen #26617)) was detected using laser 600 and 700 on the Odyssey® FC imaging system and protein of interest directed with a yellow arrow.

Figure S28. Full representative western blot of microtubule cytoskeleton protein markers in HT29. Represented data normalized to GAPDH relative to control (lane 1). A. β-actin. B. β-tubulin C. α-tubulin. Protein expression upon treatment with platinum(II) (**Pt^{II}PHEN*SS*** (lane 3), **Pt^{II}5ME*SS*** (lane 4) and **Pt^{II}56ME*SS*** (lane 5)) and platinum(IV) (**Pt^{II}PHEN*SS*(OH)$_2$** (lane 6), **Pt^{IV}5ME*SS*(OH)$_2$** (lane 7) and **Pt^{IV}56ME*SS*(OH)$_2$** (lane 8)) complexes, as well as cisplatin (lane 2) in HT29 cells at 72 h compared with control (lane 1).

A

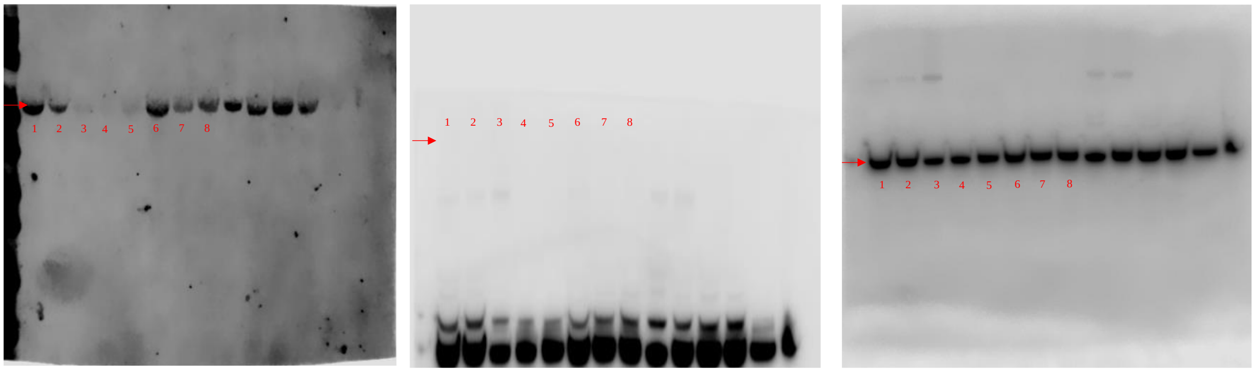

p-AKT **AKT** **GAPDH**

p-AKT/AKT /GAPDH normalized to control (1)	**1**	**2**	**3**	**4**	**5**	**6**	**7**	**8**
	1	0.28804206	0.09218709	0.07337685	0.53591585	1.01113631	0.42486161	0.72090759

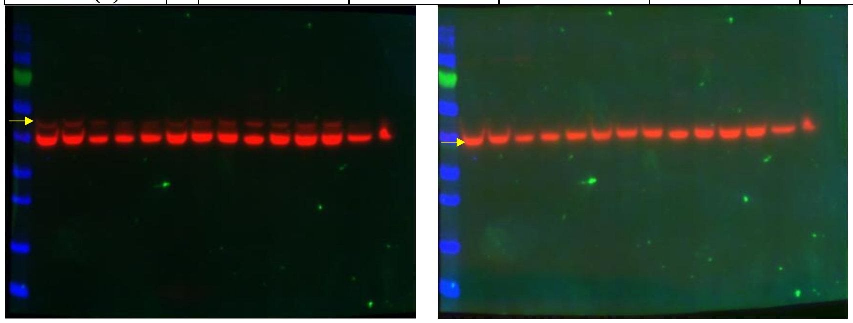

AKT **GAPDH**

Representative uncropped western blot is presented with protein of interest directed with a red arrow and lanes marked 1-8 in red, on the chemiluminescence blot. Representative fluorescent western blot of AKT and GAPDH with molecular marker (PageRuler Prestained Protein Ladder (Invitrogen #26617)) was detected using laser 600 and 700 on the Odyssey® FC imaging system and protein of interest directed with a yellow arrow. p-AKT molecular marker can be seen on the chemiluminescence blot and tracked using the AKT fluorescent blot, given p-AKT will fall at the same molecular weight.

B

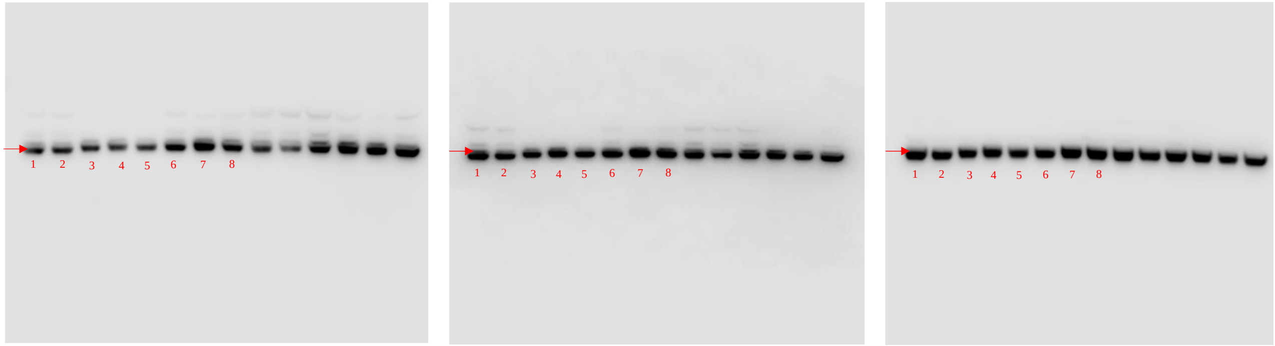

p-ERK **ERK** **GAPDH**

p-ERK/ERK /GAPDH normalized to control (1)	1	2	3	4	5	6	7	8
	1	1.1248135	0.95088206	0.70761589	0.83252105	1.22923095	1.2973034	1.28018524

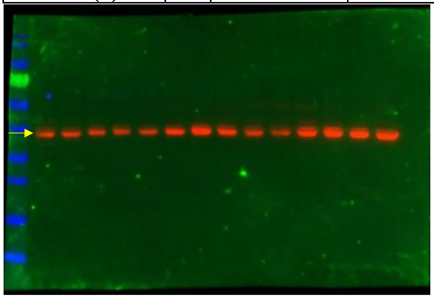

ERK

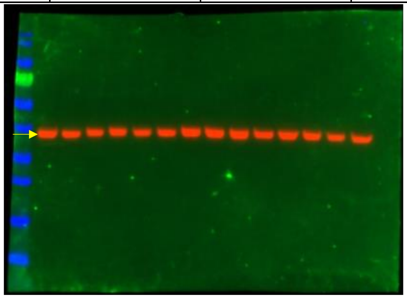

GAPDH

Representative uncropped western blot is presented with protein of interest directed with a red arrow and lanes marked 1-8 in red, on the chemiluminescence blot. Representative fluorescent western blot of ERK and GAPDH with molecular marker (PageRuler Prestained Protein Ladder (Invitrogen #26617)) was detected using laser 600 and 700 on the Odyssey® FC imaging system and protein of interest directed with a yellow arrow. p-ERK molecular marker can be seen on the chemiluminescence blot and tracked using the ERK fluorescent blot, given p-ERK will fall at the same molecular weight.

C

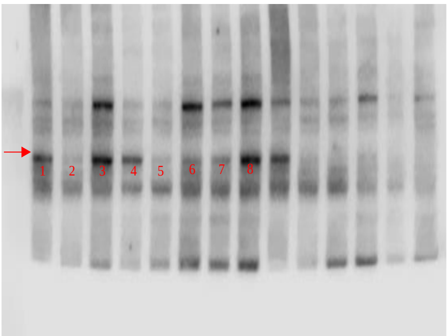

p53

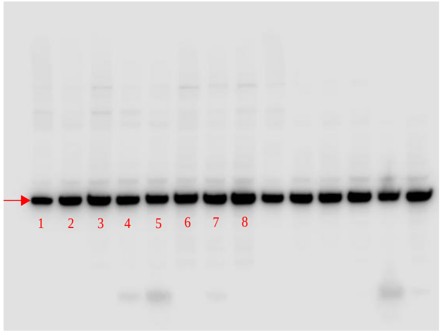

GAPDH

p53/GAPDH normalized to control (1)	1	2	3	4	5	6	7	8
	1	0.95073342	4.79055651	1.27530306	1.01424683	4.54269273	3.34882757	5.0467805

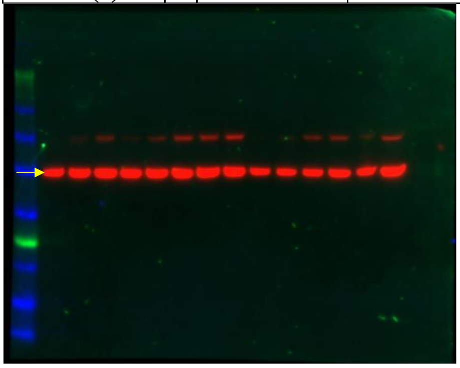

GAPDH

Representative uncropped western blot is presented with protein of interest directed with a red arrow and lanes marked 1-8 in red, on the chemiluminescence blot. Representative fluorescent western blot of GAPDH with molecular marker (SeeBlue Prestained Protein Standard (Invitrogen #LC5625)) was detected using laser 600 and 700 on the Odyssey® FC imaging system and protein of interest directed with a yellow arrow. p53 molecular marker can be tracked on the

chemiluminescence blot using the GAPDH fluorescent blot, given p53 will fall above GAPDH as slightly detected on the fluorescent blot.

D

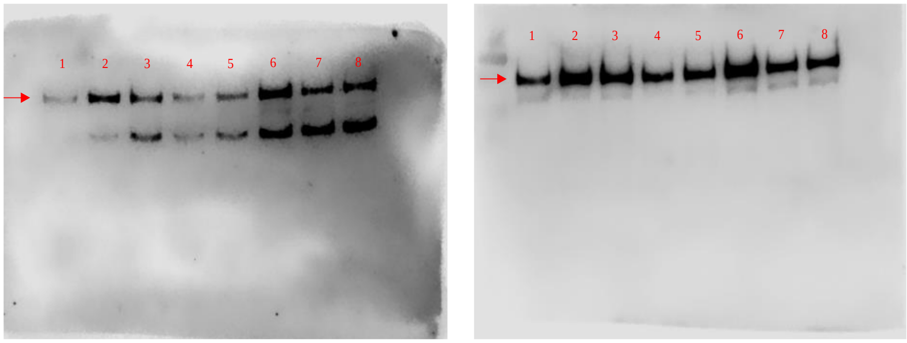

p21 **GAPDH**

	**1**	**2**	**3**	**4**	**5**	**6**	**7**	**8**
p21/GAPDH normalized to control (1)	1	2.96395985	3.17969746	1.14677595	1.28135232	3.2931754	2.65845744	2.80816882

Representative uncropped western blot is presented with protein of interest directed with a red arrow and lanes marked 1-8 in red, on the chemiluminescence blot. Representative fluorescent western blot could not be detected with fault in lasers 600 and 700 of Odyssey® FC imaging system requiring replacement. Molecular marker (PageRuler Prestained Protein Ladder (Invitrogen #26617)) was still loaded to the left of the gel and can still be observed in GAPDH chemiluminescent blot. Protein markers p21 and GAPDH were matched for confirmation with the molecular marker and antibodies reference in Table S7, given previous observational knowledge of GAPDH fluorescent blots.

Figure S29. Full representative western blot of cell proliferation protein markers in HT29. Represented data normalized to GAPDH relative to control (lane 1). A.p-AKT/AKT, B. p-ERK/ERK, C. p53 and D. p21. Protein expression upon treatment with platinum(II) (**Pt^{II}PHEN*SS*** (lane 3), **Pt^{II}5ME*SS*** (lane 4) and **Pt^{II}56ME*SS*** (lane 5)) and platinum(IV) (**Pt^{II}PHEN*SS*(OH)$_2$** (lane 6), **Pt^{IV}5ME*SS*(OH)$_2$** (lane 7) and **Pt^{IV}56ME*SS*(OH)$_2$** (lane 8)) complexes, as well as cisplatin (lane 2) in HT29 cells at 72 h compared with control (lane 1).

A

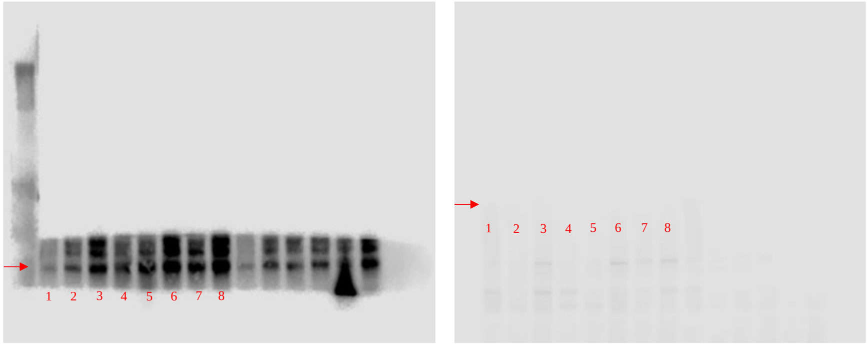

Bax **GAPDH**

	**1**	**2**	**3**	**4**	**5**	**6**	**7**	**8**
Bax/GAPDH normalized to control (1)	1	1.8099396	3.40723107	1.62402021	2.69316916	3.3881979	3.07538421	3.49768802

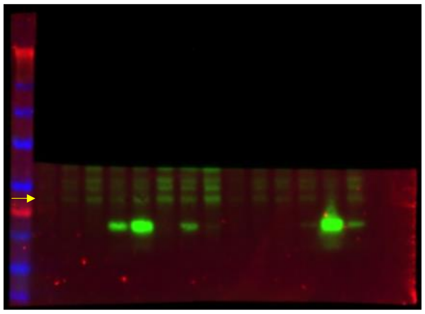

Bax

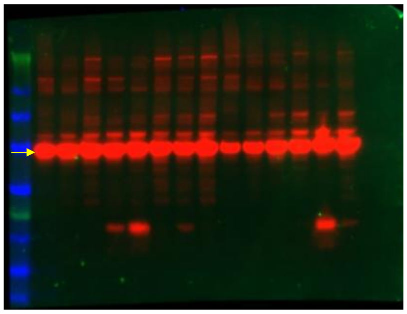

GAPDH

Representative uncropped western blot is presented with protein of interest directed with a red arrow and lanes marked 1-8 in red, on the chemiluminescence blot. Representative fluorescent western blot with molecular marker (SeeBlue Prestained Protein Standard (Invitrogen #LC5625)) was detected using laser 600 and 700 on the Odyssey® FC imaging system and protein of interest directed with a yellow arrow.

B

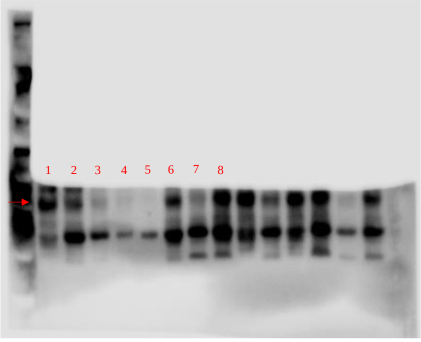

Bcl2

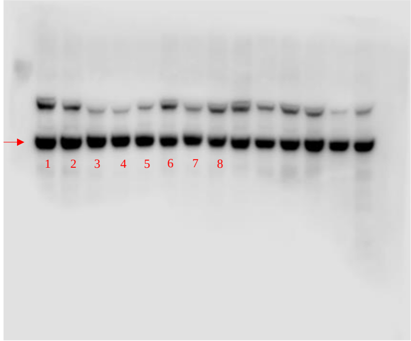

GAPDH

Bcl2/GAPDH normalized to control (1)	**1**	**2**	**3**	**4**	**5**	**6**	**7**	**8**
	1	0.56594514	0.28723981	0.09591015	0.04575617	1.19447984	0.43808048	1.03376818

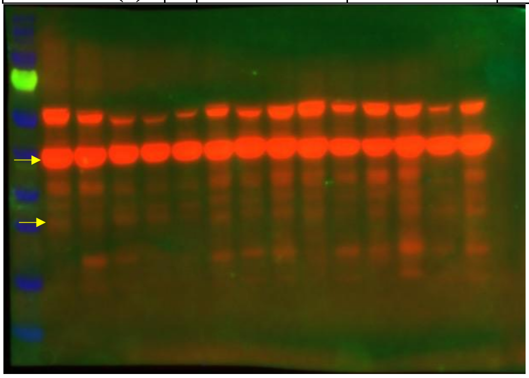

Bcl2 -top
GAPDH -bottom

Representative uncropped western blot is presented with protein of interest directed with a red arrow and lanes marked 1-8 in red, on the chemiluminescence blot. Representative fluorescent western blot with molecular marker (PageRuler Prestained Protein Ladder (Invitrogen #26617)) was detected using laser 600 and 700 on the Odyssey® FC imaging system and protein of interest directed with a yellow arrow.

C

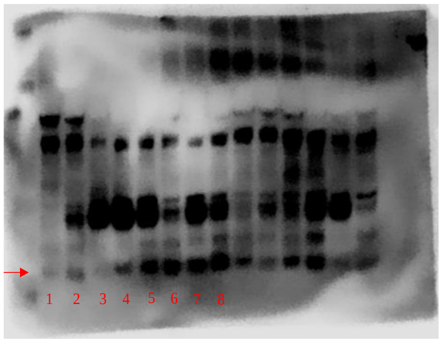

Cyt C

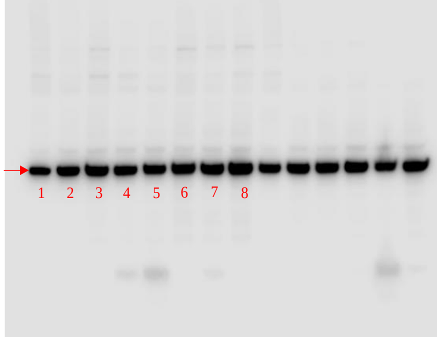

GAPDH

Cyt C /GAPDH normalized to control (1)	**1**	**2**	**3**	**4**	**5**	**6**	**7**	**8**
	1	2.41871468	1.69154667	3.74101488	6.70558815	7.0905811	6.1838904	7.66289382

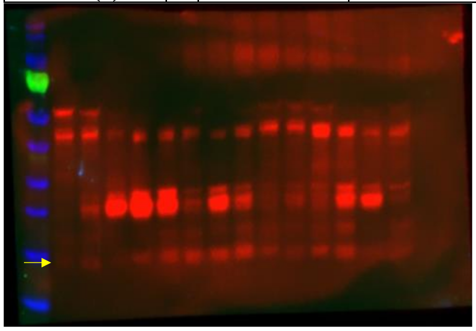

Cyt C

Representative uncropped western blot is presented with protein of interest directed with a red arrow and lanes marked 1-8 in red, on the chemiluminescence blot. Representative fluorescent western blot with molecular marker (PageRuler Prestained Protein Ladder (Invitrogen #26617)) was detected using laser 600 and 700 on the Odyssey® FC imaging system and protein of interest directed with a yellow arrow.

D

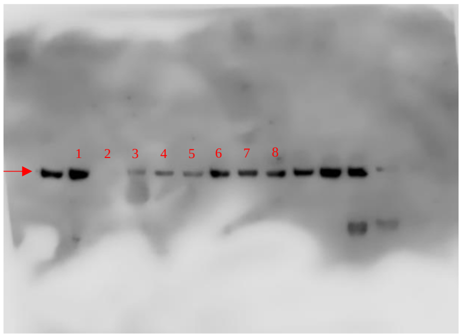

Procaspase 8

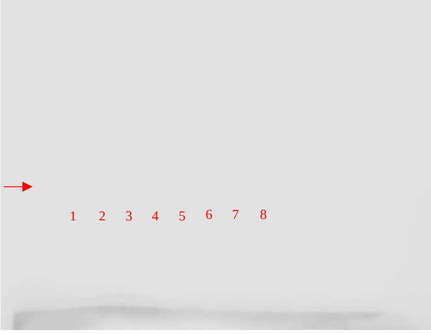

GAPDH

Procaspase 8 /GAPDH normalized to control (1)	**1**	**2**	**3**	**4**	**5**	**6**	**7**	**8**
	1	0.00402925	0.16334698	0.29343619	0.25047865	0.94737332	0.43718733	0.40350856

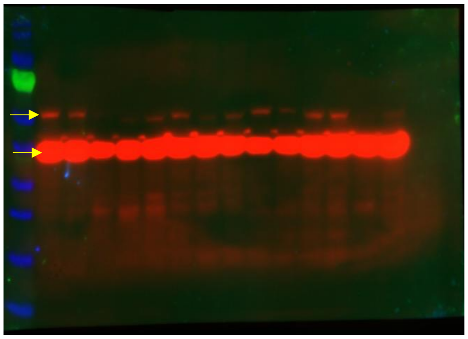

Procaspase 8 -top
GAPDH -bottom

Representative uncropped western blot is presented with protein of interest directed with a red arrow and lanes marked 1-8 in red, on the chemiluminescence blot. Representative fluorescent western blot with molecular marker (PageRuler Prestained Protein Ladder (Invitrogen #26617)) was detected using laser 600 and 700 on the Odyssey® FC imaging system and protein of interest directed with a yellow arrow.

E

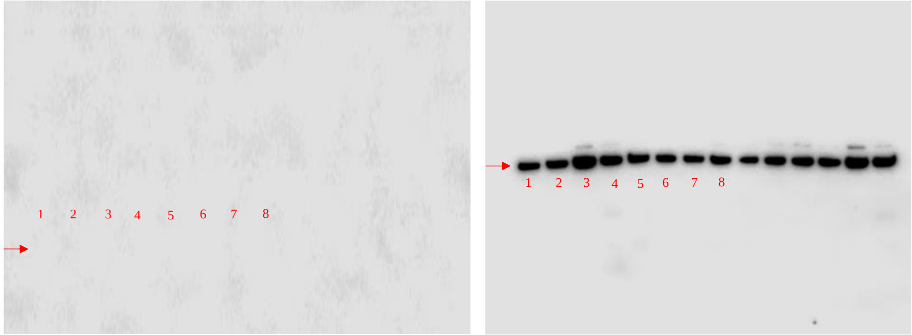

Cleaved BID **GAPDH**

Cleaved BID /GAPDH normalized to control (1)	**1**	**2**	**3**	**4**	**5**	**6**	**7**	**8**
	1	2.52691567	1.61137382	1.68964872	2.82106515	2.7111347	2.71169915	2.65145596

Representative uncropped western blot is presented with protein of interest directed with a red arrow and lanes marked 1-8 in red, on the chemiluminescence blot. Representative fluorescent western blot could not be detected with fault in lasers 600 and 700 of Odyssey® FC imaging system requiring replacement. Molecular marker (PageRuler Prestained Protein Ladder (Invitrogen #26617)) was still loaded to the left of the gel. Protein markers cleaved BID and GAPDH were matched for confirmation with the molecular marker and antibodies reference in Table S7, given previous observational knowledge of GAPDH fluorescent blots.

F

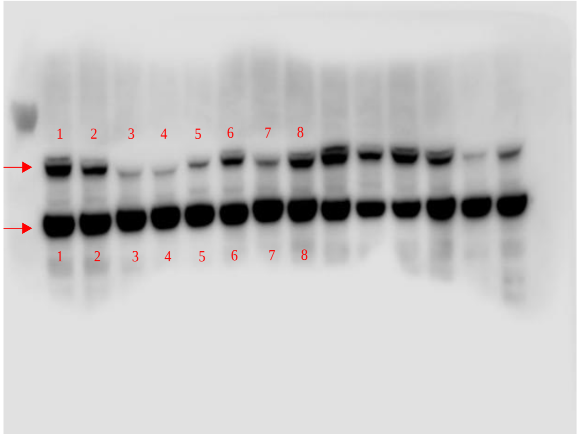

Procaspase 9 -top

GAPDH -bottom

Procaspase 9 /GAPDH normalized to control (1)	**1**	**2**	**3**	**4**	**5**	**6**	**7**	**8**
	1	0.63876707	0.19809129	0.13933932	0.27172406	0.59273615	0.20260478	0.49638347

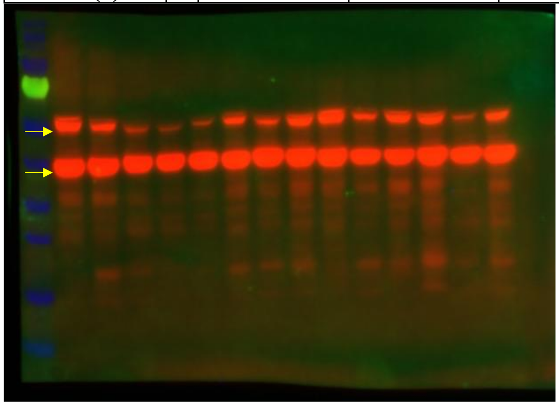

Procaspase 9 -top
GAPDH -bottom

Representative uncropped western blot is presented with protein of interest directed with a red arrow and lanes marked 1-8 in red, on the chemiluminescence blot. Representative fluorescent western blot with molecular marker (PageRuler Prestained Protein Ladder (Invitrogen #26617)) was detected using laser 600 and 700 on the Odyssey® FC imaging system and protein of interest directed with a yellow arrow.

G

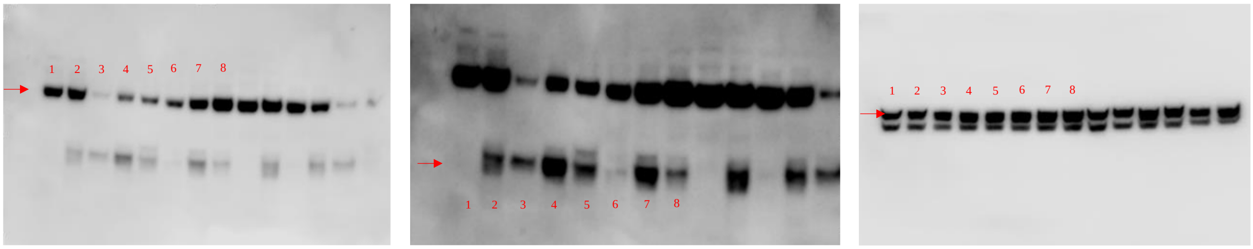

Procaspase 3 **Cleaved Caspase 3** **GAPDH**

	**1**	**2**	**3**	**4**	**5**	**6**	**7**	**8**
Procaspase 3 /GAPDH normalized to control (1)	1	1.20012485	0.48743248	0.76777954	0.89362428	1.14123935	1.23755226	1.29742903

Cleaved caspase 3 /GAPDH normalized to control (1)	1	11.8149621	17.4647582	10.8813144	5.09893053	1.01684366	7.8873937	1.74578195

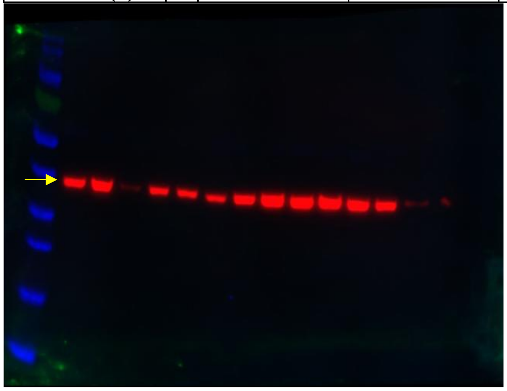

Procaspase 3

Representative uncropped western blot is presented with protein of interest directed with a red arrow and lanes marked 1-8 in red, on the chemiluminescence blot. Representative fluorescent western blot with molecular marker (PageRuler Prestained Protein Ladder (Invitrogen #26617)) was detected using laser 600 and 700 on the Odyssey® FC imaging system and protein of interest directed with a yellow arrow. While cleaved caspase 3 and GAPDH were not detected in the fluorescent blot, they were both manually confirmed and estimated with reference to the procaspase 3 and known antibodies reference in Table S7.

H

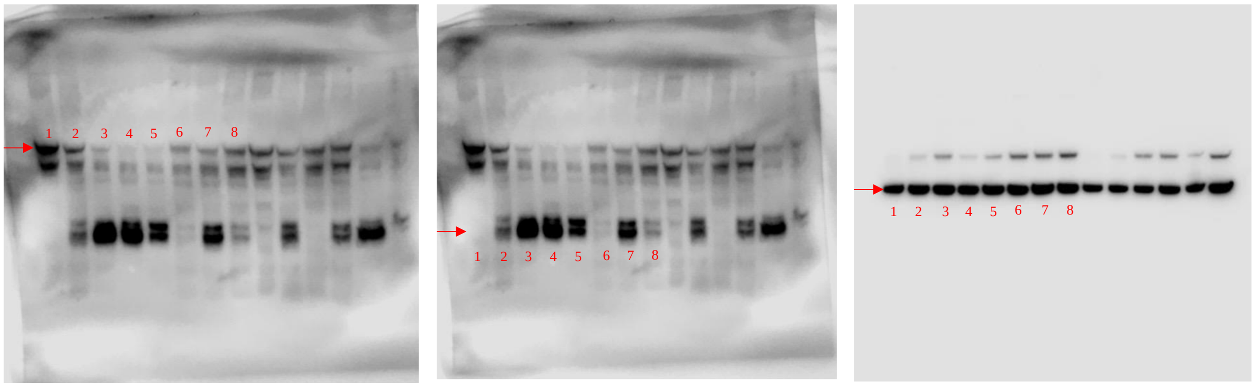

PARP-1 **GAPDH**

	1	2	3	4	5	6	7	8
PARP-1 /GAPDH normalized to control (1)	1	0.82364919	0.26115596	0.25541081	0.31700455	0.64751417	0.62066593	0.71718936
Cleaved PARP-1 /GAPDH normalized to control (1)	1	3.4836068	8.49620931	11.420335	7.0644866	2.23930102	5.7612047	2.7242088

Representative uncropped western blot is presented with protein of interest directed with a red arrow and lanes marked 1-8 in red, on the chemiluminescence blot. Representative fluorescent western blot could not be detected with fault in lasers 600 and 700 of Odyssey® FC imaging system requiring replacement. Molecular marker (PageRuler Prestained Protein Ladder (Invitrogen #26617)) was still loaded to the left of the gel. Protein markers PARP-1 and GAPDH were matched for confirmation with the molecular marker and antibodies reference in Table S7, given previous observational knowledge of GAPDH fluorescent blots.

Figure S30. Full representative western blot of intrinsic and extrinsic apoptotic cell death markers in HT29. Represented data normalized to GAPDH relative to control (lane 1). A. Bax, B. Bcl2, C. Cyt C, D.Procaspase 8, E. Cleaved BID, F. Procaspase 9, G. Procaspase 3 and H. PARP-1. Protein expression upon treatment with platinum(II) (**Pt^{II}PHEN*SS*** (lane 3), **Pt^{II}5ME*SS*** (lane 4) and **Pt^{II}56ME*SS*** (lane 5)) and platinum(IV) (**Pt^{II}PHEN*SS*(OH)$_2$** (lane 6), **Pt^{IV}5ME*SS*(OH)$_2$** (lane 7) and **Pt^{IV}56ME*SS*(OH)$_2$** (lane 8)) complexes, as well as cisplatin (lane 2) in HT29 cells at 72 h compared with control (lane 1).

A

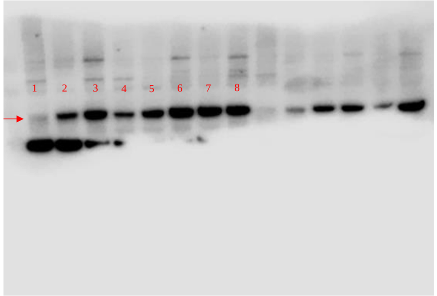

Beclin1

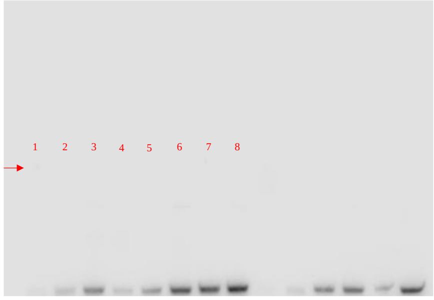

GAPDH

Beclin1 /GAPDH normalized to control (1)	1	2	3	4	5	6	7	8
	1	3.88961086	5.32405666	3.10952730	5.63173026	8.0058431	6.94268267	6.22185923

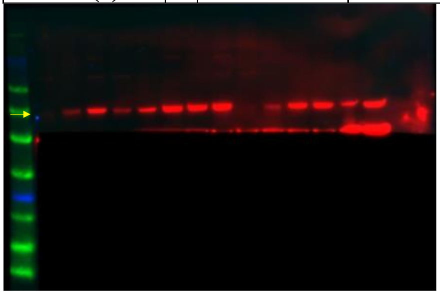

Beclin1

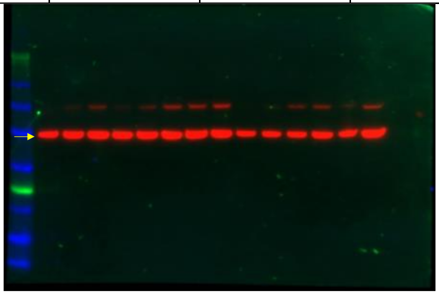

GAPDH

Representative uncropped western blot is presented with protein of interest directed with a red arrow and lanes marked 1-8 in red, on the chemiluminescence blot. Representative fluorescent western blot with molecular marker (SeeBlue Prestained Protein Standard (Invitrogen #LC5625)) was detected using laser 600 and 700 on the Odyssey® FC imaging system and protein of interest directed with a yellow arrow.

B

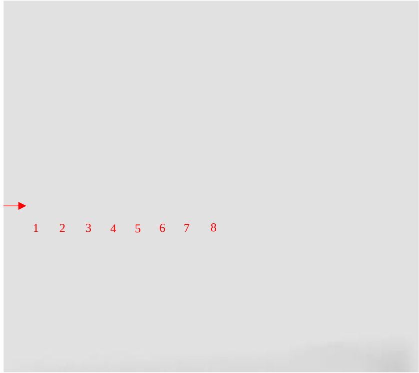

APG5L/ATG5

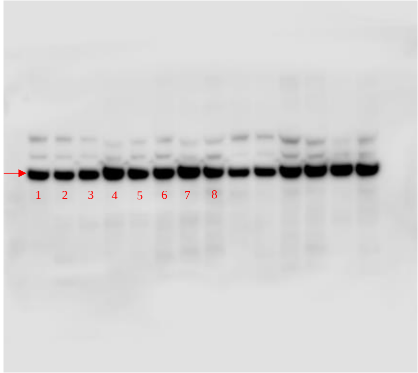

GAPDH

ATG5 /GAPDH normalized to control (1)	1	2	3	4	5	6	7	8
	1	1.1927417	0.94276752	0.99689099	0.61852183	0.31017834	0.23640377	0.32207934

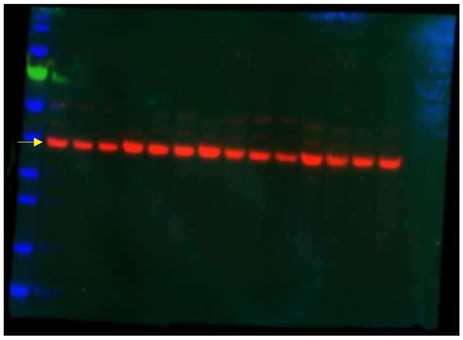

GAPDH

Representative uncropped western blot is presented with protein of interest directed with a red arrow and lanes marked 1-8 in red, on the chemiluminescence blot. Representative fluorescent western blot with molecular marker (SeeBlue Prestained Protein Standard (Invitrogen #LC5625)) was detected using laser 600 and 700 on the Odyssey® FC imaging system and protein of interest directed with a yellow arrow. While APG5/ATG5 fluorescent blot was not detected, its fluorescent bands fall at the same level as GAPDH. We observed two band rows above the APG5/ATG5 in the chemiluminescent blot and the same bands can be seen above GAPDH in the fluorescence blot. This confirms the correct observation of APG5/ATG5 at its correct molecular marker.

C

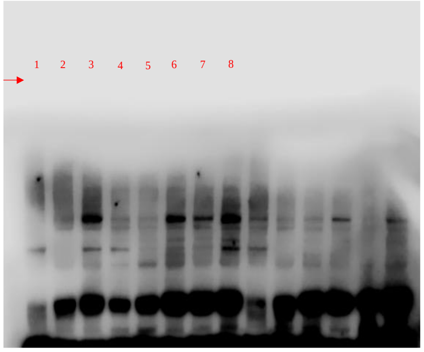

ATG16L1

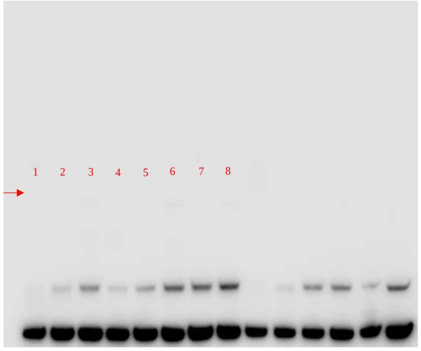

GAPDH

ATG16L1 /GAPDH normalized to control (1)	**1**	**2**	**3**	**4**	**5**	**6**	**7**	**8**
	1	1.173333	3.84385	1.2845093	1.309065	4.092945	2.006203	4.523285

Representative uncropped western blot is presented with protein of interest directed with a red arrow and lanes marked 1-8 in red, on the chemiluminescence blot. Representative fluorescent western blot could not be detected with fault in lasers 600 and 700 of Odyssey® FC imaging system requiring replacement. Molecular marker (PageRuler Prestained Protein Ladder (Invitrogen #26617)) was still loaded to the left of the gel. Protein markers ATG16L1 and GAPDH were matched for confirmation with the molecular marker and antibodies reference in Table S7, given previous observational knowledge of GAPDH fluorescent blots.

D

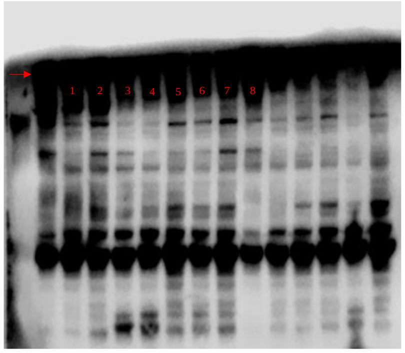

ATG9A

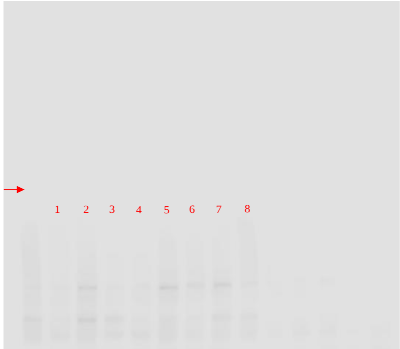

GAPDH

ATG9A /GAPDH normalized to control (1)	1	2	3	4	5	6	7	8
	1	2.21216263	0.46496635	0.44859831	1.51595621	1.20296996	2.33441205	0.90437915

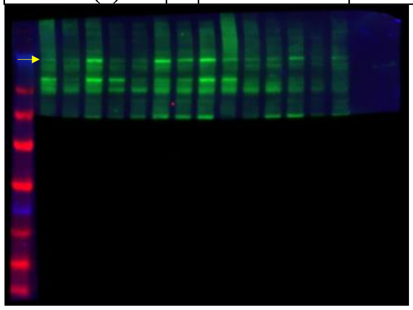

ATG9A

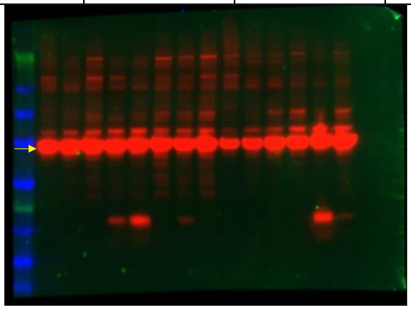

GAPDH

Representative uncropped western blot is presented with protein of interest directed with a red arrow and lanes marked 1-8 in red, on the chemiluminescence blot. Representative fluorescent western blot with molecular marker (SeeBlue Prestained Protein Standard (Invitrogen #LC5625)) was detected using laser 600 and 700 on the Odyssey® FC imaging system and protein of interest directed with a yellow arrow.

E

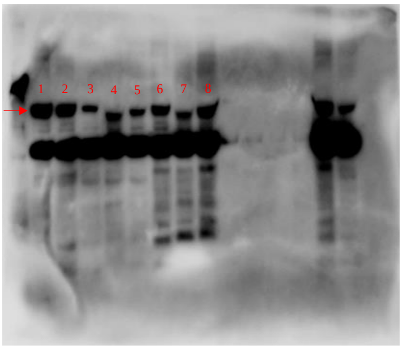

ATG4B

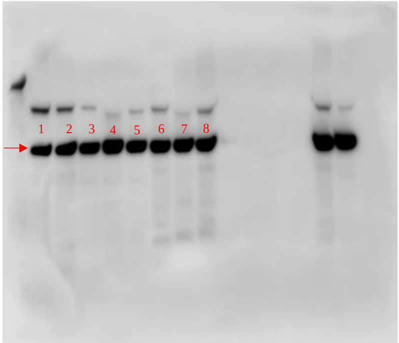

GAPDH

ATG4B /GAPDH normalized to control (1)	1	2	3	4	5	6	7	8
	1	1.10064684	0.68440434	0.73063446	0.71064903	0.90489986	0.5921638	0.92231009

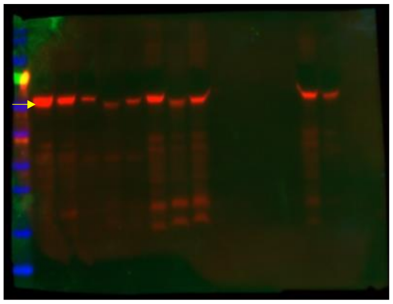

ATG4B

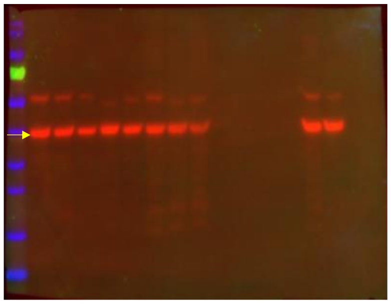

GAPDH

Representative uncropped western blot is presented with protein of interest directed with a red arrow and lanes marked 1-8 in red, on the chemiluminescence blot. Representative fluorescent western blot with molecular marker (PageRuler Prestained Protein Ladder (Invitrogen #26617)) was detected using laser 600 and 700 on the Odyssey® FC imaging system and protein of interest directed with a yellow arrow.

F

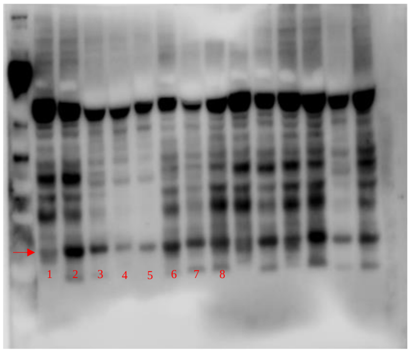

LC3B

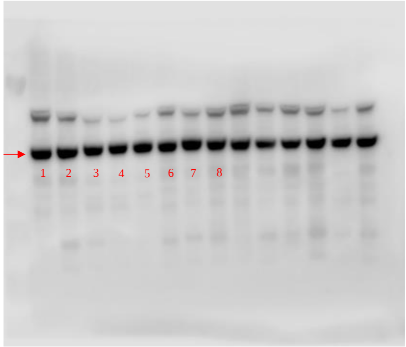

GAPDH

LC3B /GAPDH normalized to control (1)	**1**	**2**	**3**	**4**	**5**	**6**	**7**	**8**
	1	3.23330908	2.12095134	1.20741902	1.04367288	2.23203726	1.71266931	1.97638983

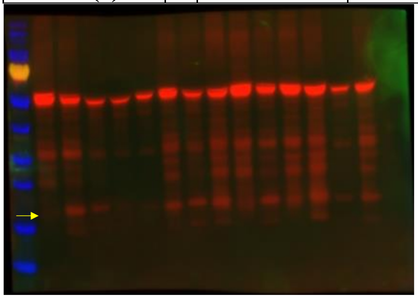

LC3B

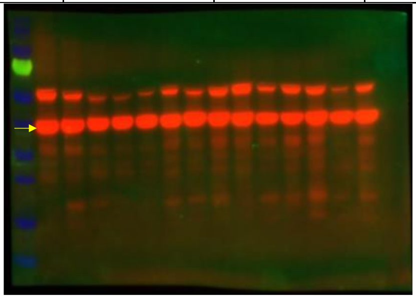

GAPDH

Representative uncropped western blot is presented with protein of interest directed with a red arrow and lanes marked 1-8 in red, on the chemiluminescence blot. Representative fluorescent western blot with molecular marker (PageRuler Prestained Protein Ladder (Invitrogen #26617)) was detected using laser 600 and 700 on the Odyssey® FC imaging system and protein of interest directed with a yellow arrow.

Figure S31. Full representative western blot of autophagy markers in HT29. Represented data normalized to GAPDH relative to control (lane 1). A. Beclin1, B. APG5L/ATG5, C. ATG16L1, D. ATG9A, E. ATG4B and F. LC3B. Protein expression upon treatment with platinum(II) (**Pt^{II}PHEN*SS*** (lane 3), **Pt^{II}5ME*SS*** (lane 4) and **Pt^{II}56ME*SS*** (lane 5)) and platinum(IV) (**Pt^{II}PHEN*SS*(OH)$_2$** (lane 6), **Pt^{IV}5ME*SS*(OH)$_2$** (lane 7) and **Pt^{IV}56ME*SS*(OH)$_2$** (lane 8)) complexes, as well as cisplatin (lane 2) in HT29 cells at 72 h compared with control (lane 1).

References:

1. Aputen, A.D.; Elias, M.G.; Gilbert, J.; Sakoff, J.A.; Gordon, C.P.; Scott, K.F.; Aldrich-Wright, J.R. Potent Chlorambucil-Platinum(IV) Prodrugs. *Int J Mol Sci* **2022**, *23*, doi:10.3390/ijms231810471.
2. Deo, K.M.; Sakoff, J.; Gilbert, J.; Zhang, Y.; Aldrich Wright, J.R. Synthesis, characterisation and potent cytotoxicity of unconventional platinum(iv) complexes with modified lipophilicity. *Dalton Trans* **2019**, *48*, 17217-17227, doi:10.1039/c9dt03339d.
3. Macias, F.J.; Deo, K.M.; Pages, B.J.; Wormell, P.; Clegg, J.K.; Zhang, Y.; Li, F.; Zheng, G.; Sakoff, J.; Gilbert, J.; et al. Synthesis and Analysis of the Structure, Diffusion and Cytotoxicity of Heterocyclic Platinum(IV) Complexes. *Chemistry* **2015**, *21*, 16990-17001, doi:10.1002/chem.201502159.
4. Khoury, A.; Sakoff, J.A.; Gilbert, J.; Scott, K.F.; Karan, S.; Gordon, C.P.; Aldrich-Wright, J.R. Cyclooxygenase-Inhibiting Platinum(IV) Prodrugs with Potent Anticancer Activity. *Pharmaceutics* **2022**, *14*, doi:10.3390/pharmaceutics14040787.
5. Harper, B.W.J.P., E.; Sirota, R.; Faccioli, F.F.; Aldrich-Wright, J.R.; Gandin, V.; Gibson, D. . Synthesis, characterization and in vitro and in vivo anticancer activity of Pt(iv) derivatives of [Pt(1S,2S-DACH)(5,6-dimethyl-1,10-phenanthroline)]. . *Dalton Transactions* **2017**, *46*, 7005–7019.
